# Towards conversational artificial intelligence for disease management

**DOI:** 10.1038/s41586-026-10764-5

**Published:** 2026-06-17

**Authors:** Valentin Liévin, Anil Palepu, Wei-Hung Weng, Khaled Saab, David Stutz, Yong Cheng, Kavita Kulkarni, S. Sara Mahdavi, Joëlle Barral, Dale R. Webster, Katherine Chou, Avinatan Hassidim, Yossi Matias, James Manyika, Ryutaro Tanno, Vivek Natarajan, Adam Rodman, Tao Tu, Alan Karthikesalingam, Mike Schaekermann

**Affiliations:** 1 Google DeepMind, Mountain View, CA USA; 2 Google Research, Mountain View, CA USA

**Keywords:** Health care, Diseases

## Abstract

Although large language models have shown promise in diagnostic dialogue^1^, their capabilities for effective management reasoning, including disease progression, therapeutic response and safe medication prescription, have remained underexplored. We have advanced the previously demonstrated diagnostic capabilities of the Articulate Medical Intelligence Explorer (AMIE)^1–3^ using a new large-language-model-based agentic system optimized for multivisit clinical management and dialogue. To ground the reasoning of AMIE in authoritative clinical knowledge, we leveraged the long-context capabilities of Gemini^4^, combining in-context retrieval with structured reasoning to align its output with up-to-date clinical practice guidelines and drug formularies. In a randomized, blinded virtual Objective Structured Clinical Examination study, AMIE was compared to 21 primary care physicians (PCPs) across 100 multivisit case scenarios designed to reflect the guidance of the UK National Institute for Health and Care Excellence and BMJ Best Practice guidelines. AMIE was non-inferior to PCPs in management reasoning, as assessed by specialists, and scored better both with respect to preciseness of treatment and investigation, and in terms of its alignment with and grounding in clinical guidelines. To benchmark medication reasoning, we developed RxQA, a multiple-choice question benchmark that was derived from two national drug formularies (from the USA and UK) and validated by board-certified pharmacists. Although AMIE and PCPs both benefited from the ability to access external drug information, AMIE outperformed PCPs on higher-difficulty questions. Although further research will be needed before real-world translation of AMIE, its strong performance across evaluations marks a significant step towards use of conversational artificial intelligence as a tool in disease management.

## Abstract (web summary)

Advances to the artificial intelligence system AMIE enable it to manage disease across multiple visits while reasoning over a corpus of clinical guidelines and patient history, outperforming primary care physicians on management reasoning tasks.

## Main

There have been rapid advances with respect to the potential clinical applications of large language models (LLMs), with studies demonstrating not only that these models can render accurate differential diagnoses when given curated patient information, but that LLM-based artificial intelligence (AI) systems can actively collect medical history through conversations in a natural empathetic style that builds trust and rapport^1,3,5,6^. This process, which can be described as clinical history-taking and diagnostic reasoning, is necessary but not sufficient for providing clinical care. In addition to taking a history, physicians must select appropriate investigations for a patient, create an acceptable care plan that takes patient preferences and system constraints into account, and consider the progression of the disease and its treatment over time, including making decisions such as ‘watchful waiting’ or interval follow-up. This cognitive process, known as management reasoning, requires complex synthesis of relevant clinical guidelines, new and evolving evidence in medical literature, and local patient-specific context^7^.

Although the study of diagnostic reasoning has a long history, and there are many validated measures for both human and AI evaluation^8^, there has been comparatively little investigation of evaluation of management reasoning (Supplementary Information section 1). This is largely because management reasoning involves considerable amounts of context specificity; that is, two physicians given the same patient and information might make different decisions because of differing contextual factors^9^. Most research on management reasoning has focused on medical education; the current gold standard in assessment is the Objective Structured Clinical Evaluation (OSCE), in which a student interviews a patient actor and makes management decisions and is evaluated by means of a standardized rubric. There have been few rigorous investigations of the abilities of LLMs to perform management reasoning, and the studies that have been performed have relied on static non-conversational settings^10^.

In this paper, we detail our progress towards optimizing the capabilities of the Articulate Medical Intelligence Explorer (AMIE), an LLM-based research AI system with physician-like performance on conversational diagnostic tasks, for management reasoning over time. In this work we developed an LLM-based agentic system optimized for clinical management and dialogue, incorporating reasoning over the longitudinal evolution of disease and multiple patient visit encounters, response to therapy and professional competence in medication prescription. Using a randomized, blinded virtual OSCE study as an analogue, we compared AMIE with primary care physicians (PCPs) and evaluated their behaviour on multiple domains of management reasoning.

**Fig. 1:**
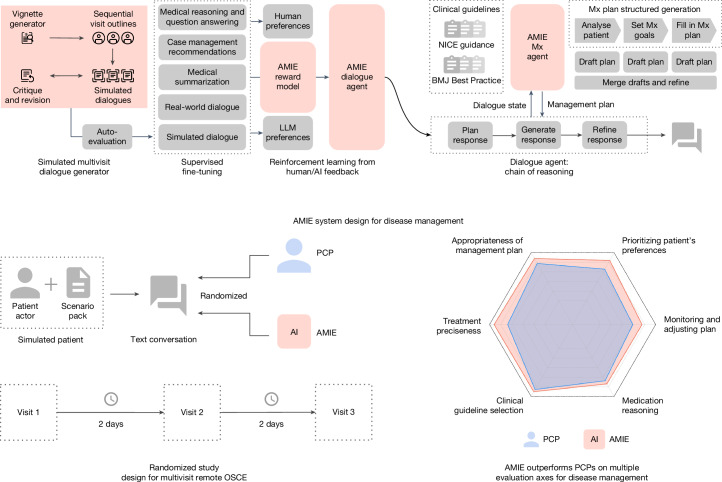
Overview of contributions. The capabilities of AMIE were advanced towards management of disease over the course of multiple visits and in concordance with authoritative knowledge in the form of clinical guidelines. AMIE leverages an agentic system incorporating a dialogue agent for conversational interaction and a management reasoning (Mx) agent for evidence-based reasoning and generation of management plans. A simulated dialogue environment enabled refinement of the capabilities of AMIE across diverse medical contexts. During online inference, AMIE uses the long-context capabilities of Gemini to reason over multiple clinical guidelines and generate a comprehensive management plan. The performance of AMIE was evaluated using a multivisit remote OSCE study, in which it was compared with 21 PCPs on the basis of *N* = 100 scenarios (3 visits per scenario).

**Fig. 2:**
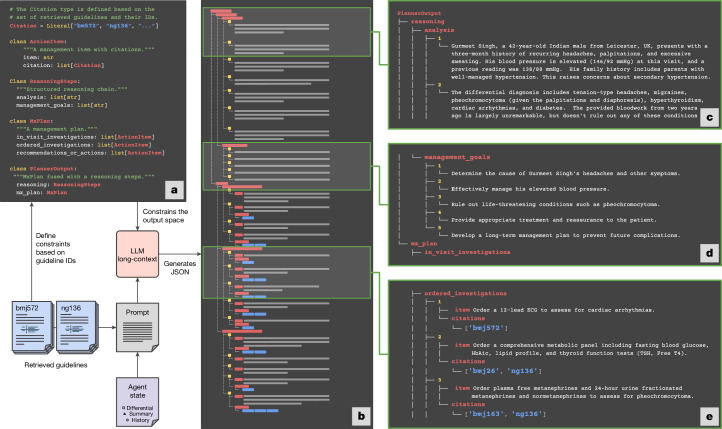
Reasoning and planning under structural constraints. Inference-time decoding constraints are applied to constrain the model output to a predefined JSON structure and sets of values. The structures are defined in Python code, generated on the basis of the set of retrieved guidelines and automatically converted into decoding constraints. The corresponding JSON schema is appended to the prompt. **a**, Target structure represented as Python code. **b**, Illustration of a reasoning trace generated using this structure, represented as a tree. **c**, generated case analysis as part of the reasoning chain. **d**, generated high-level management goals included in the reasoning chain. **e**, A section of the generated plan. Each plan item is annotated with generated references to the source documents through in-context reasoning over the set of retrieved guidelines.

**Fig. 3:**
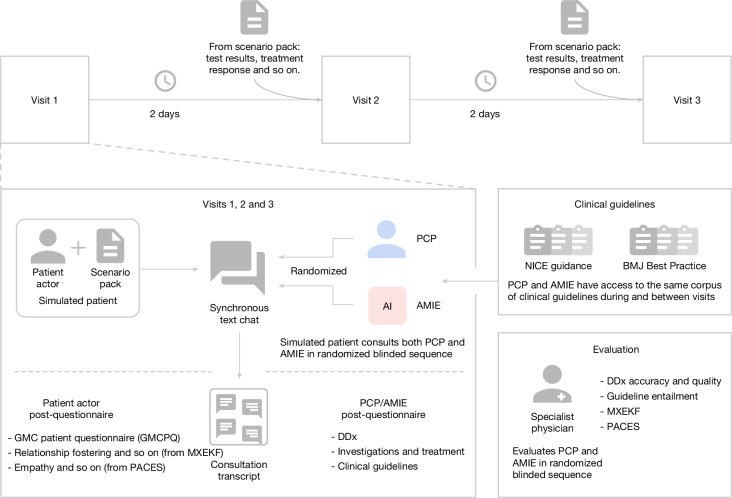
Overview of randomized study design. A PCP and AMIE performed (in randomized order) 3 virtual remote OSCE visits with 100 simulated patients through online multiturn synchronous text chat, building on an initial patient presentation with subsequent updates on symptoms, treatment responses and test results. Both the PCP and AMIE had access to a corpus of clinical guidelines. After each visit, the PCP and AMIE completed a post-questionnaire, and both were evaluated by patient actors and specialist physicians across a range of axes, including diagnostic accuracy, guideline entailment, management reasoning and clinical communication skills.

Our contributions are summarized in Figs. 1–3.

**Extended Data Fig. 1:**
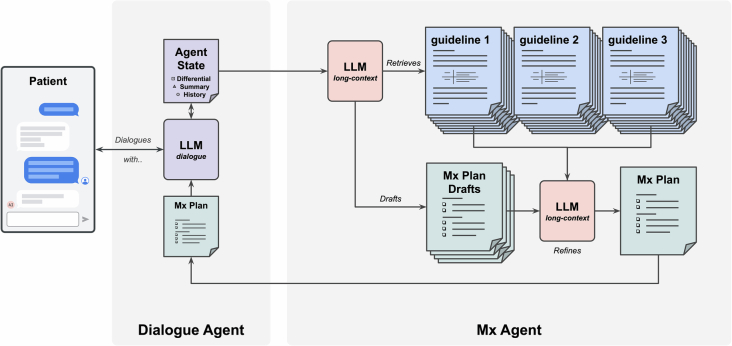
System architecture. AMIE is a symbiosis of two specialized agents: the *Dialogue Agent*, whose role is to converse with the patient to collect information and communicate clinical decisions, and the *Mx Agent*, whose role is to browse full-text clinical guidelines and compile tailored management plans, which are delivered to the Dialogue Agent.

- We advance the previously demonstrated diagnostic capabilities of AMIE through a new LLM-based agentic system (Extended Data Fig. 1) optimized for clinical management and dialogue, incorporating reasoning over the longitudinal evolution of disease and multiple patient visit encounters, response to therapy and professional competence in medication prescription. To ground the reasoning of the system in authoritative clinical knowledge, we leveraged the long-context capabilities of Gemini, combining in-context retrieval with structured reasoning to align its output with relevant and up-to-date clinical practice guidelines and drug formularies (Fig. 2).
- We conducted a randomized, blinded virtual OSCE study (Fig. 3), comparing AMIE with 21 PCPs across 100 multivisit case scenarios and five medical specialties, which were developed to involve decision-making as discussed in UK National Institute for Health and Care Excellence (NICE) guidance and BMJ Best Practice guidelines. On the basis of the OSCE interactions, specialist physicians and patient actors assessed AMIE and PCPs with respect to multiple domains of management reasoning. To benchmark medication reasoning, we used RxQA, a challenging multiple-choice question benchmark derived from two national drug formularies (from the USA and UK), which was validated by a panel of board-certified pharmacists (https://github.com/google-health/rxqa).
- We demonstrate that the management reasoning capabilities of AMIE were overall non-inferior to those of PCPs in the OSCE study, and AMIE scored better in terms of preciseness of treatment and investigation and alignment with and grounding of management plans in clinical guidelines. For medication reasoning (RxQA), whereas AMIE and PCPs both benefited significantly from the ability to access external drug information, AMIE outperformed PCPs on the subset of question rated as higher difficulty by pharmacists.

### Management plan quality

**Fig. 4:**
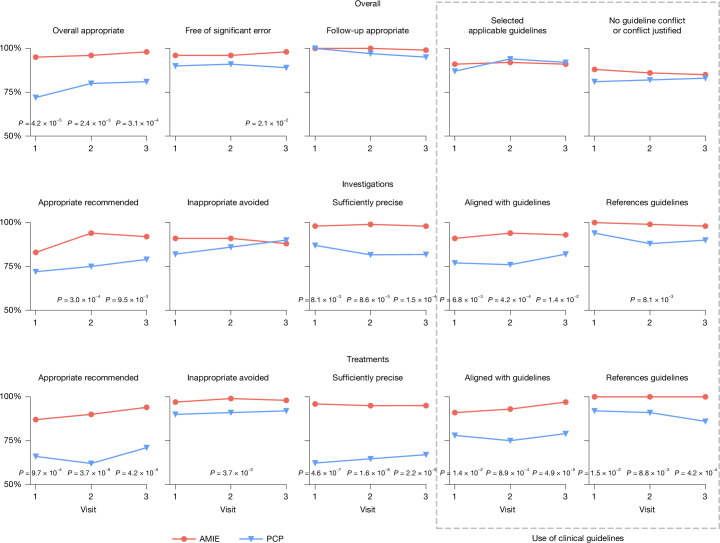
Management plan quality. The quality of management plans for each of three visits per scenario was measured as the proportion of cases in which AMIE or PCPs received favourable median ratings from a panel of three independent specialist physicians. We present overall quality criteria alongside criteria specific to investigations and treatments, respectively. For each category, we include two quality criteria addressing the use of clinical guidelines (right). Of 15 evaluation axes tested, 9 were based on yes/no ratings scales. The remaining ones were binarized using the top two options on the respective scales: ‘overall appropriate’ (five-point scale), ‘selected applicable guidelines’ (five-point scale), ‘aligned with guidelines’ (five-point scale) and ‘references guidelines’ (four-point scale). For each evaluation axis, cases with ‘NA’ ratings on either study arm were excluded for each visit. The sample size was *n* = 100 scenarios for all evaluation axes and visits, except ‘sufficiently precise’ (investigations: *n* = 100, *n* = 98, *n* = 99 for 3 visits; treatments: *n* = 98, *n* = 99, *n* = 100 for 3 visits). *P* values from two-sided McNemar’s tests are shown for all comparisons with *P* < 0.05 after false discovery rate correction.

Specialists (Supplementary Information section 11) evaluated management quality using triplicate ratings (see the interrater reliability in Supplementary Information section 10) across the 100 multivisit scenarios (Supplementary Information section 13) using the interface shown in Extended Data Fig. 6. The evaluation axes shown in Fig. 4 pertain to three categories, with five evaluation axes per category: the overall quality of the management plan, the quality of investigation recommendations and the quality of treatment recommendations. For each of the three categories, two evaluation axes specifically related to the use of clinical guidelines were included (Fig. 4, right).

#### AMIE plans non-inferior to PCPs

Across all 15 evaluation axes and 3 visits, the management plans produced by AMIE scored at least as well as those from PCPs. AMIE scored significantly higher than PCPs on appropriateness of the overall plan (95% versus 72%, *P* < 0.001, for visit 1; 96% versus 80%, *P* = 0.002, for visit 2; 98% versus 81%, *P* < 0.001, for visit 3) and recommended treatments (87% versus 66% for visit 1; 90% versus 62% for visit 2; 94% versus 71% for visit 3; all *P* < 0.001) across all three visits. The investigation recommendations by AMIE were also scored as appropriate significantly more frequently across the two follow-up visits (94% versus 75%, *P* < 0.001, for visit 2; 92% versus 79%, *P* = 0.009, for visit 3). For at least one of three visits, AMIE scored significantly higher than PCPs with respect to being free of significant errors (98% versus 89%, *P* = 0.019, for visit 3), providing appropriate follow-up recommendations (100% versus 97%, *P* < 0.001, for visit 2) and avoiding inappropriate treatments (99% versus 91%, *P* = 0.035, for visit 2). PCPs scored marginally higher than AMIE on some axes, including avoiding inappropriate investigations during follow-up visits (88% versus 90%, *P* = 0.841, for visit 3), but the differences were not statistically significant.

#### AMIE had more precise recommendations

A key strength of AMIE was its level of preciseness in recommending investigations and treatments at the end of each visit. For investigations, AMIE consistently received higher preciseness scores than PCPs for all three visits, with the gap widening during follow-up visits (98% versus 87%, *P* = 0.008, for visit 1; 99% versus. 82%, *P* < 0.001, for visit 2; 98% versus 82%, *P* < 0.001, for visit 3). The difference was more pronounced for preciseness of treatment recommendations. with large gaps across all three visits (96% versus 62% for visit 1; 95% versus 65% for visit 2; 95% versus 67% for visit 3; all *P* < 0.001).

#### AMIE had superior guideline alignment

With respect to use of clinical guidelines, AMIE and PCPs achieved similar high scores for selection of applicable guidelines, with PCPs scoring marginally though not significantly higher during follow-up visits (91% versus 87%, *P* = 0.541, for visit 1; 92% versus 94%, *P* = 0.841, for visit 2; 91% versus 92%, *P* = 1.000 for visit 3). For all three visits, AMIE received significantly higher scores for recommending treatments that were aligned with the guidelines (91% versus 87%, *P* = 0.014, for visit 1; 93% versus 75%, *P* < 0.001, for visit 2; 97% versus 79%, *P* < 0.001, for visit 3) and supported by explicit references to a guideline (100% versus 92%, *P* = 0.015, for visit 1; 100% versus 91%, *P* = 0.009, for visit 2; 100% versus 86%, *P* < 0.001, for visit 3). Similar though less consistent and less pronounced trends were observed for recommending investigations that were aligned with the guidelines (91% versus 77%, *P* = 0.007, for visit 1; 94% versus 76%, *P* < 0.001, for visit 2; 93% versus 82%, *P* = 0.014, for visit 3) and supported by explicit references to a guideline (99% versus 88%, *P* = 0.008, for visit 2).

#### Ablations of model and agents

**Extended Data Fig. 2:**
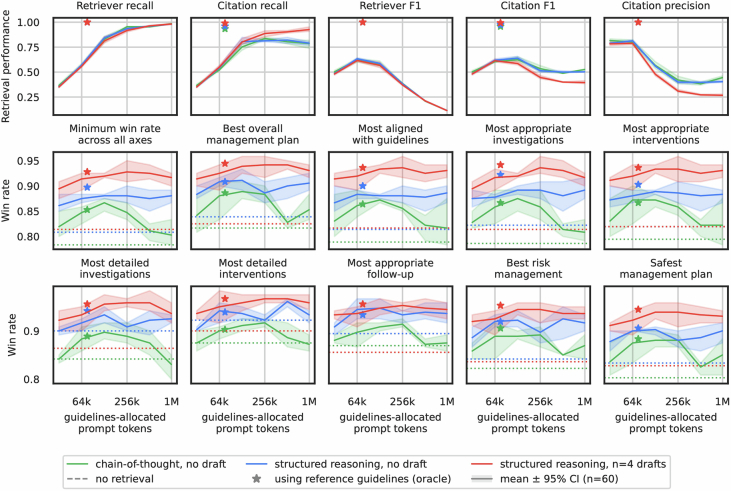
Mx agent auto-evaluation. For the selection of methods, we report validation performances aggregated over 20 evaluation scenarios and three random seeds (*n* = 60) and report bootstrapped 95% CI in shaded area centered around the mean. **Row 1**. Retrieval performances are evaluated based on the reference clinical guidelines associated with each scenario, reported based on the embedding-based retrieval step and based on the citations generated along with each management item. **Rows 2** and **3**. Management performances are measured based on the expected win-rate across 9 categories, auto-evaluated using Gemini 1.5 Pro as a judge, comparing human-written reference plans against the generated ones. We compared classic chain-of-thought (unstructured reasoning component) against our best performing reasoning structure (analyze, then state high-level goals).

**Extended Data Fig. 3:**
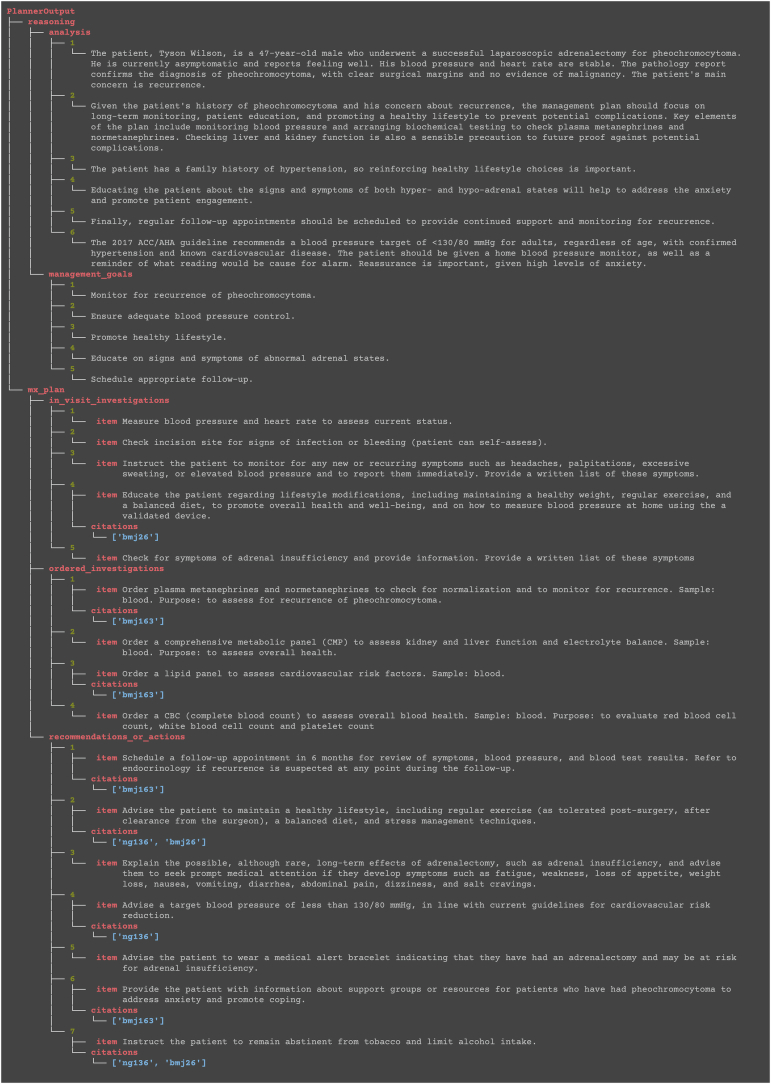
Mx Agent reasoning trace and plan. A fictive patient treated for pheochromocytoma. In this example, Mx Agent considers appropriate post-surgical follow up, including counseling about unlikely long-term side effects, while also following recommendations for blood pressure control, healthy diet, and smoking cessation. Note the imperfection that recommendations in this sample plan do not include genetic testing which could be considered appropriate in this setting.

**Extended Data Fig. 4:**
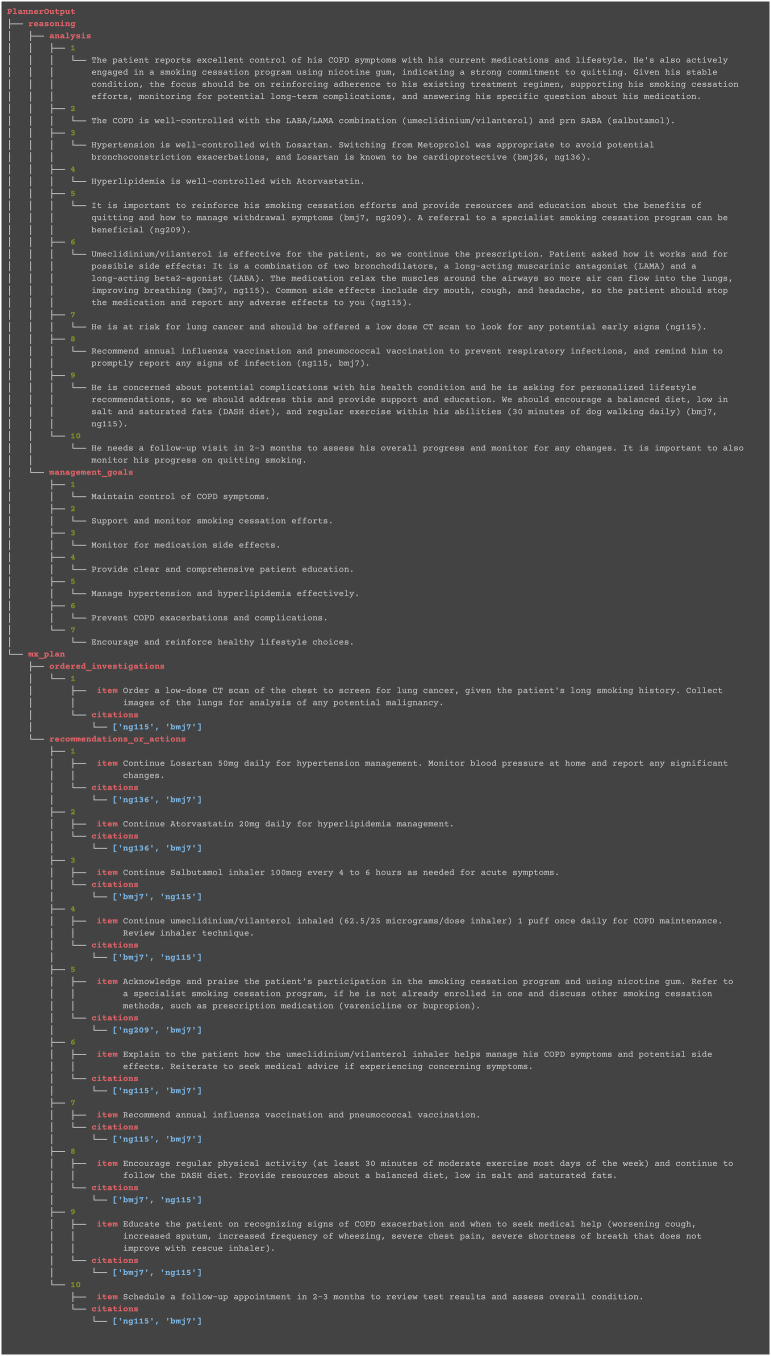
Mx Agent reasoning trace and plan. A fictive patient with COPD. In this example, Mx Agent reviews the medications for each of the patient’s chronic medical conditions, considering possible drug interactions (such as beta blockers worsening COPD symptoms). It also recommends appropriate cancer screening and vaccinations given his smoking history and comorbidities.

**Extended Data Fig. 5:**
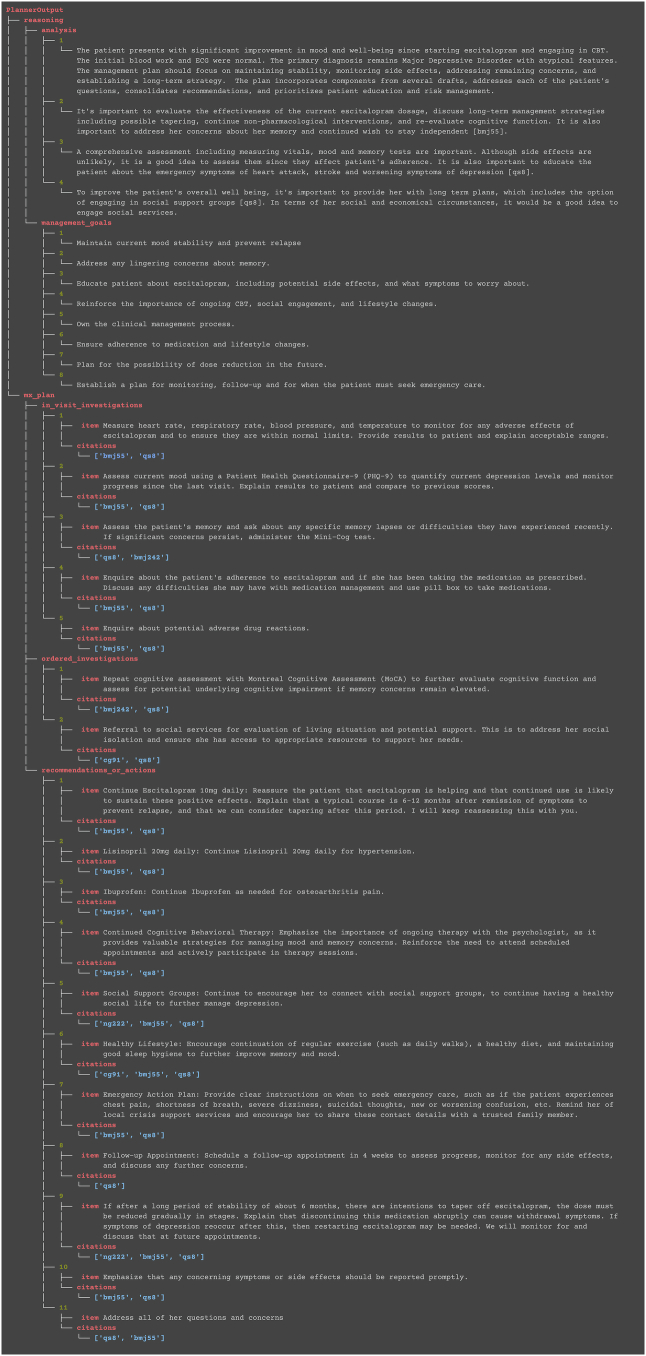
Mx Agent reasoning trace and plan. A fictive patient being followed up for a major depressive disorder. In this example, the Mx Agent develops an expectant plan for major depressive disorder that combines medication, lifestyle changes, and cognitive behavioral therapy, with appropriate monitoring and follow up, including a possible tapering plan. Note that interrogation of reasoning traces allows inspection of latent reasoning errors that are not propagated into the final (appropriate) Mx Plan. In this case, a reasoning trace inspection reveals that AMIE’s intermediate reasoning analysis suggests educating the patient about emergency symptoms of heart attack and stroke which are not clinically relevant in this context.

As described in Supplementary Information section 14, we ablated the effects of the posttrained model (Supplementary Information sections 5–8), the dialogue agent (Supplementary Information section 9) and the management reasoning agent (Mx agent) (Fig. 2 and Supplementary Information section 4). Further ablations of the Mx agent are provided in Extended Data Fig. 2, with details in Supplementary Information section 3. Example reasoning traces are shown in Extended Data Figs. 3–5, and an example management plan is presented in Supplementary Information section 2.

### Management Reasoning Empirical Key Features

**Fig. 5:**
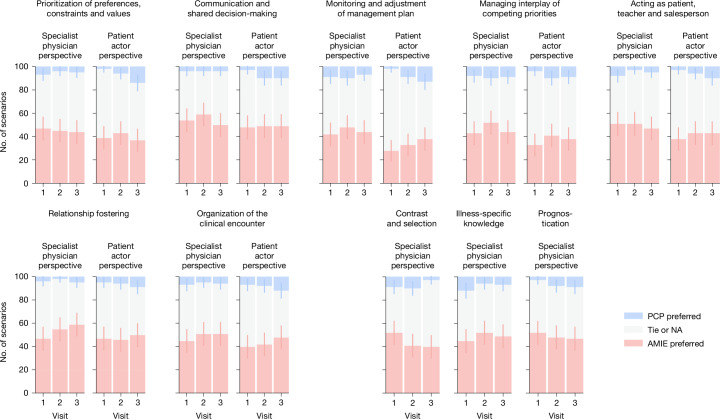
MXEKF performance. Relative performance of PCPs and AMIE on each of the ten MXEKF evaluation axes in terms of preferences expressed by specialist physicians and patient actors, respectively. Preferences were derived from independent ratings (on a five-point scale ranging from ‘Poor’ to ‘Excellent’) for each of three visits per scenario. Specialist physician preferences were based on median scores across three independent raters. For three of the ten MXEKF evaluation axes (contrast and selection, illness-specific knowledge, and prognostication), ratings were collected only from specialist physicians. Cases with identical ratings and those including at least one NA rating were grouped together as ‘Tie or NA’. Error bars represent 95% confidence intervals for binomial proportions.

The relative performance of PCPs and AMIE on each of the ten Management Reasoning Empirical Key Features (MXEKF) evaluation axes (Extended Data Table 1) was measured in terms of preferences expressed by specialist physicians and patient actors, respectively. Figure 5 provides a visualization of preference rates for a total of 51 unique combinations of MXEKF axis, scenario visit and rater perspective (3 visits × 10 MXEKF axes for specialist physicians + 3 visits × 7 MXEKF axes for patient actors).

#### AMIE preferred by patient actors and specialists

In roughly half of cases, no clear preference could be determined between AMIE and PCPs, resulting in a median tie rate of 49% (95% CI: 37%–62%; *n* = 51). However, when a preference was expressed, AMIE, with a median win rate of 47% (95% CI: 33%–58%; *n* = 51), was preferred significantly more often than PCPs, who had a median win rate of only 7% (95% CI: 2%–13%; *n* = 51). This trend was consistent across specialist physician and patient actor perspectives. Extended Data Figs. 7 and 8 show the full distributions of patient actor and specialist physician ratings, respectively.

#### MXEKF scores evolved across visits

For certain MXEKF axes, especially those pertaining to the temporal aspects of management reasoning, preference rates exhibited distinctive patterns across the three visits per scenario. For example, preferences for ‘Monitoring and adjustment of management plan’ and ‘Organization of the clinical encounter’ became more pronounced over the course of several visits, with tie rates shrinking and preference rates increasing for both PCPs and AMIE, as measured from the patient actor perspective. For ‘Relationship fostering’, preference rates for AMIE increased across visits, whereas PCP scores remained consistent, as measured from a specialist physician perspective.

### Medication reasoning accuracy

We present medication reasoning accuracy on the RxQA benchmark (Supplementary Information sections 15 and 16) separately for lower-difficulty (*n* = 282) and higher-difficulty (*n* = 318) questions in Extended Data Fig. 9. We compare a ‘closed-book’ setting, in which neither PCPs nor AMIE had access to external knowledge resources, with an ‘open-book’ setting, in which PCPs were given the relevant medication labels for each question, and AMIE was permitted to retrieve relevant information from the OpenFDA and British National Formulary (BNF) drug formularies. Supplementary Table 33 provides detailed results for all comparisons. In addition, Supplementary Table 34 provides results on this benchmark compared with other LLMs including DeepSeek-V3, o-3 and GPT-5.

#### RxQA is hard for both PCPs and AMIE

We observed that RxQA was a challenging test for both AMIE and PCPs, leaving ample room for future improvements. Although test accuracy differed significantly between closed-book and open-book settings as well as between difficulty levels, peak performance was below 75% for both PCPs (67.4%) and AMIE (73.8%) even in the lower-difficulty open-book setting.

#### AMIE wins on the more difficult subset

For the subset of questions rated by pharmacists as higher difficulty, AMIE was significantly more accurate than PCPs in both the closed-book setting (50.6% versus 41.5%, *P* = 0.013) and the open-book setting (57.9% versus 47.8%, *P* < 0.001). No significant difference was detected for questions of lower pharmacist-rated difficulty, for either the closed-book setting (52.8% versus 46.5%, *P* = 0.147) or the open-book setting (73.8% versus 67.4%, *P* = 0.071).

#### External knowledge helps PCPs and AMIE

Both PCPs and AMIE benefited significantly from the ability to access external knowledge resources. For lower-difficulty questions, both PCPs (46.6% versus 67.4%, *P* < 0.001) and AMIE (52.8% versus 73.8%, *P* < 0.001) significantly increased their accuracy (by more than 20%) when transitioning from the closed-book to the open-book setting. We observed the same effect with higher-difficulty questions, for which differences were less pronounced yet statistically significant for both PCPs (41.5% versus. 47.8%, *P* = 0.045) and AMIE (50.6% versus 57.9%, *P* = 0.010).

## Discussion

In this study, we showed that AMIE performed consistently well across a variety of management reasoning challenges, conducting clinical conversations that spanned multiple visits per patient and producing management plans that were non-inferior to those produced by board-certified PCPs.

The non-inferior performance of AMIE compared with PCPs was maintained throughout longitudinal clinical encounters. For the initial consultation, in particular, AMIE outperformed PCPs with respect to the overall appropriateness of the plan and provision of appropriate follow-up recommendations, although the performance gap with PCPs closed over the subsequent encounters. There were no domains in which PCPs outperformed AMIE. The use of AMIE in longitudinal care settings, including successful interpretation of further diagnostic information such as laboratory results and information from other medical consultants, was an important test of a potential real-world use case for conversational AI. Health systems worldwide are experiencing increased care fragmentation, which is associated with worsened morbidity for patients with chronic illnesses. In the real world, it is often more likely that a patient has seen different clinicians over sequential visits^11^. AI systems have not previously been specifically evaluated for this crucial unmet need. Our results indicate that AMIE and other conversational management agents could eventually provide continuity in otherwise fragmented health systems, either independently or working with clinicians. If such systems could be developed and tested with care and robust clinical evidence, they would have the potential to address growing unmet clinical needs caused by global shortages and inequalities of supply of PCPs, which are exacerbated by concerns of physician burnout and increasingly complex populations under management^12^.

There were important differences between the management plans produced by AMIE and those produced by human clinicians; for example, AMIE was considerably more precise in recommending both treatments and investigations, a trend that held over all three clinic visits. This improved preciseness, which involved giving specific and clear instructions for medications, follow-up plans, and contingencies, represented a change from generalized recommendations to actionable clinical directives. Previous versions of AMIE^1^ might have offered broad suggestions, such as ‘prescribe an antibiotic’ for a bacterial infection or ‘recommend a follow-up appointment’ for a patient requiring further monitoring. Although directionally correct, such recommendations have limited practicality in actual clinical care situations. By contrast, the enhanced variant of AMIE proposed in this work generates plans with a level of granularity that directly supports clinical implementation, such as naming the precise antibiotic, its dose, the duration of treatment and administration route, and any monitoring or follow-up actions that are required. In addition to clinical acceptability, preciseness has important implications for care engagement, especially given the increasing movement for patients to have access to their own medical notes. Precise, clear language is preferred by patients^13^ and might drive increased completion of follow-up visits and ordered diagnostics and tests^14^.

AMIE also selected the appropriate guidelines better than PCPs for the first visit, and its management decisions were better aligned with guidelines for all three visits. Management reasoning encompasses far more than guidelines, which often do not apply to individual practice settings, and experienced clinicians deviate from guidelines more often than novices^15,16^. However, implementation of clinical practice guidelines has been associated with higher quality of care^17^ and can reduce patient morbidity and mortality in cases of complex chronic illnesses^18^. AMIE’s guideline selection and alignment shows promise for increasing the feasibility of quality initiatives that might seek to implement guideline-directed medical management of chronic medical conditions such as congestive heart failure and diabetes mellitus, or to assist in the study of unwarranted variation from such recommendations. AMIE’s use of clinical guidelines also allows considerable flexibility and customization, as these guidelines can be easily revised: a clinic in a low- or medium-income country, for example, could easily switch from NICE guidance or BMJ Best Practice guidelines to World Health Organization guidelines more relevant to their care setting. Similarly, drug shortages have continued to be a global problem affecting health systems^19^ and can present challenges to correct implementation of practice guidelines; health systems using AMIE could just as easily revise their guidelines as they provide practice updates to clinicians. Although NICE guidance and BMJ Best Practice guidelines are primarily authored by UK-based institutions, they are applied or adapted in other parts of the world. We acknowledge that PCPs participating in this study were based in Canada and India; thus, their familiarity with these guidelines obtained through everyday practice may have been limited. However, all participating PCPs were given access to the corpus of guidelines throughout the entire study period and were free to peruse the guidelines without time limitation after the virtual consultations. We encourage future work to explore the use of localized guidelines in specific geographies and contexts of care.

In this study, we also developed the MXEKF rubric, which was derived from psychological research involving out-patient clinicians^7^. This pilot evaluation rubric addresses some of the main challenges in measurement of management reasoning quality: that decisions are subject to considerable amounts of context specificity, and that there is often no single answer to the best management plan. LLMs have generally fared less well on management tasks than diagnostic tasks compared with humans. However, on blinded ratings of MXEKF by specialist physicians, AMIE was preferred to PCPs significantly more often than PCPs were preferred, although they were tied in about half of the cases. These results require further exploration in follow-up studies but suggest that AMIE has potential to exhibit PCP-like quality in management reasoning.

The abilities of AMIE reflect the growing capabilities of LLMs for clinical conversation and reasoning. Its capabilities are intrinsically linked to advances across various domains, including reasoning^20–22^ and long-context processing^4,23,24^. For this reason, we suspect that current limitations (particularly confabulations, which represent considerable risks in clinical medicine) could be mitigated through steady improvement of the state-of-the-art LLMs underpinning the AMIE system. AMIE’s multiagent system and its ability to cite guidelines seem to have had a significant impact: confabulation rates were comparable between PCPs and AMIE and considerably lower than those reported in other studies^25,26^. The rapid progress of state-of-the-art LLMs shows promise for factual summarization. Whereas our backbone model, Gemini 1.5 Flash, exhibited a hallucination rate above 5% on the Hughes Hallucination Evaluation Model leaderboard^27^, this rate was reduced to 0.7% for the updated Gemini 2.0 Flash. Our ablation analysis (Supplementary Information section 14) further demonstrated this rapid evolution, with Gemini 2.5 Flash substantially improving conversational performance compared with the AMIE system built on Gemini 1.5 Flash. For AMIE, with the exception of a few criteria, the agent scaffolding substantially improved the agent’s history-taking (Supplementary Table 30). By contrast, when this scaffolding was provided to Gemini 2.5 Flash, there was little to no improvement in our auto-evaluated simulations. Although our Mx agent-specific ablation analysis (Supplementary Information section 2) quantitatively demonstrated the value of agentic guideline retrieval and reasoning leveraging long-context capabilities of base models, our auto-evaluated ablations, taken together, challenged the strict necessity of certain aspects of posttraining and multiagent setups as base models continue to improve. This research demonstrates the potential applications of conversational AI in disease management using agentic reasoning and long-context processing. The durable contributions of this research are the evaluation paradigm, rigorous ablation techniques, careful assessment of the safety-critical longitudinal capabilities of AI systems in a critical task for practising medicine, as well as the demonstration of the physician-level management reasoning capabilities of an AI system at a snapshot in time.

We also introduced a benchmark for medication reasoning, RxQA. The saturation of traditional medical reasoning benchmarks has added urgency to the need for development of new and more discriminant benchmarks^28,29^. It also demonstrates a new reality involving previously unseen capabilities of AI systems with human-like performance in domains that were once thought to be out of reach of machines. RxQA benchmarks knowledge of and reasoning about the use of medications. Derived from the national drug formularies of two countries, it is not intended to be fully comprehensive but rather to be revised over time using local data. RxQA was a challenging benchmark for both AMIE and human PCPs, with equivalent performance on the less difficult questions. However, AMIE outperformed human PCPs on the more difficult questions, both open- and closed-book. Notably, contemporary foundation models, including Gemini 2.5 Pro, o3 and GPT-5, achieved performance largely comparable with that of the AMIE system built on Gemini 1.5 Flash (Supplementary Table 34). This indicates that the relative performance gain provided by the AMIE system is modulated as the underlying foundation models become more capable. However, unlike base models, which were evaluated with medication labels provided directly in the context, AMIE uses dynamic retrieval to actively search for relevant information. This mimics a more realistic and challenging clinical workflow and remains a critical component of a deployable clinical system.

Despite these promising results, our study had several limitations. Although patient actors are a gold standard in medical education for the assessment of trainee clinicians, they are not representative of clinical care. Clinical scenarios were constructed, meaning that they had definitive answers, and were constrained for reliability in scoring. Owing to this, cases lacked much of the chart review to determine clinical history that characterizes real patient care. The case mix was also not indicative or representative of a real clinical practice setting. Our cases used guidelines from the UK, whereas our PCPs were from North America and India. Although PCPs were given relevant guidelines to review for each case as a method of mitigating these differences, this was not fully consistent with clinical practice, in which clinicians are unconstrained by guidelines and use their personal experience or point-of-care reference tools to aid management decisions.

In addition, although our cases unfolded over the course of weeks or months in the narrative specified in scenarios, the actual time between visits in our study was 1–2 days. This probably increased human performance on these cases, as it was not a realistic test of human memory. The interval history was also considerably constrained to the patient’s main complaint, in contrast to the open-ended paths of real patient care.

Although use of patient actors in an OSCE format mirrors standards for evaluation of physicians’ consultations, our results require further contextualization. Patient actors communicated with both AMIE and PCPs through a text-based chat interface. Despite the increased popularity of text-based patient portals for communication with PCPs^30^, telehealth visits are generally conducted using either audio or video chat and are by nature multimodal. Telehealth has grown in both popularity and importance since the COVID-19 pandemic^31^, spurring the development of telehealth OSCEs for health professions students^32^. The use of a text-only interface for AMIE was selected because of the convenience of this modality in communicating with a chatbot and the relative uniformity of the interaction compared with the complexities of intonation, body language and other cues in a multimodal audio/visual setting. It would also be significantly harder to blind both the patient actors and specialist raters to the source of the conversation in a multimodal setting.

The user interface configured for AMIE in this study has considerable differences compared with actual patient care. As mentioned above, telehealth is generally conducted by means of audio or video chat, not a text interface. Orders are not entered as free text but using an electronic provider order entry system. This is an important agentic task and constrains order entry in a manner that would be likely to improve the performance of AMIE, because such systems often contain order sets and only allow medication from acceptable drug formularies, as well as including prepopulated doses and clinical decision support. Practice alerts and oversight from pharmacists in health systems also improve accuracy; these were absent from our study.

Although we have attempted to develop and validate a tool for global measures of management reasoning that has construct validity, more work is necessary to demonstrate the reliability of MXEKF in the real world and to further describe its psychometric characteristics. This should be seen as a first step in the measurement of management reasoning. In this work, we assessed management reasoning on the basis of conversation content and post-questionnaire responses from both AMIE and PCPs. In addition, the AMIE system produced structured reasoning traces at several points throughout each conversation. Although we provide qualitative examples of these reasoning traces and highlight the possibility of latent errors within such traces, a comprehensive quantitative analysis of such traces was beyond the scope of this work. We encourage future work to study reasoning traces of medical AI systems in a comprehensive and quantitative manner and to test system architectures that further reduce errors in latent reasoning traces. Such approaches could involve agentic components that proactively critique the system’s reasoning traces and verify the correctness of guideline citations during inference time. Although such approaches would incur further computational or latency cost, they have the potential, if implemented in an efficient manner, to further mitigate latent reasoning errors and are therefore suitable candidates for future work.

We emphasize that this research is an art-of-the-possible demonstration of the capabilities and limitations of AMIE in longitudinal management reasoning using simulated settings with patient actors. Although the system shows promises, it is not ready for real-world translation and requires further research to mitigate issues such as latent reasoning errors, as well as prospective clinical studies to provide real-world evidence.

The RxQA benchmark also had several limitations. Selecting questions for which Gemini initially required medication labels to select the correct answer probably skewed the questions to be relatively harder and not necessarily representative of typical practice. Although each question was revised and validated by a board-certified pharmacist, there may have been significant interpharmacist variability. Furthermore, although we benchmarked against PCPs, medication decisions in the real world are often made with guidance and oversight from pharmacists. We do not suggest that these examples of human performance indicate any measure of real-world competence but rather intend them to serve as an experimental control group or baseline assessment to help contextualize comparisons of various AI systems in medication knowledge retrieval tasks.

## Conclusion

In this work, we demonstrated the ability of AMIE to both take histories and form differential diagnoses and to make nuanced management decisions that meet or exceed those of PCPs, including in cases that involve visits. Our findings do not suggest that AMIE is ready for clinical care. Our study was intended to explore possibilities, and many further steps, including prospective feasibility studies involving patients with appropriate ethical and safety oversight, will be necessary to ensure that AMIE can function as part of a healthcare team. Nonetheless, this study represents a milestone towards the goal of safe, equitable and ethical agentic health AI systems that can scale quality healthcare in increasingly fragmented health systems.

## Methods

### Conversational AI for disease management

In this work, we optimized AMIE for a setting in which it would converse with a patient by means of synchronous text chat in real time and over the course of multiple patient visit encounters. To reconcile the need for swift responses in synchronous conversation with the need for in-depth clinical reasoning, we used a multiagent system, inspired by the dual-process theory of cognition popularized by Kahneman in *Thinking, Fast and Slow*^33^ and also discussed in the context of diagnosis in ref. ^34^. AMIE with management reasoning capabilities is a system composed of two agents (Extended Data Fig. 1):

1. a dialogue agent, which engages in fast, intuitive and empathetic dialogue with the patient while maintaining a persistent conversational state across multiple visits;
2. an Mx agent, which plans patient care through more extensive inference-time computation. It continuously analyses the patient’s case, reasons about clinical guidance from a corpus of authoritative clinical knowledge (for example, clinical guidelines), and generates detailed and structured management plans.

In a dual-process theory analogy, the dialogue agent and Mx agent correspond to ‘system 1’ and ‘system 2’, respectively. Both agents are built on Gemini language models^35^ and use reasoning to improve response quality. The dialogue agent, which builds on work by Tu et al.^1^, is fine-tuned for multivisit medical conversations and diagnostic dialogue, whereas the Mx agent is optimized for complex reasoning and long-context understanding in multivisit patient contexts and hundreds of pages of full-text clinical guidelines. The Mx agent is invoked as a tool by the dialogue agent, whereas the dialogue agent references the latest management plan delivered by the Mx agent as an input to the conversation.

### Dialogue agent

The dialogue agent has two roles. First, it functions as a conversational interface, quickly generating appropriate responses to the user’s messages. Second, it orchestrates the rest of the system, internally managing the memory and state of the interaction, as well as pulling the latest plan from the specialized Mx agent.

#### Posttraining

We extended the simulated learning framework described by Tu et al.^1^ to optimize the LLM powering the dialogue agent for a multiturn, multivisit conversational setting and scale learning across a wide range of medical contexts and specialties (Supplementary Information sections 5 and 6). As in the work of Tu et al.^1^, we relied heavily on simulated doctor–patient dialogues and other clinically relevant datasets to train our model; however, we made a few notable changes to improve its performance in this setting.

1. Base model: we built on top of Gemini 1.5 Flash instead of the previously used PaLM-2 (ref. ^36^) (refer to the Gemini technical report for more details on the base LLM architecture^4^). We chose to use Gemini 1.5 Flash for this study primarily owing to its long-context capabilities, which are necessary for inference-time reasoning across a large corpus of medical knowledge. This is a key capability studied in this work and would not have been possible with PaLM-2.
2. Multivisit simulated dialogues: we developed a new set of simulated dialogues (Supplementary Information section 5.2) to improve multivisit reasoning. Prompts for dialogue generation and auto-evaluation are listed in Supplementary Information sections 7 and 8, respectively. The final datasets used to fine-tune the base model for this study are a superset of those described by Tu et al.^1^ and include various medical question-answering tasks, electronic health record summarization tasks, real-world medical dialogues^37^ and the simulated dialogue datasets.
3. Reinforcement learning with human/AI feedback: following supervised fine-tuning (Supplementary Information section 6.1), we performed reinforcement learning with human/AI feedback^38^, curating medically relevant tasks to further enhance the conversational and disease-management capabilities of the model (details are provided in Supplementary Information section 6.1). Here the reward model is trained from both human- and LLM-generated pairwise preferences for dialogue responses, case management recommendations^39,40^ and more (Supplementary Table 8).

#### Chain of reasoning

During inference, the dialogue agent uses a sequence of model calls, or a ‘chain of reasoning’ to generate its ultimate response to the patient (see Supplementary Information section 9.1 for specific prompting details). These model calls are low latency; the response of the dialogue agent can be output a few seconds after a new patient message is received. Unlike the previous version of the AMIE system^1^, each chain-of-reasoning step in the dialogue agent is dependent not only on the full dialogue history up to that point but also on an internally managed state that is maintained across multiple patient visit encounters.

The chain-of-reasoning steps for this agent are as follows.

1. Plan response: the dialogue agent reasons about what it should do next, including details to enquire about, patient questions to respond to, and deciding whether to wrap up or continue the conversation.
2. Generate response: with its output from the previous step in context, the dialogue agent drafts a response to the patient’s last message.
3. Refine response: before sending out the drafted response, the dialogue agent revises it against a set of criteria to ensure the response meets quality standards.

#### Agent state

The agent state is a modular data structure that represents the agent’s beliefs about the current and past conversations. This state is accessible to both the dialogue agent and the Mx agent. State fields are revised periodically throughout the dialogue by an asynchronous background reasoning subroutine. The state decomposes into the following fields.

1. Current patient summary, which condenses all confirmed positive and/or negative symptoms, demographics, medical, drug, family and social history, and more into a concise summary.
2. Current differential diagnosis: this lists what AMIE believes to be the most probable diagnosis and other plausible diagnoses that should be considered at a given point in time.
3. Current management plan, including investigations to be done during the visit, investigations to be ordered after the visit and recommended actions following the visit. Rather than being generated by the dialogue agent itself, this task is outsourced to the Mx agent, which more thoroughly and carefully reasons over clinical guidelines to formulate this plan.

The specific prompts used to update the agent state are described in Supplementary Information section 9.2. Further attributes of the agent state, such as ‘visit number’, are also used to enable more context-specific logic, for instance, unique prompts for initial versus follow-up visits.

### Mx agent

The goal of the Mx agent is to plan the patient’s care. Building on Gemini’s state-of-the-art long-context capabilities^4^, this agent synthesizes and reasons over large amounts of information, comprising patient context across several visit encounters and hundreds of pages of clinical guidelines, to craft a management plan tailored to the patient’s specific context. The resulting management plan is a structured set of investigations and interventions. Individual recommendations in the management plan are annotated with citations to the reference documents, such as clinical guidelines, to provide interpretability and traceability. An example is provided in Supplementary Information section 2.

#### Design optimization

The Mx agent was designed to use the maximum computational power permissible under real-time user interaction constraints, on the basis of the expectation that management reasoning capabilities would improve with increasing test-time computation^23,41–45^. For this work, we targeted a response time of no more than 1 min to avoid dissociation between the two agents. We used auto-evaluation to guide our search over potential parameters (for example, context length, prompts, inference strategy, structural constraints) for the optimal agent design. Details of this optimization process are described in Supplementary Information section 3.

In the final design, the Mx agent operates in overlapping stages (Extended Data Fig. 1). First, it retrieves clinical guideline documents on the basis of generated queries and abstract embeddings. Second, it produces four draft management plans. Third, it refines and merges the drafts into a final management plan using a final generation step conditioned on retrieved documents. Plan drafting and refinement are analogous to ‘ensemble refinement’^46^ and allow diverse management strategies to be explored concurrently. Each plan is generated by chaining long reasoning and plan construction within a single model call; this is enabled by structural constraints.

#### Long-context reasoning

We designed the Mx agent to tap into Gemini’s long-context reasoning capabilities^4,23,24^ rather than investing in engineering complex retrieval pipelines^47^, which come with inherent performance limitations^24,48^ (for example, compounding errors), technical dilemmas (for example, choice of chunking algorithm) and often higher latency (that is, extra communication costs). In addition to the shared agent state (which holds patient-specific information), we provide various clinical guideline documents as input to the model; these are minimally converted from their original source format to Markdown while retaining key elements of the document structure such as section headings, lists and tables, to allows preservation of global semantic coherence within a single document. Clinical guideline documents can be extensive, with a typical range of 30 to 50 pages. Our approach enables the model to interact with in-context data at every step of the generation process for rich cross-document reasoning.

In the context of management reasoning, multidocument reasoning is often crucial for addressing complex presentations. For instance, determining the optimal treatment for a patient with comorbidities may necessitate integrating information from distinct guidelines, at a minimum of one for each condition. Nevertheless, tracing management recommendations back to reference documents remains an open problem, especially when using a large number of in-context sources. Rather than involving specialized tools for post hoc citation attribution, we generate citations as an integral part of the reasoning process. Each citation task can be viewed as an instance of in-context retrieval^24^, at which long-context models like Gemini already perform on par with specialized methods^24^.

Long-context capabilities allow reasoning models to interact with large amounts of data through repeated in-context interactions. This reasoning process can be seen as emulating multistep agentic workflows. In a single call, the model can look up key information in source documents, summarize and reference multiple passages, and search the documents again following preliminary conclusions. Long-context models are competitive with specialist systems on corpus-in-context tasks such as open-domain question answering^24^. To make our system more robust, we used decoding constraints to enforce target behaviours, including citing sources and summarizing high-level management strategy on the basis of in-context documents (more details are provided in the ‘Structured generation’ subsection).

#### Coarse retrieval

The corpus of clinical guidelines used in this study accounts for a total of 10.5 million tokens (across 627 documents), which exceeds Gemini’s 2 million context window. Therefore, a preliminary retrieval step was required to coarsely filter out irrelevant documents. We built a simple retriever system using Gecko 1B text embeddings^49^, which we use to index all guidelines on the basis of titles and abstracts. The abstracts are generated ahead of time, emphasizing clinically relevant features such as target demographics, clinical goals and relevant conditions, symptoms, tests and interventions. Upon receiving a new patient case, the Mx agent generates up to five search queries in natural language. We used auto-evaluation (Supplementary Information section 3) to determine the optimal number of context tokens (256,000) to allocate for external knowledge resources. On average, this corresponds to six clinical guideline documents, which can all be synthesized and reasoned over in context simultaneously. The prompts for query and abstract generation are provided in Supplementary Information section 4.

#### Structured generation

Prompting alone does not offer guarantees with respect to the structure used for internal reasoning steps or the generated output. This can be problematic in cases in which a certain structure is expected, either for reasons of interpretability and traceability (for example, a requirement for explicit citations) or to serve an interface between two components (for example, between the dialogue agent and Mx agent). To enable controllable multistep reasoning with structural guarantees, we used decoding constraints^50^ to guide the model output using a predefined JSON schema^51^. Chaining a predefined number of reasoning steps and final plan construction together within a single model call guarantees a valid plan structure and also ensures that predicted leaf values, such as citations, match a predefined format or range of values. Chaining multiple steps in a single model call also reduces latency, as it requires encoding the documents only once.

Figure 2a provides a visualization of the reasoning structure imposed by the Mx agent. The structure is defined in Python code and automatically converted along with docstrings to JSON-equivalent decoding constraints. Auto-evaluation (Supplementary Information section 3) was used to determine the reasoning structure for this task. We illustrate a reasoning trace generated using this structure in Fig. 2b. The reasoning trace consists of three components:

1. analyse patient (evaluate symptoms, medical history and overall clinical picture) (Fig. 2c);
2. set objectives (define high-level management goals) (Fig. 2d);
3. plan and cite (plan management steps and cite appropriate clinical guidelines) (Fig. 2e).

The model prompt for this approach is available in Supplementary Information section 4. Several examples of reasoning traces and management plans are presented in Supplementary Tables 21–23, and further modelling details are provided in Supplementary Information section 3.

### Evaluation

In this work, we sought to evaluate AMIE’s capabilities in conversational AI for disease management, extending our evaluation beyond diagnostic accuracy to encompass longitudinal reasoning and guideline-driven clinical decision-making. To this end, we adapted and extended the remote virtual OSCE paradigm introduced in ref. ^1^. To assess medication reasoning specifically, we developed RxQA, a multiple-choice question benchmark derived from drug formularies and validated by board-certified pharmacists.

### Multivisit OSCE study

We used a randomized, blinded design comparing AMIE with PCPs with respect to their performance of multivisit consultations with trained patient actors through a synchronous text-chat interface (Fig. 3). This approach allowed us to simulate the longitudinal nature of disease management and assess ability to adapt plans and recommendations on the basis of evolving patient information that was revealed in an incremental manner. Patient actors completed each multivisit scenario twice, once with AMIE and once with a PCP, in a blinded and randomized order. Each scenario completion involved three text-chat conversations corresponding to visits 1, 2 and 3, with an interval of around 2 days (minimum 24 h) between visits. The exact interval depended on the availability of patient actors and PCPs during a given week. For logistical reasons, the between-visit intervals enforced in the OSCE study were static and did not necessarily reflect the duration of time between visits specified by the narrative of the case scenario (for example, follow-up after 2 weeks). Patient actors, PCPs and AMIE were instructed to act on the basis of the time intervals described in the scenarios. To simulate the patient–physician relationship and assess longitudinal reasoning, the same patient actor was used for all three visits in a given scenario and for both scenario completions (one with AMIE and one with a PCP) to ensure consistency in how scenarios were enacted. For PCP interactions, the same PCP was paired with the same patient actor for all three scenario visits. PCPs were allowed to take notes and review them between visits. In addition, the interface displayed full conversation transcripts from all previous visits during each session to allow PCPs to review details from previous conversations about the case as needed.

#### Scenarios

Scenario packs were prepared by clinical providers in Canada and India and designed to describe the evolution of a patient’s condition over three distinct visits. Each scenario specified the chief concern, symptoms and medical history at the initial visit, as well as changes in these factors, treatment responses, test results and imaging findings available at the beginning of subsequent follow-up visits. In total, 100 scenarios were used. The scenarios were equally distributed between providers in India and Canada and across five medical specialties (20 scenarios each): cardiology; pulmonology; obstetrics, gynaecology and urology; gastroenterology; and neurology and musculoskeletal. This balanced design ensured a comprehensive evaluation across diverse medical domains and clinical contexts. To assess ability to handle the complexities of real-world practice, we designed scenarios with varying difficulty levels, many of which included information inconsistency (for example, a patient not sharing all information truthfully) and/or multimorbidity (for example, several copresenting diseases requiring treatment trade-offs). We provide an overview of all 100 evaluation scenarios and 20 validation scenarios in Supplementary Information section 13, including metadata such as medical specialty, provider location, underlying condition, relevant clinical guidelines and types of added difficulty. For illustrative purposes, we also provide a detailed view of two sample scenarios, including all information given to the patient actor, as well as multiturn conversations, post-questionnaire responses, and gradings from specialist physicians and patient actors for all three visits and for AMIE and PCPs, respectively (Supplementary Data 1). For all 120 scenarios, we provide scenario details and AMIE output in Supplementary Data 2 and 3 in PDF and CSV format, respectively.

#### Clinical guidelines

Incorporation of clinical guidelines was a key aspect of the study design. Each scenario was designed to have a specific ground truth diagnosis, and was grounded in a pair of clinical practice guidelines, one from the UK NICE Guidance corpus (https://www.nice.org.uk/guidance) and one from the BMJ Best Practice corpus (https://bestpractice.bmj.com). The NICE guidance is a corpus of evidence-based recommendations for the healthcare and social care sector, developed by independent committees including professionals and lay members and consulted on by stakeholders. BMJ Best Practice is a clinical decision support tool that supports healthcare professionals to make evidence-based decisions about diagnosis, treatment and prevention. For cases in which evidence is scarce or equivocal, expert opinion is provided in BMJ Best Practice. For each visit, the scenario specified a reference treatment plan consistent with these guidelines, outlining appropriate investigations and tests to perform during the visit, investigations to order after the visit, and action and treatment recommendations. This approach ensured that the consultations were grounded in established evidence-based practices and allowed us to test the consistency of the systems involved with respect to clinical guidelines.

The guideline corpus used in this study was a limited sample of the content covered by the NICE guidance and BMJ Best Practice collections. To simulate information resources and the lookup tasks faced by clinicians, both PCPs and AMIE had access to a corpus of 627 clinical guidelines, comprising 527 NICE guidance and 100 BMJ Best Practice documents. These included 50 NICE guidance and 50 BMJ Best Practice guidelines that were directly relevant to the scenarios, as well as a sample of less relevant guidelines. Both PCPs and AMIE were instructed to reference this corpus during the study and ground their clinical decision-making in the guidelines as appropriate. We report statistics about the corpus of guidelines in Supplementary Information section 12. Guideline content was used by AMIE at test time.

#### Participants

The study involved 21 board-certified PCPs and 21 validated patient actors, equally distributed across India and Canada. PCPs had a median of 9 years of postresidency experience (interquartile range: 5–12 years). To assess the quality of AMIE and PCP consultations and responses to post-questionnaires, we recruited 30 specialist physicians from India and North America, ensuring a range of clinical perspectives, with 3 specialist physicians for each of the combinations of medical specialty and geographic location (5 medical specialties, 2 geographic locations and 3 specialist physicians each). These specialists were matched to evaluation tasks on the basis of medical specialty and geographic location (for example, cardiologists in India evaluated cardiology scenarios enacted by patient actors and PCPs in India). Specialist physicians had a median of 7 years of postresidency experience (interquartile range: 4–12 years). Further metadata of PCP participants and specialist physician raters are provided in Supplementary Information section 11.

#### Evaluation measures

For each scenario visit, data were collected from various sources, including patient actors, AMIE or PCPs, and specialist physicians. In addition to the consultation transcripts, data included responses to post-questionnaires from AMIE and PCPs. Post-questionnaires were completed by both AMIE (by offline generation) and PCPs (manually) after each visit. Post-questionnaires asked for a differential diagnosis list (at least one and up to ten items), the set of clinical guidelines deemed most applicable (at least one and up to three from NICE guidance and BMJ Best Practice each) and a management plan consisting of a list of investigations performed during the visit, as well as investigations, treatments and follow-up actions recommended for after the visit. To enable assessment of MXEKF rubrics from a specialist physician perspective, the post-questionnaire also asked about aspects of management reasoning including whether and how AMIE and PCPs intentionally deviated from one of the selected clinical guidelines, as well as comments about other acceptable management plans, competing priorities, the cost, effectiveness and side effects of the proposed plan, the prognosis of the patient with and without treatment, and recommendations for escalation and follow-up. The evaluation framework considered both clinician-centred and patient-centred perspectives to provide a holistic assessment, with raters blinded to the source of the data (AMIE or PCP) in both cases.

At the patient actor level, we assessed conversation quality and patient experience using rubrics from ref. ^1^, including the General Medical Council Patient Questionnaire, which evaluates aspects such as being polite and making the patient feel at ease; and the Practical Assessment of Clinical Examination Skills (PACES) rubric, which assesses skills in eliciting information and managing patient concerns. However, we replaced the Patient-Centered Communication Best Practice rubric from ref. ^1^ with a new pilot evaluation rubric, which we refer to as MXEKF. The MXEKF rubric (Extended Data Table 1) was designed to specifically capture key aspects of management reasoning. MXEKF was inspired by previous work^52^ that identified 12 empirically determined key features. Of these 12 features, we retained 10 for MXEKF: (1) contrast and selection among several reasonable and defensible solutions; (2) prioritization of preferences, constraints and values; (3) communication and shared decision-making; (4) continuing monitoring and adjustment of the management plan; (5) dynamic interplay among people, systems, settings and competing priorities; (6) illness-specific knowledge; (7) clinician roles as patient, teacher and salesperson; (8) clinician–patient relationship; (9) prognostication; and (10) organization of the clinical encounter. We chose not to include the other two features identified in ref. ^52^: process knowledge (assessing understanding of systems of care), because it would be specific to particular clinical environments; and management scripts (understanding of conceptual knowledge structures and clinician tasks), because it would have been infeasible to collect management scripts from PCPs as part of the OSCE study. The MXEKF rating questions are detailed in Extended Data Table 1. Patient actors completed the full General Medical Council Patient Questionnaire rubric and subsets of the PACES and MXEKF rubrics after each of the three scenario visits.

**Extended Data Fig. 6:**
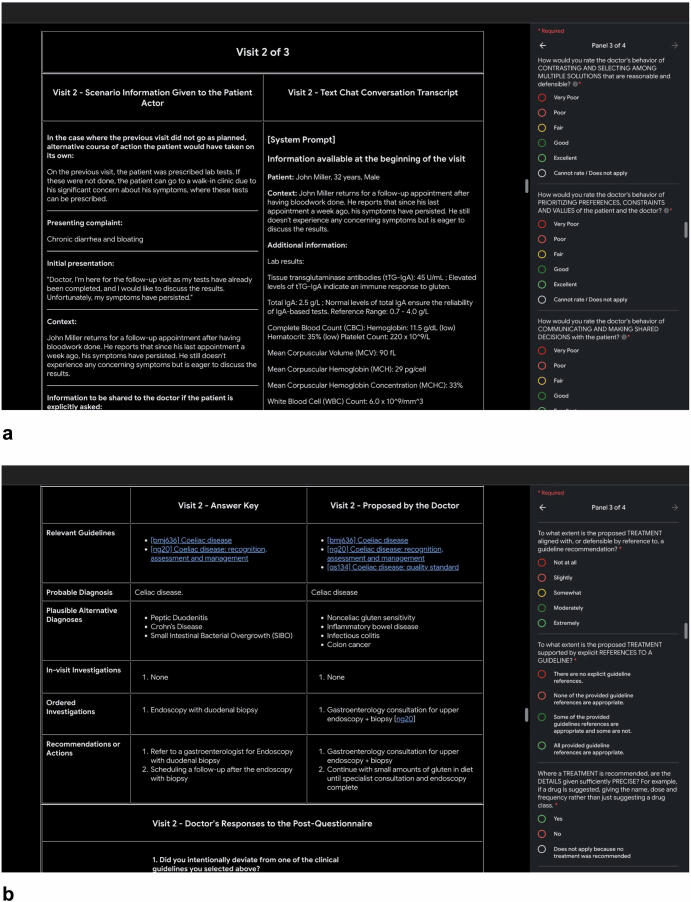
UI for specialist evaluators. a) Specialist physician reviews the conversation transcript for visit 2 of 3 and assesses the doctor’s behavior). b) Specialist physician assesses the proposed management plan and selected guidelines for visit 2 of 3.

**Extended Data Fig. 7:**
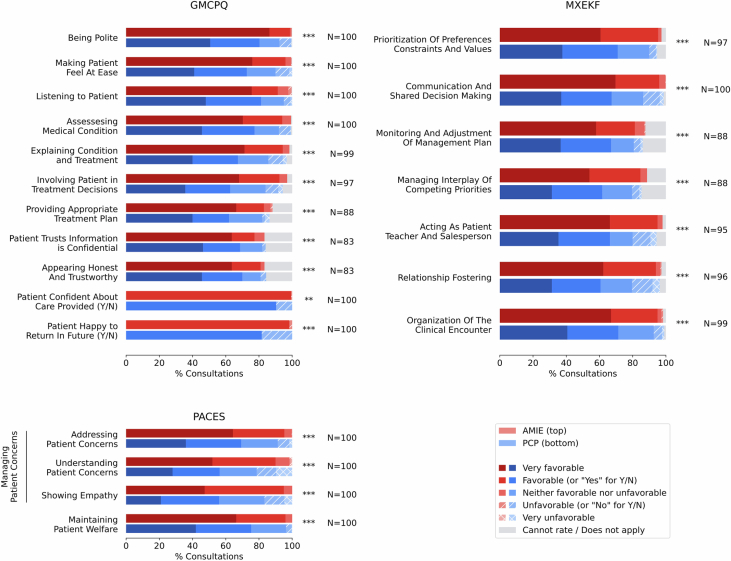
Patient actor ratings. Conversation qualities as assessed by patient actors upon conclusion of the consultation. For illustration purposes, all responses from five-point rating scales were mapped to a generic five-point scale ranging from ‘Very favorable’ to ‘Very unfavorable’. For Yes/No questions, a (positive) ‘Yes’ response was mapped to the same color as ‘Favorable’ and a (negative) ‘No’ response to the same color as ‘Unfavorable’. Rating scales were adapted from the General Medical Council Patient Questionnaire (GMCPQ), the Practical Assessment of Clinical Examination Skills (PACES), and a set of Management Reasoning Empirical Key Features (MXEKF). Details on question wording and response options are provided in Extended Data Table 1. Asterisks represent statistical significance (*: *p* < 0.05, **: *p* < 0.01, ***: *p* < 0.001, *n*. *s*. : not significant).

**Extended Data Fig. 8:**
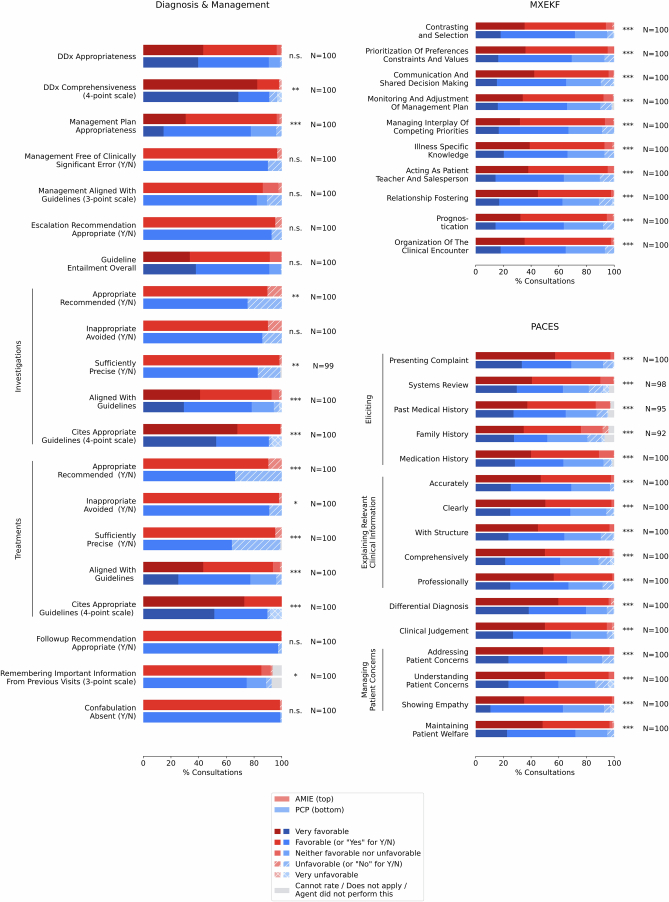
Specialist physician ratings. Conversation and reasoning qualities as assessed by specialist physicians. For illustration purposes, all responses from five-point rating scales were mapped to a generic five-point scale ranging from ‘Very favorable’ to ‘Very unfavorable’. The only four-point scale (DDx Comprehensiveness) was mapped to the same scale, ignoring the ‘Neither favorable nor unfavorable’ option. For Yes/No questions, a (positive) ‘Yes’ response was mapped to the same color as ‘Favorable’ and a (negative) ‘No’ response to the same color as ‘Unfavorable’. Rating scales were adapted from the Practical Assessment of Clinical Examination Skills (PACES), a set of Management Reasoning Empirical Key Features (MXEKF), and other sources (see Extended Data Table 1). For statistical testing, ratings from three distinct specialist physician raters and three visits for each case were aggregated using the median. Asterisks represent statistical significance (*: *p* < 0.05, **: *p* < 0.01, ***: *p* < 0.001, *n*. *s*. : not significant).

**Extended Data Fig. 9:**
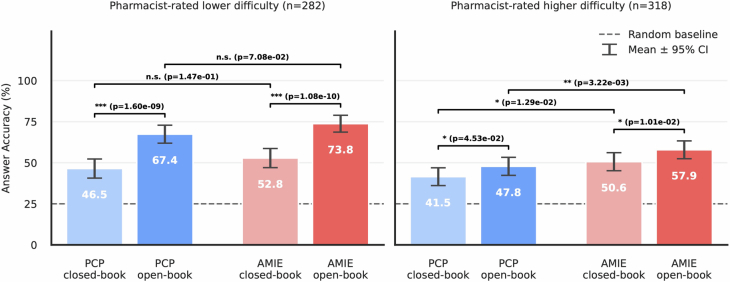
RxQA Medication Reasoning Accuracy. Question-answering accuracy of PCPs and AMIE on the RxQA medication reasoning benchmark. Results are presented separately for lower-difficulty (left) and higher-difficulty (right) questions as rated by a set of board-certified pharmacists. Here, we consider questions which pharmacists rated as “Trivial” or “Easy” to be lower-difficulty questions. We compare a “closed-book” setting in which neither PCPs nor AMIE have access to external knowledge resources, to an “open-book” setting where PCPs are given the relevant medication label for each question, and AMIE is permitted to retrieve relevant information from drug formularies. Data are presented as mean accuracies with error bars representing 95% confidence intervals for binomial proportions. Statistical significance was determined using two-sided McNemar’s tests with false discovery rate (FDR) correction (*, *p* < 0.05; **, *p* < 0.01; ***, *p* < 0.001; n.s., not significant). Exact *P* values for all comparisons are provided in Table SI.33. *n* = 282 (lower-difficulty) and *n* = 318 (higher-difficulty) independent questions examined over a single independent evaluation.

At the specialist physician level, evaluations focused on consultation quality, guideline adherence, and the appropriateness of diagnostic and management decisions. Specialist physicians assessed AMIE and PCPs on the basis of their consultation transcripts and responses to the post-questionnaires using various rubrics. In addition to the full MXEKF and PACES rubrics, these included an expanded version of the ‘Diagnosis and management’ rubric from^1^. The extra rating questions in this expanded rubric are detailed in Extended Data Table 1. In addition to accuracy and completeness of the differential diagnosis, and appropriateness of escalation, investigations and treatments, the expanded rubric incorporated detailed evaluation axes related to guideline entailment and implementation, the preciseness of recommended investigations and treatment, and ability to remember relevant information from previous visits. Specialist physicians rated all rubric items for each visit separately (and considering past visits). During the evaluation task, information for all three visits per scenario was presented to specialist physicians at once to enable a holistic assessment based on full information. Each rating task was completed by a set of three independent specialist physicians. For robustness, results are reported using the median rating score (or majority vote for binary ratings) among the three independent raters. We also provide evidence of interrater reliability for our framework in Supplementary Information section 10. The evaluation interface for specialist physicians is shown in Extended Data Fig. 6. Before involvement in the final evaluation study, specialist physician raters completed a set of pilot rating tasks (based on the 20 scenarios in the validation set) to familiarize themselves with the task setup and instructions. Detailed rating instructions were embedded in the rating interface itself, along with tooltips contextualizing specific rating prompts. Although we leveraged one specific model and agent configuration in the final study, we conducted an ablation analysis of various configurations using an entirely AI-simulated end-to-end recreation of our OSCE study and corresponding auto-evaluations. The methods and results for this ablation analysis are detailed in Supplementary Information section 14.

### RxQA medication reasoning benchmark

To assess AMIE’s medication reasoning further, we developed a multiple-choice question benchmark, RxQA, which consisted of 600 questions derived from national drug formularies across two jurisdictions: OpenFDA (https://open.fda.gov/) and the BNF (https://bnf.nice.org.uk/). We used Gemini 1.5 Flash^4^, with access to these medication labels, to draft and filter the questions, which were further refined by board-certified pharmacists in each jurisdiction (Supplementary Information section 16). The dataset was built as follows.

1. Medication labels: we ingested the OpenFDA and BNF drug formularies, formatting the medication labels in a clean format and sampling a subset of labels from each source.
2. Question generation: from each label, we generated five short questions, each with four answer choices, using the prompt shown in Supplementary Information section 16.1, and three longer questions using the process shown in Supplementary Information section 16.2.
3. Question filtering: we validated question quality by asking Gemini to confirm that each question was adequately specified and that the listed answer was unambiguously correct among the four choices.
4. Question selection: we randomly selected 100 ‘short questions’ and 200 ‘long questions’ from each jurisdiction for which Gemini required the corresponding medication context to answer correctly.
5. Pharmacist revision: we recruited four board-certified pharmacists in each jurisdiction to revise the question wording, answer options and correct answer. The pharmacists rated the difficulty of each question (trivial, easy, medium, hard or impossible). For subgroup analyses, trivial and easy were mapped to lower difficulty, whereas medium, hard and impossible were mapped to higher difficulty.

An example question, derived from OpenFDA, is shown in Supplementary Fig. 2. We assessed the performance of AMIE on RxQA by ingesting the OpenFDA and BNF drug formularies with the Mx agent and prompting the system with the question in a zero-shot manner. As a baseline of human test-taking performance, we also asked a group of three PCPs in each jurisdiction to answer each question, first without the reference medication context and then with access to this context. The OpenFDA portion of the RxQA dataset is available at https://github.com/Google-Health/rxqa.

### Statistical analysis

The quality of management plans for each of three visits was measured as the proportion of cases with favourable ratings from specialist physicians. Of 15 evaluation axes tested, 9 were based on yes/no rating scales. The remaining ones were binarized using the top two options on the respective scales: ‘overall appropriate’ (five-point scale), ‘selected applicable guidelines’ (five-point scale), ‘aligned with guidelines’ (five-point scale), ‘references guidelines’ (four-point scale). For each evaluation axis, cases with ratings of ‘NA’ on either study arm were excluded for each visit. The McNemar test was used for each evaluation axis and visit, and *P* values were adjusted using false discovery rate correction.

The relative performance of PCPs and AMIE on each of the ten MXEKF evaluation axes was measured in terms of preferences expressed by specialist physicians and patient actors, respectively. Preferences were derived from independent ratings (on a five-point scale ranging from ‘poor’ to ‘excellent’) for each of the ten axes and each of three visits per scenario. For three of the ten MXEKF evaluation axes (contrast and selection, illness-specific knowledge, and prognostication), ratings were collected from specialist physicians but not patient actors. In total, we computed preference rates for 51 unique combinations of MXEKF axis, scenario visit and rater perspective (three visits with ten MXEKF axes for specialist physicians; and three visits with seven MXEKF axes for patient actors). Summary metrics for preference rates refer to the spread of preferences across these 51 sets of preference rates.

For comparisons of RxQA medication reasoning accuracy, the McNemar test was used with question-level pairing, and *P* values were adjusted using false discovery rate correction.

### Reporting summary

Further information on research design is available in the Nature Portfolio Reporting Summary linked to this article.

## Online content

Any methods, additional references, Nature Portfolio reporting summaries, source data, extended data, supplementary information, acknowledgements, peer review information; details of author contributions and competing interests; and statements of data and code availability are available at 10.1038/s41586-026-10764-5.

## Supplementary information

Supplementary Information Supplementary discussion, methods and results (sections 1–16). Contains related work, details of the system design for the Mx agent and dialogue agent, details of the OSCE evaluation study (interrater reliability analysis, clinician metadata, scenario metadata, ablation analysis), and method details and further results for the RxQA medication reasoning benchmark.

Reporting Summary

Supplementary Data 1 Detailed view of two sample scenarios with AMIE and PCP output and evaluation gradings. Full details for two sample scenarios used in the OSCE evaluation study, including scenario information, AMIE–patient-actor conversations, PCP–patient-actor conversations, specialist physician gradings and patient actor gradings for all three visits per scenario.

Supplementary Data 2 Details for all 120 OSCE scenarios with AMIE output (PDF). Scenario details and AMIE output for all 120 scenarios used either in the OSCE evaluation study (100) or for validation purposes (20), in human-readable PDF format.

Supplementary Data 3 Details for all 120 OSCE scenarios with AMIE output (CSV). Scenario details and AMIE output for all 120 scenarios used either in the OSCE evaluation study (100) or for validation purposes (20), in machine-readable CSV format.

Peer Review File

## Figures and Tables

**Extended Data Table 1:**
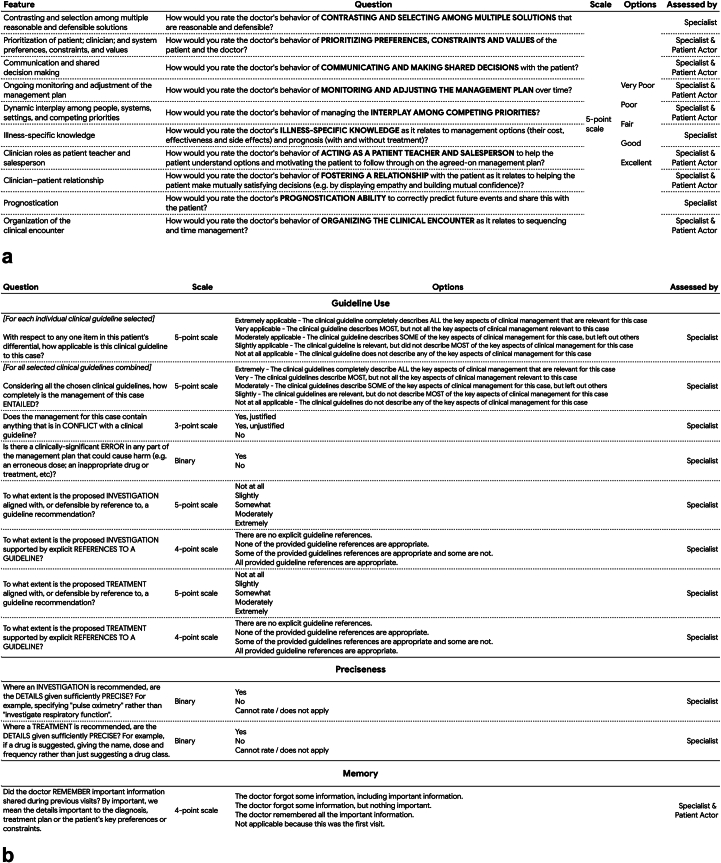
Evaluation Rubrics

a) Management Reasoning Empirical Key Features (MXEKF) rubric details. b) Guideline Use, Preciseness and Memory rubric details. Adapted with permission from ref. ^52^, Oxford University Press.

## Data Availability

Some of the datasets used in the development of AMIE were open-source (MedQA, MultiMedQA, MIMIC-III). Details of the 120 OSCE scenarios used in this work (20 for validation and 100 for evaluation) are available as part of this publication, including AMIE output for all 120 scenarios. For two sample scenarios, we also share PCP output and ratings from patient actors and specialist physicians. These OSCE study details are provided as part of the Supplementary Information. The OpenFDA portion of the RxQA dataset is available at GitHub (https://github.com/Google-Health/rxqa). Owing to licensing restrictions, the BNF portion cannot be shared directly by Google. However, we have shared this portion with BNF itself, and the interested reader is encouraged to contact BNF to request access to the data.

## Code availability

AMIE is an LLM-based research AI system for conversational disease management. We have not open-sourced the model code or weights owing to the safety implications of unmonitored use of such a system in medical settings. In the interest of responsible innovation, we will work with research partners, regulators and providers to validate and explore safe onward uses of AMIE. For reproducibility, we have documented technical deep learning methods while keeping the paper accessible to a clinical and general scientific audience. Our work builds on Gemini 1.5, for which technical details have been described extensively in the technical report^4^.
