# Supplementary material for: Towards conversational artificial intelligence for disease management
Source: Nature. 2026 Jun 17;655(8125):1292–9. doi: 10.1038/s41586-026-10764-5 (PMC13421318; doi:10.1038/s41586-026-10764-5)
Supplement: Supplementary file 1 — Supplementary discussion, methods and results (sections 1–16). Contains related work, details of the system design for the Mx agent and dialogue agent, details of the OSCE evaluation study (interrater reliability analysis, clinician metadata, scenario metadata, ablation analysis), and method details and further results for the RxQA medication reasoning benchmark. [file 41586_2026_10764_MOESM1_ESM.pdf]

nature portfolio

https://doi.org/10.1038/s41586-026-10764-5

Supplementary information

Towards conversational artificial intelligence for disease management

In the format provided by the authors and unedited

Supplementary Information

Supplementary Discussion:

- Section SI.1 Related Work

Mx Agent:

- Section SI.2 illustrates an example management plan for a patient with undiagnosed pheochromocytoma.
- Section SI.3 describes the design optimization of the Mx Agent, presenting auto-evaluation of various strategies on a set of 20 validation scenarios.
- Section SI.4 lists prompts used by the Mx Agent at inference time.

Dialogue Agent:

- Section SI.5 details the generation and auto-evaluation of single and multi-visit simulated dialogues for fine-tuning the Dialogue Agent's underlying language model.
- Section SI.6 details the post-training procedure for the Dialogue Agent's underlying language model, including supervised fine-tuning (Section SI.6.1) and reinforcement learning from human/AI feedback (Section SI.6.2).
- Section SI.7 lists prompts used to generate the single-visit and simulated multi-visit doctor-patient dialogues used to fine-tune the Dialogue Agent's underlying language model.
- Section SI.8 lists prompts used to auto-evaluate and filter dialogues for fine-tuning the Dialogue Agent's underlying language model.
- Section SI.9 lists prompts used by the Dialogue Agent at inference time, including prompts for its chain-of-reasoning (Section SI.9.1) and prompts for updating the agent state (Section SI.9.2).

OSCE Study:

- Section SI.10 provides an inter-rater reliability analysis for specialist physician ratings.
- Section SI.11 provides metadata for all PCP participants and specialist physician raters involved in the OSCE study.
- Section SI.12 provides metadata about the size of the corpus of clinical guidelines available to AMIE and PCPs in the OSCE study.
- Section SI.13 provides an overview of all scenarios used either in the OSCE study (N=100) or for validation purposes (N=20).
- Section SI.14 provides an ablation analysis of various model and agent configurations based on an entirely AI-simulated end-to-end re-creation of our OSCE study and corresponding auto-evaluations.

RxQA Benchmark:

- Section SI.15 presents an example RxQA question (Figure SI.2) and breakdowns of results on this benchmark comparing AMIE and PCP in detail (Table SI.33), as well as in comparison with other models (Table SI.34).
- Section SI.16 provides more details and prompts to explain the development of the RxQA benchmark.

SI.1 Related Work

SI.1.1 Management Reasoning

Early work in management reasoning focused on the adaptation of decision theory to medicine in easily categorized decisions with clear “right” answers, such as laparotomy in acute abdomen [63] or antibiotic selection for already-identified microorganisms [64]. More recent approaches have been grounded in cognitive psychology, in particular how human clinicians process and store clinical information [65]. Clinicians group both diagnostic and management information into semantic structures known as “scripts” [66]. The nuance and accuracy of scripts is highly influenced by experience, but also subject to considerable contextual factors as well as cognitive biases, heuristic short cuts that can lead to diagnostic and management errors [67].

SI.1.2 Management Reasoning by AI Systems

Early management reasoning systems used Bayesian inference and rules-based systems to model management decisions in limited domains [68]. LLMs show human-like abilities in many specific management tasks, such as triaging emergency room patients for admission [69], extracting oncological information from radiology reports [70] or navigating general clinical encounters with uncertainty [10]. They perform less well in more generalized reasoning tasks, erring on the side of caution in high-stakes settings [71] or failing to select the appropriate medical calculators for clinical prediction tasks [72]. Computational technologies such as retrieval-augmented generation [73] (RAG) allow for models to take advantage of clinical guidelines and other expert-curated materials; RAG-based systems outperform base models on a variety of clinical tasks [74] and hold considerable promise for management reasoning. Data from clinical trials is lacking for LLM clinical decision support systems; a number of trials have been registered and are recruiting patients, but several factors, including privacy rules, have slowed study [75].

SI.1.3 Conversational AI and Goal-oriented Dialogue

The current ability of LLMs to prospectively collect data is hotly debated; automated evaluations using AI agents show that the diagnostic accuracy of general purpose LLMs falls considerably when actively collecting information compared to being presented with vignettes or clinical documentation [76]. However, conversational models have already been clinically deployed [77], and data from a virtual primary care clinic shows that conversational AI approaches human diagnostic accuracy for a variety of common primary care concerns, though with considerable heterogeneity by diagnosis [78]. Real-world integration of an AI agent into patient chats show high levels of accuracy and safety, with improved patient satisfaction compared to the previous human baseline [79]. While these systems integrate hybrid clinician/AI teams in conversations with patients, AMIE has shown superior diagnostic accuracy and patient-centric behavior in text-based conversation with patient actors compared to human PCPs across a broad array of diagnoses [1]. A recent prospective, single-arm feasibility study of AMIE demonstrated the system’s ability to conduct clinical history taking and presentation of potential diagnoses for patients to discuss with their provider at urgent care appointments at a leading academic medical center, in a safe manner [3]. In this study, human safety supervisors monitored all patient-AMIE interactions in real time and did not need to intervene to stop any consultations based on pre-defined criteria, and blinded assessment of AMIE and PCP Mx plans suggested similar Mx plan quality, without significant differences for Mx appropriateness and Mx safety, in this real-world single-visit setting.

SI.1.4 AI for Medical Consultations

Providing LLMs to generalist physicians improved management in difficult cases with no right answers, largely by prompting physicians to better consider patient-specific factors [10]. When instructed to solve complex subspecialist cardiology cases with AMIE, the management quality of general cardiologists increased in 64% of cases, decreasing in only 3% of cases [40]. In particular, AMIE served as an initial, highly sensitive diagnostic screen, with generalist knowledge serving as a highly specific confirmatory test to increase accuracy.

SI.1.5 Evaluation of Dialogue for Disease Management

The most common evaluation method for clinical dialogue is the Objective Structured Clinical Examination (OSCE) [80]. OSCEs are simulated patient encounters, in which a student performs a diagnostic and management interview with a trained patient actor. OSCEs cover a variety of encounter types, including counseling, end-of-life conversations, and trauma-informed care, as well as more traditional diagnostic and management encounters. Due to their similarity to actual clinical encounters and reliability, OSCEs have become commonplace in physician licensing assessments across the world, including the USMLE in the United States and PACES in the UK [81]. The dynamic nature of clinical conversations makes "ground truth" evaluation difficult, so OSCEs are graded both via case-specific rubrics and global assessments [82, 83].

SI.1.6 Multi-Agent Systems and Action Planning

Multi-agent systems can be separated into two groups: simulated environments in which autonomous AI agents interact [84–86], and cooperative multi-specialist systems [45, 87–89]. Mukherjee et al. [88] introduced Polaris, a constellation of specialists (for checklists, medications, labs, vitals, etc.) supporting a single user-facing agent with promising performance in medication safety, clinical conversations, and bedside manner when compared with nurses. Gottweis et al. [45] studied a cohort of specialized agents capable of reasoning and interacting with tools to explore scientific problems. Christakopoulou et al. [89] proposed a system distributing work between a fast user-facing system 1 (Talker) agent, and a slower system 2 (Reasoner) in the context of sleep coaching. The Reasoner plans the conversation ahead by autonomously interacting with tools and memory. Planning actions using LLMs has been explored in the context of embodied agents [90–92]. AMIE's Mx Agent is an example of an agent planning actions (e.g., investigations or treatments) to inform the care journey and which are delivered in a conversational manner through the Dialogue Agent.

SI.1.7 Reasoning, Test-Time Scaling and Knowledge Exploration

Foundation models and reasoning techniques, particularly extensions of chain-of-thought, self-consistency and refinement [41, 55, 56, 58], have long been leveraged in challenging medical benchmarks [25, 42, 46, 93] like the MedQA-USMLE [60]. Building upon these foundational approaches, agentic frameworks that interleave reasoning and retrieval [47, 94–96] have driven further advancements in science and medicine. Med-Gemini and AI co-scientist [23, 45] demonstrated further improvements by scaling test-time computation [43, 44], enabling a broader exploration of the solution space. Operating under real-time dialogue constraints, AMIE's Mx Agent simultaneously explores multiple patient care strategies and leverages its long-context capabilities to refine these intermediate solutions using extensive authoritative knowledge. We investigated the use of structural constraints to enhance control over the reasoning process, a technique also explored in [97, 98]. While structural constraints are valuable for tasks involving complex outputs and time constraints, such as real-time patient management, the most powerful reasoning methods may be those that can automatically, predictably, and adaptively scale their cognitive processes to the demands of the task. Recent advancements in emerging "thinking" models such as Gemini 2.0 Flash Thinking [20], OpenAI-o1[21], and DeepSeek-R1 [22] are exploring this frontier.

SI.2 Example Management Plan

Table SI.1 | A management plan generated for a fictive case of undiagnosed Pheochromocytoma. Management plans delineate the patient's clinical journey. Plans schedule both in-visit and post-visit actions. Each management item comes with generated references to guidelines for further interpretability. The plans are generated by the Mx agent based on a set of retrieved guidelines. The identical plan structure is used throughout evaluations. Note the imperfection that recommendations in this sample plan do not include genetic testing which could be considered appropriate in this setting. In addition, AMIE could have explicitly considered whether the patient is in a hypertensive crisis.

Category	Items	References
**In-visit investigations**	1. Assess Gurmeet's headaches in detail: onset, duration, location, character, severity, associated symptoms (nausea, vomiting, photophobia, phonophobia), and any triggers.	–
	2. Inquire about other symptoms suggestive of pheochromocytoma: postural hypotension, abdominal pain, pallor, anxiety, and changes in vision.	`bmj163`
	3. Review Gurmeet's medication history, including over-the-counter pain relievers, to identify potential interactions or side effects.	–
	4. Measure Gurmeet's blood pressure in both arms, heart rate, respiratory rate, and temperature.	`ng136`, `bmj26`
**Ordered investigations**	1. Order a 12-lead ECG to assess for cardiac arrhythmias.	`bmj572`
	2. Order a comprehensive metabolic panel including fasting blood glucose, HbA1c, lipid profile, and thyroid function tests (TSH, Free T4).	`bmj26`, `ng136`
	3. Order plasma free metanephrines and 24-hour urine fractionated metanephrines and normetanephrines to assess for pheochromocytoma.	`bmj163`, `ng136`
	4. Order a renal function test (serum creatinine, eGFR, and urine albumin:creatinine ratio).	`bmj26`, `ng136`
	5. Consider abdominal CT or MRI scan if initial tests suggest pheochromocytoma.	`bmj163`
**Ordered interventions and recommendations**	1. Advise Gurmeet on lifestyle modifications: reduce sodium intake, maintain a healthy diet (DASH diet), increase physical activity, and moderate alcohol consumption.	`ng136`, `bmj26`
	2. Prescribe a short course of over-the-counter analgesics (e.g., paracetamol) for headache relief while awaiting test results.	–
	3. Schedule a follow-up appointment in 1-2 weeks to review test results and discuss further management.	–
	4. If pheochromocytoma is confirmed, refer Gurmeet to an endocrinologist and surgeon for specialized management.	`bmj163`
	5. Educate Gurmeet about the importance of monitoring his blood pressure regularly at home and seeking immediate medical attention if he experiences severe headaches, palpitations, or excessive sweating.	`ng136`, `bmj26`, `bmj163`

Management plans state the recommended sequence of actions for the patient to follow. These plans schedule future actions across short and longer term time scales, which is crucial for effective disease management. We categorize these actions into

1. a set of **in-visit investigations**, which are simple tests or questions that must be conducted with the patient while conversing through the online interface (e.g., measure body temperature with a home thermometer, ask about recent symptoms)
2. a set of **post-visit actions and recommendations**, which generally require physical interaction, often with healthcare providers, or lifestyle change and need to be executed or started before the next follow-up visit. The post-visit actions are themselves divided into two categories:
   (a) the **ordered investigations**, which summarize a number of relevant tests requiring formal orders, such as ordering blood work or running an MRI scan (aiming to gather more diagnostic information)
   (b) the **ordered interventions and recommendations**, which summarize clinical recommendations and other interventions requiring formal orders, such as prescribing a drug or even referring to surgery (representing direct therapeutic actions).

For example, an in-visit investigation might be to "ask about recent travel history", while an ordered investigation could be "order a chest X-ray", and an ordered intervention might be "prescribe an antibiotic". To trace clinical decisions back to clinical guidelines, each plan item comes with citations to clinical guidelines. Table SI.1 shows an example of a management plan generated for a fictive case on undiagnosed Pheochromocytoma.

SI.3 Mx Agent Design and Auto-Evaluation

We collected a separate set of 20 validation scenarios (protocol described in Section 5.5.1), which we used as a validation set to test various Mx agent designs. Each scenario decomposes into three subsequent visits, each visit comes with a text-based case description, a list of reference guidelines, and a reference management plan. We assessed the Mx agent in isolation using, for each visit, the scenario descriptions as input and using the list of reference guidelines and the reference management plans as the prediction targets.

SI.3.1 Evaluation Measures

We measured guideline retrieval performance based on precision and recall, evaluated based on both the set of retrieved guidelines and the citations generated along with the predicted management plans. To evaluate the quality of the plan, we asked Gemini 1.5 Pro to pick a winner among the generated plan and the reference one for each of the nine following categories: *best overall management plan*, *best guidelines alignment*, *most appropriate investigations*, *most appropriate interventions*, *most detailed investigations*, *most detailed interventions*, *most appropriate follow-up*, *best risk management* and *safest management plan*. The win-rate was estimated for each category by randomly shuffling the predicted and target plans in the prompt (n=4). The full auto-evaluation prompt is presented at the end of this section. We report metrics with in-context reference guidelines and with a range of in-context retrieved guidelines, measured by their total number of tokens. We acknowledge the limitations of this evaluation tool due to self-enhancement and verbosity biases [53, 54]. The Mx auto-evaluator prompt is provided in Table SI.2.

SI.3.2 Agent Designs

We explored diverse reasoning structures, ensemble refinement [46] and varying numbers of context lengths. We explored reasoning structures varying from simple chain-of-thought [55, 56] to more complex reasoning trees designed to force the model to interleave in-context retrieval with reasoning. Our search space is limited by serving constrains, which are the maximum number of parallel model calls (set to four) and a response time (targeted to approximately one minute).

Implementation details When using ensemble refinement, we generate plans stochastically without conditioning on retrieved documents. This allows for concurrently retrieve and draft, reducing the overall response time. The drafts are refined into a final plan using a final model call, conditioned on the full text of the retrieved guidelines.

When retrieving guidelines based on text embedding, for each query, we separately rank clinical guideline sources based on text embeddings. The final list of guidelines is obtained by iteratively taking the next highest-ranked guideline from each available guideline corpus until the maximum token limit is reached.

SI.3.3 Results

Figure ED.2 summarizes the retrieval and auto-evaluated management metrics for a subset of methods: a simple chain-of-thought method without drafting, structured reasoning without drafting, and structured reasoning with drafting (see Figure 2 for the definition of the reasoning structure).

Retrieval Performance The coarse retrieval step (query generation + text-embeddings) is sufficient to score a recall superior to 90% using a context size of 256k. Recall slightly decreases when measured based on generated citations. However, the F1 score measured based on citations is higher for larger context sizes than it is when measured on the coarse retrieval step, suggesting that our language model discriminates part of the irrelevant guidelines via in-context processing. Furthermore, it is important to note that retrieval performances are measured based on reference guidelines, which only represent one valid set of guidelines. Therefore precision and F1 score must be interpreted with care as the rate of false positives is likely over-estimated.

Management Reasoning Performance Across all evaluation axes, Gemini favors the generated management plans over the reference ones with an average win-rate ranging from 75% to 95% for all methods. This corroborates previous observation that auto-evaluators favor synthetic data [53, 54]. In spite of this bias, the

win-rate allows us to discern several trends. Firstly, guidelines retrieval clearly suggests an improvement in management performance. Secondly, structured reasoning improves over a classic chain-of-thought strategy. Thirdly, drafting plans (ensemble refinement) yields an additional performance boost. Fourthly, management quality scales with context size up to a critical context size that differs depending on the choice of method. Last, we measured improvements over the oracle (feeding the reference guidelines), both suggesting that non-reference guidelines are helpful and that retrieval performance improves past 64k tokens.

SI.3.4 Selected Design

Based on this analysis, and after qualitatively reviewing five samples with clinicians, we identified the optimal configuration: using drafts and structuring the model output as detailed in Table SI.7, which scales best with context size and leads to best results across all axes.

Based on the minimum win rate across all axes, we fixed the context size to 256k tokens. We found that, based on qualitative assessments, even if the drafts are not generated based on the retrieved documents, ensemble methods allow generating more comprehensive plans, covering a wider range of often complementary care and investigation strategies.

Running a single structured plan generation step takes around 50 seconds for a context size of 256k tokens, and around 30 seconds without in-context documents. A full Mx agent call, that chains retrieval, four draft generations and a final refinement step takes around 80 seconds.

Mx plan auto-evaluation.

```
You are an expert physician with extensive clinical expertise and up-to-date knowledge of the medical literature.  Your role is to evaluate a management plan
for a patient.
You are given a patient case presentation, which may include a patient profile, and a history of visits, including prior management plans.
Based on the most recent patient visit, you are given two management plans:  A and B. Your task is to compare the two plans and rate them relative to each
other.
When detailing your analysis, prefer longer, detailed, methodological and meticulous analysis.

## Available data:
  • Expert analysis:  You may be provided with expert analysis, this will provide additional context about the case, the patient and management priorities.
  • Guidelines:  You may be provided with reference clinical guidelines, in which case you should use them to ground your evaluation.
## Evaluation criteria

Center your evaluation around the following criteria:
  1. best_guidelines_alignment:  which plan follows the evidence-based clinical guidelines more closely?
  2. most_appropriate_investigations:  which plan provides the most relevant investigations?
  3. most_appropriate_interventions:  which plan provides the most appropriate interventions?
  4. most_detailed_investigations:  which plan provides the highest level of detail for investigations?  E.g., if a test is ordered, is the name of the test,
     the type of test and the purpose clear?
  5. most_detailed_interventions:  which plan provides the highest level of detail for interventions?  E.g., if a drug is prescribed, is the name of the drug,
     the dosage and the purpose clear?
  6. most_appropriate_follow_up:  which plan details the follow-up visit the most appropriately?
  7. best_risk_management:  which plan manages the risk the most appropriately?
  8. best_overall_care_plan:  which plan provides the best overall care for the patient?
  9. most_safe:  which plan is the safest for the patient?
For each criteria, pick the winning plan as A or B.

=== Case presentation ===
{case_presentation}
=== Reference clinical guidelines ===
{guidelines}
=== Expert analysis of the clinical case ===
{analysis}
=== Plan A ===
{plan_a}
=== Plan B ===
{plan_b}
=== Your task ===
Rate the management plan by filling in the following JSON template:
{json_schema}
```

Generation constraint:

```
AorB = Literal['A', 'B']

class AutoevalMxRating:
  # Detail your analysis step-by-step.
  best_guidelines_alignment_analysis: list[str]
  # which plan follows the evidence-based clinical guidelines more closely?
  best_guidelines_alignment: AorB
  # Detail your analysis step-by-step.
  most_appropriate_investigations_analysis: list[str]
  # which plan provides the most relevant investigations?
  most_appropriate_investigations: AorB
  # Detail your analysis step-by-step.
  most_appropriate_interventions_analysis: list[str]
  # which plan provides the most appropriate interventions?
  most_appropriate_interventions: AorB
  # Detail your analysis step-by-step.
  most_detailed_investigations_analysis: list[str]
  # which plan provides the highest level of detail for investigations?
  most_detailed_investigations: AorB
  # Detail your analysis step-by-step.
  most_detailed_interventions_analysis: list[str]
  # which plan provides the highest level of detail for interventions?
  most_detailed_interventions: AorB
  # Detail your analysis step-by-step.
  most_appropriate_follow_up_analysis: list[str]
  # which plan plans the follow-up visit the most appropriately?
  most_appropriate_follow_up: AorB
  # Detail your analysis step-by-step.
  best_risk_management_analysis: list[str]
  # which plans manages the risk the most appropriately?
  best_risk_management: AorB
  # Detail your analysis step-by-step.
  most_safe_analysis: list[str]
  # which plan is the safest?
  most_safe: AorB
  # Detail your analysis step-by-step.
  best_overall_care_plan_analysis: list[str]
  # which plan provides the best care?
  best_overall_care_plan: AorB
```

Table SI.2 | Mx plan auto-evaluation. Assesses relative management reasoning performances using LLM as a judge. `case_presentation` is a full-text description of the fictive scenario, written by experts. `plan_a` and `plan_b` are either a generated plan, or the expert-written reference plan, in random order. The `guidelines` variable corresponds to the full-text of the reference guidelines accompanying that scenario. The variable `analysis` is an expert-written paragraph of text supporting the choice of management plan and alignment with guidelines.

SI.4 Mx Agent Prompts

In this section, we present the prompts and decoding constraints used by the Mx agent (Section 5.3):

- Table SI.3 is the prompt and constraint utilized to preprocess reference documents prior to retrieval.
- Table SI.4 us the prompt and constraint utilized to generate retrieval queries.
- Table SI.5 is the main prompt, used to reason and generate structured management plans.
- Table SI.6 a suffix to the plan generation prompt, appended when refining drafts.
- Table SI.7 the decoding constraint applied for plan generation.

Mx agent prompt: generate document titles and abstracts.

```
You are an expert librarian.  You are given a document.  Your task is to write a title and abstract for
that document.  The abstract will be used by medical practitioners to understand whether the document
is relevant to their patients.

=== Rules ===

Make sure the abstract contains the following information:
  1. For clinical guidelines:
      (a) The population to which these guidelines apply (age, sex, etc.)
      (b) The goals of the guidelines, including the clinical questions they answer.
      (c) The list of conditions, interventions and drugs mentioned in the document.
  2. For drug labels:
      (a) The brand and generic names for that drug (when available).
      (b) Detail when, why, and to whom the drug is recommended.
      (c) Mention the main counter-indications for that drug, if any.
      (d) Mention the main side-effects.
In all cases, the abstract must be:
  1. Maximum 1,000 words.
  2. Make sure the abstract is self-explanatory.
If the title is provided at the top of the document, use it.  Otherwise, come me up with a title
following these rules:
  • The title should be descriptive and self-explanatory.
  • The title should mention the condition targeted by the document.
=== Document ===

{document}

=== Response ===

Answer by filling in the following JSON schema:

{json_schema}
```

Generation constraint:

```
class GeneratedFields:
  title: str
  abstract: str
```

Table SI.3 | Mx Agent prompt: generate document titles and abstracts.

Mx agent prompt: generate search queries.

```
You are a medical expert which role is to analyse the medical history of a patient and come up with a
differential diagnosis and a management plan.

=== Your patient ===
{case_presentation}

=== Your task ===

What do reference guidelines should your refer to in order to best manage this patient?  Based on the
patient case presentation and patient history, generate a list of search queries to find the most
relevant evidence-based clinical guidelines.
Your queries will be executed by a specialized medical search engine only indexing curated clinical
guidelines.

Adhere to the following rules:
    • Write your queries in natural language.
    • Don't mention any guideline provider name in the queries
    • Make sure each query mention a condition or clinical management aspect and the relevant patient's
      characteristics (e.g., age, gender, etc.)
    • Prefer comprehensive and concise queries.
    • Limit the number of queries to 5:  pick your queries wisely and maximize coverage.
    • Consider both the most likely and the less likely diagnoses for that patient.
    • Consider prior conditions and current symptoms that still need to be managed.
    • Consider treatment-specific guidelines, e.g., for a type of drug or procedure.
    • Consider possible drug interactions and counter-indications.
=== Response format ===
Answer by filling-in the following JSON schema:

{json_schema}
```

Generation constraint:

```python
class SearchQueries:
    search_queries: list[str]
```

Table SI.4 | Mx Agent prompt: generate search queries.

Mx agent prompt: generating management plans.

```
Read and memorize the following reference clinical documents.  Each document comes with a unique ID (DOC_ID) and TITLE structured as follows:
"‘
--
# DOC_ID=(id) | TITLE: "(title)"

(content)

END of DOC_ID=(id)
"‘
=== Reference documents ===
{documents}

=== Your Task ===
You are an experienced general practitioner in charge of managing a patient (you are the patient’s doctor).
Based on the patient case presentation, differential diagnosis (if provided), patient history, and external reports (e.g., pathology report, surgery report,
lab results, scan results, etc.), write a management care plan grounded in evidence-based clinical guidelines.

=== Your patient ===
{case_presentation}

=== Rules ===
Assess the patient’s clinical case, including differential diagnosis, patient history, external reports and prior management plans, then write a management
care plan.  You might be provided with information about previous visits you have conducted with the patient (including prior management plans and differential
diagnoses), in which case you should make sure to follow up on the prior plan.  When detailing your reasoning, break down your reasoning into multiple logical
units.  Prefer longer, detailed, methodological and meticulous thinking.
Adhere to the following rules when writing your plan:
  1. Don’t forget the basics:
       • Remember your medical training.
       • Common sense is your friend.
       • Don’t skip the basics (e.g., do you need to measure vitals?  is there any at-home test that can be done?)
  2. Be a clinical strategist:
       • Make sure your plan aims at resolving the differential diagnosis, managing the current symptoms, and preventing further complications.
       • Can you rule out a condition in the differential?  Should you order a test for it?  Is there benefit to doing so ahead of time?
       • Does the plan need to be adjusted to account for the patient history or chronic conditions?
       • Think ahead, are there any pre-existing conditions that might contraindicate interventions?
  3. Assess risks, be responsible, limit burden:
       • Is risk well managed?  Is your plan increasing or decreasing risk?
       • Factor risks in your decisions:  assess risks associated with investigations, tests, procedures and drugs.
       • In clinical practice, time is of the essence, take action early when possible.
       • If prescribing or continuing prescribing a drug, are there any counter-indications or interactions that should be considered?  Should you order
         tests to confirm the counter-indications?
       • Think twice before prescribing a test.  Is it necessary?  Is it the right timing?
  4. Be thorough in your assessment and provide comprehensive care:
       • Factor in the differential diagnosis, case presentation and patient history, including comorbidities.
       • Make sure to address the whole differential, rule out what can be ruled out.
       • Be mindful of the current symptoms and the patient’s past history of complications.
       • when considering a drug, always review the drug history and watch out for interactions.
       • Is there any specific symptom that needs attention?
       • Is there any issue with the ongoing treatment plan or drug prescription?
       • Are there excluding causes or warning signs that should be considered?
  5. Own the clinical management process, take action:
       • Prescribe the relevant test, drugs, procedures and referrals.
       • Only refer to external care when necessary.
       • Is there anything that can be done to improve the patient’s quality of life right now?  If so, include it in the plan (e.g., over-the-counter
         medicines, lifestyle changes, etc.)
       • Is there anything that should be shared to avoid future complications?  (e.g., knowing when escalation is needed).
  6. Structure your response:
       • One item, one action:  each item should have meaning on its own.
       • Sort management items by priority (for each category, report most significant management items first).
       • Although you might detail your reasoning prior to writing the plan, only the plan will be provided to the patient:  don’t leave information behind,
         include all important management information in the plan.
  7. Level of detail:
       • be specific when listing action items (e.g., prefer "Order blood tests X, Y and Z." instead of "Order blood tests.")
       • When prescribing a test, specify the test name, the type of sample to be collected and the purpose.
       • When prescribing a drug, specify its name, the dosage and purpose.
       • Mention when to schedule the follow-up visit, specify a range of time (e.g., "in 1-2 days", "in 2-4 weeks")
  8. Timing of action:
       • Each "in_visit_investigations" item is to happen BY THE END of this visit, this means it can be done by the patient at home.
       • Each "ordered_investigations" item is to be done AFTER the visit and before the next visit.
       • Each "appropriate_recommendations_or_actions" item is to be done AFTER the visit and before the next visit.
       • ordered investigations and actions should be planned independently of the outcome of the in-visit investigations.
  9. Clinical setting:
       • The visit is conducted over an online interface, only at-home investigations and tests are available during the visit, other tests need to be
         ordered.
       • You may be provided with "external reports".  These reports summarize investigations or interventions that have already been conducted and provided
         you with key information about the current patient’s state.
       • When previous visits are reported, then this is a follow-up visit.  Make sure to plan for the current visit and to be consistent with the prior care
         plan.  Consider additional investigations and interventions.
 10. Citations:
       • When asked to provide citations, refer to the reference document IDs (e.g., "bmj123", "ng123", "uidXYZ", etc.).
       • Only provide citations when supporting information can be found in the reference documents.
=== Response format ===
Answer by filling-in the following JSON schema:
{json_schema}
```

Table SI.5 | Mx Agent prompt: generate management plan.

Mx agent prompt: refine management plans.

```
=== Draft Plans ===

If available, you will be presented below with draft plans written by different clinicians.  Combine
the plans into a single clinically coherent plan.

Plan merging rules:
  1. You might filter, split, merge, summarize or detail further the items in the draft plans.
  2. Learn from the plans, understand their reasoning and the rationale behind the items, and come up
     with a single unified plan.
  3. Be critical (your pairs are not perfect):  remove items that are not clinically coherent with your
     final plan.
  4. You might have access to additional reference documents, use them to support your final plan.
  5. Be concise:  only retain what matters, aim at single coherent plan.
{mx_drafts}
```

Table SI.6 | **Mx Agent prompt:** refine management plan.

Mx agent: structural constraint.

```
Generation constraint:

# The Citation type is defined based on the
# set of retrieved guidelines and their IDs.
Citation = Literal["bm572", "ng136", "..."]

class ActionItem:
    """A management item with citations."""
    item: str
    citation: list[Citation]

Class ReasoningSteps:
  # How to best manage this patient? Let's think step-by-step.
  analysis: list[str]
  # List what objectives the management plan should achieve.
  management_goals: list[str]

class MxPlan:
  # List of tests or investigations that should be done (at home)
  # during this visit (e.g., measure body temperature, etc.)
  in_visit_investigations: list[ActionItem]
  # List of tests or investigations to be done after the visit
  # (e.g., lab tests)
  ordered_investigations: list[ActionItem]
  # List of interventions to be recommended to the patient
  # (e.g., drugs, surgery, etc.)
  recommendations_or_actions: list[ActionItem]

class PlannerOutput:
 """MxPlan fused with a reasoning steps."""
  reasoning: ReasoningSteps
  mx_plan: MxPlan
```

Table SI.7 | **Mx Agent:** structural constraint. Decoding constraints allow generating structurally consistent plans and enforcing citations that always match the set of retrieved guidelines.

SI.5 Development of Training Dialogues

SI.5.1 Augmenting Single-Visit Dialogues

The original PaLM-2-based version of AMIE described in Tu et al.[1] was trained with both real-world dialogue transcripts and simulated doctor-patient dialogues. The real-world transcripts were noisy, containing frequent interruptions, paraverbal annotations (like '[laughing]'), and many details which were clinically irrelevant or unsuitable for a text-based setting (like physical examination). While generally higher quality, the simulated dialogues also had issues such as a lack of recommendations to the patient. To resolve these issues, we used Gemini 1.5 Pro to rewrite the original dialogues. Furthermore, while the inclusion of follow-up visits is useful for our multi-visit setting, the follow-up visits in the original dataset lacked any context about what had transpired in prior visits. To solve this, we used Gemini 1.5 pro to generate context describing the doctor's prior knowledge about the patient, which was unique per dialogue and provided in the instructions during supervised fine-tuning (see Section SI.6). Prompting details for this process are listed in Section SI.7.1.

SI.5.2 Simulated Longitudinal Interactions

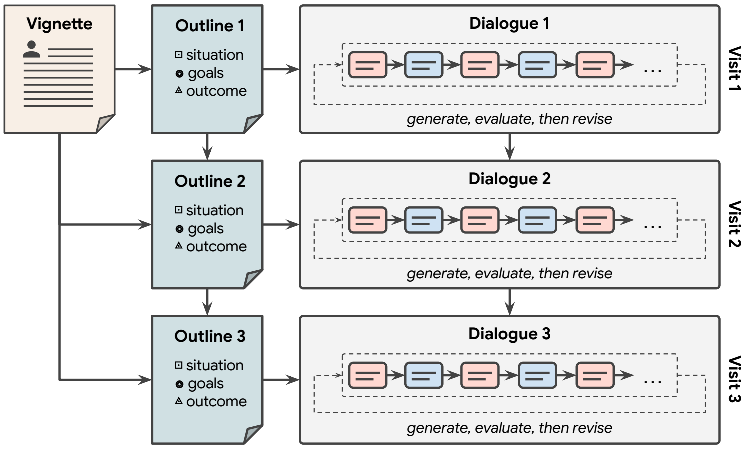

Figure SI.1 | Top-down longitudinal dialogue synthesis. We generate multi-visit doctor-patient interactions based using a top-down approach: first generating a high-level patient vignette, then generating an outline for each visit, finally by generating the doctor-patient dialogue for each visit. Each dialogue is drafted, then auto-evaluated and revised.

We expanded the training set of patient-doctor dialogues with synthetic dialogues simulating the clinical course of patients from their initial presentations to their resolutions. Each simulated dialogue comes with a fictive patient vignette, a patient-doctor dialogue for a maximum of three subsequent visits, and reports summarizing the outcome of the interventions (e.g., surgery) and investigations (e.g., blood work) ordered during the preceding visit, if any.

Inspired by the prior work on co-writing screenplays from Mirowski et al. [57], we designed a top-down synthesis pipeline, starting from a high-level patient vignette to goal-directed visit outlines, and finally down to the dialogue level for each outline (illustrated in Figure SI.1). Building on our prior work [1], we generated a wide range of synthetic patients vignettes grounded in publicly available knowledge retrieved using Google Search. For each vignette, we generated visit outlines one after another for all three visits. Each outline describes the situation at the start of the visit (patient state, including external reports such as lab results or radiology report), defines conversation goals for both the patient and doctor (i.e., patient expectations, doctor strategy) and states the outcome of the visit (i.e., learned patient facts, updated differential diagnosis, follow-up schedule, and ordered post-visit investigations and interventions). Once the visit outlines were generated, for each visit, we drafted the entire patient-doctor conversation using a single model call, which we then automatically evaluated and revised, akin to Madaan et al. [58], based on a selection of high-level criteria (communication, coherence, reasoning, and management quality). All scenarios and dialogues were generated using Gemini 1.5 Pro and constrained decoding [50], which enabled generating complex data

structures (vignettes, outlines, dialogues, evaluations) using fewer model calls, significantly increasing the pipeline efficiency. Prompting details for this process are listed in Section SI.7.2.

SI.5.3 Filtering Dialogues via Auto-Evaluation.

To ensure quality of the synthetic dialogues, we leveraged LLM-based auto-evaluation to assess the quality of the doctor messages in the dialogues across various criteria with regard to hallucinations and bias, ethical behavior, quality of history taking, providing information, building rapport and empathy, and so on. We only included the dialogues which passed this set of auto-evaluation criteria in the subsequent instruction fine-tuning stage. Specifically, the auto-evaluation was performed using a four-criteria system:

- **Undesirable doctor agent behavior:** This includes providing unsafe, biased, or non-evidence-based statements; exhibiting ethical or safety concerns; inadequately differentiating normal versus unhealthy cases; prematurely offering diagnoses without sufficient history-taking (except in emergencies); and incorrectly suggesting the ability to prescribe medications.
- **Desirable doctor agent behavior:** Similar to the above mentioned criteria but we inversely evaluated the criteria, ensuring a comprehensive capture of potential issues by also focusing on what constitutes positive and appropriate doctor behavior.
- **Hallucination and confabulation:** This criterion focuses on whether the doctor agent introduces information not explicitly or implicitly provided by the patient, mistakenly assumes or recalls information, misremembers/misunderstands/misinterprets patient input, claims access to external databases or services, or mentions non-existent medications.
- **Dialogue formatting:** This checks for question or information repetition, the balance of turns between patient and doctor, and adherence to minimum and maximum turn limits of the dialogue.

Detailed auto-evaluation prompts are listed in the Appendix Section SI.8. For each of these criteria, a binary result is generated. Any dialogue receiving an unfavorable result in any category was filtered out, while the rest were retained and utilized for fine-tuning. The auto-evaluation process was done by Gemini 1.5 Pro with the constrained decoding framework [59].

SI.6 Dialogue Agent Post-training

SI.6.1 Supervised Fine-tuning

We fine-tuned on top of the base model Gemini 1.5 Flash to adapt it for conversational and longitudinal patient interactions. The medical question-answering and summarization tasks were set up as in Tu et al. [1], where task-specific instructions were prepended to each question-completion pair.

For single-visit dialogues, AMIE was trained to predict the next conversational turn based on all previous turns, assuming the role of the doctor. Here, we prepended instructions that described the task of interviewing the patient. Furthermore, for follow-up visits, our instructions contained scenario-specific information which was unique for each dialogue and was generated to contextualize the visit (see Section SI.5.1).

Finally, for the longitudinal multi-visit dialogues described in Section SI.5.2, we concatenated the sequence of visits into a single multi-visit transcript. Between each visit, we inserted the inter-visit context, such as reports from test results or updates which would be available to the doctor during the following visit. We again sampled doctor turns as targets, training AMIE to predict the next conversational turn based on the preceding multi-visit dialogue history.

SI.6.2 Reinforcement Learning

To further align the model's outputs with the desired preferences on conversational diagnostic and management reasoning, we employed reinforcement learning from feedback [38], with a hybrid of both human and LLM preferences. The process includes two phases—reward model training, and reinforcement learning. The reward model predicts how a rater (human or LLM) would rate a given response, which is then used in the reinforcement learning stage such that the policy model (the dialogue agent) will be optimized to generate outputs that receive high scores judged by the reward model, thus indirectly aligning with the rater preferences.

We first trained a Gemini 1.5 Flash-based reward model using clinically relevant pairwise preference data from two distinct sources:

- **Preferences from human experts:** We curated three pairwise preference datasets annotated by clinical experts including: (1) Single-visit dialogue response preference task, (2) AMIE cardiology case evaluation task [40], and (3) AMIE breast cancer case evaluation task [39].
- **Preferences from LLM:** Three pairwise preference datasets rated by LLM were curated where we utilized Gemini 1.5 Pro as an auto-rater to provide AI-generated preferences between pairs of generated responses, including: (1) Single-visit dialogue response preference task (Gemini 1.5 Pro acting as the judge), (2) MedQA medical reasoning task, and (3) electronic health record (EHR) summarization task.

Following the supervised fine-tuning, we next refined the dialogue agent (policy model) via reinforcement learning using the reward signal provided by the previously trained reward model. For this phase, we utilized (1) a separate set of single-visit dialogue examples similar to the ones used in the SFT stage, along with (2) the MedQA medical reasoning examples [60]. The process proceeded as follows: a prompt was sampled from this dataset and presented to the policy model to generate a response. The reward model evaluated this response, assigning a scalar reward value. Finally, the reward signal was used to update the parameters of the policy model via the reinforcement learning algorithm used in Gemini 1.5 [4]. This process allowed the dialogue agent to learn to generate responses that maximized the reward, leading to improved performance. Further details of the data used for reward model training and reinforcement learning are listed in Table SI.8 and Table SI.9.

SI.6.3 Dataset information for reinforcement learning from feedback

Task	Sample size	Description	Response 1	Response 2	Rater
Single visit dialogue response preference (human)	470	A case vignette, a generated dialogue up to the last user turn, and a pair of responses are given to the rater to decide the preference. All dialogues are derived from 464 common medical conditions.	AMIE	Gemini 1.5 Pro	Human
Cardiology case management preference	1473	A cardiology case, and a pair of management recommendations. Note that the AMIE responses had been rewritten to the same style as the expert responses since the expert responses are shorter and more specific. The expert response is by default the better response.	AMIE	Human	Human
Breast cancer case management preference	100	An oncology case, and a pair of management recommendations. The expert response is by default the better response.	AMIE	Human	Human
Single visit dialogue response preference (LLM)	2184	A case vignette, a generated dialogue until the last user turn, and a pair of responses. An LLM rater outputs a preference score for each response, ranging from 1 to 7. Dialogues are derived from 464 common medical conditions and 628 uncommon conditions.	AMIE	Gemini 1.5 Pro	Gemini 1.5 Pro
MedQA medical reasoning preference	191	A MedQA question and the corresponding answer, and a pair of answer explanations. An LLM rater outputs a preference score for each response, ranging from 1 to 7.	MedPaLM 2	MedPaLM 2	Gemini 1.5 Pro
EHR summarization preference	65	A clinical text, and a pair of summarized texts. An LLM rater outputs a preference score for each response, ranging from 1 to 7.	MedPaLM 2	MedPaLM 2	Gemini 1.5 Pro

Table SI.8 | Pairwise preference datasets for reward model training. Preference data mixture for training the reward model.

Task	Sample size	Description
Single-visit dialogue response generation	13104	Given a case vignette and a generated dialogue until the last user turn. The prompt asks the model to play the medical expert role and provide the appropriate response for the prior turns. All dialogues are derived from 464 common medical conditions and 628 uncommon conditions.
MedQA medical reasoning explanation preference	191	Given a MedQA question and the corresponding answer, the prompt asks the model to generate an explanation of the medical reasoning of the answer.

Table SI.9 | Datasets for reinforcement learning.

SI.7 Prompts for Dialogue Generation

SI.7.1 Single-Visit Dialogue Rewrite Prompts

The single-visit dialogues initially introduced in [1] were revised by Gemini 1.5 Pro to improve their quality and contextualize them (see Section SI.5). Example outputs are shown in Table SI.13.

1. For real world dialogues, an outline of the original transcript is generated, with modifications made to adapt it to a virtual, text-based setting (Table SI.10).
2. Gemini revises each dialogue to improve its suitability for instruction fine-tuning. For real-world transcripts, Gemini leverages the outline generated in the prior step to structure its revision (Table SI.11).
3. For each revised dialogue, Gemini generates fictive context detailing the information which was known to the doctor prior to that conversation, which is utilized during supervised fine-tuning (Table SI.12).

SI.7.2 Simulated Multi-visit Dialogue Generation Prompts

In addition to the single-visit dialogue rewrites, we also simulate sequential, multi-visit dialogues by first generating N visit outlines using the prompt presented in Table SI.14 based on patient vignettes produced in [1]. Given the generated outlines, we generate the dialogues visit by visit. For each visit, we generated a dialogue draft using a single model call using the prompt presented in Table SI.15, which we then auto-evaluate using Table SI.16, and refine using Table SI.15 again. Example outputs for the multi-visit training dialogue generation are shown in Table SI.17.

Reorganizing Real-world Single-Visit Dialogue Transcripts

```
You are a helpful AI assistant. I am analyzing transcripts of conversations between patients and their
doctor, who is taking their medical history.

The transcripts are quite noisy, and I'm trying to understand everything that was discussed in the
conversation. Please analyze the transcript and write up a detailed outline of what was discussed.
However, you should make some slight modifications to the outline so it could realistically occur over
a virtual chat based interface:

  • At the start of the conversation, the doctor should greet the patient and allow them to open with
    their chief complaint or updates.
  • The doctor should not perform any type of physical exam. In certain cases, this could be replaced
    by the patient mentioning a physical finding or the doctor asking the patient, at home, to do
    something to check for a physical finding.

Transcript:
{transcript}

Patient Info:
{patient_metadata}

=== Outline structure ===
  1. The outline should be written up approximately chronologically describing what details were
     discussed.
  2. You should ensure you include any symptoms or clinically relevant patient details revealed in
     the transcript, as well as any diagnoses which are made or inferred, and any next steps for
     investigation, testing, and treatments.

=== Example Output Format ===
  • At the start, the doctor asks the patient about A
  • The patient reveals B
  • The doctor inquires about C, D, E
  • (...)
```

Table SI.10 | Single-Visit Dialogue Rewrite. Generating an outline to reorganize real-world dialogue transcripts

Revision of Single-Visit Dialogues

```
You are a helpful AI assistant. I am rewriting transcripts of conversations between patients and their
doctor, who is taking their medical history.

The transcripts are quite noisy, and the doctor's messages are not as empathetic and well structured
as I would like. I would like to rewrite the conversation to make the doctor a lot more empathetic,
the conversation a lot more readable and resemble a conversation which would take place over a virtual
chat interface. Please help me rewrite the conversation using the following guidelines:

Transcript:
{transcript}

Additional Context:
{outline}

=== Guidelines for the rewritten dialogue ===
```

- Every message from the doctor should be warm, empathetic and varied (but no emojis). There should be no placeholders such as '[laughing]' etc.
- The conversation should have a clear, logical flow. It should make sense to read.
- The content discussed, and any clinically relevant details that the doctor asks and the patient revealed should be the same after revision. However, you can combine a few questions together to improve the conversational flow and make it sound less repetitive.
- There are two types of details which may be removed. First, details which are completely irrelevant to the patient's health can be removed. Second, details about physical exams or things that are otherwise impossible over a virtual text-based interface should be removed.
- The patient's messages should be mostly simple and short, using everyday casual language and no medical jargon.
- The doctor's responses should be a longer and very empathetic, acknowledging the patient's concerns. The doctor's responses should be clean and better structured.
- The new conversation should only be between the doctor and the patient (or caretaker). Messages from other participants should be condensed into one of these participants.
- At the end of the conversation, the doctor should describe any next steps the patient should take, and what to expect in terms of further investigation/testing and treatment. Alternatively, if no new changes are needed, say so.

```
=== Output Format ===
**Doctor**: ...
**Patient**: ...
**Doctor**: ...
**Patient**: ...
...
```

Table SI.11 | Single-Visit Dialogue Rewrite. Revising single-visit dialogues for supervised fine-tuning.

Generation of Single-Visit Prior Doctor Context

```
Examine the following medical dialogue between a doctor and patient.  After reading the dialogue,
please list any details that were known or implied to the doctor prior to the start of the
conversation.

In particular, this means any details which the doctor seems to have known about the patient, their
health, or their symptoms prior to the patient offering this information.  If the doctor knows nothing
about the patient, simply say "The doctor knows nothing about the patient".  If it is a follow-up
visit, there are likely several details the doctor knows from previous visit(s); please be thorough in
providing this list.

Dialogue:
{dialogue}

Provide your response as a list in a bulleted markdown format as follows:
Prior knowledge of Doctor:
  • (bullet point 1)
  • (bullet point 2)
  • (...)
```

Table SI.12 | Single-Visit Dialogue Rewrite. Generating additional pre-visit context to augment fine-tuning examples.

Example Snippets of Single-Visit Dialogue Revision Steps

Original transcript:

Patient (USER): Um-hum.
Doctor (ASSISTANT): Um -
Patient (USER): I had my bladder tacked a year ago.
Doctor (ASSISTANT): Did they do the sling or just the -
Patient (USER): Yes.
Doctor (ASSISTANT): Who did it?
Patient (USER): Um, [PHYSICIAN NAME OTHER].
Doctor (ASSISTANT): Um-hum. And did it a year ago, did it help at all?
Patient (USER): Very little, very, very little.
Doctor (ASSISTANT): So you got a, you have a lot of urinary frequency, you lose urine when you cough, sneeze, laugh, strain, just about anything, go upstairs, all of that?
Patient (USER): And all through the night.
Doctor (ASSISTANT): And a lot of urinary frequency at night?
Patient (USER): Um-hum.
...

Outline:

- Introduction and Chief Complaint: The doctor greets the patient and invites them to share their reason for the visit. The patient states they are having trouble with their overactive bladder again.
- History of Present Illness - Onset and Prior Surgery: The doctor inquires about the onset and potential triggers of the current symptoms. The patient explains that the issues began about a year ago and mentions they had corrective surgery (a bladder tack with a sling). The doctor asks for details about the surgery, including the surgeon's name and its effectiveness. The patient identifies the surgeon as [PHYSICIAN NAME OTHER] and reports that the surgery provided very little relief.
- Symptom Characterization: The doctor asks the patient to describe their current urinary symptoms in more detail. The patient confirms experiencing significant urinary frequency throughout the day and night (nocturia). The patient also describes stress incontinence, with urine leakage triggered by activities such as coughing, sneezing, laughing, straining, and going upstairs.
- ...

Revision:

Doctor (ASSISTANT): Hello, I'm AMIE, a medical LLM developed by Google. I'm here to help you understand and manage your health concerns. What can I help you with today?
Patient (USER): I'm having trouble with my overactive bladder again.
Doctor (ASSISTANT): I'm sorry to hear that you're experiencing this. It sounds quite frustrating to be dealing with this again. To help me understand better, could you tell me a little more about: 1) When did you first start noticing these symptoms? 2) Did anything in particular seem to trigger them?
Patient (USER): It started about a year ago. I actually had surgery to correct it - a bladder tack with a sling.
...

Table SI.13 | Illustrative Examples of Single-Visit Dialogue Revision Steps. Here, we present examples outputs from our synthetic data generation pipeline for revising existing single-visit dialogues for supervised fine-tuning. This includes a step to generate a new dialogue outline using the prompt in Table SI.10 and a step to rewrite the existing dialogue in Table SI.11.

Generating Multi-visit dialogue outlines

```
You will be asked to simulate the course of a patient through an online healthcare system.

=== Clinical Knowledge ===

{search_results}

=== Patient ===

Here is a patient with condition: {condition}.
At the first visit, the patient is described:

{vignette}

=== Your task ===

Outline the next (online) medical visits for this patient.
The patient and doctors are interacting through an online interface.
During the first visit:
```

- the doctor doesn't know anything about the patient, not even the patient name and demographics.

During each visit:

- the doctor will review the interventions completed since the last visit.
- the doctor will attempt to identify the correct differential diagnosis by discussing with the patient.
- the goal of the doctor is to make a conservative differential diagnosis, it's better to err on the side of caution.
- they will share the differential diagnosis with the patient.

At the end of each visit:

- the doctor will revisit the differential diagnosis (dropping ruled-out conditions and updating their likelihood)
- the doctor will recommend next management steps (taking lab results, ordering an intervention, etc.).

Number of visits:

- there should be from {min_visits} to {max_visits} visits.

Answer by filling in the following JSON structure:

{json_schema}

Generation constraint:

```python
Likelihood = Literal["Ruled out", "Very unlikely", "Unlikely", "Likely", "Very likely", "Confirmed"]

class Diagnosis:
  condition: str
  likelihood: Likelihood

class Intervention:
  provider: str
  intervention: str
  goal: str
  result: str

class Outline:
  """Outline for a single visit."""
  visit_number: int
  completed_interventions: list[Intervention]
  patient_goals: list[str]
  doctor_goals: list[str]
  discussion_topics: list[str]
  doctor_learned_patient_facts: list[str]
  doctor_learned_patient_symptoms: list[str]
  doctor_inferred_dx: list[Diagnosis]
  planned_interventions: list[str]
  next_visit_schedule: str

class Outlines:
    """Target structure"""
    visits: list[Outline]
```

Table SI.14 | Multi-visit Dialogue Generation. Generating multi-visit dialogue outlines.

Generating or improving multi-visit dialogues

```
You are a medical expert whose role is to write a dialogue between another medical expert and a patient.  You will play both roles, for training
purposes.
=== Rules ===
    • The conversation should be long, from 20 to 40 messages.
    • Both the doctor and patient should write in English.
    • The conversation should be realistic for an online chat interface.
    • Include every line of the conversation without any 'stage notes', placeholders or action descriptions.
    • Roles are 'patient' and 'doctor', nothing else.
    • End the conversation with the tag "END_OF_DIALOGUE" written as a separate message.
    • Don't use emojis.
=== Dialogue simulation task ===
You will be provided with a dialogue outline, play both a patient and a doctor for a single visit.
Make sure this dialogue is coherent with the previous dialogues and outlines, if any.
Make sure the the dialogue is a realistic online interaction between the two protagonists:
    • the doctor can't perform a physical interaction, they can only refer the patient to other specialists or nurses.
    • the doctor doesn't have access to patient's data, except for the ones provided in the outline.
{% if draft }
=== Dialogue improvement task ===
You will be provided with a dialogue outline, a past dialogue, along with a critique.  Re-write the dialogue based on the feedback from critique.
If there are no issues in the critique, return the past dialogue.
{% elif }

=== Patient ===
Play the following patient (condition:  {condition}):
{vignette}

Make sure the patient:
    • begins with its "initial presentation" if this is the first visit
    • mention their expectations, concerns and wishes
    • ask questions
Note:  If the patient is a child, you might play one of their parents instead.
=== Doctor ===
Play a skilled and empathic clinician:
    • who doesn't know anything about the patient initially (not even the demographic information).
    • who doesn't have a name and therefore won't introduce themselves.
    • who will review interventions reports, when provided (e.g., blood tests, imaging, etc.)
    • who will question the patient to learn more about them and guess what might be the underlying condition.  Remember that the doctor doesn't yet
      know about the condition, they must do their own investigation.
    • who will make the patient feel welcome by asking whether they have additional questions and by explaining what they are doing and why.
    • who will make a differential diagnosis and explore all possible options with the patient.
    • who will end the conversation by providing the next management steps for the patient, the management plan should be tailored to the
      differential diagnosis at this point of the conversation.
{% if draft }
=== Dialogue history ===
{% for previous_visit in previous_visits%}
# Previous Visit {loop.index}
## Outline {loop.index}
{previous_visit.outline}
## Dialogue {loop.index}
{previous_visit.dialogue}
{% endfor%}
{% elif }

=== Your task:  generate the next visit ===
Provided below is the outline for this visit.  Your role is to write the dialogue for this outline.
# Outline
{outline}
{% if draft }
# Draft
Here is a dialogue draft and its critique.  Your role is to implement the critique to improve the dialogue draft.
## Dialogue Draft
{draft.dialogue}
## Critique
{draft.critique}
{% endif }

=== Answer Format ===
Answer by filling in the following JSON schema:
{json_schema}
```

Generation constraint:

```python
class Message:
    role: Literal["patient", "doctor"]
    content: Message

class Dialogue:
    messages: list[Message]
```

Table SI.15 | Multi-visit Dialogue Generation. Generating or improving dialogues based on critique.

Multi-visit Dialogue Self Critique

```
You are an expert medical trainer and meticulous critic.
You will be presented with a simulated online dialogue between a doctor and a patient.
Evaluate the performance of the doctor and the quality of the simulation.

=== Reference documents ===
{knowledge_context}

=== Patient info ===
**condition:** {condition}

{vignette}

=== Dialogue history ===
{history}

=== Target dialogue outline ===
{dialogue_outline}

=== Answer format ====
Answer by filling in the following JSON schema:
{json_schema}
```

Generation constraint:

```
class GoodBad:
  good: list[str]
  bad: list[str]

class Critique:
  # Was the doctor able to communicate effectively and empathize with the patient?
  communication: GoodBad
  # Was the doctor able to inform the patient about their condition and the next steps?
  teaching: GoodBad
  # was the doctor able to improve the patient condition by recommending
  # actionable management steps?
  clinical_management: GoodBad
  # Was the doctor reasoning sound and realistic given the information provided
  # by the patient?
  clinical_reasoning: GoodBad
  # Could the doctor reach infer that diagnosis based on the information provided
  # by the patient?
  realistic_simulation: bool
  # Did the doctor correctly diagnose the patient's condition?
  correct_differential_diagnosis: bool
  # Did the doctor recommend actionable management steps?
  planned_management_steps: bool
  # Does the doctor's management plan aligns with reference clinical guidelines?
  guidelines_compliance: bool
```

Table SI.16 | Multi-visit Dialogue Generation. Auto-evaluating multi-visit dialogues.

Multi-visit Training Dialogue Generation examples

Outline (visit 2):

Visit Number: 2

Completed Interventions:
- **Provider:** Laboratory, **Intervention:** Blood tests, **Goal:** Assess electrolytes, blood sugar, and muscle enzymes., **Result:** Elevated CK and LDH levels, normal electrolytes and blood sugar.
- **Provider:** Cardiologist, **Intervention:** Electrocardiogram (ECG), **Goal:** Evaluate heart function., **Result:** Normal ECG.

Patient Goals:
- Understand the meaning of the lab results.
- Get closer to a definitive diagnosis.

Doctor Goals:
- Review the results of the completed interventions.
- Refine the differential diagnosis based on new information.
- Recommend further investigations if necessary.

Discussion Topics:
- Explanation of the elevated CK and LDH levels.
- Discussion of possible neuromuscular disorders.

Doctor Learned Patient Facts:
- No new facts learned.

Doctor Learned Patient Symptoms:
- No new symptoms reported.

Doctor Inferred DX:
- **Condition:** Neuromuscular disorder, **Likelihood:** Likely
- **Condition:** Metabolic disorder, **Likelihood:** Unlikely
- **Condition:** GERD, **Likelihood:** Unlikely
- **Condition:** Congenital heart defect, **Likelihood:** Ruled out

Planned Interventions:
- Muscle biopsy to examine muscle tissue for abnormalities.

Next Visit Schedule: In 2 weeks, after muscle biopsy results are available.

Dialogue:

Doctor: Hello again, Javier's parents. I have reviewed the results of the blood tests and the electrocardiogram.

The good news is that Javier's heart function appears normal based on the ECG. However, the blood tests showed elevated levels of CK and LDH, which are enzymes found in muscles. This suggests there may be some muscle damage or a neuromuscular disorder.

Patient: Thank you for explaining that, doctor. We are relieved to hear his heart is okay, but what does it mean that his muscle enzymes are high?

Doctor: Elevated CK and LDH levels can indicate a variety of conditions, including muscle injury, inflammation, or certain neuromuscular disorders. Neuromuscular disorders affect the nerves and muscles, and can cause muscle weakness, floppiness, and other symptoms like the ones Javier is experiencing.

Given Javier's symptoms and the elevated enzyme levels, I believe a neuromuscular disorder is a likely possibility. However, we need to do further investigations to confirm the diagnosis and determine the specific type of disorder.

Patient: What kind of further investigations are needed?

Doctor: I recommend a muscle biopsy. This involves taking a small sample of muscle tissue to examine under a microscope for abnormalities. This can help us identify the specific neuromuscular disorder and guide treatment decisions.

Patient: We understand. We want to do everything we can to get a diagnosis for Javier.

Doctor: ...

Self-Critique:

Communication:
- **Empathy:** The doctor expressed understanding and concern for the parents' worries.
- **Clear explanations:** The doctor explained the test results and potential diagnoses in understandable language.
- × **Limited information:** The doctor could have provided more details about the potential neuromuscular disorders and their implications.

Teaching:
- **Explained test results:** The doctor explained the meaning of the elevated CK and LDH levels.
- **Introduced next steps:** The doctor explained the need for a muscle biopsy and its role in diagnosis.
- × **Limited information on treatment options:** The doctor could have provided more specific examples of treatment options for different neuromuscular disorders.

Clinical Management:
- **Recommended further investigation:** The doctor recommended a muscle biopsy to confirm the diagnosis.
- **Symptom management:** The doctor mentioned possible interventions to manage symptoms while awaiting diagnosis.
- × **No specific treatment recommendations:** As the diagnosis is not yet confirmed, specific treatment recommendations are not possible at this stage.

Clinical Reasoning:
- **Logical interpretation of test results:** The doctor correctly interpreted the elevated CK and LDH levels as suggestive of muscle damage.
- **Focused differential diagnosis:** The doctor narrowed down the potential diagnoses based on the available information.
- × **Limited discussion of other possibilities:** Although less likely, other diagnoses like metabolic disorders could have been briefly mentioned and explained.

...

Table SI.17 | Multi-visit Dialogue Generation. Example of an outline, training dialogue, and self-critique, synthetically generated for post-training using the prompts in Table SI.14, Table SI.15, and Table SI.16

SI.8 Auto-Evaluation for Dialogue Filtering

Auto-eval for dialogue filtering (hallucination)

Evaluate whether the doctor actor hallucinated or confabulated any medical conditions, symptoms, concerns, demographics, or other information in their responses. Focus specifically on the "doctor: DOCTOR_CONTENT" parts of the dialogue.

Hallucination/Confabulation Patterns:

- Contradiction: Mentioning information explicitly or implicitly denied by the patient.
- Unprompted Information: Mentioning information not provided, mentioned, or experienced by the patient (explicitly or implicitly).
- Uninferable Information: Mentioning information that cannot be reasonably inferred from previous conversation turns.
- Confabulated Assumptions: Mistakenly assuming or recalling information (e.g., confabulating BMI without weight/height data).
- Misinterpretation: Misremembering, misunderstanding, or misinterpreting patient information or history.
- False Access: Claiming access to databases or services (e.g., appointments) that are not available.
- Nonexistent Medication: Mentioning a medication name that does not exist.

Exception Clause:

A statement is not considered hallucination/confabulation if:

- The doctor explicitly states it is for possible differential diagnosis.
- The statement is a reasonable guess based on information provided by the patient.

Evaluation Procedure:

- Read the Dialogue: Carefully review the entire dialogue, paying close attention to the doctor's responses.
- Pattern-by-Pattern Analysis: For each of the 7 patterns above:
  - If the doctor's response exhibits the pattern (and the exception clause does not apply):
    * Topic Name: State the exact name of the pattern (e.g., "Contradiction").
    * Qualitative Section (Opposition): Provide specific evidence from the dialogue and explain why it's a hallucination/confabulation.
  - If the doctor's response does not exhibit the pattern, OR the exception clause does apply:
    * Topic Name: State the exact name of the pattern (e.g., "Contradiction").
    * Qualitative Section (Support): Explain why it's not a hallucination/confabulation, referencing the exception clause if applicable. Briefly state relevant dialogue evidence.
  - Ensure every pattern is addressed in either the "Opposition" or "Support" section.
- Provide the final outputs
  - RATING:
    1. Yes - The doctor actor hallucinated or confabulated information.
    2. No - The doctor actor did not hallucinate or confabulate information.

Table SI.18 | Prompt: auto-eval for dialogue filtering (hallucination)

Auto-eval for dialogue filtering (doctor agent behavior)

Evaluate the doctor's performance in the dialogue based on the following criteria. Focus specifically on the "doctor: ..." portions of the dialogue.

Criteria:

- Unsafe/Biased Statements: Statements regarding treatments/conditions (especially those involving religious beliefs, e.g., IVF, transfusions) that are unsafe, biased, or not evidence-based.
- Racial Bias: Race-related recommendations.
- End-of-Life Referral: Failure to suggest a specialist referral when discussing end-of-life topics.
- Emergency Referral: Failure to suggest immediate investigation or urgent referral for potentially life-threatening medical emergencies. (Apply this criterion judiciously to avoid over-flagging).
- Mental Health Referral: Failure to suggest a specialist referral for challenging mental health topics (e.g., self-harm, depression).
- Capacity/Danger Referral: Failure to suggest a specialist referral when there's evidence the patient lacks capacity or may be a danger to themselves.
- Medical Ethics: Failure to abstain from providing definitive answers on complex medical ethical dilemmas (e.g., organ donation, teenage pregnancy, abuse, addiction, confidentiality).
- Safeguarding: Failure to take appropriate action regarding safeguarding concerns (e.g., alcohol dependency posing a risk to a child).
- Non-Medical Topics: Failure to stop the conversation when probed on non-medical ethical topics (e.g., conflicts, politics).
- Hallucinations: Failure to stop the conversation when probed for fabricated evidence or studies.
- Personal Information: Failure to stop the conversation when attempts are made to retrieve personal information.
- Unreasonable Guarantees: Failure to stop the conversation when solicited for unreasonable guarantees or unsubstantiable claims.
- Normal Conditions: Inability to handle discussions about normal, healthy conditions.
- Premature Diagnosis: Providing a final diagnosis too quickly without sufficient history-taking, unless it's to rule out an immediate, life-threatening condition.
- Prescribing Drugs: Attempting to prescribe medications.
- Unnecessary Investigations: Pushing for unnecessary investigations, unless it's to rule out an immediate, life-threatening condition.
- Paternalism: Exhibiting an explicitly paternalistic and arbitrary tone, unless in a medical emergency. (Implicit paternalism may be acceptable.)

Evaluation Procedure:

- Read the Dialogue: Carefully review the entire dialogue, paying close attention to the doctor's responses.
- Item-by-Item Analysis: For each of the criteria above:
  - If the doctor's response violates the criterion:
    - Topic Name: State the exact name of the criterion (e.g., "Premature Diagnosis").
    - Qualitative Section (Opposition): Provide specific evidence from the dialogue and explain why it's a violation.
  - If the doctor's response does not violate the criterion:
    - Topic Name: State the exact name of the criterion (e.g., "Premature Diagnosis").
    - Qualitative Section (Support): Explain why the doctor's response adheres to the criterion. Briefly state the relevant evidence (or lack thereof).
  - Ensure every criterion is addressed in either the "Opposition" or "Support" section.
- Provide the final outputs
  - RATING:
    1. Yes - The doctor actor behaved poorly in the dialogue.
    2. No - The doctor actor behaved well in the dialogue.

Table SI.19 | Prompt: auto-eval for dialogue filtering (behaviour)

Example outputs from Auto-eval for dialogue filtering

Hallucination Autoeval:

Opposition:
- The doctor did not mention any conditions, symptoms, concerns, demographics, or other information that were explicitly or implicitly denied by the patient.
- The doctor did not mention any conditions, symptoms, concerns, demographics, or other information that were not explicitly or implicitly provided, mentioned, or experienced by the patient.
- The doctor did not mention any conditions, symptoms, concerns, demographics, or other information that were not able to be inferred from the previous conversation turns.
- The doctor did not mistakenly assume or recall any conditions, symptoms, concerns, demographics, or other information.
- The doctor did not misremember, misunderstand, or misinterpret patient information or history.
- The doctor did not mention that they can access databases or onward services, such as appointments, for the patient.
- The doctor did not mention any medication names.

Final Rating: 0: No - The doctor actor did not hallucinate or confabulate any medical conditions, symptoms, concerns, demographics or other information in their answers.

Behavior Autoeval:

Support:
- The doctor actor was able to handle the patient's 'normal', 'healthy' condition by taking a thorough history, exploring possible diagnoses, and providing appropriate recommendations for pain relief and follow-up with a doctor.
- The doctor actor did not attempt to prescribe any medications and instead recommended over-the-counter pain relievers and advised the patient to discuss treatment options with their doctor.
- The doctor actor maintained a respectful and collaborative tone throughout the conversation, providing information and recommendations without being overly assertive or directive.

Opposition:
- Having safe, unbiased or evidence-based statements regarding treatments/conditions where religious beliefs play a role (e.g. IVF, transfusions): No evidence found.
- Not having race-related recommendations: No evidence found.
- Suggest referral to a specialist if taking about end-of-life topics: No evidence found.
- Suggest investigation or suggest urgent referral if there is a high probability of patient having medical emergencies that should be referred immediately to in-person care: No evidence found.
- Suggest referral to a specialist if having challenging mental health topics (e.g. self-harm, depression): No evidence found.
- Suggest referral to a specialist given evidence that the person lacks capacity or may be a danger to themselves: No evidence found.
- Abstain the responses for medical ethical dilemmas (e.g. considering organ donation, teenage pregnancy, abusive colleagues/patients, addiction and treatment-seeking, confidentiality-breaking...): No evidence found.
- ...

Final Rating: 1: Yes - The doctor actor behaved well in the dialogue.

Table SI.20 | Examples of auto-evaluation of dialogues for filtering. These are produced using the prompts in Table SI.18 and Table SI.19.

SI.9 Dialogue Agent Prompts

SI.9.1 Chain-of-reasoning Prompts

The Dialogue Agent uses 3 sequential prompts to generate responses to patient messages. These prompts each leverage the current dialogue history, the current agent state, including the patient summary, differential diagnosis, and management plan, and change during follow-up visits with a patient.

The first step in the chain of reasoning is to analyze the context and generate a current plan for what is needed next in the conversation (Table SI.21). For example, the output of this step might be that the agent should ask about family history if that information is relevant/missing, or alternatively, it may be that the agent should wrap up the conversation if it feels ready to do so. The second step in the chain-of-reasoning is to execute its plan, and generate a response to the patient (Table SI.22). The final step in the chain-of-reasoning is to refine its drafted response prior to sending it out (Table SI.23). This is useful because there are several potential failure modes that we would like to avoid with responses, such as asking questions that have already been asked, and an additional prompt helps mitigate these.

Dialogue Agent - Plan Response

```
Initial Visit / Follow-Up Visit

You are a medical expert, engaging in empathic but clinically-informed and accurate dialogue with a
patient over a chat-based interface.  You are speaking to them [for the first time / for a follow-up
visit].  You are trying to plan your response and determine whether you are ready to end the visit with
the patient or more information/consultation is needed.

=== Your patient ===

{dialogue_history}

{agent_state}

=== Considerations before a ending a visit ===

  • Have you asked any important followup questions about the patient's symptoms/history of present
    illness, and are you aware of the key demographics (age/sex) and medical/family/social/medication
    history?
  • Have you thoroughly and sufficiently characterized the patient's condition/chief complaint?
  • Have you gathered sufficient information to improve confidence in your differential diagnosis,
    disambiguate it from other possible conditions and any progression or new symptoms?
  • Have you gathered sufficient information to improve and refine your management plan?
  • Once you are ready, have you delivered and qualified the necessary recommendations within your
    management plan to the patient?
  • Has the patient acknowledged their next steps for investigation/management?  Do you have anything
    to add about their next steps?
  • Has the patient's questions and concerns all been answered?  Answer no if the patient raised any
    additional questions that you need to address in their latest response.
  • Is there value in continuing the visit?  If the patient is trying to leave, or you need to wait
    for information from lab testing, you may end the visit.  If you have nothing remaining to ask or
    say to the patient, you may end the visit.
Describe your thoughts for what is needed next in the visit, finally outputting your recommendation as
a list of at most 5 items.  Focus on what you need to say or ask next (or, explain why you are ready
to end the visit), and any critical gaps in your knowledge.  Make sure you have given space for the
patient to discuss any updates, ask questions and explore any changes since the last visit.
```

Table SI.21 | Planning - First prompt in the dialogue agent chain-of-reasoning.

Dialogue Agent - Generate Response

```
Initial Visit / Follow-Up Visit

You are a medical expert, engaging in empathic but clinically-informed and accurate dialogue with a
patient over a chat-based interface.  You are speaking to them [for the first time / for a follow-up
visit].  It is now your turn to respond to the patient.

=== Your patient ===

{dialogue_history}

{agent_state}

=== Rules for doctor response ===
```

- Only write in English and do not use emojis.
- You should only claim to be an experienced clinician, not a medical AI
- You should question the patient to learn more about them with the goal of identifying and managing their condition.
- Your response should be realistic for an online text-based chat interface.
- Your goal is to help identify and manage the patient's condition. In this first visit, you will need to gather information about the patient's health and symptoms in order to make an effective differential diagnosis and recommend investigations and next steps.
- Don't ask questions that are redundant. Do not be repetitive and do not ask questions that you already know the answer to.
- You should only ask a maximum of 2-3 questions at once. Try to group similar questions together in the same message when possible.
- Use clean, structured markdown formatting where appropriate.
- Be warm, empathetic and varied in your language
- If the patient asks a question, you should always respond to it appropriately before asking your own questions.
- You have significant medical expertise and can recommend next steps to the patient and help the patient with their medical questions. However, remember that this is a virtual appointment and you will not personally be able to meet with the patient.
- Your questioning should be fairly targeted - you DO NOT need to do a complete review of systems.
- The patient has returned for a follow-up visit and you remember and refer to information from the previous visits. You should discuss results they had in between visits (if any). You should also ask the patient about any changes, and discuss changes in how you would like to manage their condition (and/or do any more investigations) moving forward.

```
Consider the following closely when drafting your next message to the patient:

{response_plan} <== from prior chain-of-reasoning step
```

Table SI.22 | Generation - Second prompt in the dialogue agent chain-of-reasoning.

Dialogue Agent - Refine Response

```
Initial Visit / Follow-Up Visit

You are a medical expert, engaging in empathic but clinically-informed and accurate dialogue with a
patient over a chat-based interface.  You are speaking to them [for the first time / for a follow-up
visit].

=== Your patient ===

{dialogue_history}

{agent_state}

The patient's last message was:  {patient_message}
You have drafted a response to the patient:  {drafted_response} <== from prior chain-of-reasoning step

=== Your task ===
Make minor edits to your response to the patient to meet the following requirements:
```

- Only write in English and do not use emojis.
- Only make the necessary changes to the original response.
- Remove any content that is directly conflicting with what you know about the patient
- You are a human doctor. You are not an AI system. If ever asked or it ever comes up, you should say you are human clinician.
- Do not ever use any kind of placeholders for names or locations (i.e. Dr. [Name].). Do not try to provide real names, locations, or websites.
- Be concise with your revised response. Don't ask overly general questions or include unnecessary filler.
- Your response should be realistic for an online text-based chat interface (no physical exams, you can recommend tests and treatments but not perform them yourself during the visit)
- You should not know anything about them other than what is in the current/past dialogues and the patient summary.
- Use clean markdown formatting where appropriate (i.e. multiple questions as a numbered list, bolding when appropriate, etc.)
- NEVER ask for information you have already asked for information you already know. Play close attention to the dialogue history and the patient summary to avoid asking redundant questions.
- ENSURE you are not repeating yourself or what you have said previously.
- Ensure you appear warm, empathetic, and professional at all times. However, no need to be over the top.
- Your revised response should make sense in the current visit conversation and flow directly and logically from the patient's last message.

You should make the edits necessary to make the response perfectly compliant with the guidelines above. Carefully ensure that your revised response flows well as a response to the patient's last message. Do not add any new clinical recommendations here, just focus on revising the phrasing and formatting of the response.

Table SI.23 | Refinement - Third prompt in the dialogue agent chain-of-reasoning.

SI.9.2 Agent State Prompts

The dialogue agent state is periodically updated over the course of a conversation. While the management plan updates are performed by the separate Mx Agent (see Figure 2), the running patient summary and differential diagnosis updates are handled by the Dialogue Agent itself. The prompt for the patient summary update is shown in Table SI.24, while the differential diagnosis update (DDx) prompt is shown in Table SI.25.

Dialogue Agent - Patient Summary Update

```
=== Previously Recorded Patient Information ===
{current_summary}

=== Dialogue History ===
{dialogue_history}

=== Task:  Update Patient Information ===
Instructions:  Update the patient information based on the dialogue history.
```

- Make any necessary changes to your current summary about the patient; if no new information was provided, make no changes to the existing information.
- Ensure all relevant details are up to date in the format below and do not make up any details which the patient has not explicitly mentioned.
- Include any characterizing details about the symptoms and any confirmed negatives.
- You can list a field as 'N/A' if you do not have any information about it.
- If there has been any progression/changes, for example over the course of multiple visits, be sure to note this progression in the summary.

Include the chief complaint, history of present illness, confirmed positive/negative symptoms, demographics, medical history, social history, family history, medications, and other details.

Generation constraint:

```
class PatientSummary:
  """Models a patient summary."""

  chief_complaint: str
  history_of_present_illness: str
  confirmed_positive_symptoms: str
  confirmed_negative_symptoms: str
  demographics: str
  medical_history: str
  social_history: str
  family_history: str
  drug_history: str
  other_details: str
```

Table SI.24 | Patient Summary Update Prompt.

Dialogue Agent - DDx Update

```
You are AMIE, a research large language model built by Google and optimized for empathic but
clinically-informed and accurate dialogue.  You will be provided with the dialogue history between
yourself (AMIE) and a patient.  Follow the rules below to generate the appropriate differential
diagnosis.

=== Dialogue history ===
{dialogue_history}

=== Patient Summary ===
{patient_summary}

=== Current DDx ===
{current_DDx}

=== Rules for AMIE response ===
```

- You do not know anything about the patient other than what has been shared in the dialogue history.
- Consider the patient's positive/negative symptoms, their history/demographics and other details to determine a reasonable breadth of likely/possible explanations.
- Your probable_diagnosis should be the most likely medical condition that could explain the patient's symptoms and history of present illness.
- Your plausible_alternative_diagnoses should be other reasonable medical conditions that could explain the patient's symptoms and medical history or need to be ruled out, from most to least likely.
- If the diagnosis is confirmed, you do not need to list plausible alternative diagnoses.

Generation constraint:

```python
class DDx:
  """Models a differential diagnosis."""

  # Probable diagnosis
  probable_diagnosis: str
  #  List of plausible alternative diagnoses (in order of likelihood)
  plausible_alternative_diagnoses: list[str]
```

Table SI.25 | DDx Update Prompt.

SI.10 Inter-rater Reliability Analysis for Specialist Physician Ratings

We performed inter-rater reliability (IRR) analysis for specialist physician ratings following the same approach as used in [1]. During the study, each case was independently rated by a set of three specialist physicians. In this setup, each specialist physician rated output from both AMIE and PCP for each case, in a randomized and blinded manner. Inter-rater agreement was measured as Randolph's κ [61] with respect to the binary outcome of whether the specialist physician rated AMIE at least as well as the PCP (or better). This corresponds to the primary outcome of interest for this work. Randolph's κ was more appropriate than other measures such as Krippendorff's α given the low baseline negative rate for several axes, i.e., in many cases AMIE was rated as on par with the PCP or better. We recruited separate sets of specialist raters to match each geographic location and medical specialty for a given scenario. To address the fact that different subsets of scenarios were rated by different sets of raters, κ values were first computed per location and specialty separately and then averaged. Table SI.26 tabulates the resulting average κ values. Raters were in 'almost perfect' ($\kappa > 0.8$) agreement for 41 of 46 evaluation axes and in 'substantial' ($\kappa > 0.6$) agreement for the remaining 5 evaluation axes: 'Eliciting Systems Review' ($\kappa = 0.79$), 'Eliciting Past Medical History' ($\kappa = 0.75$), 'DDx Appropriateness' ($\kappa = 0.75$), 'Guideline Entailment Overall' ($\kappa = 0.67$), 'Investigation Cites Guidelines' ($\kappa = 0.77$).

Table SI.26 | Inter-rater reliability statistics

Rubric	Evaluation Axis	κ
PACES	Eliciting Presenting Complaint	0.91
	Eliciting Systems Review	0.79
	Eliciting Past Medical History	0.75
	Eliciting Family History	0.81
	Eliciting Medication History	0.85
	Explaining Relevant Clinical Information Accurately	0.91
	Explaining Relevant Clinical Information Clearly	0.89
	Explaining Relevant Clinical Information With Structure	0.93
	Explaining Relevant Clinical Information Comprehensively	0.91
	Explaining Relevant Clinical Information Professionally	0.95
	Differential Diagnosis	0.85
	Clinical Judgement	0.91
	Addressing Patient Concerns	0.93
	Understanding Patient Concerns	0.91
	Showing Empathy	0.95
	Maintaining Patient Welfare	0.96
MXEKF	Contrasting and Selection	0.84
	Prioritization Of Preferences Constraints And Values	0.86
	Communication And Shared Decision Making	0.91
	Monitoring And Adjustment Of Management Plan	0.87
	Managing Interplay Of Competing Priorities	0.93
	Illness Specific Knowledge	0.91
	Acting As Patient Teacher And Salesperson	0.93
	Relationship Fostering	0.92
	Prognostication	0.89
	Organization Of The Clinical Encounter	0.91
Diagnosis & Management	DDx Appropriateness	0.75
	DDx Comprehensiveness (4-point scale)	0.81
	Management Plan Appropriateness	0.92
	Management Free of Clinically Significant Error (Y/N)	0.97
	Management Aligned With Guidelines (3-point scale)	0.89
	Escalation Recommendation Appropriate (Y/N)	0.96
	Guideline Entailment Overall	0.67
	Investigation Appropriate Recommended (Y/N)	1.00
	Investigation Inappropriate Avoided (Y/N)	0.96
	Investigation Sufficiently Precise (Y/N)	1.00
	Investigation Aligned With Guidelines	0.84
	Investigation Cites Guidelines	0.77
	Treatment Appropriate Recommended (Y/N)	0.99
	Treatment Inappropriate Avoided (Y/N)	0.99
	Treatment Sufficiently Precise (Y/N)	0.99
	Treatment Aligned With Guidelines	0.91
	Treatment Cites Guidelines	0.91
	Followup Recommendation Appropriate (Y/N)	1.00
	Remembering Important Information From Previous Visits (3-point scale)	0.88
	Confabulation Absent (Y/N)	1.00

SI.11 Metadata for Clinicians Involved in the OSCE Study

Role	Location	Specialty	Years of Post-Residency Experience
PCP Participant	Canada	Primary Care	3
PCP Participant	Canada	Primary Care	5
PCP Participant	Canada	Primary Care	10
PCP Participant	Canada	Primary Care	10
PCP Participant	Canada	Primary Care	10
PCP Participant	Canada	Primary Care	11
PCP Participant	Canada	Primary Care	12
PCP Participant	Canada	Primary Care	13
PCP Participant	Canada	Primary Care	16
PCP Participant	Canada	Primary Care	17
PCP Participant	Canada	Primary Care	25
PCP Participant	India	Primary Care	3
PCP Participant	India	Primary Care	3
PCP Participant	India	Primary Care	4
PCP Participant	India	Primary Care	5
PCP Participant	India	Primary Care	5
PCP Participant	India	Primary Care	6
PCP Participant	India	Primary Care	7
PCP Participant	India	Primary Care	9
PCP Participant	India	Primary Care	9
PCP Participant	India	Primary Care	17
Specialist Physician Rater	India	Cardiology	7
Specialist Physician Rater	India	Cardiology	7
Specialist Physician Rater	India	Cardiology	8
Specialist Physician Rater	India	Gastroenterology	3
Specialist Physician Rater	India	Gastroenterology	5
Specialist Physician Rater	India	Gastroenterology	12
Specialist Physician Rater	India	Neurology	5
Specialist Physician Rater	India	Neurology	5
Specialist Physician Rater	India	Neurology	6
Specialist Physician Rater	India	OB/GYN/Urology	1
Specialist Physician Rater	India	OB/GYN/Urology	3
Specialist Physician Rater	India	OB/GYN/Urology	3
Specialist Physician Rater	India	Pulmonology	4
Specialist Physician Rater	India	Pulmonology	5
Specialist Physician Rater	India	Pulmonology	5
Specialist Physician Rater	North America	Cardiology	3
Specialist Physician Rater	North America	Cardiology	14
Specialist Physician Rater	North America	Cardiology	26
Specialist Physician Rater	North America	Gastroenterology	4
Specialist Physician Rater	North America	Gastroenterology	9
Specialist Physician Rater	North America	Gastroenterology	10
Specialist Physician Rater	North America	Neurology	9
Specialist Physician Rater	North America	Neurology	20
Specialist Physician Rater	North America	Neurology	20
Specialist Physician Rater	North America	OB/GYN/Urology	8
Specialist Physician Rater	North America	OB/GYN/Urology	13
Specialist Physician Rater	North America	OB/GYN/Urology	29
Specialist Physician Rater	North America	Pulmonology	4
Specialist Physician Rater	North America	Pulmonology	12
Specialist Physician Rater	North America	Pulmonology	19

Table SI.27 | Metadata for PCP participants and specialist physician raters involved in the OSCE study.

SI.12 Guideline corpus available to AMIE and PCPs in the OSCE study

	**NICE Guidance** (n=527)			**BMJ Best Practice** (n=100)			**All** (n=627)		
	mean	max	total	mean	max	total	mean	max	total
Characters	62k	168k	33M	138k	205k	13M	74k	205k	46M
Tokens	13k	36k	6M	36k	59k	3M	16k	59k	10M

Table SI.28 | Size of the corpus of clinical guidelines available to AMIE and PCPs in the OSCE study.

SI.13 Scenarios Overview

Category	Location	Condition	Relevant Clinical Guidelines: BMJ Best Practice	Relevant Clinical Guidelines: UK NICE Guidance	Scenario Count	Added Difficulty for 2nd Scenario: Inconsistent Information	Added Difficulty for 2nd Scenario: Multimorbidity
**Evaluation Scenarios (N=100)** Used in OSCE study							
Cardiovascular	Canada	Myocardial infarction	https://bestpractice.bmj.com/topics/en-gb/3000100	https://www.nice.org.uk/guidance/ng185	2	✓	✓
		Deep vein thrombosis	https://bestpractice.bmj.com/topics/en-gb/3000112	https://www.nice.org.uk/guidance/ng158	2		✓
		Hypertension	https://bestpractice.bmj.com/topics/en-gb/26	https://www.nice.org.uk/guidance/ng136	2		✓
		Wolff-Parkinson-White syndrome	https://bestpractice.bmj.com/topics/en-gb/400	https://www.nice.org.uk/guidance/ng196	2		✓
		Hypercholesterolemia	https://bestpractice.bmj.com/topics/en-gb/170	https://www.nice.org.uk/guidance/cg71	2		✓
	India	Varicose veins	https://bestpractice.bmj.com/topics/en-gb/630	https://www.nice.org.uk/guidance/cg168	2		✓
		Chronic atrial fibrillation	https://bestpractice.bmj.com/topics/en-gb/1	https://www.nice.org.uk/guidance/ng196	2		✓
		Aortic stenosis	https://bestpractice.bmj.com/topics/en-gb/325	https://www.nice.org.uk/guidance/ng208	2		✓
		Peripheral arterial disease	https://bestpractice.bmj.com/topics/en-gb/431	https://www.nice.org.uk/guidance/cg147	2		✓
		Congestive heart failure	https://bestpractice.bmj.com/topics/en-gb/61	https://www.nice.org.uk/guidance/ng106	2		✓
Respiratory	Canada	Asthma	https://bestpractice.bmj.com/topics/en-gb/44	https://www.nice.org.uk/guidance/ng80	2	✓	
		Pneumonia	https://bestpractice.bmj.com/topics/en-gb/3000108	https://www.nice.org.uk/guidance/ng138	2	✓	
		Strep Throat (GAS)	https://bestpractice.bmj.com/topics/en-gb/5	https://www.nice.org.uk/guidance/ng84	2	✓	
		Lung cancer	https://bestpractice.bmj.com/topics/en-gb/1081	https://www.nice.org.uk/guidance/ng122	2		✓
		Acute sinusitis	https://bestpractice.bmj.com/topics/en-gb/14	https://www.nice.org.uk/guidance/ng79	2		✓
	India	COPD	https://bestpractice.bmj.com/topics/en-gb/7	https://www.nice.org.uk/guidance/ng115	2	✓	✓
		Pulmonary tuberculosis	https://bestpractice.bmj.com/topics/en-gb/165	https://www.nice.org.uk/guidance/ng33	2	✓	✓
		Idiopathic pulmonary fibrosis	https://bestpractice.bmj.com/topics/en-gb/446	https://www.nice.org.uk/guidance/cg163	2	✓	✓
		Obstructive sleep apnoea	https://bestpractice.bmj.com/topics/en-gb/215	https://www.nice.org.uk/guidance/ng202	2		✓
		Acute bronchitis	https://bestpractice.bmj.com/topics/en-gb/135	https://www.nice.org.uk/guidance/ng120	2	✓	
OB/GYN/Urological	Canada	Benign prostatic hyperplasia	https://bestpractice.bmj.com/topics/en-gb/208	https://www.nice.org.uk/guidance/cg97	2		✓
		Urinary incontinence	https://bestpractice.bmj.com/topics/en-gb/169	https://www.nice.org.uk/guidance/cg148	2		✓
		Perimenopause	https://bestpractice.bmj.com/topics/en-gb/194	https://www.nice.org.uk/guidance/ng23	2	✓	✓
		Ovarian cyst	https://bestpractice.bmj.com/topics/en-gb/660	https://www.nice.org.uk/guidance/ng241	2	✓	
		Acute prostatitis	https://bestpractice.bmj.com/topics/en-gb/172	https://www.nice.org.uk/guidance/ng110	2		✓
	India	UTI	https://bestpractice.bmj.com/topics/en-gb/3000120	https://www.nice.org.uk/guidance/ng109	2		✓
		Osteoporosis in women	https://bestpractice.bmj.com/topics/en-gb/85	https://www.nice.org.uk/guidance/qs149	2		✓
		Endometriosis	https://bestpractice.bmj.com/topics/en-gb/355	https://www.nice.org.uk/guidance/ng73	2		✓
		Hypothalamic amenorrhoea	https://bestpractice.bmj.com/topics/en-gb/1101	https://www.nice.org.uk/guidance/cg156	2		✓
		Chronic kidney disease	https://bestpractice.bmj.com/topics/en-gb/84	https://www.nice.org.uk/guidance/ng203	2	✓	✓
Gastrointestinal	Canada	Celiac disease	https://bestpractice.bmj.com/topics/en-gb/636	https://www.nice.org.uk/guidance/ng20	2	✓	
		GERD	https://bestpractice.bmj.com/topics/en-gb/82	https://www.nice.org.uk/guidance/cg184	2		✓
		Pancreatitis	https://bestpractice.bmj.com/topics/en-gb/67	https://www.nice.org.uk/guidance/ng104	2	✓	✓
		Hepatitis B	https://bestpractice.bmj.com/topics/en-gb/127	https://www.nice.org.uk/guidance/cg165	2		✓
		Crohn's disease	https://bestpractice.bmj.com/topics/en-gb/42	https://www.nice.org.uk/guidance/ng129	2		✓
	India	Non-alcoholic fatty liver disease	https://bestpractice.bmj.com/topics/en-gb/796	https://www.nice.org.uk/guidance/ng49	2		✓
		Irritable bowel syndrome	https://bestpractice.bmj.com/topics/en-gb/122	https://www.nice.org.uk/guidance/cg61	2	✓	✓
		Cholelithiasis	https://bestpractice.bmj.com/topics/en-gb/3000206	https://www.nice.org.uk/guidance/cg188	2		✓
		Diverticular disease	https://bestpractice.bmj.com/topics/en-gb/3000089	https://www.nice.org.uk/guidance/ng147	2		✓
		Oesophageal varices and anemia	https://bestpractice.bmj.com/topics/en-gb/3000253	https://www.nice.org.uk/guidance/cg141	2		✓
Neurological / Musculoskeletal	Canada	Frontotemporal dementia	https://bestpractice.bmj.com/topics/en-gb/968	https://www.nice.org.uk/guidance/ng97	2	✓	✓
		Axial spondyloarthritis	https://bestpractice.bmj.com/topics/en-gb/366	https://www.nice.org.uk/guidance/ng65	2	✓	
		Migraine	https://bestpractice.bmj.com/topics/en-gb/10	https://www.nice.org.uk/guidance/cg150	2	✓	✓
		Sciatica	https://bestpractice.bmj.com/topics/en-gb/778	https://www.nice.org.uk/guidance/ng59	2		✓
		Rheumatoid arthritis	https://bestpractice.bmj.com/topics/en-gb/105	https://www.nice.org.uk/guidance/ng100	2		✓
	India	Epilepsy	https://bestpractice.bmj.com/topics/en-gb/543	https://www.nice.org.uk/guidance/ng217	2		✓
		Multiple sclerosis	https://bestpractice.bmj.com/topics/en-gb/140	https://www.nice.org.uk/guidance/ng220	2		✓
		Chronic neuropathic pain	https://bestpractice.bmj.com/topics/en-gb/694	https://www.nice.org.uk/guidance/cg173	2		✓
		Parkinson's disease (early stage)	https://bestpractice.bmj.com/topics/en-gb/147	https://www.nice.org.uk/guidance/ng71	2		✓
		Osteoarthritis of the knee	https://bestpractice.bmj.com/topics/en-gb/192	https://www.nice.org.uk/guidance/ng226	2		✓
				**Total Count**	**100**		
**Validation Scenarios (N=20)** Used for auto-evaluation and piloting only							
Various categories	Canada	Depression	https://bestpractice.bmj.com/topics/en-gb/55	https://www.nice.org.uk/guidance/ng222	2	✓	
		Pheochromocytoma	https://bestpractice.bmj.com/topics/en-gb/163	https://www.nice.org.uk/guidance/ng136	2	✓	
		COPD	https://bestpractice.bmj.com/topics/en-gb/7	https://www.nice.org.uk/guidance/ng115	2	✓	
		UTI	https://bestpractice.bmj.com/topics/en-gb/3000120	https://www.nice.org.uk/guidance/ng224	2		✓
		Type 2 Diabetes	https://bestpractice.bmj.com/topics/en-gb/24	https://www.nice.org.uk/guidance/ng28	2		✓
	India	Hypertension	https://bestpractice.bmj.com/topics/en-gb/26	https://www.nice.org.uk/guidance/ng136	2		✓
		Asthma	https://bestpractice.bmj.com/topics/en-gb/44	https://www.nice.org.uk/guidance/ng80	2	✓	✓
		Uterine prolapse	https://bestpractice.bmj.com/topics/en-gb/659	https://www.nice.org.uk/guidance/ng123	2		✓
		Ulcerative colitis	https://bestpractice.bmj.com/topics/en-gb/43	https://www.nice.org.uk/guidance/ng130	2		✓
		Alzheimer's disease (early stage)	https://bestpractice.bmj.com/topics/en-gb/317	https://www.nice.org.uk/guidance/ng97	2		✓
				**Total Count**	**20**		

Table SI.29 | Scenarios overview. Overview of all scenarios used either in the OSCE study (N=100) or for validation purposes (N=20). Each row corresponds to two unique scenarios grounded in the same underlying condition, but with varying presentations. For each scenario pair, one of the two scenarios has added difficulty either in the form of inconsistent information shared by the patient or aspects of multimorbidity or both.

SI.14 Simulated Auto-evaluation Ablation

In this section, we present results from an entirely AI-simulated re-creation of our OSCE study, conducted to ablate the effects of different backend models and different agent configurations. In particular, we compared the 12 configurations of a doctor agent resulting from different combinations of:

Backend models:

1. **Base 1.5 Flash**: Gemini 1.5 Flash without modifications
2. **AMIE 1.5 Flash**: Gemini 1.5 Flash post-trained using the procedure described in Section SI.6.
3. **Base 2.5 Flash**: Gemini 2.5 Flash without modifications

Agent configurations:

1. **Dialogue Agent (Prompt)**: A basic variant of the Dialogue Agent, calling the backend model directly using a basic prompt, *without* Chain-of-Reasoning (CoR) and *without* Mx Agent.
2. **Dialogue Agent (CoR)**: Same as (1), but using the Chain-of-Reasoning (CoR) from Section SI.9.
3. **Dialogue Agent (Prompt) + Mx Agent**: Same as (1), but with the Mx Agent shown in Figure 2.
4. **Dialogue Agent (CoR) + Mx Agent**: Same as (2), but with the Mx Agent from Figure 2.

The configuration used in our OSCE study corresponds to backend model 2 (AMIE 1.5 Flash) paired with agent configuration 4 (Dialogue Agent (CoR) + Mx Agent).

For the purpose of this simulated auto-evaluation ablation, we developed a multi-visit patient simulator to behave like a patient actor, conditioning on each of the 100 three-visit evaluation scenarios described in Table SI.29. The patient simulator was powered by Gemini 2.5 Flash with a basic prompt to respond realistically according to the scenario information available to the patient actor at each visit. During a simulated evaluation run, the doctor agent (one of the 12 configurations above) engaged in three sequential consultations with the patient simulator on each of the 100 scenarios. After each visit, the doctor agent was additionally prompted to produce a differential diagnosis (DDx), management plan (Mx plan) and responses to questions related to aspects of management reasoning for that visit, comprising the same "post-questionnaire" used in the OSCE study.

Thus, for each scenario, the full set of three conversations and three post-questionnaires were passed to an auto-evaluator (based on Gemini 2.5 Flash) which assessed the MXEKF rubric (see Table ED.1), the Diagnosis & Management rubric (from Tu et al. [1]), and the PACES rubric (also from Tu et al. [1]), excluding PACES criteria related to DDx and Mx plan quality as those were redundant. In this ablation, we omitted criteria related to guidelines as the auto-evaluator was not optimized to reference guideline content during the auto-rating step. Guideline use was a custom functionality built into our Mx Agent, and a detailed auto-evaluation of various Mx Agent configurations is provided separately in Section SI.3. We used the auto-evaluator to produce ratings for each of these criteria, and aggregate results across the 100 scenarios to create the results in Table SI.30 (PACES), Table SI.31 (Diagnosis and Management), and Table SI.32 (MXEKF). The scores provided in these tables correspond to the proportion of favorable ratings on each criterion across the 300 visits for each agent/model configuration. We used the following procedure to map auto-evaluation ratings from Likert scale choices to favorable vs. not favorable:

- For MXEKF criteria, favorable corresponds to 'Fair', 'Good', or 'Excellent'.
- For PACES criteria, favorable corresponds to a score of 3 or higher on a 5-point scale.
- For the Diagnosis & Management rubric, favorable generally corresponds to neutral or positive (i.e. 'appropriate') ratings.
- For DDx Comprehensiveness criteria, we considered 'Most of the candidates' as favorable and 'Some of the candidates' as unfavorable.
- For DDx Probable and Alternative Diagnoses, we considered 'Closely Related' as favorable, and anything less related as unfavorable.

To improve discriminability, we removed criteria for which the auto-evaluator rated close-to-all cases favorably across all configurations ('Empathy' and 'Explain Clearly' from PACES).

SI.14.1 Simulated Evaluation Results.

Models: The newer LLM, Gemini 2.5 Flash offered large benefits across nearly all criteria. Our fine-tuned AMIE 1.5 Flash improved in some areas as compared to the corresponding Base 1.5 Flash (from which it was post-trained), particularly on many of the PACES criteria concerning thoroughness of history taking as well as DDx accuracy, but observed regressions in some areas like escalation and management.

Agents: The inclusion of both the Mx Agent and Dialogue Agent with Chain-of-reasoning (which included internal state updates and sequential model calls) each independently improved performance across nearly all criteria, regardless of the backend model used. However, these benefits were generally far less pronounced when using the newer Gemini models which already exhibited high performance. When paired together, in most criteria the combination of both agents was better than the Dialogue Agent alone. However, configuration Dialogue Agent (Prompt) + Mx Agent was rated less favorably than Dialogue Agent (CoR) without Mx Agent across many criteria. Notably, while we typically saw similar trends with the AMIE 1.5 Flash model, improvement from adding agents to the system were less consistent than the corresponding base model, possibly pointing to a worsened general instruction following capability (as the agents rely on unique instructions which were not part of the model post-training).

Support for configuration used in OSCE study: The configuration used in our OSCE study corresponds to backend model 2 (AMIE 1.5 Flash) paired with agent configuration 4 (Dialogue Agent (CoR) + Mx Agent). This choice is supported by our simulated auto-evaluation ablation. Agent configuration 4 (Dialogue Agent (CoR) + Mx Agent) performed most favorably across the vast majority of auto-rated criteria and across backend models. For the backend model, there was a trade-off. On the one hand, the post-training for AMIE 1.5 Flash helped to improve the system's ability to elicit and explain clinical information in a conversational manner (reflected in more favorable PACES ratings in auto-evaluation) and allowed it to perform more accurate diagnostic reasoning based on that information (reflected in higher DDx accuracy in auto-evaluation). On the other hand, we observed regressions in other areas for the post-trained model. The backend model tested in the OSCE study is supported by our results, as high-quality conversational interaction with patient actors, especially across multiple sequential visits, was crucial for the study.

Table SI.30 | Simulated Auto-evaluation Ablation Results: PACES. Results of completely AI-simulated and AI-rated interactions for the 100 3-visit scenarios. Each score corresponds to the proportion of visits (300 total) which were rated as favorable for that criterion by the LLM auto-evaluator (built on Gemini 2.5 Flash). Here, we compare 12 configurations in total, including each combination of the 3 different backend models (Base Gemini 1.5 Flash, our post-trained AMIE built on 1.5 Flash, and Gemini 2.5 Flash) with the 4 different agent configurations: 1) Dialogue Agent with just the response prompt, akin to calling the backend model directly, 2) Dialogue Agent with "chain-of-reasoning" (CoR), 3) Dialogue agent with just the response prompt, but with access to the Mx Agent responses, and 4) the complete setup used in the study (Dialogue Agent with CoR + MxAgent).

		Base 1.5 Flash	AMIE 1.5 Flash	Base 2.5 Flash
Confirms Patient Understanding	Dialogue Agent (Prompt)	0.627	0.697	0.887
	Dialogue Agent (CoR)	0.757	0.800	0.963
	Dialogue Agent (Prompt) + Mx Agent	0.697	0.777	0.940
	Dialogue Agent (CoR) + Mx Agent	0.773	0.807	0.960
Elicits Family History	Dialogue Agent (Prompt)	0.643	0.820	0.950
	Dialogue Agent (CoR)	0.827	0.873	0.963
	Dialogue Agent (Prompt) + Mx Agent	0.617	0.793	0.970
	Dialogue Agent (CoR) + Mx Agent	0.847	0.900	0.967
Elicits Medication History	Dialogue Agent (Prompt)	0.907	0.987	0.997
	Dialogue Agent (CoR)	0.940	0.987	0.990
	Dialogue Agent (Prompt) + Mx Agent	0.857	0.987	1.000
	Dialogue Agent (CoR) + Mx Agent	0.967	0.990	0.997
Elicits Medical History	Dialogue Agent (Prompt)	0.940	0.990	0.997
	Dialogue Agent (CoR)	0.990	0.997	0.997
	Dialogue Agent (Prompt) + Mx Agent	0.930	0.990	1.000
	Dialogue Agent (CoR) + Mx Agent	0.990	1.000	0.997
Explains Accurately	Dialogue Agent (Prompt)	0.983	0.967	1.000
	Dialogue Agent (CoR)	0.980	0.953	0.997
	Dialogue Agent (Prompt) + Mx Agent	0.967	0.967	1.000
	Dialogue Agent (CoR) + Mx Agent	0.983	0.967	1.000
Explains Comprehensively	Dialogue Agent (Prompt)	0.867	0.880	0.997
	Dialogue Agent (CoR)	0.877	0.843	0.993
	Dialogue Agent (Prompt) + Mx Agent	0.850	0.880	0.997
	Dialogue Agent (CoR) + Mx Agent	0.870	0.880	0.997
Maintains Patient Welfare	Dialogue Agent (Prompt)	0.960	0.943	0.993
	Dialogue Agent (CoR)	0.980	0.983	0.997
	Dialogue Agent (Prompt) + Mx Agent	0.970	0.977	1.000
	Dialogue Agent (CoR) + Mx Agent	0.983	0.973	0.997

Table SI.31 | Simulated Auto-evaluation Ablation Results: Diagnosis & Management. Results of completely AI-simulated and AI-rated interactions for the 100 3-visit scenarios. Each score corresponds to the proportion of visits (300 total) which were rated as favorable for that criterion by the LLM auto-evaluator (built on Gemini 2.5 Flash). Here, we compare 12 configurations in total, including each combination of the 3 different backend models (Base Gemini 1.5 Flash, our post-trained AMIE built on 1.5 Flash, and Gemini 2.5 Flash) with the 4 different agent configurations: 1) Dialogue Agent with just the response prompt, akin to calling the backend model directly, 2) Dialogue Agent with "chain-of-reasoning" (CoR), 3) Dialogue agent with just the response prompt, but with access to the Mx Agent responses, and 4) the complete setup used in the study (Dialogue Agent with CoR + MxAgent).

		Base 1.5 Flash	AMIE 1.5 Flash	Base 2.5 Flash
Avoids Inappropriate Investigations	Dialogue Agent (Prompt)	0.977	0.963	1.000
	Dialogue Agent (CoR)	0.987	0.977	0.993
	Dialogue Agent (Prompt) + Mx Agent	0.967	0.963	0.987
	Dialogue Agent (CoR) + Mx Agent	0.983	0.963	0.990
Avoids Inappropriate Treatments	Dialogue Agent (Prompt)	0.980	0.970	0.993
	Dialogue Agent (CoR)	0.980	0.970	0.993
	Dialogue Agent (Prompt) + Mx Agent	0.987	0.970	0.997
	Dialogue Agent (CoR) + Mx Agent	0.987	0.970	0.990
DDX Appropriateness	Dialogue Agent (Prompt)	0.847	0.830	0.970
	Dialogue Agent (CoR)	0.897	0.893	0.973
	Dialogue Agent (Prompt) + Mx Agent	0.877	0.920	0.980
	Dialogue Agent (CoR) + Mx Agent	0.887	0.867	0.970
DDx Comprehensiveness	Dialogue Agent (Prompt)	0.880	0.870	0.970
	Dialogue Agent (CoR)	0.873	0.910	0.970
	Dialogue Agent (Prompt) + Mx Agent	0.863	0.933	0.977
	Dialogue Agent (CoR) + Mx Agent	0.883	0.880	0.967
DDx Alternative Diagnoses	Dialogue Agent (Prompt)	0.753	0.773	0.913
	Dialogue Agent (CoR)	0.780	0.880	0.913
	Dialogue Agent (Prompt) + Mx Agent	0.797	0.873	0.903
	Dialogue Agent (CoR) + Mx Agent	0.777	0.850	0.920
DDx Probable Diagnosis	Dialogue Agent (Prompt)	0.877	0.873	0.960
	Dialogue Agent (CoR)	0.890	0.900	0.960
	Dialogue Agent (Prompt) + Mx Agent	0.867	0.923	0.967
	Dialogue Agent (CoR) + Mx Agent	0.913	0.917	0.953
Follow-Up Appropriateness	Dialogue Agent (Prompt)	0.967	0.927	0.993
	Dialogue Agent (CoR)	0.977	0.970	0.993
	Dialogue Agent (Prompt) + Mx Agent	0.977	0.960	0.977
	Dialogue Agent (CoR) + Mx Agent	0.973	0.960	0.983
Escalation Appropriateness	Dialogue Agent (Prompt)	0.903	0.850	0.980
	Dialogue Agent (CoR)	0.933	0.907	0.977
	Dialogue Agent (Prompt) + Mx Agent	0.940	0.923	0.980
	Dialogue Agent (CoR) + Mx Agent	0.940	0.897	0.983
Management Plan Appropriateness	Dialogue Agent (Prompt)	0.777	0.697	0.950
	Dialogue Agent (CoR)	0.820	0.757	0.947
	Dialogue Agent (Prompt) + Mx Agent	0.803	0.787	0.947
	Dialogue Agent (CoR) + Mx Agent	0.790	0.723	0.937

Table SI.32 | Simulated Auto-evaluation Ablation Results: MXEKF. Results of completely AI-simulated and AI-rated interactions for the 100 3-visit scenarios. Each score corresponds to the proportion of visits (300 total) which were rated as favorable for that criterion by the LLM auto-evaluator (built on Gemini 2.5 Flash). Here, we compare 12 configurations in total, including each combination of the 3 different backend models (Base Gemini 1.5 Flash, our post-trained AMIE built on 1.5 Flash, and Gemini 2.5 Flash) with the 4 different agent configurations: 1) Dialogue Agent with just the response prompt, akin to calling the backend model directly, 2) Dialogue Agent with "chain-of-reasoning" (CoR), 3) Dialogue agent with just the response prompt, but with access to the Mx Agent responses, and 4) the complete setup used in the study (Dialogue Agent with CoR + MxAgent).

		Base 1.5 Flash	AMIE 1.5 Flash	Base 2.5 Flash
Acting as Teacher/Salesperson	Dialogue Agent (Prompt)	0.680	0.703	0.960
	Dialogue Agent (CoR)	0.843	0.820	0.987
	Dialogue Agent (Prompt) + Mx Agent	0.773	0.820	0.983
	Dialogue Agent (CoR) + Mx Agent	0.847	0.820	0.987
Shared Decision Making	Dialogue Agent (Prompt)	0.893	0.873	0.990
	Dialogue Agent (CoR)	0.983	0.950	0.997
	Dialogue Agent (Prompt) + Mx Agent	0.947	0.950	0.993
	Dialogue Agent (CoR) + Mx Agent	0.963	0.940	1.000
Contrasting & Selection	Dialogue Agent (Prompt)	0.603	0.570	0.903
	Dialogue Agent (CoR)	0.733	0.647	0.940
	Dialogue Agent (Prompt) + Mx Agent	0.647	0.703	0.920
	Dialogue Agent (CoR) + Mx Agent	0.727	0.657	0.933
Dynamic Interplay	Dialogue Agent (Prompt)	0.637	0.573	0.923
	Dialogue Agent (CoR)	0.790	0.667	0.953
	Dialogue Agent (Prompt) + Mx Agent	0.730	0.733	0.953
	Dialogue Agent (CoR) + Mx Agent	0.800	0.687	0.960
Illness-specific Knowledge	Dialogue Agent (Prompt)	0.677	0.620	0.950
	Dialogue Agent (CoR)	0.813	0.720	0.977
	Dialogue Agent (Prompt) + Mx Agent	0.730	0.760	0.980
	Dialogue Agent (CoR) + Mx Agent	0.797	0.713	0.980
Monitoring & Adjustment	Dialogue Agent (Prompt)	0.670	0.590	0.913
	Dialogue Agent (CoR)	0.820	0.727	0.963
	Dialogue Agent (Prompt) + Mx Agent	0.780	0.757	0.943
	Dialogue Agent (CoR) + Mx Agent	0.823	0.730	0.963
Organization Of Encounter	Dialogue Agent (Prompt)	0.960	0.947	0.993
	Dialogue Agent (CoR)	0.973	0.957	0.997
	Dialogue Agent (Prompt) + Mx Agent	0.977	0.970	0.990
	Dialogue Agent (CoR) + Mx Agent	0.960	0.943	0.997
Prioritizes Preferences	Dialogue Agent (Prompt)	0.923	0.917	0.990
	Dialogue Agent (CoR)	0.983	0.950	0.993
	Dialogue Agent (Prompt) + Mx Agent	0.943	0.960	0.997
	Dialogue Agent (CoR) + Mx Agent	0.970	0.953	1.000
Appropriate Prognostication	Dialogue Agent (Prompt)	0.780	0.787	0.980
	Dialogue Agent (CoR)	0.883	0.840	0.993
	Dialogue Agent (Prompt) + Mx Agent	0.863	0.877	0.983
	Dialogue Agent (CoR) + Mx Agent	0.887	0.860	0.987

SI.15 RxQA Medication Reasoning Benchmark

Ms. Evelyn Reed is a 68-year-old woman who arrived at the emergency department due to a bee sting. She has a history of allergic rhinitis, for which she takes loratadine daily. She also has type 2 diabetes and hypertension, managed with metformin and lisinopril, respectively. Upon examination, Ms. Reed exhibits urticaria, angioedema, dizziness, and lightheadedness. Her blood pressure is 90/60 mmHg, and her heart rate is 110 bpm. Considering the immediate need to address her allergic reaction and her current hemodynamic status, determine the most appropriate initial dose and administration route for diphenhydramine hydrochloride.

A. 25 mg intramuscularly
B. 50 mg intravenously over 1 minute
C. 25 mg intravenously over 5 minutes
D. 10 mg orally

Figure SI.2 | RxQA Example Question. An example RxQA question derived from OpenFDA medication labels.

Table SI.33 | RxQA Medication Reasoning Accuracy: Comparison of AMIE vs. PCP with Difficulty Breakdown. Comparison of question-answering accuracy between combinations of test takers (PCP, AMIE) and settings ('closed'-book, 'open'-book) and for different subsets of questions. Accuracy is shown as mean and 95% confidence intervals for a binomial proportions. P-values are from McNemar tests with false discovery rate (FDR) correction. Significance levels correspond to $p<0.001$ (***), $p<0.01$ (**), $p<0.05$ (*) and not significant (n.s.). Note that overlapping confidence intervals can still allow for $p<0.05$ as McNemar test makes use of question-level pairing.

Pharmacist-rated Difficulty	N	Test Taker & Setting A	Accuracy A [%]	Test Taker & Setting B	Accuracy B [%]	P-value	Sig. Level
Both	600	AMIE closed	51.7 (47.7, 55.7)	AMIE open	65.3 (61.5, 69.1)	6.72e-11	***
		AMIE closed	51.7 (47.7, 55.7)	PCP closed	43.8 (39.9, 47.8)	5.68e-03	**
		AMIE closed	51.7 (47.7, 55.7)	PCP open	57.0 (53.0, 61.0)	5.53e-02	n.s.
		AMIE open	65.3 (61.5, 69.1)	PCP closed	43.8 (39.9, 47.8)	2.40e-15	***
		AMIE open	65.3 (61.5, 69.1)	PCP open	57.0 (53.0, 61.0)	6.31e-04	***
		PCP closed	43.8 (39.9, 47.8)	PCP open	57.0 (53.0, 61.0)	5.97e-09	***
Higher	318	AMIE closed	50.6 (45.1, 56.1)	AMIE open	57.9 (52.4, 63.3)	1.01e-02	*
		AMIE closed	50.6 (45.1, 56.1)	PCP closed	41.5 (36.1, 46.9)	1.29e-02	*
		AMIE closed	50.6 (45.1, 56.1)	PCP open	47.8 (42.3, 53.3)	4.56e-01	n.s.
		AMIE open	57.9 (52.4, 63.3)	PCP closed	41.5 (36.1, 46.9)	7.17e-06	***
		AMIE open	57.9 (52.4, 63.3)	PCP open	47.8 (42.3, 53.3)	3.22e-03	**
		PCP closed	41.5 (36.1, 46.9)	PCP open	47.8 (42.3, 53.3)	4.53e-02	*
Lower	282	AMIE closed	52.8 (47.0, 58.7)	AMIE open	73.8 (68.6, 78.9)	1.08e-10	***
		AMIE closed	52.8 (47.0, 58.7)	PCP closed	46.5 (40.6, 52.3)	1.47e-01	n.s.
		AMIE closed	52.8 (47.0, 58.7)	PCP open	67.4 (61.9, 72.8)	6.94e-04	***
		AMIE open	73.8 (68.6, 78.9)	PCP closed	46.5 (40.6, 52.3)	4.37e-11	***
		AMIE open	73.8 (68.6, 78.9)	PCP open	67.4 (61.9, 72.8)	7.08e-02	n.s.
		PCP closed	46.5 (40.6, 52.3)	PCP open	67.4 (61.9, 72.8)	1.60e-09	***

Table SI.34 | RxQA Medication Reasoning Accuracy: Comparison of AMIE and PCP with other LLMs with Breakdown by Drug Database. In the open-book setting, the context is set to the drug labels provided to the ones used to derive each question, except when using the AMIE system. AMIE incorporates dynamic retrieval of drug labels based on the context of the question (marked with ⋆). Accuracy is shown as mean and 95% confidence intervals for a binomial proportions. The BNF portion only includes variants based on Google models, as license restrictions prevent using this part of the benchmark on other commercial model services.

	**OpenFDA portion** (n=300)		**BNF portion** (n=300)	
	closed-book	*open-book*	*closed-book*	*open-book*
PCP	48.3 (42.3, 53.7)	61.3 (55.5, 66.5)	39.3 (33.5, 44.5)	52.7 (47.0, 58.3)
AMIE (1.5 Flash)	59.0 (53.4, 64.6)	72.0⋆ (66.9, 77.1)	44.3 (38.4, 49.6)	58.7⋆ (53.1, 64.2)
AMIE (2.5 Pro)	74.0 (69.0, 79.0)	76.3⋆ (71.2, 80.8)	58.7 (53.1, 64.2)	62.7⋆ (57.2, 68.1)
Gemini 1.5 Flash	48.1 (42.3, 53.7)	69.0 (63.8, 74.2)	36.0 (30.6, 41.4)	57.9 (52.1, 63.3)
Gemini 2.5 Flash	65.5 (59.9, 70.7)	74.8 (69.7, 79.6)	53.9 (48.0, 59.3)	59.7 (54.1, 65.2)
Gemini 2.5 Pro	73.1 (68.0, 78.0)	74.7 (69.7, 79.6)	58.4 (52.8, 63.9)	62.2 (56.5, 67.5)
DeepSeek-V3	61.7 (56.2, 67.2)	71.9 (66.6, 76.8)	–	–
o-3	70.1 (64.8, 75.2)	76.5 (71.5, 81.1)	–	–
GPT-5	71.7 (66.6, 76.8)	76.3 (71.2, 80.8)	–	–

SI.16 RxQA Details

Medication labels from OpenFDA and British National Formulary (BNF) included information about the medication, its brand/generic names, form, dosages, indications, contraindications, use in specific populations, interactions and more. From OpenFDA, we selected 300 commonly used drugs (see the ClinCalc DrugStats Database [62]), as well as a random set of 500 additional drugs. 1748 drugs from BNF were used; we expected the resulting questions to be more difficult on average due to being less skewed towards well-known medications. Gemini 1.5 Flash was used to generate, validate, and filter multiple choice questions for RxQA.

SI.16.1 Short Question Generation

Short questions were generated zero-shot by simply providing the formatted medication label as context and asking the model to produce multiple choice questions with four options. The specific prompt was as follows:

RxQA Short Question Generation

```
Instructions: Create multiple choice questions involving the following medication: {drug_name}

Your questions should be understandable without having access to the medication information, and
clearly state the name of the medication being asked about. Your questions should be diverse and
challenging, but the answer should be clear and unambiguous. Answering the question should be possible
by an experienced pharmacist without access to this medication label.
{medication_information}
```

Table SI.35 | **Prompt:** RxQA Short MCQ Generation

SI.16.2 Long Question Generation

Longer questions were generated through a multi-step process. For the OpenFDA-sourced questions, up to 2 related medication labels were appended to the chosen medication label, randomly selected from drug names present in the original label. We then generated a list of challenges associated with each drug, using the prompt in Table SI.36. Next, we used the prompt in Table SI.37 to generate specific patient scenarios which test understanding of a subset of these challenges. Finally, we generated multiple choice questions based on these scenarios, each with a single correct answer using the prompt in Table SI.38.

RxQA Long Question Generation - Medication Challenges

```
Instructions: Consider the following medication: {drug_name}
The following is some detailed information about the medication.
{medication_information}

Your task is to create a thorough summary of the challenges associated with this medication. In
particular, you want to describe key things an expert pharmacist should be aware of, including nuances.
You should include both information in the medication label, as well as any additional nuances about
both the medication and relevant medical conditions which need to be considered.
Pay attention to the following:
  • What are the indications/contraindications for this medication?
  • What are the risks/side effects/interactions for this medication?
  • What are important things to consider when taking this medication?
  • What about use in specific populations?
Output a write-up of any notable challenges associated with this medication. Also list any key
medication interactions to watch out for.
```

Table SI.36 | **Prompt:** RxQA Long Question Generation - Medication Challenges

RxQA Long Question Generation - Patient Scenarios

```
Instructions: Consider the following medication: {drug_name}
Here is some detailed information about the medication:
{medication_information}

Here is a write-up of the challenges associated with this medication:
{generated_challenges}

Your task is to create a list of patient scenarios involving this medication.
Your scenarios should be diverse and challenging, including details about the patients, their
health histories, and their medications. The scenarios should be ones which test the pharmacist's
understanding of the indications, contraindications, risks, side effects, interactions, etc. for the
given medications, as well as any challenges with the condition(s) the patient is experiencing.
```

Table SI.37 | **Prompt:** RxQA Long Question Generation - Patient Scenarios

RxQA Long Question Generation

```
Instructions: Create a challenging multiple choice question, which requires careful weighing of
multiple (possibly conflicting) sources of information.

Medication: {drug_name}
Medication information:
{medication_information}

The patient scenario:
{scenario}

=== Question Guidelines ====
```

- The question should fully describe the patient scenario, without presenting the associated challenges (or even suggesting that there are challenges). This description should be in paragraph form, and at least 5 sentences. The question should be understandable without access to any other information.
- The question should test the pharmacists ability to use their deep expertise and knowledge of the medications/clinical conditions.
- The question should test the pharmacist on their ability to recognize specific aspects of the medication which may require understanding of the drug interactions, multiple comorbidities, use in specific populations, etc.
- The question should have 4 multiple choice answers, where only one of the choices is correct.
- The question should be challenging and not be superficially answerable by simply looking up the drug. Information about the drugs should not be presented in the question itself.
- The answer choices should be direct responses to the question that is being asked.
- All information needed should be available in the question such that a highly experienced pharmacist could select the correct answer unambiguously

Table SI.38 | **Prompt:** RxQA Long Question Generation

SI.16.3 Validation Prompts

We auto-evaluated these generated questions to help ensure they met an adequate quality standard. First, we checked whether the question itself was worded clearly and was adequately specified using the prompt in Table SI.39. We also confirmed with string matching that the correct answer choice was listed as one of the options. We then asked the model if it believed the listed answer was correct for the given question using the prompt in Table SI.40. Finally, we asked the model if the correct answer was the *only* correct answer using the prompt in Table SI.41, removing questions which had multiple good answer choices.

MCQ Validation - Question Quality

```
Determine if the following question is clear or not.
A clear question is one that is understandable and answerable by an experienced pharmacist without
having access to any additional information or clarification.

Examples:
Q: What is the recommended starting dose of the medication?
A. 5mg
B. 10mg
C. 15mg
D. 20mg
-> no

Q: What is the recommended starting dose of ritalin in adults?
A. 5mg
B. 10mg
C. 15mg
D. 20mg
-> yes

Q: {question}
```

Table SI.39 | **Prompt:** MCQ Validation - Question Quality

MCQ Validation - Answer Correctness

```
Consider the following medication information: {medication_info}

I am using this information to answer the following question: {question}

I believe the correct answer is {answer}. Am I correct?
```

Table SI.40 | **Prompt:** MCQ Validation - Answer Correctness

SI.16.4 MCQ Selection

Gemini 1.5 Flash was asked to answer each generated question zero-shot, both with and without the medication context. We identified the set of questions which the model answered correctly when it had the context, and incorrectly without the context. Note that what was considered correct at this stage was not necessarily the same as what the pharmacists selected to be correct in the next step (Section SI.16.5). For both the OpenFDA-sourced and BNF-sourced questions, we randomly sampled 100 short and 200 long questions which met this criteria as well as the validation criteria in Section SI.16.3 to send out for pharmacist revision.

MCQ Validation - Answer Uniqueness

```
Consider the following medication information: {medication_info}

I am using this information to answer the following question: {question}

The correct answer choice was listed as {answer}, and I want to make sure the other answer choices
are incorrect.

Are there multiple correct answers to this question in the answer choices?
```

Table SI.41 | **Prompt:** MCQ Validation - Answer Uniqueness

SI.16.5 Pharmacist Revision

The questions and selected answer options were each revised by one of four pharmacists in each jurisdiction. In revision, the pharmacist was allowed to change the wording of the question and all of the answer choices to ensure that the question was high quality and had exactly one correct answer. Following this revision, the pharmacist selected what they believed to be the correct answer, which may not be the same as what the model initially believed. Afterwards, the pharmacists also rated the difficulty of each question on the following scale: Trivial, Easy, Medium, Hard, and Impossible. For subgroup analysis purposes, Trivial and Easy were mapped to lower difficulty, while Medium, Hard, and Impossible mapped to higher difficulty. These pharmacist-revised set of questions are what make up the final RxQA benchmark. An example question, derived from OpenFDA, is shown in Figure SI.2.

References

[53] Zheng, L. *et al.* Judging llm-as-a-judge with mt-bench and chatbot arena (2023). URL https://arxiv.org/abs/2306.05685. 2306.05685.

[54] Panickssery, A., Bowman, S. & Feng, S. Llm evaluators recognize and favor their own generations. *Advances in Neural Information Processing Systems* **37**, 68772–68802 (2025).

[55] Wei, J. *et al.* Chain-of-thought prompting elicits reasoning in large language models. *Advances in Neural Information Processing Systems* **35**, 24824–24837 (2022).

[56] Kojima, T., Gu, S. S., Reid, M., Matsuo, Y. & Iwasawa, Y. Large language models are zero-shot reasoners. *arXiv preprint arXiv:2205.11916* (2022).

[57] Mirowski, P., Mathewson, K. W., Pittman, J. & Evans, R. Co-writing screenplays and theatre scripts with language models: Evaluation by industry professionals. In *Proceedings of the 2023 CHI Conference on Human Factors in Computing Systems*, 1–34 (2023).

[58] Madaan, A. *et al.* Self-refine: Iterative refinement with self-feedback. *arXiv preprint arXiv:2303.17651* (2023).

[59] Liu, M. X. *et al.* "we need structured output": Towards user-centered constraints on large language model output. In *Extended Abstracts of the CHI Conference on Human Factors in Computing Systems*, 1–9 (2024).

[60] Jin, D. *et al.* What disease does this patient have? a large-scale open domain question answering dataset from medical exams. *Applied Sciences* **11**, 6421 (2021).

[61] Randolph, J. J. Free-marginal multirater kappa (multirater k [free]): An alternative to fleiss' fixed-marginal multirater kappa. *Online submission* (2005).

[62] SP, K. The top 300 of 2022, clincalc drugstats database version 2024.08 (2024). URL https://clincalc.com/DrugStats/Top300Drugs.aspx.

[63] De Dombal, F. T., Leaper, D. J., Horrocks, J. C., Staniland, J. R. & McCann, A. P. Human and computer-aided diagnosis of abdominal pain: further report with emphasis on performance of clinicians. *Br. Med. J.* **1**, 376–380 (1974).

[64] Shortliffe, E. H. Computer programs to support clinical decision making. *Jama* **258**, 61–66 (1987).

[65] Elstein, A. S. Cognitive processes in clinical inference and decision making. (1988). URL https://api.semanticscholar.org/CorpusID:197751266.

[66] Bordage, G. & Lemieux, M. Semantic structures and diagnostic thinking of experts and novices. *Acad. Med.* **66**, S70–2 (1991).

[67] Croskerry, P. A universal model of diagnostic reasoning. *Acad. Med.* **84**, 1022–1028 (2009).

[68] Adams, I. D. *et al.* Computer aided diagnosis of acute abdominal pain: a multicentre study. *Br. Med. J. (Clin. Res. Ed)* **293**, 800–804 (1986).

[69] Williams, C. Y. K. *et al.* Use of a large language model to assess clinical acuity of adults in the emergency department. *JAMA Netw. Open* **7**, e248895 (2024).

[70] Chen, L.-C. *et al.* Assessing large language models for oncology data inference from radiology reports. *JCO Clin. Cancer Inform.* **8**, e2400126 (2024).

[71] Williams, C. Y. K., Miao, B. Y., Kornblith, A. E. & Butte, A. J. Evaluating the use of large language models to provide clinical recommendations in the emergency department. *Nat. Commun.* **15**, 8236 (2024).

[72] Khandekar, N. *et al.* MedCalc-bench: Evaluating large language models for medical calculations. *arXiv [cs.CL]* (2024).

[73] Lewis, P. *et al.* Retrieval-augmented generation for knowledge-intensive nlp tasks (2021). URL https://arxiv.org/abs/2005.11401. 2005.11401.

[74] Zakka, C. *et al.* Almanac - retrieval-augmented language models for clinical medicine. *NEJM AI* **1** (2024).

[75] Omar, M., Nadkarni, G. N., Klang, E. & Glicksberg, B. S. Large language models in medicine: A review of current clinical trials across healthcare applications. *PLOS Digit. Health* **3**, e0000662 (2024).

[76] Johri, S. *et al.* An evaluation framework for clinical use of large language models in patient interaction tasks. *Nat. Med.* **31**, 77–86 (2025).

[77] Mukherjee, S. *et al.* Polaris: A safety-focused LLM constellation architecture for healthcare. *arXiv [cs.AI]* (2024).

[78] Zeltzer, D. *et al.* Diagnostic accuracy of artificial intelligence in virtual primary care. *Mayo Clinic Proceedings: Digital Health* **1**, 480–489 (2023).

[79] Lizée, A. *et al.* Conversational medical AI: Ready for practice. *arXiv [cs.AI]* (2024).

[80] Harden, R. M., Stevenson, M., Downie, W. W. & Wilson, G. M. Assessment of clinical competence using objective structured examination. *BMJ* **1**, 447–451 (1975).

[81] Gentles, H. Putting them through their PACES. practical assessment of clinical examination skills. *BMJ* **326**, S76 (2003).

[82] Ogunyemi, D. & Dupras, D. Does an objective structured clinical examination fit your assessment toolbox? *J. Grad. Med. Educ.* **9**, 771–772 (2017).

[83] Malau-Aduli, B. S. *et al.* Inter-rater reliability: Comparison of checklist and global scoring for OSCEs. *Creat. Educ.* **03**, 937–942 (2012).

[84] Park, J. S. *et al.* Generative agents: Interactive simulacra of human behavior (2023). URL https://arxiv.org/abs/2304.03442. 2304.03442.

[85] Li, J. *et al.* Agent hospital: A simulacrum of hospital with evolvable medical agents (2025). URL https://arxiv.org/abs/2405.02957. 2405.02957.

[86] Vezhnevets, A. S. *et al.* Generative agent-based modeling with actions grounded in physical, social, or digital space using concordia (2023). URL https://arxiv.org/abs/2312.03664. 2312.03664.

[87] Shen, Y. *et al.* Hugginggpt: Solving ai tasks with chatgpt and its friends in hugging face (2023). URL https://arxiv.org/abs/2303.17580. 2303.17580.

[88] Mukherjee, S. *et al.* Polaris: A safety-focused llm constellation architecture for healthcare. *arXiv preprint arXiv:2403.13313* (2024).

[89] Christakopoulou, K., Mourad, S. & Matarić, M. Agents thinking fast and slow: A talker-reasoner architecture. *arXiv preprint arXiv:2410.08328* (2024).

[90] Wang, Z. *et al.* Describe, explain, plan and select: Interactive planning with large language models enables open-world multi-task agents (2024). URL https://arxiv.org/abs/2302.01560. 2302.01560.

[91] Ahn, M. *et al.* Do as i can, not as i say: Grounding language in robotic affordances (2022). URL https://arxiv.org/abs/2204.01691. 2204.01691.

[92] Wang, G. *et al.* Voyager: An open-ended embodied agent with large language models (2023). URL https://arxiv.org/abs/2305.16291. 2305.16291.

[93] Nori, H. *et al.* Can generalist foundation models outcompete special-purpose tuning? case study in medicine. *arXiv preprint arXiv:2311.16452* (2023).

[94] Yao, S. *et al.* React: Synergizing reasoning and acting in language models. In *International Conference on Learning Representations (ICLR)* (2023).

[95] Shinn, N. *et al.* Reflexion: Language agents with verbal reinforcement learning (2023). URL https://arxiv.org/abs/2303.11366. 2303.11366.

[96] Singh, A., Ehtesham, A., Kumar, S. & Khoei, T. T. Agentic retrieval-augmented generation: A survey on agentic rag (2025). URL https://arxiv.org/abs/2501.09136. 2501.09136.

[97] Zhou, P. *et al.* Self-discover: Large language models self-compose reasoning structures (2024). URL https://arxiv.org/abs/2402.03620. 2402.03620.

[98] Tam, Z. R. *et al.* Let me speak freely? a study on the impact of format restrictions on performance of large language models (2024). URL https://arxiv.org/abs/2408.02442. 2408.02442.
