# Supplementary material for: Towards conversational artificial intelligence for disease management
Source: Nature. 2026 Jun 17;655(8125):1292–9. doi: 10.1038/s41586-026-10764-5 (PMC13421318; doi:10.1038/s41586-026-10764-5)
Supplement: Supplementary file 2 — Reporting Summary [file 41586_2026_10764_MOESM2_ESM.pdf]

nature portfolio

Corresponding author(s): Valentin Liévin, Anil Palepu, Alan Karthikesalingam, Mike Schaekermann

Last updated by author(s): May 26, 2026

Reporting Summary

Nature Portfolio wishes to improve the reproducibility of the work that we publish. This form provides structure for consistency and transparency in reporting. For further information on Nature Portfolio policies, see our Editorial Policies and the Editorial Policy Checklist.

Statistics

For all statistical analyses, confirm that the following items are present in the figure legend, table legend, main text, or Methods section.

n/a	Confirmed	
☐	☒	The exact sample size (n) for each experimental group/condition, given as a discrete number and unit of measurement
☐	☒	A statement on whether measurements were taken from distinct samples or whether the same sample was measured repeatedly
☐	☒	The statistical test(s) used AND whether they are one- or two-sided<br>*Only common tests should be described solely by name; describe more complex techniques in the Methods section.*
☐	☒	A description of all covariates tested
☐	☒	A description of any assumptions or corrections, such as tests of normality and adjustment for multiple comparisons
☐	☒	A full description of the statistical parameters including central tendency (e.g. means) or other basic estimates (e.g. regression coefficient) AND variation (e.g. standard deviation) or associated estimates of uncertainty (e.g. confidence intervals)
☐	☒	For null hypothesis testing, the test statistic (e.g. F, t, r) with confidence intervals, effect sizes, degrees of freedom and P value noted<br>*Give P values as exact values whenever suitable.*
☒	☐	For Bayesian analysis, information on the choice of priors and Markov chain Monte Carlo settings
☒	☐	For hierarchical and complex designs, identification of the appropriate level for tests and full reporting of outcomes
☐	☒	Estimates of effect sizes (e.g. Cohen's d, Pearson's r), indicating how they were calculated

Our web collection on statistics for biologists contains articles on many of the points above.

Software and code

Policy information about availability of computer code

Data collection	The algorithms and scripts for data collection were implemented using Python 2.7.18. Gemini 1.5 Flash was used as a base model for parts of the AMIE system tested in this work. Accuracy results on the RxQA medication reasoning benchmark were obtained for Gemini 1.5 Flash, Gemini 2.5 Flash, Gemini 2.5 Pro, DeepSeek-V3, o-3, and GPT-5 models.
Data analysis	The data analysis scripts were implemented in Python 2.7.18. We will not be able to open source the LLMs used in this study.

For manuscripts utilizing custom algorithms or software that are central to the research but not yet described in published literature, software must be made available to editors and reviewers. We strongly encourage code deposition in a community repository (e.g. GitHub). See the Nature Portfolio guidelines for submitting code & software for further information.

Data

Policy information about availability of data

All manuscripts must include a data availability statement. This statement should provide the following information, where applicable:

- Accession codes, unique identifiers, or web links for publicly available datasets
- A description of any restrictions on data availability
- For clinical datasets or third party data, please ensure that the statement adheres to our policy

Some of the datasets used in the development of AMIE are open-source (MedQA, MultiMedQA, MIMIC-III). We make details of the 120 OSCE scenarios used in this work (20 for validation and 100 for evaluation) available as part of this publication, including AMIE output for all 120 scenarios. For two sample scenarios, we also

share PCP output and ratings from patient actors and specialist physicians. These OSCE study details are provided as part of the Supplementary Information. The OpenFDA portion of the RxQA dataset is available at: https://github.com/Google-Health/rxqa. Due to licensing restrictions, the BNF portion cannot be shared directly by Google. However, we have shared this portion with BNF itself, and the interested reader is encouraged to contact BNF to request access to the data.

Research involving human participants, their data, or biological material

Policy information about studies with human participants or human data. See also policy information about sex, gender (identity/presentation), and sexual orientation and race, ethnicity and racism.

Reporting on sex and gender	N/A
Reporting on race, ethnicity, or other socially relevant groupings	N/A
Population characteristics	N/A
Recruitment	N/A
Ethics oversight	N/A

Note that full information on the approval of the study protocol must also be provided in the manuscript.

Field-specific reporting

Please select the one below that is the best fit for your research. If you are not sure, read the appropriate sections before making your selection.

☒ Life sciences ☐ Behavioural & social sciences ☐ Ecological, evolutionary & environmental sciences

For a reference copy of the document with all sections, see nature.com/documents/nr-reporting-summary-flat.pdf

Life sciences study design

All studies must disclose on these points even when the disclosure is negative.

Sample size	The study involved 100 OSCE scenarios with 3 distinct virtual visits each. No sample size calculation was performed. The sample was selected to cover 5 different medical specialties and 2 geographic locations. Its size was bounded by practical constraints including budget and time.
Data exclusions	No data was excluded.
Replication	The evaluations were performed by specialist physicians. Each case was evaluated by three distinct specialist physician raters. The patient actors were sourced from two different institutions in two separate countries and the OSCE scenario packs were from the same two countries. Inter-rater reliability statistics from specialist physician raters suggest that the replication was successful.
Randomization	The order in which (a) patient actors completed both study arms, and (b) specialist physicians assessed quality for both study arms, was randomized.
Blinding	Patient actors and specialist physician evaluators were not told which study arm they were exposed to during text-based conversation and evaluation respectively.

Reporting for specific materials, systems and methods

We require information from authors about some types of materials, experimental systems and methods used in many studies. Here, indicate whether each material, system or method listed is relevant to your study. If you are not sure if a list item applies to your research, read the appropriate section before selecting a response.

Materials & experimental systems

n/a	Involved in the study	
☒	☐	Antibodies
☒	☐	Eukaryotic cell lines
☒	☐	Palaeontology and archaeology
☒	☐	Animals and other organisms
☒	☐	Clinical data
☒	☐	Dual use research of concern
☒	☐	Plants

Methods

n/a	Involved in the study	
☒	☐	ChIP-seq
☒	☐	Flow cytometry
☒	☐	MRI-based neuroimaging

Plants

Seed stocks	N/A
Novel plant genotypes	N/A
Authentication	N/A
