# Supplementary material for: Towards conversational artificial intelligence for disease management
Source: Nature. 2026 Jun 17;655(8125):1292–9. doi: 10.1038/s41586-026-10764-5 (PMC13421318; doi:10.1038/s41586-026-10764-5)
Supplement: Supplementary file 3 — Detailed view of two sample scenarios with AMIE and PCP output and evaluation gradings. Full details for two sample scenarios used in the OSCE evaluation study, including scenario information, AMIE–patient-actor conversations, PCP–patient-actor conversations, specialist physician gradings and patient actor gradings for all three visits per scenario. [file 41586_2026_10764_MOESM3_ESM.pdf]

Detailed view of two sample scenarios

Summary of information provided for each scenario
Scenario A (Rheumatoid arthritis)
  Information for the patient actor
  Visit 1
  Visit 2
  Visit 3
Scenario B (Celiac disease)
  Information for the patient actor
  Visit 1
  Visit 2
  Visit 3

We provide a detailed view of two sample scenarios below. A summary of the information provided for each scenario is given on the next page to help navigate the material. Scenarios were sampled to showcase a mix of both strengths and areas for improvement in AMIE and PCPs respectively, based on a combination of rating scores and qualitative review. We focused on cases which included new diagnostic information (e.g. lab results or imaging reports) being made available in the course of the multi-visit interaction.

Scenario A is a case of rheumatoid arthritis with x-ray imaging reports. In this case, AMIE successfully performed a disease characterization with the patient actor, but certain elements could have been emphasized more strongly in an ideal consultation. For example, more detail on joint distribution including symmetricality of the small joints could have been elicited; drawing out more clearly that the patient is a smoker as this is a risk factor; elucidating duration and intensity, or "pack-years"; and advising and supporting smoking cessation. AMIE also tended to use technical terms (such as 'inflammatory arthritides' or 'minimize potential gastrointestinal side effects') more often than the PCP for this case. In comparison, the PCP was more direct, though less explanatory, and could perhaps be seen as abrupt, which may relate to the unfamiliar text-based communication. Both AMIE and PCP provided management plans that were generally well aligned with the relevant clinical guidelines. However, for a subset of individual items on AMIE's management plan, recommendations were not explicitly mentioned in guidelines, yet reasonable.

Scenario B is a case of celiac disease which includes lab results. In this case, AMIE's management plan included certain items that were not supported by the referenced guidelines. For example, after the first visit, AMIE recommends ordering a stool test for fat content to assess for malabsorption and screen for infectious causes. This could not be found in the referenced guidance during qualitative review, and may not be considered routine practice.

Summary of information provided for each scenario

**Scenario information for the patient actor**	**For each of 3 visits, we provide the following information for both AMIE and PCP in a side-by-side manner.**
Patient Background:<br><br>● Name<br>● Sex<br>● Age<br>● Ethnic background<br>● Location<br>● Lifestyle<br>● Social and personal circumstances<br>● Occupational history<br>● Travel history<br>● Family history<br><br>For each of 3 visits:<br><br>● Presenting complaint<br>● Initial presentation<br>● Context<br>● Information to be shared to the doctor if the patient is explicitly asked<br>● Past medical history and surgical history<br>● Current medications<br>● Relevant previous medications<br>● Allergies and adverse reactions<br>● Patient concerns, expectations and wishes<br>● Questions the patient wants to ask the doctor<br>● In the case where the previous visit did not go as planned, alternative course of action the patient would have taken on their own (only applicable to follow-up visits)	Information available at the beginning of the visit (including new lab or imaging results, etc)<br><br>Multi-turn text chat dialogue with the patient actor<br><br>Responses to post-questionnaire:<br><br>● Guidelines selected as relevant<br>● Differential diagnosis<br>● In-visit investigations<br>● Ordered investigations<br>● Recommendations or actions<br><br>● Did you intentionally deviate from one of the clinical guidelines you selected above?<br>● If yes, please explain why (e.g. guideline does not cover this clinical scenario, patient preference, your preference). If no, enter 'N/A'.<br>● What are other acceptable management plans? Why did you choose yours?<br>● Please describe any competing priorities that affected your decisions.<br>● Describe the cost, effectiveness and side effects of your management plan.<br>● Describe the prognosis with and without treatment of this patient.<br>● Does this case require escalation of this text-based consultation to a video-call or in-person consultation?<br>● Describe why it requires or does not require escalation.<br>● Is a follow-up required?<br>● If yes, when would you like to follow-up the patient? Otherwise, enter 'N/A'.<br>● Describe why it requires or does not require follow-up.<br><br>Specialist physician and patient actor ratings

Scenario A (Rheumatoid arthritis)

Information for the patient actor

<table>
<tr><th colspan="3">Patient background</th></tr>
<tr><td colspan="3">

Name: Oluwassen Odozor
Sex: Female
Age: 53 years
Ethnic background: Nigerian
Location: Nottingham, UK
Lifestyle: Has smoked 10 cigarettes/ day for the past 30 years. Drinks socially only. Work involves long hours sitting and typing. Exercises by going to dance classes. Diet is healthy.
Social and personal circumstances: Lives with a supportive husband. Has a daughter in university.
Occupational history: Accountant
Travel history: N/A
Family history: Mother had arthritis

</td></tr>
<tr><th>Visit 1</th><th>Visit 2</th><th>Visit 3</th></tr>
<tr><th colspan="3">In the case where the previous visit did not go as planned, alternative course of action the patient would have taken on their own:</th></tr>
<tr><td>N/A</td><td>This is a follow up to discuss the results of the bloodwork and imaging. If these were not ordered at the last visit, perhaps the patient went to the ER due to growing concern at which point these investigations would have been ordered.</td><td>This is a regular follow-up to check on pain control and changes in symptoms.</td></tr>
<tr><th colspan="3">Presenting complaint</th></tr>
<tr><td>Joint pain in fingers and feet</td><td>Follow-up for persistent joint pain</td><td>Follow-up for pain control of rheumatoid arthritis.</td></tr>
<tr><th colspan="3">Initial presentation</th></tr>
<tr><td>"I've been having this joint pain in my fingers and feet for the past 4 months.”</td><td>"I'm back to discuss my joint pain. It feels like it’s getting worse.”</td><td>"The medications and lifestyle changes seem to be helping a little bit.”</td></tr>
</table>

**Context**		
Oluwassen has been experiencing joint pain in her fingers and feet for the past 4 months.	Oluwassen returns for a follow-up appointment. She continues to experience joint pain.	Oluwassen returns for a follow-up appointment. She has been taking the prescribed medications and trying lifestyle changes as recommended which is somewhat helping.
**Information to be shared to the doctor if the patient is explicitly asked**		
Symptoms: "The pain started 4 months ago. Initially, it was mild and intermittent, but over time, it has become more severe and persistent. The pain is sharp and sometimes throbbing. I would rate it a 6/10. The pain is worse in the morning and after periods of little activity. Sometimes, I also notice some swelling and stiffness, often in the mornings. The pain usually lasts for several hours and sometimes for the entire day. It's strange, it's sometimes painful to even touch. I'd say it's mostly in the joints that connect my hands to my fingers and my big toe joints. I haven't noticed any specific triggers, but it seems to be worse when I'm stressed or tired. I don't have any recent history of trauma to my fingers or feet. I haven't experienced this type of pain before."<br><br>Other: "I'm still working, but it's becoming increasingly difficult to use my hands for	Symptoms: "I've been trying to keep a pain diary as you suggested. The joint pain is now occurring daily, and I would rate the pain as 8/10. I'm taking the ibuprofen you recommended, but it doesn't seem to help much. I've noticed that I get stiff and swollen fingers and feet in the morning, which lasts for a few hours. I still don't have any vision changes, skin rashes, or other new symptoms. The pain is really impacting my ability to work and enjoy my daily activities. I am still working but finding it harder to meet deadlines because I'm typing so slow."<br><br>Lifestyle: "I work as an accountant, so I spend a lot of time sitting and typing. I try to stay active by going to dance classes but its getting harder because of the pain and feeling tired. My diet is generally balanced, I make my foods at home."	Symptoms: "My joint pain is occurring a little less but it is still there. I'm getting more sleep and have adjusted my work schedule to reduce stress. I would now rate the pain 6/10. The stiffness and swelling have also reduced but are still there. I don't have any new symptoms."<br><br>Other: "I will be seeing the rheumatologist next week."<br><br>Lifestyle: "I work as an accountant, so I spend a lot of time sitting and typing. I try to stay active by going to dance classes but its getting harder because of the pain and feeling tired. My diet is generally balanced, I make my foods at home."<br><br>Family History: "My mother had arthritis, but I'm not sure what type."<br><br>Past Medical and drug history: "I've been healthy overall with no major

detailed tasks. I'm worried about my ability to continue working if this gets worse."<br><br>Lifestyle: "I work as an accountant, so I spend a lot of time sitting and typing. I try to stay active by going to dance classes but its getting harder because of the pain and feeling tired. My diet is generally balanced, I make my foods at home."<br><br>Family History: "My mother had arthritis, but I'm not sure what type."<br><br>Past Medical and drug history: "I've been healthy overall with no major illnesses. I occasionally take ibuprofen for the pain."<br><br>Social History: "I smoke, I have smoked for the past 30 years and smoke about 10 cigarettes a day. I don't use recreational drugs. I drink alcohol socially only. I live with my husband, and we have a very supportive relationship. We have a daughter who is in university."<br><br>Height: "I am 1.75m tall."<br><br>Weight: "I weigh 75 kilograms."<br><br>Heart rate: "My tracker says it is 72 beats per minute."	Family History: "My mother had arthritis, but I'm not sure what type."<br><br>Past Medical and drug history: "I've been healthy overall with no major illnesses. I occasionally take ibuprofen for the pain."<br><br>Social History: "I smoke, I have smoked for the past 30 years and smoke about 10 cigarettes a day. I don't use recreational drugs. I drink alcohol socially only. I live with my husband, and we have a very supportive relationship. We have a daughter who is in university."<br><br>Height: "I am 1.75m tall."<br><br>Weight: "I weigh 75 kilograms."<br><br>Heart rate: "My tracker says it is 72 beats per minute."<br><br>Respiratory rate: "I'm measuring it to be 14 per minute."<br><br>Blood pressure: "It was normal the last time I checked at the pharmacy."<br><br>Temperature: "My thermometer says 36.5 degrees Celsius."	illnesses. I occasionally take ibuprofen for the pain."<br><br>Social History: "I smoke, I have smoked for the past 30 years and smoke about 10 cigarettes a day. I don't use recreational drugs. I drink alcohol socially only. I live with my husband, and we have a very supportive relationship. We have a daughter who is in university."<br><br>Height: "I am 1.75m tall."<br><br>Weight: "I weigh 75 kilograms."<br><br>Heart rate: "My tracker says it is 68 beats per minute."<br><br>Respiratory rate: "I'm measuring it to be 12 per minute."<br><br>Blood pressure: "It was normal the last time I checked at the pharmacy."<br><br>Temperature: "My thermometer says 36.5 degrees Celsius."

Respiratory rate: "I'm measuring it to be 14 per minute."<br><br>Blood pressure: "When I checked at the pharmacy 2 days ago, it said 122/80."<br><br>Temperature: "My thermometer says 36.5 degrees Celsius."		
**Past medical history and surgical history**		
N/A	N/A	N/A
**Current medications**		
Ibuprofen PRN for pain	Ibuprofen 400mg q6h PRN for pain	Methotrexate 7.5mg weekly<br>Prednisone 2.5mg daily<br>Ibuprofen 600mg q6h PRN for pain
**Relevant previous medications**		
N/A	N/A	N/A
**Allergies and adverse reactions**		
None	None	None
**Patient concerns, expectations and wishes**		
Concerns: "I'm worried about this joint pain. I hope it's not something serious."<br><br>Expectations: "I want to find out what's causing this pain."<br><br>Wishes: "I hope to get back to my normal life, I don't want this pain to limit my life."	Concerns: "I'm worried about this joint pain getting worse and affecting my life even more."<br><br>Expectations: "I want to find a treatment that works to manage this pain better."<br><br>Wishes: "I hope to get back to my normal life without this debilitating pain."	Concerns: "I'm concerned about any potential long-term complications of having rheumatoid arthritis."<br><br>Expectations: "I want to understand what else can be done to completely manage my symptoms."

		Wishes: "I hope to be disease-free and not have to deal with pain daily."
**Questions the patient wants to ask the doctor**		
"What could be causing this joint pain?"<br><br>"Do I need any tests?"<br><br>"What can I do to manage the pain and prevent it from getting worse?"	"What other treatments can I try?"<br><br>"Do I need more tests?"<br><br>"Can this condition lead to other health problems?"<br><br>“How long will it be before I can see the specialist?”	"What other treatments and cures are available if the severity of the pain comes back?"<br><br>"Should I get any additional tests or imaging to be on the safe side?"<br><br>"Can this condition lead to other complications?"

Visit 1

<table>
<tr><th>AMIE</th><th>PCP</th></tr>
<tr><th colspan="2">Dialogue with Patient</th></tr>
<tr><td colspan="2">[System Prompt]

Information available at the beginning of the visit

Patient: Oluwassen Odozor, 53 years, Female

Context: Oluwassen has been experiencing joint pain in her fingers and feet for the past 4 months.

Additional information: N/A</td></tr>
<tr><td>[Patient]

Hi, Doctor. I've been having this joint pain in my fingers and feet for the past 4 months.

[Doctor]

I'm sorry to hear you've been experiencing joint pain for four months. To help me understand better, could you describe the pain? 1. Where exactly is the pain located in your fingers and feet? 2. How would you describe the pain (e.g., sharp, aching, throbbing)?

[Patient]

I’d say it’s mostly in the joints that connect my hands to my fingers and my big toe joints. The pain is sharp and sometimes throbbing.

[Doctor]

Thanks for the details. Does the pain come and go, or is it constant? What makes it better or worse (e.g., rest, activity, position)?</td><td>[Patient]

Hi Doctor. I've been having this joint pain in my fingers and feet for the past 4 months.

[Doctor]

I will try to help. Have you had any other symptoms? Trouble with bowels? Fevers? Rashes?

[Patient]

No, I haven

[Doctor]

Do you have a family history of rheumatoid arthritis or lupus? Do you have any other medical problems?

[Patient]

My mother had arthritis but I'm not sure what type.</td></tr>
</table>

[Patient]

It used to be mild and intermittent, but over time, it has become more severe and persistent. It seems to be worse when I'm stressed or tired.

[Doctor]

I understand. Have you noticed any other symptoms with the joint pain, such as stiffness, swelling, redness, or skin changes in those areas? Also, have you had a fever or chills recently?

[Patient]

Sometimes, I also notice some swelling and stiffness, often in the mornings. The pain usually lasts for several hours and sometimes for the entire day. It's strange, it's sometimes painful to even touch. I don't have any fever or chills.

[Doctor]

Thank you for sharing that. The swelling and stiffness are helpful details. Has anyone in your family experienced similar joint pain or autoimmune conditions like rheumatoid arthritis or lupus?

[Patient]

My mother had arthritis, but I'm not sure what type. I I want to find out what's causing this pain. I hope it's not something serious.

[Doctor]

It's understandable to be concerned about your pain. Given your mother's history of arthritis and these symptoms, we need to

[Doctor]

I would like to investigate you for rheumatoid arthritis. We will do: 1) Physical exam 2) Blood tests: CBC, ESR, CRP, RF - rheumatoid factor, anti-CCP antibody 3) X-rays of hands

[Patient]

What could be causing this joint pain and what can I do to manage the pain and prevent it from getting worse? I'm worried about this joint pain. I hope it's not something serious.

[Doctor]

This can be many things. This includes 1) Rheumatoid arthritis. 2) osteoarthritis 3) Lupus (SLE) 4) Psoriatic arthritis 5) Vasculitis

[Patient]

Ok. I would eventually like to know what's going and get back to my normal life. I don't want this pain to limit my life.

[Doctor]

We need to make a diagnosis. Then, you should have good relief with the appropriate treatment. For now we can prescribe pain medication. Are you currently taking any medication? Do you have any other medical problems such as kidney problems or stomach ulcers? Are you allergic to any medication?

[Patient]

I only take ibuprofen for pain right now and nothing else. I'm not allergic to anything and I don't have any other health issues.

investigate further. To help me guide you to the right care, could you tell me:

1. Do you currently take any medications?
2. What is your occupation and how physically demanding is it?
3. Would you be willing to see a rheumatologist for further evaluation?

[Patient]

I occasionally take ibuprofen for the pain. I work as an accountant, so I spend a lot of time sitting and typing. I try to stay active by going to dance classes but its getting harder because of the pain and feeling tired. Yes, I'd be willing to see a rheumatologist. Do I need any tests and what could be causing this joint pain?

[Doctor]

Thanks for sharing that. It's great that you're staying active, even though it's become more challenging lately. Based on what you've described, conditions like rheumatoid arthritis or other inflammatory arthritides are possibilities. A rheumatologist can perform a more thorough evaluation and determine what tests are needed. I'll send you a referral to a rheumatologist; does that sound like a plan?

[Patient]

Yes, that sounds like a good plan. What can I do to manage the pain and prevent it from getting worse?

[Doctor]

It's great you're proactive! Continue taking ibuprofen for pain relief as needed, but limit the dose and duration to minimize potential

[Doctor]

You can continue taking ibuprofen for now. You can take acetaminophen (paracetamol) in addition to that for added pain relief. Further medication may be prescribed after we get results of blood tests and Xrays. We may also send you to a rheumatologist, based on the results.

[Patient]

Ok.

[Doctor]

Do you have any other questions for me today?

[Patient]

No, that's all for now.

[Doctor]

Thank you. Have a good day.

<table>
<tr><td>gastrointestinal side effects. I'll send you information about inflammatory arthritis and the importance of early diagnosis and treatment. We'll schedule a follow-up appointment in a couple of weeks to review test results and discuss the rheumatologist's findings. Does that sound good?

[Patient]

Yes, that all sounds great. Thank you, Doctor.

[Doctor]

You're welcome! I'll send the referral, educational materials, and instructions for blood work and X-rays shortly. Let me know if you have any questions.

[Patient]

Ok, great. I don't have any other questions at this point but I'll let you know, thanks.

[Doctor]

You're welcome! I'll send the referral and information shortly. Take care and I hope you feel better soon!</td><td></td></tr>
<tr><td colspan="2">Responses to Post-Questionnaire</td></tr>
<tr><td colspan="2">Guidelines Selected as Relevant</td></tr>
<tr><td>• [bmj105] Rheumatoid arthritis
• [ng100] Rheumatoid arthritis in adults: management
• [qs33] Rheumatoid arthritis in over 16s: quality standard</td><td>• [bmj105] Rheumatoid arthritis
• [ng100] Rheumatoid arthritis in adults: management
• [qs33] Rheumatoid arthritis in over 16s: quality standard</td></tr>
<tr><td colspan="2">Differential Diagnosis</td></tr>
<tr><td>1. Rheumatoid arthritis
2. Osteoarthritis
3. Psoriatic arthritis
4. Gout</td><td>1. Rheumatoid arthritis
2. LUPUS (SLE)
3. Psoriatic Arthritis
4. Viral arthritis</td></tr>
</table>

	5. Vasculitis<br>6. Osteoarthritis
In-visit Investigations	
None	1. Temp<br>2. Heart Rate<br>3. Blood Pressure<br>4. Physical Exam: Musculoskeletal
Ordered Investigations	
1. Refer Oluwassen to a rheumatologist for comprehensive evaluation and management of suspected inflammatory arthritis (likely RA). This referral should be expedited given the four-month duration and progressive nature of her symptoms. The referral should include all relevant clinical information, including her family history and current medication use.<br><br>2. Order blood tests: Complete Blood Count (CBC), Erythrocyte Sedimentation Rate (ESR), C-reactive protein (CRP), Rheumatoid Factor (RF), Anti-citrullinated peptide antibodies (ACPA).<br><br>3. Order hand and foot X-rays to assess for joint erosions or damage.	1. CBC<br>2. ESR<br>3. CRP<br>4. RF<br>5. ANTI-CCP<br>6. Liver enzymes<br>7. Creatinine and Urea<br>8. Uric Acid<br>9. Xrays: Hands and Feet
Recommendations or Actions	
1. Advise Oluwassen to continue taking ibuprofen for pain relief as needed, but to limit the dosage and duration of use to minimize potential gastrointestinal side effects. Provide her with information on safe ibuprofen use, including recommended dosages and potential side effects. Suggest she consider paracetamol as an alternative if ibuprofen causes any gastrointestinal upset.<br><br>2. Provide Oluwassen with educational materials on inflammatory arthritis, focusing	1. Based on results: Analgesics, NSAIDS, Immunosuppressive therapy/biologics if this an autoimmune rheum condition.<br><br>2. Referral to rheumatologist

on rheumatoid arthritis, including information on early diagnosis, treatment, potential long-term consequences if left untreated, and resources for patient support and information (e.g., links to the National Rheumatoid Arthritis Society).<br><br>3. Schedule a follow-up appointment in 2-4 weeks to review test results and discuss the rheumatologist's findings and recommendations. This follow-up will also allow for a discussion of the patient's response to ibuprofen and any potential need for adjustments to her pain management strategy.<br><br>4. Counsel Oluwassen on the importance of maintaining a healthy lifestyle, including regular, moderate, low-impact exercise (modifying dance classes as needed), stress management techniques, and a balanced diet to support overall health and well-being.	
Did you intentionally deviate from one of the clinical guidelines you selected above?	
No	No
If yes, please explain why (e.g. guideline does not cover this clinical scenario, patient preference, your preference). If no, enter 'N/A':	
N/A	N/A
What are other acceptable management plans? Why did you choose yours?	
While the primary management plan focuses on expediting referral to a rheumatologist for a comprehensive evaluation, alternative approaches could involve a more extensive initial workup in primary care before referral. However, given the progressive nature of Oluwassen's symptoms (4 months duration) and the potential for irreversible joint damage in RA, prioritizing rapid specialist consultation is deemed the most appropriate and effective strategy. A more conservative approach might delay diagnosis and treatment, potentially leading to worse long-term outcomes. The	Rheumatoid Arthritis Canada Guidelines. All are similar.

guidelines themselves emphasize the importance of early intervention in RA.	
Please describe any competing priorities that affected your decisions:	
N/A	N/A
Describe the cost, effectiveness and side effects of your management plan:	
The cost of the management plan includes the cost of the rheumatology referral, blood tests (CBC, ESR, CRP, RF, ACPA), hand and foot X-rays, and the follow-up appointment. The effectiveness is expected to be high due to the early intervention and comprehensive evaluation by a specialist. Potential side effects are primarily related to ibuprofen use (gastrointestinal issues), which are mitigated by advising Oluwassen on safe usage and suggesting paracetamol as an alternative. The rheumatologist will address potential side effects of any further medications prescribed.	These are the typical low cost investigations to work up polyarthritis.
Describe the prognosis with and without treatment of this patient:	
Without treatment, Oluwassen's prognosis is uncertain but potentially poor. Untreated inflammatory arthritis, particularly RA, can lead to progressive joint damage, significant functional limitations, and reduced quality of life. Early diagnosis and treatment are crucial for improving long-term outcomes. With appropriate treatment guided by the rheumatologist, the prognosis is significantly improved, aiming for remission or low disease activity to minimize joint damage and maximize functional ability and quality of life.	Likely slowly progressive causing pain and disability
Does this case require escalation of this text-based consultation to a video-call or in-person consultation?	
No, does not require escalation	No, does not require escalation
Describe why it requires or does not require escalation:	
The current text-based consultation is sufficient to initiate the appropriate management plan. The referral to the rheumatologist, along with the ordered investigations and advice provided, addresses the immediate needs of the	Does not at this point, as is non urgent condition, however a full physical exam would be useful after X-rays and blood tests are resulted.

patient. A video or in-person consultation is not necessary at this stage, as the rheumatologist will conduct a thorough evaluation and provide a tailored treatment plan.	
Is a follow-up required?	
Yes	Yes
If yes, when would you like to follow-up the patient? Otherwise, enter 'N/A':	
2-4 weeks	After bloodwork and radiograph results are available.
Describe why it requires or does not require follow-up:	
A follow-up appointment is necessary to review the results of the ordered investigations (blood tests and X-rays), discuss the rheumatologist's findings and recommendations, and assess Oluwassen's response to the initial management plan (ibuprofen use and lifestyle advice). This allows for timely adjustments to her treatment and support as needed.	Broad differential diagnosis for progressive disease causing pain and disability.

Specialist Physician Ratings

		AMIE	PCP
**Diagnosis & Management**	**Confabulation Absent (Y/N)**	Yes	Yes
	**DDx Appropriateness**	Very favorable	Favorable
	**DDx Comprehensiveness (4-point scale)**	Very favorable	Favorable
	**Escalation Recommendation Appropriate (Y/N)**	Yes	No
	**Followup Recommendation Appropriate (Y/N)**	Yes	Yes
	**Guideline Entailment Overall**	Very favorable	Favorable
	**Investigation Aligned With Guidelines**	Favorable	Favorable
	**Investigation Appropriate Recommended (Y/N)**	Yes	Yes
	**Investigation Cites Guidelines**	Very favorable	Very unfavorable
	**Investigation Inappropriate Avoided (Y/N)**	Yes	No
	**Investigation Sufficiently Precise (Y/N)**	Yes	Yes
	**Management Aligned With Guidelines (3-point scale)**	Favorable	Favorable
	**Management Free of Clinically Significant Error (Y/N)**	Yes	Yes

	**Management Plan Appropriateness**	Favorable	Favorable
	**Remembering Important Information From Previous Visits (3-point scale)**	Neither favorable nor unfavorable	Neither favorable nor unfavorable
	**Treatment Aligned With Guidelines**	Very favorable	Favorable
	**Treatment Appropriate Recommended (Y/N)**	Yes	No
	**Treatment Cites Guidelines**	Very favorable	Very unfavorable
	**Treatment Inappropriate Avoided (Y/N)**	Yes	Yes
	**Treatment Sufficiently Precise (Y/N)**	Yes	No
**MXEKF**	**Acting As Patient Teacher And Salesperson**	Favorable	Neither favorable nor unfavorable
	**Communication And Shared Decision Making**	Favorable	Unfavorable
	**Contrasting\nand Selection**	Favorable	Neither favorable nor unfavorable
	**Illness Specific Knowledge**	Favorable	Neither favorable nor unfavorable
	**Managing Interplay Of Competing Priorities**	Neither favorable nor unfavorable	Unfavorable
	**Monitoring And Adjustment Of Management Plan**	Favorable	Neither favorable nor unfavorable

	**Organization Of The Clinical Encounter**	Favorable	Unfavorable
	**Prioritization Of Preferences Constraints And Values**	Favorable	Unfavorable
	**Prognostication**	Favorable	Neither favorable nor unfavorable
	**Relationship Fostering**	Favorable	Unfavorable
**PACES**	**Addressing Patient Concerns**	Favorable	Neither favorable nor unfavorable
	**Clinical Judgement**	Favorable	Neither favorable nor unfavorable
	**Differential Diagnosis**	Very favorable	Favorable
	**Eliciting Family History**	Favorable	Neither favorable nor unfavorable
	**Eliciting Medication History**	Favorable	Neither favorable nor unfavorable
	**Eliciting Past Medical History**	Neither favorable nor unfavorable	Unfavorable
	**Eliciting Presenting Complaint**	Favorable	Neither favorable nor unfavorable
	**Eliciting Systems Review**	Neither favorable nor unfavorable	Unfavorable
	**Explaining Relevant Clinical Information Accurately**	Favorable	Neither favorable nor unfavorable

	**Explaining Relevant Clinical Information Clearly**	Favorable	Neither favorable nor unfavorable
	**Explaining Relevant Clinical Information Comprehensively**	Favorable	Unfavorable
	**Explaining Relevant Clinical Information Professionally**	Favorable	Neither favorable nor unfavorable
	**Explaining Relevant Clinical Information With Structure**	Favorable	Neither favorable nor unfavorable
	**Maintaining Patient Welfare**	Very favorable	Neither favorable nor unfavorable
	**Showing Empathy**	Favorable	Very unfavorable
	**Understanding Patient Concerns**	Very favorable	Neither favorable nor unfavorable

Patient Actor Ratings

		AMIE	PCP
**GMCPQ**	**Appearing Honest And Trustworthy**	Cannot rate / Does not apply / Agent did not perform this	Cannot rate / Does not apply / Agent did not perform this
	**Assessing Medical Condition**	Very favorable	Favorable
	**Being Polite**	Very favorable	Favorable
	**Explaining Condition and Treatment**	Very favorable	Neither favorable nor unfavorable
	**Involving Patient in Treatment Decisions**	Very favorable	Neither favorable nor unfavorable
	**Listening to Patient**	Very favorable	Favorable
	**Making Patient Feel At Ease**	Very favorable	Neither favorable nor unfavorable
	**Patient Confident About Care Provided (Y/N)**	Yes	Yes
	**Patient Happy to Return In Future (Y/N)**	Yes	No
	**Patient Trusts Information is Confidential**	Cannot rate / Does not apply / Agent did not perform this	Cannot rate / Does not apply / Agent did not perform this
	**Providing Appropriate Treatment Plan**	Very favorable	Cannot rate / Does not apply / Agent did not perform this

**MXEKF**	**Acting As Patient Teacher And Salesperson**	Very favorable	Neither favorable nor unfavorable
	**Communication And Shared Decision Making**	Very favorable	Neither favorable nor unfavorable
	**Managing Interplay Of Competing Priorities**	Cannot rate / Does not apply / Agent did not perform this	Cannot rate / Does not apply / Agent did not perform this
	**Monitoring And Adjustment Of Management Plan**	Cannot rate / Does not apply / Agent did not perform this	Cannot rate / Does not apply / Agent did not perform this
	**Organization Of The Clinical Encounter**	Very favorable	Favorable
	**Prioritization Of Preferences Constraints And Values**	Favorable	Favorable
	**Relationship Fostering**	Very favorable	Neither favorable nor unfavorable
**PACES**	**Addressing Patient Concerns**	Very favorable	Neither favorable nor unfavorable
	**Maintaining Patient Welfare**	Very favorable	Favorable
	**Showing Empathy**	Favorable	Neither favorable nor unfavorable
	**Understanding Patient Concerns**	Neither favorable nor unfavorable	Neither favorable nor unfavorable

Visit 2

AMIE	PCP
**Dialogue with Patient**	

[System Prompt]

Information available at the beginning of the visit

Patient: Oluwassen Odozor, 53 years, Female

Context: Oluwassen returns for a follow-up appointment. She continues to experience joint pain.

Additional information:

X-Ray Hands and Feet

Examination Details:

Modality: X-ray

Views Taken:

Hands: PA (posteroanterior), oblique

Feet: AP (anteroposterior), lateral

Findings:

Hands:

Joints:

MCP (Metacarpophalangeal) Joints: Mild to moderate narrowing of joint spaces observed, particularly in the second and third MCP joints bilaterally.

PIP (Proximal Interphalangeal) Joints: Joint space narrowing with periarticular osteopenia. Marginal erosions noted at the PIP joints of the index and middle fingers bilaterally.

DIP (Distal Interphalangeal) Joints: Joint spaces appear relatively preserved.

Bone Structure:

Erosions: Presence of subchondral cysts and erosions, particularly in the ulnar styloid process and the heads of the metacarpals.

Soft Tissue: Mild soft tissue swelling noted around the affected joints.

Deformities: Early signs of ulnar deviation and swan-neck deformities in the fingers.

Feet:

Joints:

MTP (Metatarsophalangeal) Joints: Moderate narrowing of joint spaces observed, particularly in the first and second MTP joints bilaterally.

IP (Interphalangeal) Joints: Joint space narrowing with periarticular osteopenia and marginal erosions, especially in the big toes.

Bone Structure:

Erosions: Erosions present at the medial aspect of the first MTP joints and the lateral aspects of the fifth MTP joints.

Soft Tissue: Soft tissue swelling noted around the affected joints.

Deformities: Early signs of hallux valgus deformity (bunion formation) bilaterally.

Impression: The X-ray findings are suggestive of rheumatoid arthritis, characterized by:

Symmetric joint space narrowing

Marginal erosions

Periarticular osteopenia

Soft tissue swelling

These findings correlate with the clinical suspicion of rheumatoid arthritis. Further evaluation and correlation with clinical and laboratory findings are recommended for a comprehensive assessment.

Radiologist: Dr. Josef Faraji, MD

Ultrasound Hands and Feet

Examination Details:

Modality: Ultrasound

Areas Examined: Hands and Feet

Findings:

Hands:

Synovitis:

MCP Joints: Moderate synovial thickening and hyperemia (increased blood flow) observed in the second and third MCP joints bilaterally, consistent with active synovitis.

PIP Joints: Synovial hypertrophy with hyperemia noted in the PIP joints of the index and middle fingers bilaterally.

Erosions:

Small cortical breaks (erosions) detected at the second and third MCP joints and PIP joints of the index and middle fingers, consistent with marginal erosions seen on X-ray.

Tenosynovitis:

Mild tenosynovitis of the extensor tendons noted, particularly in the wrist and MCP regions.

Power Doppler:

Increased power Doppler signal in the MCP and PIP joints, indicating active inflammation.

Feet:

Synovitis:

MTP Joints: Moderate synovial thickening and hyperemia observed in the first and second MTP joints bilaterally, consistent with active synovitis.

IP Joints: Synovial hypertrophy with hyperemia noted in the interphalangeal joints of the big toes.

Erosions:

Erosions identified at the medial aspect of the first MTP joints and the lateral aspects of the fifth MTP joints, correlating with the X-ray findings.

Tenosynovitis:

Mild tenosynovitis of the flexor tendons noted, particularly in the region of the first MTP joints.

Power Doppler:

Increased power Doppler signal in the MTP and IP joints, indicating active inflammation.

Impression: The ultrasound findings are consistent with the X-ray findings and suggest active rheumatoid arthritis. Key features include:

Active synovitis with synovial thickening and hyperemia in both hands and feet.

Marginal erosions at multiple joints.

Tenosynovitis and increased power Doppler signals indicating ongoing inflammation.

These ultrasound findings support the clinical diagnosis of rheumatoid arthritis and correlate well with the radiographic findings. Further clinical correlation and ongoing rheumatologic management are recommended.

Radiologist: Dr. Josef Faraji, MD

Bloodwork

Complete Blood Count (CBC) with Differential

White Blood Cell Count (WBC): 8.5 x 10^9/L (Reference Range: 4.0-11.0 x 10^9/L)

Hemoglobin (Hgb): 125 g/L (Reference Range: 120-160 g/L for females, 135-175 g/L for males)

Hematocrit (Hct): 0.38 L/L (Reference Range: 0.35-0.47 L/L for females, 0.40-0.52 L/L for males)

Platelets (Plt): 300 x 10^9/L (Reference Range: 150-400 x 10^9/L)

Mean Corpuscular Volume (MCV): 88 fL (Reference Range: 80-100 fL)

Mean Corpuscular Hemoglobin (MCH): 29 pg (Reference Range: 27-33 pg)

Mean Corpuscular Hemoglobin Concentration (MCHC): 330 g/L (Reference Range: 320-360 g/L)

Red Cell Distribution Width (RDW): 13.5% (Reference Range: 11.5-14.5%)

Neutrophils: 4.5 x 10^9/L (Reference Range: 2.0-7.5 x 10^9/L)

Lymphocytes: 2.5 x 10^9/L (Reference Range: 1.0-3.5 x 10^9/L)

Monocytes: 0.5 x 10^9/L (Reference Range: 0.2-0.8 x 10^9/L)

Eosinophils: 0.25 x 10^9/L (Reference Range: 0.04-0.4 x 10^9/L)

Basophils: 0.05 x 10^9/L (Reference Range: 0.01-0.1 x 10^9/L)

Rheumatoid Factor (RF)

Result: 40 IU/mL

Reference Range: < 14 IU/mL

Interpretation: Elevated

Anti-Cyclic Citrullinated Peptide (Anti-CCP) Antibodies

Result: 120 U/mL

Reference Range: < 20 U/mL

Interpretation: Elevated

Erythrocyte Sedimentation Rate (ESR)

Result: 35 mm/hr

Reference Range:

Males: 0-15 mm/hr

Females: 0-20 mm/hr

Interpretation: Elevated

C-Reactive Protein (CRP)

Result: 25 mg/L

Reference Range: < 5 mg/L

Interpretation: Elevated

[Patient]<br><br>Hi, Doctor. I'm back to discuss my joint pain. It feels like it’s getting worse.<br><br>[Doctor]<br><br>I'm glad to see you again, Oluwassen. I'm sorry to hear your pain is worsening. Your X-rays, ultrasound, and blood tests all strongly suggest rheumatoid arthritis. The joint damage is evident, and your blood markers—rheumatoid factor, anti-CCP antibodies, ESR, and CRP—are all significantly elevated, indicating active inflammation.<br><br>This is a lot to process, so please ask any questions you have. Then, we can discuss how this is affecting your daily activities, such as work and dancing, and create a plan to manage your pain and protect your joints. Does this make sense?<br><br>[Patient]<br><br>Yes, it does. Thank you for being so understanding about how this is a lot for me to take in. Can this condition lead to other health problems? How long will it be before I can see the specialist?<br><br>[Doctor]<br><br>You're welcome. It's understandable that this is a lot to take in.<br><br>Yes, rheumatoid arthritis can lead to other health problems if left untreated, such as joint damage, deformity, and potentially affecting other organs. Early diagnosis and treatment are key to minimizing these risks.	[Patient]<br><br>Hi Doctor. I'm back to discuss my joint pain. It feels like it’s getting worse.<br><br>[Doctor]<br><br>Blood tests abd X-ray findings are suggestive of rheumatoid arthritis<br><br>[Patient]<br><br>Can this condition lead to other health problems? I'm worried about this joint pain getting worse and affecting my life even more.<br><br>[Doctor]<br><br>Yes it can progress to other problems. I am going to 1) refer you to a rheumatologist 2) Start you on medication. First class of medication is referred to as DMARD (i.e., hydroxychloroquine, sulfasalazine, methotrexate, or leflunomide).<br><br>[Patient]<br><br>How long will it be before I can see the specialist? Do I need more tests?<br><br>[Doctor]<br><br>Careful monitoring of disease activity and adverse effects related to multiple medication use is essential. We will order the following laboratory tests: Hepatitis B and C status, purified protein derivative (PPD), full blood count (FBC), and liver function tests (LFTs) need to be checked before starting disease-modifying antirheumatic drugs (DMARDs). Laboratory monitoring for FBC and LFT abnormalities is done every 4-8 weeks at the start of treatment. When the

I've already sent your rheumatology referral. I'll follow up with their office to expedite your appointment. Expect a scheduling call within the next few weeks.

In the meantime, let's focus on managing your pain. How is the pain currently impacting your work and dancing? Are you experiencing pain in any other areas besides your hands and feet? Have you noticed any fatigue lately?

[Patient]

It's the same as before in the same areas that I mentioned before but just more painful. I'm still able to work and dance but it's harder than it was before.

[Doctor]

Okay, thank you for sharing that. It's good that you're still able to work and dance, even if it's more challenging now. Given the severity of your symptoms and test results, we need to discuss more aggressive treatment to control the inflammation and slow disease progression. I want to discuss treatment options, including disease-modifying antirheumatic drugs (DMARDs) and pain relievers. Before we do, could you rate your pain on a scale of 1 to 10 (1 being mild, 10 being the worst pain imaginable)? What makes your pain worse or better? Are there specific activities that trigger or worsen your pain?

[Patient]

The joint pain is now occurring daily, and I would rate the pain as 8/10. Like i mentioned, I am still working but finding it harder to meet deadlines because I’m typing so slow. The

patient is on a stable dose, they should be checked every 3-4 months.

[Patient]

Ok, thanks.

[Doctor]

Any other questions today?

[Patient]

No, that's all for now.

[Doctor]

Thank you. Goodbye.

only thing that makes it worse, i'd say, is when I'm stressed or tired.<br><br>[Doctor]<br><br>Your pain is significant and affecting your work, I understand. Given the X-ray, ultrasound, and blood test results confirming rheumatoid arthritis and active inflammation, I recommend starting methotrexate, a common and effective DMARD. It takes several weeks to see full benefit; we'll monitor your response. We can also address pain and inflammation with other medications like corticosteroids or biologics. Does this sound like a good plan? Do you have any questions about methotrexate or other treatment options?<br><br>[Patient]<br><br>Yes, this sounds like a good plan. Have we completed all the tests I need or are there anymore tests? I want to find a treatment that works to manage this pain better. I hope to get back to my normal life without this debilitating pain.<br><br>[Doctor]<br><br>It's great you're motivated to find a treatment that works! We've completed a thorough initial workup. We may need additional tests depending on your response to methotrexate. For example, if it's not effective, we might consider a biologic, which would require testing for infections like tuberculosis beforehand. We'll also monitor your liver function and blood counts regularly while you're on methotrexate. I'm optimistic we can find a good treatment plan for you.<br><br>To finalize your treatment plan, please tell me:	

1. Do you have any allergies to medications?<br>2. Are you taking any other medications besides ibuprofen?<br>3. Do you have any other health conditions besides joint pain?<br><br>Once I have this information, we can proceed. I'll also provide educational materials about rheumatoid arthritis and methotrexate. Does that sound good?<br><br>[Patient]<br><br>Yes, that sounds great. I don't have any allergies to any medications. I'm not taking anything other than ibuprofen and I have no other health conditions.<br><br>[Doctor]<br><br>Okay, I appreciate you sharing that information. It's good to know you don't have any allergies or other health conditions that might complicate treatment.<br><br>Given your test results, I'm prescribing methotrexate. I'm also referring you to a physical therapist specializing in rheumatoid arthritis. They'll teach you exercises to improve joint mobility and strength, helping manage pain and prevent further damage.<br><br>We'll schedule a four-week follow-up to monitor your response to methotrexate and adjust the treatment plan if needed.<br><br>Remember to:<br><br>● Take methotrexate as prescribed.<br>● Monitor for side effects like nausea, vomiting, fatigue, or rash. Contact me immediately if you experience anything unusual.	

• Attend physical therapy appointments. Regular exercise is crucial.<br>• Manage stress and fatigue, as these can worsen symptoms.<br><br>I'm confident that with appropriate treatment and self-care, you'll regain your normal life and enjoy work and dancing again. Does this sound okay? Any questions?<br><br>[Patient]<br><br>Yes, this all sounds great. No, I don't have any other questions right now. Thank you for your help.<br><br>[Doctor]<br><br>You're very welcome! I'm glad we could create a plan you're comfortable with. Please reach out if any questions or concerns arise before your next appointment. I look forward to seeing you in four weeks!<br><br>[Patient]<br><br>Great, I will. I look forward to seeing you as well. Take care.<br><br>[Doctor]<br><br>You're welcome! I'm glad we could create a plan you're comfortable with. Please don't hesitate to contact me if any questions or concerns arise before your next appointment. I look forward to seeing you in four weeks!	
**Responses to Post-Questionnaire**	
Guidelines Selected as Relevant	
• [bmj105] Rheumatoid arthritis<br>• [ng100] Rheumatoid arthritis in adults: management	• [bmj105] Rheumatoid arthritis<br>• [ng100] Rheumatoid arthritis in adults: management

• [qs33] Rheumatoid arthritis in over 16s: quality standard	• [qs33] Rheumatoid arthritis in over 16s: quality standard
Differential Diagnosis	
1. Rheumatoid arthritis<br>2. Psoriatic arthritis<br>3. Osteoarthritis<br>4. Lupus (Systemic lupus erythematosus)<br>5. Reactive arthritis	1. Rheumatoid arthritis<br>2. Psoriatic arthritis<br>3. Lupus<br>4. Osteoarthritis<br>5. Viral arthritis
In-visit Investigations	
None	1. Hepatitis B and C status,<br>2. Purified protein derivative (PPD)<br>3. CBC<br>4. Liver function tests (LFTs)
Ordered Investigations	
1. Prescribe Methotrexate 7.5mg orally once weekly, increasing the dose gradually (e.g., 2.5mg increments every 1-2 weeks) based on response and tolerability, to a maximum of 25mg weekly. Instruct the patient to take folic acid 5mg once weekly, starting concurrently with methotrexate, to reduce gastrointestinal side effects.<br><br>2. Refer to a physical therapist specializing in rheumatoid arthritis for a tailored exercise program focusing on joint mobility, strengthening, and joint protection techniques.<br><br>3. Order Liver Function Tests (LFTs) including ALT, AST, ALP, and bilirubin, and a Complete Blood Count (CBC) to establish baseline values and monitor for methotrexate-induced hepatotoxicity and hematological side effects. Repeat these tests in 4 weeks and then every 4-6 weeks thereafter.	N/A
Recommendations or Actions	

1. Schedule a four-week follow-up appointment to assess the patient's response to methotrexate, review LFTs and CBC results, adjust the dosage as needed, and discuss any side effects or concerns. At this visit, we will also discuss the patient's progress with physical therapy and address any challenges she is facing.<br><br>2. Provide comprehensive patient education materials on rheumatoid arthritis, including disease progression, treatment goals, methotrexate's mechanism of action, potential side effects, and strategies for managing symptoms (pain, fatigue, stiffness). Emphasize the importance of medication adherence, recognizing early signs of flares, and promptly reporting any side effects. Provide information on support resources (e.g., National Rheumatoid Arthritis Society).<br><br>3. Advise the patient to discontinue ibuprofen use due to the initiation of methotrexate to minimize potential drug interactions and hepatotoxicity.<br><br>4. Counsel the patient on lifestyle modifications to manage stress and fatigue, which can exacerbate RA symptoms. This may include stress management techniques (yoga, meditation, CBT), regular low-impact exercise (modified dance classes), and a balanced diet.<br><br>5. Instruct the patient to report any signs of infection (fever, chills, sore throat, etc.) immediately due to the potential immunosuppressive effects of methotrexate.	1. Repeat blood tests every four weeks until patient and treatment regimen stable:<br>2. Hepatitis B and C status<br>3. purified protein derivative (PPD)<br>4. CBC<br>5. Liver function tests (LFTs)<br>6. May require higher combination of drugs or advanced tier of drugs (i.e. biologics) if not responsive. These would typically be under care of rheumatologist.<br>7. Rheumatologist consult.
Did you intentionally deviate from one of the clinical guidelines you selected above?	
No	No
If yes, please explain why (e.g. guideline does not cover this clinical scenario, patient	

preference, your preference). If no, enter 'N/A':	
N/A	N/A
What are other acceptable management plans? Why did you choose yours?	
While the chosen plan prioritizes rapid treatment initiation with methotrexate based on the strong evidence of active RA, alternative approaches could involve a slower step-up approach with other cDMARDs or a trial of other treatments before initiating methotrexate. However, given the significant joint damage already present (evident in the imaging and elevated inflammatory markers), the potential for irreversible damage, and the patient's desire for rapid pain relief, the aggressive approach with methotrexate is deemed the most appropriate and effective strategy to minimize long-term damage and improve quality of life. The guidelines emphasize the importance of early intervention and treat-to-target strategies in RA management.	Canadian Rheumatology Association Guidelines
Please describe any competing priorities that affected your decisions:	
N/A	N/A
Describe the cost, effectiveness and side effects of your management plan:	
The cost includes methotrexate prescription, physical therapy referral, and regular blood tests (LFTs and CBC). Effectiveness is expected to be high due to early intervention with a highly effective DMARD, combined with physical therapy for joint protection and symptom management. Side effects are a possibility with methotrexate (hepatotoxicity, hematological issues, gastrointestinal upset), but these are mitigated by careful monitoring (LFTs and CBC), folic acid supplementation, and close follow-up. The physical therapy will help manage pain and prevent further joint damage. The patient will be educated on recognizing and reporting side effects promptly.	First line drugs are generally inexpensive. Second line mediations (i.e. biologics) associated with greater cost but worthwhile in the context of a progressive disabling disease. These are extablished guidelines.
Describe the prognosis with and without treatment of this patient:	
Without treatment, Oluwassen's prognosis is	Chronic progressive condition: pain, disability

poor. Untreated RA leads to progressive joint destruction, severe functional limitations, and significant impact on quality of life. With the recommended treatment plan, the prognosis is significantly improved, aiming for remission or low disease activity to minimize joint damage, maximize functional ability, and improve quality of life.	that impacts ability to perform activities of daily living (ADL) and impacts employability.
Does this case require escalation of this text-based consultation to a video-call or in-person consultation?	
No, does not require escalation	Yes, requires escalation to an IN-PERSON consultation
Describe why it requires or does not require escalation:	
The current text-based consultation is sufficient to initiate the appropriate management plan. The prescription of methotrexate, referral to physical therapy, and ordered blood tests address the immediate needs of the patient. A video or in-person consultation is not necessary at this stage, as the close follow-up appointments will allow for monitoring of treatment response and adjustments as needed.	Physical exam (i.e. musculoskeletal exam) is central to diagnosis as well as monitoring response to disease.
Is a follow-up required?	
Yes	Yes
If yes, when would you like to follow-up the patient? Otherwise, enter 'N/A':	
4 weeks	Four weeks following start of medication
Describe why it requires or does not require follow-up:	
A follow-up appointment is crucial to monitor Oluwassen's response to methotrexate, review blood test results (LFTs and CBC), assess for side effects, adjust the methotrexate dosage as needed, and discuss her progress with physical therapy. This allows for timely adjustments to her treatment and support, ensuring optimal management of her rheumatoid arthritis.	Assess response to therapy and results of lab tests: adequacy of treatment, as well as potential adverse effects of meds

Specialist Physician Ratings

		AMIE	PCP
**Diagnosis & Management**	**Confabulation Absent (Y/N)**	Yes	Yes
	**DDx Appropriateness**	Very favorable	Favorable
	**DDx Comprehensiveness (4-point scale)**	Very favorable	Very favorable
	**Escalation Recommendation Appropriate (Y/N)**	Yes	Yes
	**Followup Recommendation Appropriate (Y/N)**	Yes	Yes
	**Guideline Entailment Overall**	Very favorable	Favorable
	**Investigation Aligned With Guidelines**	Very favorable	Neither favorable nor unfavorable
	**Investigation Appropriate Recommended (Y/N)**	Yes	No
	**Investigation Cites Guidelines**	Favorable	Very favorable
	**Investigation Inappropriate Avoided (Y/N)**	Yes	No
	**Investigation Sufficiently Precise (Y/N)**	Yes	Yes
	**Management Aligned With Guidelines (3-point scale)**	Favorable	Favorable
	**Management Free of Clinically Significant Error (Y/N)**	Yes	Yes
	**Management Plan Appropriateness**	Favorable	Favorable

	**Remembering Important Information From Previous Visits (3-point scale)**	Favorable	Favorable
	**Treatment Aligned With Guidelines**	Favorable	Neither favorable nor unfavorable
	**Treatment Appropriate Recommended (Y/N)**	No	No
	**Treatment Cites Guidelines**	Favorable	Very unfavorable
	**Treatment Inappropriate Avoided (Y/N)**	Yes	Yes
	**Treatment Sufficiently Precise (Y/N)**	Yes	No
**MXEKF**	**Acting As Patient Teacher And Salesperson**	Very favorable	Unfavorable
	**Communication And Shared Decision Making**	Favorable	Unfavorable
	**Contrasting\nand Selection**	Favorable	Neither favorable nor unfavorable
	**Illness Specific Knowledge**	Favorable	Unfavorable
	**Managing Interplay Of Competing Priorities**	Favorable	Unfavorable
	**Monitoring And Adjustment Of Management Plan**	Favorable	Neither favorable nor unfavorable
	**Organization Of The Clinical Encounter**	Favorable	Unfavorable
	**Prioritization Of Preferences Constraints And Values**	Favorable	Unfavorable
	**Prognostication**	Favorable	Unfavorable
	**Relationship Fostering**	Very favorable	Unfavorable

**PACES**	**Addressing Patient Concerns**	Very favorable	Unfavorable
	**Clinical Judgement**	Favorable	Neither favorable nor unfavorable
	**Differential Diagnosis**	Very favorable	Favorable
	**Eliciting Family History**	Cannot rate / Does not apply / Agent did not perform this	Cannot rate / Does not apply / Agent did not perform this
	**Eliciting Medication History**	Favorable	Cannot rate / Does not apply / Agent did not perform this
	**Eliciting Past Medical History**	Very favorable	Cannot rate / Does not apply / Agent did not perform this
	**Eliciting Presenting Complaint**	Very favorable	Unfavorable
	**Eliciting Systems Review**	Favorable	Cannot rate / Does not apply / Agent did not perform this
	**Explaining Relevant Clinical Information Accurately**	Very favorable	Neither favorable nor unfavorable
	**Explaining Relevant Clinical Information Clearly**	Favorable	Neither favorable nor unfavorable
	**Explaining Relevant Clinical Information Comprehensively**	Favorable	Very unfavorable
	**Explaining Relevant Clinical Information Professionally**	Very favorable	Unfavorable
	**Explaining Relevant Clinical Information With Structure**	Favorable	Unfavorable

	**Maintaining Patient Welfare**	Very favorable	Neither favorable nor unfavorable
	**Showing Empathy**	Favorable	Neither favorable nor unfavorable
	**Understanding Patient Concerns**	Very favorable	Neither favorable nor unfavorable

Patient Actor Ratings

		AMIE	PCP
**GMCPQ**	**Appearing Honest And Trustworthy**	Cannot rate / Does not apply / Agent did not perform this	Cannot rate / Does not apply / Agent did not perform this
	**Assessing Medical Condition**	Very favorable	Favorable
	**Being Polite**	Very favorable	Favorable
	**Explaining Condition and Treatment**	Very favorable	Neither favorable nor unfavorable
	**Involving Patient in Treatment Decisions**	Very favorable	Neither favorable nor unfavorable
	**Listening to Patient**	Very favorable	Favorable
	**Making Patient Feel At Ease**	Very favorable	Neither favorable nor unfavorable
	**Patient Confident About Care Provided (Y/N)**	Yes	Yes
	**Patient Happy to Return In Future (Y/N)**	Yes	No
	**Patient Trusts Information is Confidential**	Cannot rate / Does not apply / Agent did not perform this	Cannot rate / Does not apply / Agent did not perform this
	**Providing Appropriate Treatment Plan**	Very favorable	Favorable
**MXEKF**	**Acting As Patient Teacher And Salesperson**	Very favorable	Neither favorable nor unfavorable

	**Communication And Shared Decision Making**	Very favorable	Neither favorable nor unfavorable
	**Managing Interplay Of Competing Priorities**	Cannot rate / Does not apply / Agent did not perform this	Cannot rate / Does not apply / Agent did not perform this
	**Monitoring And Adjustment Of Management Plan**	Cannot rate / Does not apply / Agent did not perform this	Cannot rate / Does not apply / Agent did not perform this
	**Organization Of The Clinical Encounter**	Very favorable	Favorable
	**Prioritization Of Preferences Constraints And Values**	Very favorable	Cannot rate / Does not apply / Agent did not perform this
	**Relationship Fostering**	Very favorable	Neither favorable nor unfavorable
**PACES**	**Addressing Patient Concerns**	Very favorable	Favorable
	**Maintaining Patient Welfare**	Very favorable	Favorable
	**Showing Empathy**	Very favorable	Neither favorable nor unfavorable
	**Understanding Patient Concerns**	Very favorable	Neither favorable nor unfavorable

Visit 3

<table>
<tr><th>AMIE</th><th>PCP</th></tr>
<tr><td colspan="2">Dialogue with Patient</td></tr>
<tr><td colspan="2">[System Prompt]

Information available at the beginning of the visit

Patient: Oluwassen Odozor, 53 years, Female

Context: Oluwassen returns for a follow-up appointment. She has been taking the prescribed medications and trying lifestyle changes as recommended which is somewhat helping.

Additional information: N/A</td></tr>
<tr><td>[Patient]

Hi, Doctor. The medications and lifestyle changes seem to be helping a little bit.

[Doctor]

That's encouraging to hear you're experiencing some improvement! Can you describe how your pain levels have changed since our last visit (it was an 8/10 then)? Are you experiencing any side effects from the methotrexate?

[Patient]

"My joint pain is occurring a little less but it is still there. I’m getting more sleep and have adjusted my work schedule to reduce stress. I would now rate the pain 6/10. The stiffness and swelling have also reduced but are still there. I don’t have any new symptoms. No, I haven't experienced any side effects so far.

[Doctor]</td><td>[Patient]

Hi, Doctor. The medications and lifestyle changes seem to be helping a little bit.

[Doctor]

This is good news. How long have you been on the medications. Which ones are you taking now?

[Patient]

I started them just after I last saw you and you prescribed them for me. I'm taking Methotrexate 7.5mg weekly, Prednicone 2.5mg daily, and Ibuprofen 600mg whenever I need it for the pain.

[Doctor]

If you are stable now we will keep you on this dose. I need to recheck blood tests 1) CBC 2) Liver function tests every 4-8 weeks following the start of this this treatment. Once you are</td></tr>
</table>

It's good to hear you're feeling some improvement! That reduction in your pain from 8/10 to 6/10 is positive. The absence of side effects from the methotrexate is also encouraging. It's great that you are implementing lifestyle changes.<br><br>To better understand your progress, could you tell me:<br><br>1. How are you managing at work and with dancing now compared to before?<br>2. Are there specific activities that still trigger or worsen your joint pain?<br>3. Have you noticed any change in your ability to do daily tasks like dressing or cooking?<br><br>[Patient]<br><br>have adjusted my work schedule to reduce stress which has helped and I'm able to dance now with more ease when compared to before. I still feel the pain but it's much better. Stress was a big factor in making my symptoms worse so I'm trying to reduce it where I can. I'm able to do things now with less pain before and less difficulty.<br><br>[Doctor]<br><br>That's wonderful to hear you're seeing improvements in your work and dancing! It sounds like the combination of medication and lifestyle changes is making a positive impact. It's great that you've identified stress as a trigger and are actively working to manage it.<br><br>It's also encouraging that you're finding it easier to do daily tasks.	on a stable dose we can check blood every 3-4 months.<br><br>[Patient]<br><br>Ok, I will schedule my follow up appointments as soon as wer<br><br>[Doctor]<br><br>Do you have any other questions for me?<br><br>[Patient]<br><br>What other treatments and cures are available if the severity of the pain comes back and should I get any additional tests or imaging to be on the safe side?<br><br>[Doctor]<br><br>Multiple other treatments are available. I would refer to a rheumatologist. Treatments include biologic medications such as » etanercept: » infliximab: » adalimumab. We could also do Chet Xray and Xray of wrist. I need to recheck blood tests 1) CBC 2) Liver function tests every 4-8 weeks following the start of this this treatment. Once you are on a stable dose we can check blood every 3-4 months.<br><br>[Patient]<br><br>Ok. Do you think this condition might lead to other complications?<br><br>[Doctor]<br><br>It is possible. 1) The medication can affect your liver and bllod. That is why we repeat the blood tests. 2) With rheumatoid arthritis you are at risk of developing: work disability, needing increased joint replacement surgery,

To help me further assess your progress, could you tell me:<br><br>1. How long does the morning stiffness and swelling usually last?<br>2. Are there any specific times of day when your pain is worse?<br>3. Have you noticed any patterns in your pain, such as it being worse after certain activities or in certain weather conditions?<br><br>[Patient]<br><br>The stiffness and swelling used to last for several hours and it was painful to even touch but now It has reduced. It's there but it's tolerable now. The mornings and during stress were the worst times now its not as bad now. I haven't noticed any other patterns other than what I've mentioned.<br><br>[Doctor]<br><br>That's encouraging, Oluwassen. It sounds like you're making progress. To track your progress, please continue monitoring your pain (1-10 scale), note any triggers, and report any new symptoms or methotrexate side effects. Let's schedule another follow-up in three months. Any questions?<br><br>[Patient]<br><br>I'll do that, thank you! What other treatments and cures are available if the severity of the pain comes back? Should I get any additional tests or imaging to be on the safe side? I hope to be disease-free and not have to deal with pain daily.<br><br>[Doctor]	increased coronary artery disease, interstitial lung disease (ILD), increased mortality<br><br>[Patient]<br><br>I want to definitely stay on top of all my testing then. I don't want to have any complications.<br><br>[Doctor]<br><br>Good. Any other questions?<br><br>[Patient]<br><br>No, I don<br><br>[Doctor]<br><br>Ok thank you. See you in 4 weeks then.

It's great you're thinking ahead! I'm glad you're committed to managing your rheumatoid arthritis.<br><br>There isn't a cure for rheumatoid arthritis, but many treatments manage symptoms and prevent progression. If your pain significantly worsens, we can:<br><br>● Increase methotrexate dosage.<br>● Add another DMARD.<br>● Start a biologic (requires pre-treatment infection testing).<br>● Use corticosteroids (short-term).<br>● Explore pain management strategies (physical therapy, occupational therapy, medications).<br><br>We'll continue monitoring your bloodwork and may repeat imaging.<br><br>To personalize your plan, tell me:<br><br>1. What's your definition of 'disease-free'? What pain level and function would you consider acceptable?<br>2. What are your biggest concerns about living with RA?<br>3. What life aspects do you most want to improve with treatment?<br><br>I'm here to support you. Let's work together to help you live a full life. Does this address your questions?<br><br>[Patient]<br><br>Well I'd like to not worry about having any pain at all and not have RA. My biggest concern would probably be about any potential long-term complications of having rheumatoid arthritis. I just want to be able to live the way I did before I had all of this pain.	

[Doctor]<br><br>It's understandable to want to be pain-free and live as you did before. I admire your determination!<br><br>Unfortunately, there's no cure for rheumatoid arthritis, but with proactive management, we can significantly reduce your pain and prevent long-term complications. Let's discuss those complications and how to minimize them:<br><br>● **Joint damage:** RA can cause irreversible joint damage, leading to deformity and disability. Early diagnosis and treatment with DMARDs are crucial for joint protection. We'll closely monitor your progress and adjust your treatment as needed.<br>● **Systemic effects:** RA can sometimes affect other organs. We'll monitor your overall health and refer you to specialists if necessary.<br>● **Fatigue and other symptoms:** RA can cause fatigue and other symptoms affecting quality of life. We can address these with medication, lifestyle changes, and support groups.<br><br>Here's how we'll work towards your goals:<br><br>● Continue your current treatment: Methotrexate and lifestyle changes are a good start. We'll monitor your response and adjust treatment accordingly.<br>● Focus on self-care: Manage stress, sleep, diet, and exercise. These habits improve your symptoms and well-being.<br>● Consider additional therapies: Physical and occupational therapy,	

<table>
<tr><td>
and pain management strategies, can help.
Stay connected: Support groups can be helpful.
I'm committed to helping you. Let's schedule a three-month follow-up to assess your progress and discuss treatment adjustments. Do you have any other questions?
[Patient]
Thank you so much for your help and support. This is all new to me and I want to do everything to prevent it from getting worse and to feel better. I don't have any other questions right now.
[Doctor]
You're very welcome! It's great you're proactive and committed to your health. I'm here to support you. Remember to take your medications as prescribed, continue practicing the lifestyle changes we discussed, monitor your symptoms closely, and report any changes or concerns. Don't hesitate to reach out if you have questions or need support. I'm confident that with consistent effort and the right treatment, you can manage your rheumatoid arthritis and live a full and active life. I'll see you in three months for your follow-up! Take care.
</td><td></td></tr>
<tr><td colspan="2">Responses to Post-Questionnaire</td></tr>
<tr><td colspan="2">Guidelines Selected as Relevant</td></tr>
<tr><td>
[bmj105] Rheumatoid arthritis
[ng100] Rheumatoid arthritis in adults: management
[qs33] Rheumatoid arthritis in over 16s: quality standard
</td><td>
[bmj105] Rheumatoid arthritis
[ng100] Rheumatoid arthritis in adults: management
[qs33] Rheumatoid arthritis in over 16s: quality standard
</td></tr>
<tr><td colspan="2">Differential Diagnosis</td></tr>
</table>

1. Rheumatoid arthritis<br>2. Psoriatic arthritis<br>3. Osteoarthritis<br>4. Lupus (SLE)	1. Rheumatoid arthritis<br>2. Psoriatic arthritis<br>3. Lupus<br>4. Viral arthritis<br>5. Vasculitis<br>6. Osteoarthritis
In-visit Investigations	
None	1. CBC<br>2. Liver enzymes
Ordered Investigations	
1. Repeat Liver Function Tests (LFTs) including ALT, AST, ALP, and bilirubin, and a Complete Blood Count (CBC) to monitor for methotrexate-induced hepatotoxicity and hematological side effects.<br><br>2. Order Erythrocyte Sedimentation Rate (ESR) and C-reactive protein (CRP) to monitor inflammation.	1. CBC every 4-8 weeks until stable<br>2. Liver enzymes every 4-8 weeks until stable<br>3. Xray wrist<br>4. Chest x-ray
Recommendations or Actions	
1. Continue methotrexate 7.5mg weekly and folic acid 5mg weekly. Monitor for side effects such as nausea, vomiting, fatigue, mouth sores, or rash. Instruct patient to report any side effects immediately.<br><br>2. Continue to encourage commitment to lifestyle modifications to manage stress and improve sleep (stress reduction techniques, regular low-impact exercise, balanced diet).<br><br>3. Schedule a three-month follow-up appointment to assess the patient's response. At the three-month appointment, we will discuss possible treatment adjustments based on lab results and symptom response. Instruct the patient to document daily pain levels (1-10), note any triggers or patterns, and contact if pain returns to an 8/10 or higher.	1. Refer to a rheumatologist.<br>2. If symptoms progress, add biologic medications such as: etanercept, infliximab, adalimumab.

4. Discuss shared-decision making in relation to treating future exacerbations and/or progression. Develop a plan wherein the patient can recognize the increased severity of their symptoms which require escalation and communicate that to the clinician. Discuss realistic expectations regarding pain management in RA; complete remission is unlikely, but low disease activity with minimal pain and improved function is a realistic goal.<br><br>5. Educate the patient on potential long-term complications of RA (joint damage, systemic effects, fatigue, cardiovascular disease) and strategies to minimize these risks, such as medication adherence, early reporting of flares, lifestyle modifications, and regular follow-up appointments.<br><br>6. Provide educational materials about rheumatoid arthritis, methotrexate, and potential adverse events. Emphasize the importance of recognizing and reporting any signs of infection (fever, chills, sore throat) due to the immunosuppressive effects of methotrexate.<br><br>7. Reinforce the importance of regular physical therapy. If not improved, discuss referral again. Consider referral to occupational therapy to address challenges with daily activities.<br><br>8. Provide information on support resources, including patient support groups or online forums, to aid in navigating her condition and connect with other patients facing similar challenges.	
Did you intentionally deviate from one of the clinical guidelines you selected above?	
No	No

If yes, please explain why (e.g. guideline does not cover this clinical scenario, patient preference, your preference). If no, enter 'N/A':	
N/A	N/A
What are other acceptable management plans? Why did you choose yours?	
While the chosen plan continues methotrexate based on the patient's partial response and the absence of side effects, alternative approaches could include increasing the methotrexate dose more aggressively, adding a second cDMARD, or even considering a biologic DMARD if the response remains suboptimal. However, given the patient's reported improvement, the absence of side effects, and the need to avoid potential toxicity from rapid escalation, maintaining the current dose with close monitoring and a planned three-month follow-up is deemed the most prudent approach. This allows for a more gradual titration of the methotrexate dose, minimizing the risk of adverse events while still aiming for treatment targets. The guidelines emphasize the importance of shared decision-making and individualizing treatment plans based on patient response and tolerability.	Canadian rheumatology association guidelines.
Please describe any competing priorities that affected your decisions:	
N/A	N/A
Describe the cost, effectiveness and side effects of your management plan:	
The cost of the management plan includes the cost of methotrexate and folic acid prescriptions, regular blood tests (LFTs, CBC, ESR, CRP), and the follow-up appointment. The effectiveness is expected to be moderate to high, given the patient's reported improvement and the continued use of methotrexate, a highly effective DMARD. Potential side effects are a possibility with methotrexate (hepatotoxicity, hematological issues, gastrointestinal upset), but these are mitigated by careful monitoring (LFTs and CBC), folic acid supplementation, and close follow-up. The patient's proactive lifestyle modifications further enhance the	Highly effective considering high risk of progression to increased morbidity and disability impact ability to work.

effectiveness of the plan. The patient's reported improvement suggests that the current treatment strategy is effective and well-tolerated.	
Describe the prognosis with and without treatment of this patient:	
Without continued treatment, Oluwassen's prognosis is poor. Untreated RA leads to progressive joint destruction, severe functional limitations, and significant impact on quality of life. With the recommended treatment plan, which includes continued methotrexate, regular monitoring, and lifestyle modifications, the prognosis is significantly improved, aiming for low disease activity with minimal pain and improved function. The patient's proactive approach to self-management further enhances her prognosis.	With rheumatoid arthritis there is increased t risk of developing: work disability, needing increased joint replacement surgery, increased coronary artery disease, interstitial lung disease (ILD), Felty syndrome, liver disease secondary to methotrexate toxicity - increased mortality
Does this case require escalation of this text-based consultation to a video-call or in-person consultation?	
No, does not require escalation	Yes, requires escalation to a VIDEO-CALL consultation
Describe why it requires or does not require escalation:	
The current text-based consultation is sufficient to adjust the management plan based on the patient's progress. The continued methotrexate prescription, scheduled three-month follow-up, and recommendations for continued lifestyle modifications address the patient's immediate needs. A video or in-person consultation is not necessary at this stage, as the close follow-up appointments will allow for monitoring of treatment response and adjustments as needed.	Multi-system disease with progressive morbidity and mortality risk. Benefits from full physical exam.
Is a follow-up required?	
Yes	Yes
If yes, when would you like to follow-up the patient? Otherwise, enter 'N/A':	
3 months	Liver function tests every 4-8 weeks following the start of this treatment. Once on a stable dose we can check blood every 3-4 months.

Describe why it requires or does not require follow-up:	
A three-month follow-up is necessary to assess Oluwassen's response to the continued methotrexate therapy, review updated blood test results (LFTs, CBC, ESR, CRP), assess for any emerging side effects, and discuss any adjustments to the treatment plan based on her symptom response and progress with lifestyle modifications. This allows for timely adjustments to her treatment and support, ensuring optimal management of her rheumatoid arthritis.	Potential for poor response to medication, disease progression - Potential for drug toxicity - i.e. methotrexate-related liver disease.

Specialist Physician Ratings

		AMIE	PCP
**Diagnosis & Management**	**Confabulation Absent (Y/N)**	Yes	Yes
	**DDx Appropriateness**	Very favorable	Favorable
	**DDx Comprehensiveness (4-point scale)**	Very favorable	Favorable
	**Escalation Recommendation Appropriate (Y/N)**	Yes	Yes
	**Followup Recommendation Appropriate (Y/N)**	Yes	Yes
	**Guideline Entailment Overall**	Very favorable	Favorable
	**Investigation Aligned With Guidelines**	Favorable	Neither favorable nor unfavorable
	**Investigation Appropriate Recommended (Y/N)**	Yes	Yes
	**Investigation Cites Guidelines**	Favorable	Very favorable
	**Investigation Inappropriate Avoided (Y/N)**	Yes	No
	**Investigation Sufficiently Precise (Y/N)**	Yes	No
	**Management Aligned With Guidelines (3-point scale)**	Favorable	Favorable
	**Management Free of Clinically Significant Error (Y/N)**	Yes	Yes
	**Management Plan Appropriateness**	Favorable	Favorable

	**Remembering Important Information From Previous Visits (3-point scale)**	Favorable	Neither favorable nor unfavorable
	**Treatment Aligned With Guidelines**	Favorable	Neither favorable nor unfavorable
	**Treatment Appropriate Recommended (Y/N)**	Yes	No
	**Treatment Cites Guidelines**	Very favorable	Very unfavorable
	**Treatment Inappropriate Avoided (Y/N)**	Yes	Yes
	**Treatment Sufficiently Precise (Y/N)**	Yes	No
**MXEKF**	**Acting As Patient Teacher And Salesperson**	Very favorable	Unfavorable
	**Communication And Shared Decision Making**	Very favorable	Neither favorable nor unfavorable
	**Contrasting\nand Selection**	Favorable	Neither favorable nor unfavorable
	**Illness Specific Knowledge**	Favorable	Neither favorable nor unfavorable
	**Managing Interplay Of Competing Priorities**	Favorable	Neither favorable nor unfavorable
	**Monitoring And Adjustment Of Management Plan**	Favorable	Neither favorable nor unfavorable
	**Organization Of The Clinical Encounter**	Favorable	Unfavorable
	**Prioritization Of Preferences Constraints And Values**	Favorable	Unfavorable
	**Prognostication**	Favorable	Unfavorable
	**Relationship Fostering**	Favorable	Very unfavorable

**PACES**	**Addressing Patient Concerns**	Very favorable	Neither favorable nor unfavorable
	**Clinical Judgement**	Favorable	Neither favorable nor unfavorable
	**Differential Diagnosis**	Very favorable	Favorable
	**Eliciting Family History**	Cannot rate / Does not apply / Agent did not perform this	Cannot rate / Does not apply / Agent did not perform this
	**Eliciting Medication History**	Favorable	Neither favorable nor unfavorable
	**Eliciting Past Medical History**	Cannot rate / Does not apply / Agent did not perform this	Cannot rate / Does not apply / Agent did not perform this
	**Eliciting Presenting Complaint**	Favorable	Unfavorable
	**Eliciting Systems Review**	Favorable	Cannot rate / Does not apply / Agent did not perform this
	**Explaining Relevant Clinical Information Accurately**	Very favorable	Neither favorable nor unfavorable
	**Explaining Relevant Clinical Information Clearly**	Very favorable	Neither favorable nor unfavorable
	**Explaining Relevant Clinical Information Comprehensively**	Favorable	Unfavorable
	**Explaining Relevant Clinical Information Professionally**	Very favorable	Unfavorable
	**Explaining Relevant Clinical Information With Structure**	Very favorable	Neither favorable nor unfavorable

	**Maintaining Patient Welfare**	Very favorable	Neither favorable nor unfavorable
	**Showing Empathy**	Very favorable	Neither favorable nor unfavorable
	**Understanding Patient Concerns**	Very favorable	Neither favorable nor unfavorable

Patient Actor Ratings

		AMIE	PCP
**GMCPQ**	**Appearing Honest And Trustworthy**	Cannot rate / Does not apply / Agent did not perform this	Cannot rate / Does not apply / Agent did not perform this
	**Assessing Medical Condition**	Very favorable	Favorable
	**Being Polite**	Very favorable	Favorable
	**Explaining Condition and Treatment**	Very favorable	Favorable
	**Involving Patient in Treatment Decisions**	Very favorable	Neither favorable nor unfavorable
	**Listening to Patient**	Very favorable	Favorable
	**Making Patient Feel At Ease**	Very favorable	Favorable
	**Patient Confident About Care Provided (Y/N)**	Yes	Yes
	**Patient Happy to Return In Future (Y/N)**	Yes	Yes
	**Patient Trusts Information is Confidential**	Cannot rate / Does not apply / Agent did not perform this	Cannot rate / Does not apply / Agent did not perform this
	**Providing Appropriate Treatment Plan**	Cannot rate / Does not apply / Agent did not perform this	Favorable
**MXEKF**	**Acting As Patient Teacher And Salesperson**	Very favorable	Favorable

	**Communication And Shared Decision Making**	Very favorable	Neither favorable nor unfavorable
	**Managing Interplay Of Competing Priorities**	Cannot rate / Does not apply / Agent did not perform this	Cannot rate / Does not apply / Agent did not perform this
	**Monitoring And Adjustment Of Management Plan**	Cannot rate / Does not apply / Agent did not perform this	Favorable
	**Organization Of The Clinical Encounter**	Very favorable	Favorable
	**Prioritization Of Preferences Constraints And Values**	Very favorable	Favorable
	**Relationship Fostering**	Very favorable	Neither favorable nor unfavorable
**PACES**	**Addressing Patient Concerns**	Very favorable	Favorable
	**Maintaining Patient Welfare**	Very favorable	Favorable
	**Showing Empathy**	Very favorable	Neither favorable nor unfavorable
	**Understanding Patient Concerns**	Very favorable	Neither favorable nor unfavorable

Scenario B (Celiac disease)

Information for the patient actor

Patient background		
• **Name:** John Miller<br>• **Sex:** Male<br>• **Age:** 32 years<br>• **Ethnic background:** Turkish<br>• **Location:** Kingston upon Hull, UK<br>• **Lifestyle:** Non-smoker, occasionally drinks, no drug use. Sedentary lifestyle<br>• **Social and personal circumstances:** Lives with partner, no children.<br>• **Occupational history:** Accountant<br>• **Travel history:** N/A<br>• **Family history:** Sister has sleep apnea		
**Visit 1**	**Visit 2**	**Visit 3**
**In the case where the previous visit did not go as planned, alternative course of action the patient would have taken on their own:**		
N/A	On the previous visit, the patient was prescribed lab tests. If these were not done, the patient can go to a walk-in clinic due to his significant concern about his symptoms, where these tests can be prescribed.	On the previous visit, the patient was prescribed diagnostic tests. If these were not done, the patient can go to a walk-in clinic due to his significant concern about his symptoms, where these tests can be prescribed.
**Presenting complaint**		
Diarrhea and bloating	Chronic diarrhea and bloating	Chronic diarrhea and bloating
**Initial presentation**		
"Doctor, I've been experiencing diarrhea and bloating for several months now. The abdominal pain comes and goes, but it's really affecting my daily activities."	"Doctor, I'm here for the follow-up visit as my tests have already been completed, and I would like to discuss the results. Unfortunately, my symptoms have persisted."	"Doctor, since starting the gluten-free diet, I've noticed some improvement in my symptoms. The diarrhea is less frequent, and my abdominal pain has reduced. I completed the endoscopy as well."

Context		
John Miller, a 32-year-old male, presents a history of diarrhea, bloating, and weight loss over the past several months. He doesn't experience any concerning symptoms but feels a vague sense of unease due to the diarrhea.	John Miller returns for a follow-up appointment after having bloodwork done. He reports that since his last appointment a week ago, his symptoms have persisted. He still doesn't experience any concerning symptoms but is eager to discuss the results.	John returned for a follow-up visit, after having completed the endoscopy and small bowel biopsy.
**Information to be shared to the doctor if the patient is explicitly asked**		
Blood pressure: 125/80<br><br>Heart Rate: 85<br><br>Weight: 130lbs<br><br>BMI: 23<br><br>Height: 5’3<br><br>Weight loss: "I've lost about 10 pounds in the last three months without changing my diet or exercise routine."<br><br>Dietary habits: "I usually eat a balanced diet with a lot of bread, pasta, and cereals."<br><br>Bowel movements: "The diarrhea is persistent, and I go to the bathroom at least 4-5 times a day. The stools are loose and sometimes greasy."<br><br>Abdominal pain: "The pain is usually a dull ache around my lower abdomen and sometimes it's crampier."	Symptoms management: "I've been trying to avoid foods, like pasta, that make the symptoms worse, but I haven't noticed a significant improvement."<br><br>Weight: "I've continued to lose weight, about 2 more pounds since last week."<br><br>Fatigue: "The fatigue is still there, and it's hard to get through my day without feeling exhausted."<br><br>Family history: "My younger sister was diagnosed with sleep apnea a few years ago."<br><br>Past medical history: "I don’t have any past medical history."<br><br>Allergies: "I have no known allergies."<br><br>Medications: "I'm not currently taking any medications regularly."	Diet changes: "I've been strictly avoiding gluten, and my partner is helping me with meal planning."<br><br>Symptoms management: "The diarrhea is now only 1-2 times a day, and the abdominal pain is less frequent."<br><br>Weight: "I haven't lost any more weight, and I might have even gained a pound or two."<br><br>Fatigue: "The fatigue is still there but not as severe as before."<br><br>Family history: "My younger sister was diagnosed with sleep apnea a few years ago."<br><br>Past medical history: "I don’t have any past medical history."<br><br>Allergies: "I have no known allergies."

Fatigue: "I feel tired all the time, even after a full night's sleep."<br><br>Family history: "My younger sister was diagnosed with sleep apnea few years ago."<br><br>Past medical history: "I don't have any past medical history."<br><br>Allergies: "I have no known allergies."<br><br>Medications: "I'm not currently taking any medications regularly."<br><br>Lifestyle: "I don't smoke or use drugs, but I drink alcohol socially. I try to exercise a few times a week, but lately, I've been too tired."<br><br>Social history: "I live with my partner, and we don't have any children."<br><br>Travel history: "I haven't traveled recently."	Lifestyle: "I don't smoke or use drugs, but I drink alcohol socially. I try to exercise a few times a week, but lately, I've been too tired."<br><br>Social history: "I live with my partner, and we don't have any children."<br><br>Travel history: "I haven't traveled recently."	Medications: "I'm not currently taking any medications regularly."<br><br>Lifestyle: "I don't smoke, and I drink alcohol socially. I try to exercise a few times a week, but lately, I've been too tired."<br><br>Social history: "I live with my partner, and we don't have any children."<br><br>Travel history: "I haven't traveled recently."
**Past medical history and surgical history**		
N/A	N/A	Recently diagnosed with Celiac disease
**Current medications**		
N/A	N/A	N/A
**Relevant previous medications**		
N/A	N/A	N/A

Allergies and adverse reactions		
None	None	None
**Patient concerns, expectations and wishes**		
Concern: "I'm worried about why I'm losing weight and feeling so tired all the time."<br><br>Expectation: "I hope to find out what's causing these symptoms and get treatment to feel better."<br><br>Wish: "I wish to return to my normal activities and regain my energy."	Concern: "I'm worried about why I'm losing weight and feeling so tired all the time."<br><br>Expectation: "I hope to find out what's causing these symptoms and get treatment to feel better."<br><br>Wish: "I wish to return to my normal activities and regain my energy."	Concern: "I'm still worried about the fatigue and how long it will take to feel completely normal again."<br><br>Expectation: "I hope to continue improving and get back to my normal routine soon."<br><br>Wish: "I wish to understand how to maintain a gluten-free diet and avoid future health issues."
**Questions the patient wants to ask the doctor**		
"Could my symptoms be related to my diet?"<br><br>"What tests do I need to find out what's wrong?"<br><br>“Should I be worried about a more serious underlying problem?”	"What do the test results mean?"<br><br>"What are the next steps in my treatment?"<br><br>“Is this something I will have to deal with for the rest of my life?”	"How long will it take for my energy levels to return to normal?"<br><br>"Are there any other treatments or supplements I need?"<br><br>“Do I have to keep coming in for follow-ups long term?”

Visit 1

<table>
<tr><th>AMIE</th><th>PCP</th></tr>
<tr><td colspan="2" align="center">Dialogue with Patient</td></tr>
<tr><td colspan="2">[System Prompt]

Information available at the beginning of the visit

Patient: John Miller, 32 years, Male

Context: John Miller, a 32-year-old male, presents a history of diarrhea, bloating, and weight loss over the past several months. He doesn't experience any concerning symptoms but feels a vague sense of unease due to the diarrhea.

Additional information: N/A</td></tr>
<tr><td>[Patient]

Good evening doctor!

[Doctor]

Good evening John! I'm sorry to hear you've been experiencing diarrhea, bloating, and weight loss. Can you tell me a bit more about the diarrhea? How often are you having bowel movements, and what is the consistency like? Have you noticed any blood or mucus in your stool?

[Patient]

Well, the diarrhea and bloating have been happening for several months now. I do get some abdominal pain, which comes and goes. The diarrhea is just constant. I go to the bathroom at least 4-5 times a day. The stool is mostly loose and sometimes greasy. I haven't seen any blood though.</td><td>[Patient]

Hi doctor!

[Doctor]

Hi John! Sorry to hear about your recent symptoms -- hoping we can help you out. Sounds like you've been having some ongoing symptoms over the last few months -- diarrhea, weight loss, and bloating. Do you have any other symptoms, like fevers or chills, nausea or vomiting, or blood in the stool (or really dark/black stool)?

[Patient]

No, nothing else. Just a vague sense of unease during the diarrhea.

[Doctor]</td></tr>
</table>

[Doctor]

Thanks for sharing that. Can you describe the abdominal pain? Where is it located? What kind of pain is it (sharp, cramping, dull ache)? Does anything make it better or worse? Finally, have you noticed any unintentional weight loss?

[Patient]

The abdominal pain is mostly in the lower abdomen. It's usually dull, but sometimes, it feels like cramps. And yes, I have lost weight. I'm not very active, but I've still lost about 10 pounds in the last 3 months.

[Doctor]

I understand. Have you noticed any changes in your appetite? Also, have you been experiencing any nausea or vomiting?

[Patient]

No, I haven't noticed any change in my appetite. And no, no nausea or vomiting.

[Doctor]

Okay, thank you. Have you made any recent dietary changes?

[Patient]

No, I haven't made any changes to my diet. I usually eat pretty balanced. But maybe a little more emphasis on bread, pasta, and cereals.

[Doctor]

Okay, that's helpful. Have you traveled recently, or been around anyone who's been sick? Also, does anyone in your family have

Do you have any ongoing medical conditions? Or prior major illnesses or surgeries in the past?

[Patient]

Nope!

[Doctor]

Any major illnesses in your family (parents, siblings)? Things like inflammatory bowel conditions, colorectal cancer, or any other conditions?

[Patient]

My sister was diagnosed with sleep apnea. That's all I'm aware of.

[Doctor]

Okay, good to know. Thanks! Tell me more about the "unease" — is there a sense of urgency, or having to suddenly go? Any discharge or mucous from the anal area?

[Patient]

I can't really describe the unease. Just very vague. The diarrhea is persistent. I go almost 4-5 times a day. The stool is usually loose and sometimes greasy. No discharge as far as I've noticed.

[Doctor]

Any bouts of constipation as well? Any abdominal pain?

[Patient]

celiac disease or inflammatory bowel disease?

[Patient]

No, none of that.

[Doctor]

Okay. Have you ever used over-the-counter medications for diarrhea or probiotics? Do you have any known food allergies or intolerances?

[Patient]

No I haven't tried anything off the counter. And I don't have any allergies.

[Doctor]

Okay, thank you. Based on your symptoms, several possibilities exist, including celiac disease, irritable bowel syndrome (IBS), and small intestinal bacterial overgrowth (SIBO). To determine the cause, I'll order blood tests (complete blood count and celiac panel) and a stool test to check for fat and infections. Does this plan sound alright? Any questions?

[Patient]

Yea, that sounds good. I just want to be able to do my normal things and have some energy again.

[Doctor]

I understand. To help with diagnosis, please try reducing your intake of high-gluten foods like bread, pasta, and cereals. Drink plenty of fluids. I'll schedule a follow-up in 2-4 weeks to

No constipation. But abdominal pain, yes. It's a dull ache in the lower area, sometimes crampy.

[Doctor]

Hm, sorry to hear. Sounds uncomfortable. Any changes to skin, like any yellowing that you've noticed?

[Patient]

No, nothing like that.

[Doctor]

How is your diet overall? Any foods that seem to make this worse? Any smoking or use of alcohol, or other recreational drugs?

[Patient]

I usually eat pretty balanced, but more bread, pasta, and cereals. I just drink socially.

[Doctor]

Thank you for the information. Any medications that you're currently taking? Any allergies to medications

[Patient]

No. Not taking anything and no allergies either.

[Doctor]

Any recent travel or exposure to untreated water, such as when camping or otherwise?

[Patient]

No, nothing.

review test results. Let me know if you have any questions before then.

[Patient]

Sure sounds good. I'll do that. So you think my symptoms could be related to my diet?

[Doctor]

Yes, it's possible. Celiac disease, a possibility I'm considering, is triggered by gluten found in wheat, barley, and rye. The dietary changes will help determine if this is the case. We'll discuss the test results at your follow-up.

[Patient]

Alright. Sounds good. Thank you so much, doctor!

[Doctor]

You're welcome, John. Remember to reduce your intake of high-gluten foods and stay hydrated. We'll review the test results at your follow-up in 2-4 weeks. Take care.

[Doctor]

How much weight have you lost over the last several months?

[Patient]

Maybe about 10 pounds in the last 3 months. I haven't changed my diet or exercise routine though.

[Doctor]

Have you ever experienced this before?

[Patient]

No, I haven't.

[Doctor]

Okay, thank you. I'm not quite sure what is causing your symptoms, but I think we should do some testing and a physical examination to understand what is going on. Have you done any testing recently?

[Patient]

Nope

[Doctor]

I'm not sure if I have something immediately to address your symptoms unfortunately, but I would avoid common gastrointestinal irritants (things like spicy foods, alcohol, anti-inflammatory medications) for the shortterm until we can do some more tests to understand what is going on. I think we should do some stool tests, some blood tests, and work with a specialist to coordinate further assessment and a possible

	endoscopy to see what's going on. I think we should be able to figure this out!<br><br>[Patient]<br><br>Oh ok cool Sounds good.<br><br>[Doctor]<br><br>Do you have any questions for me before I get this arranged?<br><br>[Patient]<br><br>No, not really. Just want to get this figured out.<br><br>[Doctor]<br><br>Absolutely -- I'm sure it's had a big impact on you, so we'll get these next steps moving to get you an answer and get you feeling better.
**Responses to Post-Questionnaire**	
Guidelines Selected as Relevant	
• [bmj122] Irritable bowel syndrome<br>• [bmj636] Coeliac disease<br>• [bmj67] Chronic pancreatitis<br>• [cg61] Irritable bowel syndrome in adults: diagnosis and management<br>• [ng20] Coeliac disease: recognition, assessment and management<br>• [qs134] Coeliac disease: quality standard	• [bmj42] Crohn's disease<br>• [bmj43] Ulcerative colitis<br>• [bmj636] Coeliac disease<br>• [ng20] Coeliac disease: recognition, assessment and management<br>• [qs134] Coeliac disease: quality standard<br>• [qs81] Inflammatory bowel disease: quality standard
Differential Diagnosis	
1. Celiac disease<br>2. Irritable bowel syndrome (IBS)<br>3. Small intestinal bacterial overgrowth (SIBO)<br>4. Exocrine pancreatic insufficiency<br>5. Microscopic colitis	1. Crohn's colitis<br>2. Ulcerative colitis<br>3. Celiac disease<br>4. Infectious colitis (e.g. giardia)<br>5. Colon cancer<br>6. Pancreatitis<br>7. Irritable bowel syndrome

	8. Hyperthyroidism<br>9. Food intolerance<br>10. Pancreatic exocrine insufficiency
In-visit Investigations	
None	1. Weight change from baseline
Ordered Investigations	
1. Order a complete blood count (CBC) to assess for anemia and infection.<br><br>2. Order a celiac panel (tTG IgA and total IgA antibodies, and consider IgG antibodies if IgA is deficient) to assess for celiac disease. Advise the patient to maintain a gluten-containing diet for at least 6 weeks prior to testing for accurate results.<br><br>3. Order a stool test for fat content (Sudan III stain or quantitative fecal fat) to assess for malabsorption and screen for infectious causes.<br><br>4. Order a fecal elastase-1 test to assess exocrine pancreatic function. This will help determine if exocrine pancreatic insufficiency is a contributing factor to his malabsorption.	1. CBC<br>2. ESR<br>3. CRP<br>4. AST/ALT/GGT/bilirubin<br>5. Sodium, potassium, creatinine<br>6. Fecal calprotectin<br>7. Lipase<br>8. Stool culture and sensitivities<br>9. Stool ova and parasites<br>10. Stool for C difficile toxin<br>11. Total IgA<br>12. Tissue transglutaminase IgA<br>13. Gastroenterology specialist assessment +/- colonoscopy
Recommendations or Actions	
1. Recommend reducing intake of high-gluten foods (bread, pasta, cereals) while awaiting test results. This will help determine if gluten sensitivity is a factor and potentially alleviate some GI symptoms.<br><br>2. Recommend increasing fluid intake to manage diarrhea and prevent dehydration.<br><br>3. Recommend keeping a food diary to help identify potential food triggers. He should also track bowel movements and abdominal pain.	1. Physical examination and vital signs to assess for any other signs of systemic illness or focal disease<br><br>2. Recommend minimizing any gastrointestinal irritants (NSAIDs, spicy foods, alcohol, etc)<br><br>3. Adequate fluids and hydration given GI losses<br><br>4. Gastroenterology specialist assessment +/- colonoscopy

4. Advise the patient on the appropriate use of loperamide for symptomatic relief of diarrhea, if needed, emphasizing that it is for short-term use only and not a long-term solution for his condition. Instruct him to contact the office sooner if symptoms worsen, severe abdominal pain occurs, or if he develops fever or bloody stools.<br><br>5. Schedule a follow-up appointment in 2-4 weeks to review test results and discuss further management, including potential referral to a gastroenterologist if necessary.<br><br>6. Educate the patient about the possible causes of his symptoms, the importance of adherence to dietary modifications, and answer any questions.	
Did you intentionally deviate from one of the clinical guidelines you selected above?	
No	No
If yes, please explain why (e.g. guideline does not cover this clinical scenario, patient preference, your preference). If no, enter 'N/A':	
N/A	N/A
What are other acceptable management plans? Why did you choose yours?	
While the initial investigations and recommendations largely align with multiple guidelines, there's flexibility in the approach. For example, the order of stool tests could be adjusted based on local lab capabilities and urgency. The decision to include a hydrogen breath test for SIBO is debatable, as the guidelines don't explicitly recommend it for initial assessment in this scenario. I chose the current plan because it addresses the most likely possibilities (celiac disease and malabsorption) first, while also allowing for investigation of other potential causes like SIBO and microscopic colitis if the initial tests are negative. A more aggressive approach might involve earlier referral to a gastroenterologist, but given the lack of alarming symptoms, a phased approach	Most acceptable plans would include some subset of these tests for workup of these chronic symptoms

seems more prudent.	
Please describe any competing priorities that affected your decisions:	
N/A	No other competing priorities at this stage
Describe the cost, effectiveness and side effects of your management plan:	
The cost is moderate, encompassing routine blood tests, stool analysis, and a celiac panel. Effectiveness depends on the accuracy of the tests in identifying the underlying cause. Side effects are generally minimal for the ordered tests, though some patients may experience discomfort during blood draws or stool collection. The dietary recommendations have minimal side effects, but may cause some initial inconvenience. Loperamide, if used, carries a risk of constipation and other side effects, but is generally well-tolerated for short-term use.	This set of tests, the work-up and the management plan would be effective with minimal side effects, and are not overly expensive and would provide important information that can help reveal (or rule-out) a serious underlying medical condition.
Describe the prognosis with and without treatment of this patient:	
Without treatment, John's prognosis is uncertain. His symptoms (diarrhea, bloating, weight loss) could worsen, leading to potential nutritional deficiencies, dehydration, and a decreased quality of life. The underlying cause needs to be identified and addressed to improve his prognosis.	Without adequate diagnosis and treatment, there is a significant risk of deterioration and worsening of their underlying condition, with potentially serious health ramifications.
Does this case require escalation of this text-based consultation to a video-call or in-person consultation?	
No, does not require escalation	Yes, requires escalation to an IN-PERSON consultation
Describe why it requires or does not require escalation:	
The initial management plan is appropriate for a text-based consultation. The investigations are standard and readily available. Escalation to a video call or in-person consultation would be considered if the initial test results are inconclusive or reveal alarming findings.	Would benefit from in-person physical examination with a short time frame (1-2 weeks).
Is a follow-up required?	
Yes	Yes

If yes, when would you like to follow-up the patient? Otherwise, enter 'N/A':	
2-4 weeks	Within ~1-2 weeks (once initial testing results are available).
Describe why it requires or does not require follow-up:	
To review test results, discuss findings, and determine the next steps in management, including potential referral to a gastroenterologist if necessary.	Follow-up is warranted because of the potential serious nature of these symptoms, and possible deterioration of this individual's health.

Specialist Physician Ratings

		AMIE	PCP
**Diagnosis & Management**	**Confabulation Absent (Y/N)**	Yes	Yes
	**DDx Appropriateness**	Very favorable	Very favorable
	**DDx Comprehensiveness (4-point scale)**	Very favorable	Very favorable
	**Escalation Recommendation Appropriate (Y/N)**	Yes	Yes
	**Followup Recommendation Appropriate (Y/N)**	Yes	Yes
	**Guideline Entailment Overall**	Very favorable	Very favorable
	**Investigation Aligned With Guidelines**	Very favorable	Very favorable
	**Investigation Appropriate Recommended (Y/N)**	Yes	Yes
	**Investigation Cites Guidelines**	Very favorable	Very favorable
	**Investigation Inappropriate Avoided (Y/N)**	Yes	Yes
	**Investigation Sufficiently Precise (Y/N)**	Yes	Yes
	**Management Aligned With Guidelines (3-point scale)**	Favorable	Favorable
	**Management Free of Clinically Significant Error (Y/N)**	Yes	Yes
	**Management Plan Appropriateness**	Very favorable	Very favorable

	**Remembering Important Information From Previous Visits (3-point scale)**	Favorable	Favorable
	**Treatment Aligned With Guidelines**	Very favorable	Very favorable
	**Treatment Appropriate Recommended (Y/N)**	Yes	Yes
	**Treatment Cites Guidelines**	Very favorable	Very favorable
	**Treatment Inappropriate Avoided (Y/N)**	Yes	Yes
	**Treatment Sufficiently Precise (Y/N)**	Yes	Yes
**MXEKF**	**Acting As Patient Teacher And Salesperson**	Very favorable	Favorable
	**Communication And Shared Decision Making**	Very favorable	Favorable
	**Contrasting\nand Selection**	Very favorable	Favorable
	**Illness Specific Knowledge**	Very favorable	Favorable
	**Managing Interplay Of Competing Priorities**	Very favorable	Favorable
	**Monitoring And Adjustment Of Management Plan**	Favorable	Favorable
	**Organization Of The Clinical Encounter**	Very favorable	Favorable
	**Prioritization Of Preferences Constraints And Values**	Very favorable	Favorable
	**Prognostication**	Very favorable	Favorable

	**Relationship Fostering**	Very favorable	Favorable
**PACES**	**Addressing Patient Concerns**	Very favorable	Very favorable
	**Clinical Judgement**	Very favorable	Very favorable
	**Differential Diagnosis**	Very favorable	Very favorable
	**Eliciting Family History**	Very favorable	Very favorable
	**Eliciting Medication History**	Very favorable	Very favorable
	**Eliciting Past Medical History**	Very favorable	Very favorable
	**Eliciting Presenting Complaint**	Very favorable	Very favorable
	**Eliciting Systems Review**	Very favorable	Very favorable
	**Explaining Relevant Clinical Information Accurately**	Very favorable	Very favorable
	**Explaining Relevant Clinical Information Clearly**	Very favorable	Very favorable
	**Explaining Relevant Clinical Information Comprehensively**	Favorable	Very favorable
	**Explaining Relevant Clinical Information Professionally**	Very favorable	Very favorable
	**Explaining Relevant Clinical Information With Structure**	Favorable	Very favorable
	**Maintaining Patient Welfare**	Very favorable	Very favorable

	**Showing Empathy**	Very favorable	Very favorable
	**Understanding Patient Concerns**	Very favorable	Very favorable

Patient Actor Ratings

		AMIE	PCP
**GMCPQ**	**Appearing Honest And Trustworthy**	Cannot rate / Does not apply / Agent did not perform this	Cannot rate / Does not apply / Agent did not perform this
	**Assessing Medical Condition**	Very favorable	Very favorable
	**Being Polite**	Very favorable	Very favorable
	**Explaining Condition and Treatment**	Very favorable	Very favorable
	**Involving Patient in Treatment Decisions**	Very favorable	Very favorable
	**Listening to Patient**	Very favorable	Very favorable
	**Making Patient Feel At Ease**	Very favorable	Very favorable
	**Patient Confident About Care Provided (Y/N)**	Yes	Yes
	**Patient Happy to Return In Future (Y/N)**	Yes	Yes
	**Patient Trusts Information is Confidential**	Cannot rate / Does not apply / Agent did not perform this	Cannot rate / Does not apply / Agent did not perform this
	**Providing Appropriate Treatment Plan**	Very favorable	Very favorable
**MXEKF**	**Acting As Patient Teacher And Salesperson**	Cannot rate / Does not apply / Agent did not perform this	Favorable
	**Communication And Shared Decision Making**	Very favorable	Very favorable

	**Managing Interplay Of Competing Priorities**	Cannot rate / Does not apply / Agent did not perform this	Cannot rate / Does not apply / Agent did not perform this
	**Monitoring And Adjustment Of Management Plan**	Cannot rate / Does not apply / Agent did not perform this	Cannot rate / Does not apply / Agent did not perform this
	**Organization Of The Clinical Encounter**	Very favorable	Very favorable
	**Prioritization Of Preferences Constraints And Values**	Cannot rate / Does not apply / Agent did not perform this	Cannot rate / Does not apply / Agent did not perform this
	**Relationship Fostering**	Favorable	Favorable
**PACES**	**Addressing Patient Concerns**	Very favorable	Favorable
	**Maintaining Patient Welfare**	Very favorable	Very favorable
	**Showing Empathy**	Favorable	Very favorable
	**Understanding Patient Concerns**	Very unfavorable	Favorable

Visit 2

AMIE	PCP
**Dialogue with Patient**	

[System Prompt]

Information available at the beginning of the visit

Patient: John Miller, 32 years, Male

Context: John Miller returns for a follow-up appointment after having bloodwork done. He reports that since his last appointment a week ago, his symptoms have persisted. He still doesn't experience any concerning symptoms but is eager to discuss the results.

Additional information:

Lab results:

Tissue transglutaminase antibodies (tTG-IgA): 45 U/mL ; Elevated levels of tTG-IgA indicate an immune response to gluten.

Total IgA: 2.5 g/L ; Normal levels of total IgA ensure the reliability of IgA-based tests. Reference Range: 0.7 - 4.0 g/L

Complete Blood Count (CBC): Hemoglobin: 11.5 g/dL (low) Hematocrit: 35% (low) Platelet Count: 220 x 10^9/L

Mean Corpuscular Volume (MCV): 90 fL

Mean Corpuscular Hemoglobin (MCH): 29 pg/cell

Mean Corpuscular Hemoglobin Concentration (MCHC): 33%

White Blood Cell (WBC) Count: 6.0 x 10^9/mm^3

Neutrophils: 4.0 x 10^3/mm^3

Lymphocytes: 1.5 x 10^3/mm^3

Monocytes: 0.3 x 10^3/mm^3

Eosinophils: 0.2 x 10^3/mm^3

<table>
<tr><td colspan="2">Basophils: 0.1 x 10^3/mm^3

Platelet Count: 250 x 10^9/mm^3

Reticulocytes: 1% of total RBC count

Vitamin Levels:

Folate: 12ng/ml (Normal Range: 2.7 - 17.0 ng/mL (or 6.0 - 38.7 nmol/L))

Cobalamin: 400pg/mL (Normal range: 200 - 900 pg/mL (or 148 - 665 pmol/L))

Vitamin D: 40ng/mL (Normal range: 20 - 50 ng/mL (or 50 - 125 nmol/L))

Iron: 100µg/dL (Normal range: 60 - 170 µg/dL (or 10.7 - 30.4 µmol/L))</td></tr>
<tr><td>[Patient]

Good morning, doctor!

[Doctor]

Good morning John! It's good to see you again. Let's review your lab results. Your tTG-IgA antibodies are elevated, as we suspected. How have you felt since our last visit? Have your symptoms changed at all?

[Patient]

Oh ok. What does that mean exactly...those antibodies being high? And yea, just feeling pretty much the same since last visit.

[Doctor]

The elevated tTG-IgA antibodies suggest your body is reacting to gluten, a protein in wheat, barley, and rye. This strongly indicates celiac disease. Your low hemoglobin and hematocrit suggest anemia, which we'll investigate. Has the frequency or consistency of your diarrhea changed? How's the abdominal pain? Any</td>
<td>[Patient]

Good morning, doctor!

[Doctor]

Hi John -- thanks for checking in. How are you feeling? I see you did some blood tests already, and that your symptoms have been persisting.

[Patient]

Yea, that's right.

[Doctor]

Anything new or different in terms of your symptoms? And, has anyone talked to you about your results yet?

[Patient]

No, I haven't had any new symptoms. Nothing different on that front. And no, I haven't heard anything about my results yet. I'd like to find out what came back!</td></tr>
</table>

new symptoms like fatigue or rashes? Your vitamin levels are mostly normal, but we'll monitor your iron given the anemia. To confirm celiac disease, I recommend a small intestinal biopsy. This involves a small tissue sample from your small intestine for microscopic examination. It helps confirm the diagnosis and rule out other possibilities. Are you comfortable with a biopsy? We can discuss next steps if it confirms celiac disease. How would you rate your diarrhea and abdominal pain on a scale of 1-10 (1=mild, 10=worst)?<br><br>[Patient]<br><br>No, diarrhea is still the same. Abdominal pain as well. Nothing new, no rashes. I do continue to feel tired. Just constantly exhausted. I don't mind doing the biopsy...whatever it takes to get to the bottom of this issue. Just want to feel normal finally. I'd rate the diarrhea at a 7 and the abdominal pain at a 7 as well. I did try to avoid the foods you mentioned last time. I haven't noticed much change yet though.<br><br>[Doctor]<br><br>I appreciate your willingness to do the biopsy. It's a crucial step to help us understand what's going on. I'm sorry to hear the fatigue is persistent; it's common with celiac disease due to nutrient malabsorption. It's good you've been trying to avoid gluten, but improvement can take time.<br><br>Based on your symptoms, the elevated tTG-IgA, and the anemia, I'm leaning towards a diagnosis of celiac disease. Let's proceed with the biopsy to confirm.	[Doctor]<br><br>Good to know. It looks like, based on the bloodwork, that you likely have a condition called "celiac disease", which is caused by an immune system reaction to gluten, which is common in many types of foods, such as breads, pastas etc. The immune system reacts to the gluten in the intestines, causing inflammation and damage, which likely contributes to the symptoms you've been having (diarrhea, weight loss, abdominal discomfort), and can also have impacts on how we absorb nutrients.<br><br>[Patient]<br><br>Ah ok. I've been trying to avoid pastas and stuff like you mentioned last appointment. I haven't much improvement yet though.<br><br>[Doctor]<br><br>Thanks for sharing -- did it help your symptoms at all?<br><br>[Patient]<br><br>No, not really. At least not yet.<br><br>[Doctor]<br><br>Okay. I think we need to set up an appointment with a gastroenterologist for the next steps of the assessment, which would involve using a camera to look at your intestines and take a small sample of the tissue (a biopsy) to confirm the diagnosis. To make sure this diagnosis is as accurate as possible, it's actually important to still keep up with a small amount of gluten (1 meal per day -- breads, pasta, etc) until we complete this next step of testing. It seems

In the meantime, here's a plan to manage your symptoms:

- Continue the gluten-free diet: Consistency is key; improvement may take weeks or months.
- Iron supplementation: We'll address your anemia with iron supplements.
- Vitamin monitoring: We'll monitor your iron, folate, and B12 levels.

Does this plan sound okay? Any questions?

[Patient]

Yes, this sounds great. Is this something I'll have to deal with for the rest of my life or can it get better?

[Doctor]

That's a good question. Celiac disease is a lifelong condition, so there's no cure. However, with a strict gluten-free diet, most people see significant symptom improvement and can live healthy lives. Continuing to eat gluten keeps your small intestine damaged, and your symptoms will stay. But, on a gluten-free diet, that damage can heal, and you'll feel better. It requires commitment, but with support and resources, it's manageable. We can provide you with information and resources to help.

Does that make sense?

[Patient]

Yea, that makes sense. I'm glad there's help out there for this. Just sucks that it's going to be a life long issue.

[Doctor]

counter-intuitive, but will help us understand how best to improve your symptoms.

[Patient]

Oh ok sure I can do that.

[Doctor]

Okay, we'll get this testing arranged as soon as possible, and they should see you within a few weeks. The blood tests also showed a few other abnormalities related to lower blood count levels, but these are probably all related to the likely diagnosis of celiac disease, and would improve once we confirm the diagnosis and treat it appropriately.

[Patient]

Alright, sounds good. So I'll be hearing from your office or the endocrinologist once it's set up?

[Doctor]

Yes, we'll get this arranged right away. It might be good to track your symptoms over the next little while as well as your weight. Keep up with the small amounts of the gluten in your diet for now until you see the gastroenterologist. I think we can confirm the diagnosis quickly, and figure out the next steps to help you feel better.

[Patient]

Great. Thank you! I'll keep an eye on things for sure.

[Doctor]

Sounds good -- any other questions for me?

<table>
<tr><td>I understand. It's a lot to take in, but many find it manageable, and the benefits are worth it. We'll support you. Let's schedule that biopsy and get you started on iron. I'd like to see you again in a month to discuss the results and adjust your plan as needed. Until then, continue the gluten-free diet, watch your symptoms, and don't hesitate to reach out with questions.

[Patient]

Sure thing! Thanks a bunch, doctor!

[Doctor]

You're welcome, John. Take care.</td><td></td></tr>
<tr><td colspan="2">Responses to Post-Questionnaire</td></tr>
<tr><td colspan="2">Guidelines Selected as Relevant</td></tr>
<tr><td>● [bmj122] Irritable bowel syndrome
● [bmj636] Coeliac disease
● [cg61] Irritable bowel syndrome in adults: diagnosis and management
● [ng20] Coeliac disease: recognition, assessment and management
● [qs134] Coeliac disease: quality standard</td><td>● [bmj636] Coeliac disease
● [ng20] Coeliac disease: recognition, assessment and management
● [qs134] Coeliac disease: quality standard</td></tr>
<tr><td colspan="2">Differential Diagnosis</td></tr>
<tr><td>1. Celiac disease
2. Small intestinal bacterial overgrowth (SIBO)
3. Irritable bowel syndrome (IBS)
4. Microscopic colitis
5. Tropical sprue</td><td>1. Celiac disease
2. Nonceliac gluten sensitivity
3. Inflammatory bowel disease
4. Infectious colitis
5. Colon cancer</td></tr>
<tr><td colspan="2">In-visit Investigations</td></tr>
<tr><td>None</td><td>None</td></tr>
<tr><td colspan="2">Ordered Investigations</td></tr>
</table>

1. Order a small intestinal biopsy (endoscopic procedure with duodenal biopsies) to confirm the diagnosis of celiac disease and assess the severity of villous atrophy. This is the gold standard for diagnosis.<br><br>2. Order a ferritin blood test to assess iron stores and guide iron supplementation.<br><br>3. Order a repeat CBC (complete blood count) in 2 weeks to monitor the response to iron supplementation and assess for anemia improvement.	1. Gastroenterology consultation for upper endoscopy + biopsy
Recommendations or Actions	
1. Continue the strict gluten-free diet. Provide detailed information on gluten-free dietary management, including reading food labels, avoiding cross-contamination, and meal planning strategies.<br><br>2. Prescribe ferrous sulfate 325mg once daily to treat iron deficiency anemia. Instruct the patient to take the supplement with Vitamin C to enhance absorption. Monitor for side effects such as constipation or nausea. Advise the patient to increase fluid intake and consider stool softeners if constipation occurs.<br><br>3. Refer the patient to a registered dietitian specializing in celiac disease for personalized dietary guidance and ongoing support. This will help ensure adequate nutrient intake while adhering to the gluten-free diet.<br><br>4. Schedule a follow-up appointment in 4 weeks to review the biopsy results, assess the response to iron supplementation (via repeat CBC and ferritin), and discuss further management strategies. This will also involve a discussion of the patient's experience with	1. Gastroenterology consultation for upper endoscopy + biopsy<br><br>2. Continue with small amounts of gluten in diet until specialist consultation and endoscopy complete

the gluten-free diet and any challenges they are facing.<br><br>5. Educate the patient about celiac disease, including its lifelong nature, the importance of strict dietary adherence, potential long-term complications, and available support resources (e.g., Celiac UK). Address the patient's concerns about the lifelong nature of the condition and provide reassurance and emotional support.<br><br>6. Instruct the patient to monitor their symptoms (diarrhea, bloating, abdominal pain, fatigue) and contact the office immediately if symptoms worsen, new symptoms develop (especially bloody stools or fever), or if they experience any side effects from the iron supplementation.	
Did you intentionally deviate from one of the clinical guidelines you selected above?	
No	No
If yes, please explain why (e.g. guideline does not cover this clinical scenario, patient preference, your preference). If no, enter 'N/A':	
N/A	N/A
What are other acceptable management plans? Why did you choose yours?	
The management plan is largely consistent with NICE and BMJ guidelines for celiac disease. However, there's flexibility in the approach. For instance, the decision to immediately prescribe iron supplements is based on the patient's low hemoglobin and hematocrit, even before the biopsy confirms the diagnosis. While guidelines emphasize the importance of confirming the diagnosis before starting treatment, the severity of the anemia warrants prompt intervention to prevent further complications. Another acceptable plan might involve waiting for biopsy results before initiating iron supplementation, but this could delay treatment for a potentially serious condition. I chose the current plan because the benefits	This is likely the only acceptable management plan, as the diagnosis still needs to be confirmed with endoscopy/histology.

of early iron supplementation outweigh the risks of potentially delaying treatment for anemia. The patient's significant fatigue and the severity of his anemia (Hb 11.5 g/dL, Hct 35%) support this decision. A more conservative approach would be to wait for the biopsy results before starting iron supplementation, but this could delay treatment for a potentially serious condition.	
Please describe any competing priorities that affected your decisions:	
N/A	None
Describe the cost, effectiveness and side effects of your management plan:	
The cost is moderate, including a small intestinal biopsy, ferritin blood test, and a repeat CBC. Effectiveness depends on the accuracy of the biopsy in confirming celiac disease and the patient's adherence to the gluten-free diet and iron supplementation. Side effects are minimal for the tests, but iron supplementation can cause constipation or nausea. The gluten-free diet may initially cause some inconvenience, but with proper guidance, it's manageable. The long-term effectiveness and cost-effectiveness of the plan depend on the patient's adherence to the gluten-free diet and the management of potential complications.	There is relatively low risk to upper endoscopy + biopsy, but this outweighed by the benefits of confirming the diagnosis and ensuring the patient receives the right treatment.
Describe the prognosis with and without treatment of this patient:	
Without treatment, John's prognosis is poor. His symptoms (diarrhea, bloating, weight loss, fatigue) could worsen, leading to severe nutritional deficiencies, anemia, and a significantly decreased quality of life. Untreated celiac disease can lead to long-term complications such as osteoporosis, increased risk of certain cancers, and other health issues. The underlying cause needs to be addressed to improve his prognosis.	The prognosis is likely excellent if the diagnosis is confirmed, and the patient can adhere to a gluten-free diet. Without treatment, there is strong likelihood for significant worsening of symptoms and disease, and systemic impacts from major nutritional deficiencies (vitamin D, iron, b12, etc) due to impaired gut absorption.
Does this case require escalation of this text-based consultation to a video-call or in-person consultation?	
No, does not require escalation	Yes, requires escalation to an IN-PERSON consultation

<table>
<tr><td colspan="2">Describe why it requires or does not require escalation:</td></tr>
<tr><td>The current visit's management plan is appropriate for a text-based consultation. The investigations are standard and readily available. Escalation to a video call or in-person consultation would be considered if the initial test results were inconclusive or revealed alarming findings. However, given the elevated tTG-IgA, low hemoglobin, and the patient's willingness to proceed with a biopsy, a text-based follow-up is sufficient for now.</td><td>This requires an in-person assessment and endoscopy to rule-out / confirm the diagnosis</td></tr>
<tr><td colspan="2">Is a follow-up required?</td></tr>
<tr><td>Yes</td><td>Yes</td></tr>
<tr><td colspan="2">If yes, when would you like to follow-up the patient? Otherwise, enter 'N/A':</td></tr>
<tr><td>4 weeks</td><td>After consultation with the gastroenterologist is complete (ideally within 3-4 weeks).</td></tr>
<tr><td colspan="2">Describe why it requires or does not require follow-up:</td></tr>
<tr><td>To review the biopsy results, assess the response to iron supplementation (via repeat CBC and ferritin), and discuss further management strategies. This will also involve a discussion of the patient's experience with the gluten-free diet and any challenges they are facing.</td><td>Likely will need follow-up to discuss the endoscopy results and to craft a plan for management (or further follow-up tests, as necessar), along with monitoring of symptoms and weight over time.</td></tr>
</table>

Specialist Physician Ratings

		AMIE	PCP
**Diagnosis & Management**	**Confabulation Absent (Y/N)**	Yes	Yes
	**DDx Appropriateness**	Very favorable	Very favorable
	**DDx Comprehensiveness (4-point scale)**	Very favorable	Very favorable
	**Escalation Recommendation Appropriate (Y/N)**	Yes	Yes
	**Followup Recommendation Appropriate (Y/N)**	Yes	Yes
	**Guideline Entailment Overall**	Very favorable	Very favorable
	**Investigation Aligned With Guidelines**	Very favorable	Very favorable
	**Investigation Appropriate Recommended (Y/N)**	Yes	Yes
	**Investigation Cites Guidelines**	Very favorable	Very favorable
	**Investigation Inappropriate Avoided (Y/N)**	Yes	Yes
	**Investigation Sufficiently Precise (Y/N)**	Yes	Yes
	**Management Aligned With Guidelines (3-point scale)**	Favorable	Favorable
	**Management Free of Clinically Significant Error (Y/N)**	Yes	Yes
	**Management Plan Appropriateness**	Very favorable	Very favorable

	**Remembering Important Information From Previous Visits (3-point scale)**	Favorable	Favorable
	**Treatment Aligned With Guidelines**	Very favorable	Very favorable
	**Treatment Appropriate Recommended (Y/N)**	Yes	Yes
	**Treatment Cites Guidelines**	Very favorable	Very favorable
	**Treatment Inappropriate Avoided (Y/N)**	Yes	Yes
	**Treatment Sufficiently Precise (Y/N)**	Yes	Yes
**MXEKF**	**Acting As Patient Teacher And Salesperson**	Very favorable	Very favorable
	**Communication And Shared Decision Making**	Very favorable	Very favorable
	**Contrasting\nand Selection**	Very favorable	Very favorable
	**Illness Specific Knowledge**	Very favorable	Very favorable
	**Managing Interplay Of Competing Priorities**	Very favorable	Very favorable
	**Monitoring And Adjustment Of Management Plan**	Very favorable	Favorable
	**Organization Of The Clinical Encounter**	Very favorable	Very favorable
	**Prioritization Of Preferences Constraints And Values**	Very favorable	Favorable
	**Prognostication**	Very favorable	Very favorable

	**Relationship Fostering**	Very favorable	Very favorable
**PACES**	**Addressing Patient Concerns**	Very favorable	Very favorable
	**Clinical Judgement**	Very favorable	Very favorable
	**Differential Diagnosis**	Very favorable	Very favorable
	**Eliciting Family History**	Very favorable	Very favorable
	**Eliciting Medication History**	Very favorable	Very favorable
	**Eliciting Past Medical History**	Very favorable	Very favorable
	**Eliciting Presenting Complaint**	Very favorable	Very favorable
	**Eliciting Systems Review**	Very favorable	Very favorable
	**Explaining Relevant Clinical Information Accurately**	Very favorable	Very favorable
	**Explaining Relevant Clinical Information Clearly**	Very favorable	Very favorable
	**Explaining Relevant Clinical Information Comprehensively**	Very favorable	Very favorable
	**Explaining Relevant Clinical Information Professionally**	Very favorable	Very favorable
	**Explaining Relevant Clinical Information With Structure**	Very favorable	Very favorable
	**Maintaining Patient Welfare**	Very favorable	Very favorable

	**Showing Empathy**	Very favorable	Very favorable
	**Understanding Patient Concerns**	Very favorable	Very favorable

Patient Actor Ratings

		AMIE	PCP
**GMCPQ**	**Appearing Honest And Trustworthy**	Cannot rate / Does not apply / Agent did not perform this	Cannot rate / Does not apply / Agent did not perform this
	**Assessing Medical Condition**	Very favorable	Very favorable
	**Being Polite**	Very favorable	Very favorable
	**Explaining Condition and Treatment**	Very favorable	Very favorable
	**Involving Patient in Treatment Decisions**	Very favorable	Very favorable
	**Listening to Patient**	Very favorable	Very favorable
	**Making Patient Feel At Ease**	Very favorable	Very favorable
	**Patient Confident About Care Provided (Y/N)**	Yes	Yes
	**Patient Happy to Return In Future (Y/N)**	Yes	Yes
	**Patient Trusts Information is Confidential**	Cannot rate / Does not apply / Agent did not perform this	Cannot rate / Does not apply / Agent did not perform this
	**Providing Appropriate Treatment Plan**	Very favorable	Very favorable
**MXEKF**	**Acting As Patient Teacher And Salesperson**	Very favorable	Very favorable
	**Communication And Shared Decision Making**	Very favorable	Very favorable

	**Managing Interplay Of Competing Priorities**	Very favorable	Cannot rate / Does not apply / Agent did not perform this
	**Monitoring And Adjustment Of Management Plan**	Very favorable	Very favorable
	**Organization Of The Clinical Encounter**	Very favorable	Very favorable
	**Prioritization Of Preferences Constraints And Values**	Very favorable	Cannot rate / Does not apply / Agent did not perform this
	**Relationship Fostering**	Very favorable	Cannot rate / Does not apply / Agent did not perform this
**PACES**	**Addressing Patient Concerns**	Very favorable	Very favorable
	**Maintaining Patient Welfare**	Very favorable	Very favorable
	**Showing Empathy**	Favorable	Neither favorable nor unfavorable
	**Understanding Patient Concerns**	Favorable	Very favorable

Visit 3

AMIE	PCP
**Dialogue with Patient**	

[System Prompt]

Information available at the beginning of the visit

Patient: John Miller, 32 years, Male

Context: John returned for a follow-up visit, after having completed the endoscopy and small bowel biopsy.

Additional information:

Patient Name: John Miller Procedure: Upper gastrointestinal endoscopy with duodenal biopsy

Indication: Suspected celiac disease based on clinical symptoms and serological tests.

Findings:

Normal esophagus and stomach.

Duodenal mucosa shows scalloping, reduced folds, and mosaic pattern.

Villous atrophy

Biopsy Results:

Histopathology reveals intraepithelial lymphocytosis.

Villous blunting and crypt hyperplasia consistent with celiac disease.

Procedure Notes:

Upper endoscope passed smoothly to the duodenum.

Mucosal changes noted in the second part of the duodenum.

Biopsy samples taken from duodenal mucosa for histopathological examination.

Impression: Endoscopic and histopathological findings consistent with celiac disease.

<table>
<tr><td colspan="2">Recommendations:

Start gluten-free diet immediately.

Referral to a dietitian specializing in celiac disease management.

Follow-up with gastroenterologist for further management and monitoring of symptoms and serological markers.

Signed by: Dr. Lucy Melwani</td></tr>
<tr><td>[Patient]

Good afternoon, doctor!

[Doctor]

Good afternoon John! I have your endoscopy and biopsy results. Before we discuss them, how have you been feeling since our last visit? Any changes in symptoms or with the gluten-free diet?

[Patient]

Yea, I've actually noticed some improvement in my symptoms. I find the diarrhea isn't as frequent as it used to be. The pain in my abdomen is also less than before. So yea, feeling a little bit better finally. I didn't think just a few weeks of this would make such a big change already.

[Doctor]

That's wonderful news, John! It's great the gluten-free diet is helping. Let's discuss your test results. The endoscopy and biopsy confirmed celiac disease. The duodenal lining showed characteristic changes: scalloping and reduced folds. The biopsy also showed villous atrophy and intraepithelial</td>
<td>[Patient]

Hi doctor!

[Doctor]

Hi John -- good to chat with you again. How are you doing? Looks like we got some results from the endoscopy and biopsy tests.

[Patient]

Hi doc. I'm doing ok. I did try starting the gluten-free diet like we talked about last time. I've noticed some improvement actually.

[Doctor]

Okay, that's great to hear. I would definitely adhere to the gluten-free diet, and be pretty careful to avoid all gluten going forward. I think it will help you continue to feel better over the next weeks and months going forward. Have you spoken to a dietician or nutritionist about this?

[Patient]

No I haven't. Just started it based on our last conversation. I'm curious about the results though.</td></tr>
</table>

lymphocytosis, confirming the diagnosis. Does this all make sense? Do you have any questions?<br><br>[Patient]<br><br>Well, I don't understand the specifics but as long as we know my diagnosis confidently now, that's all I really want to know. One thing that I'm curious about is my energy levels. When can I expect them to return to normal? Is there anything else I should be supplementing my diet with?<br><br>[Doctor]<br><br>That's a great question! Fatigue is common with celiac disease due to malabsorption. As your gut heals, your energy should improve, though it might take months. We'll monitor your nutrient levels with blood tests to see if supplementation is needed.<br><br>Here's our plan:<br><br>● Referral to a dietitian specializing in celiac disease.<br>● Blood tests to check nutrient levels.<br>● Regular follow-up appointments.<br><br>How does that sound? Does this address your concerns?<br><br>[Patient]<br><br>Yea that all sounds good. Do I have to continue coming for follow ups long term?<br><br>[Doctor]<br><br>Yes, regular follow-ups are important. We'll monitor your symptoms, nutrient levels, and	[Doctor]<br><br>Yes, absolutely -- the endoscopy and biopsy results showed some inflammation and changes to the small bowel/intestines ("duodenum") which were consistent with celiac disease. We had mentioned this as a likely possibility, and I think the results confirm this. This is caused by an immune reaction to gluten in the diet, which causes damage to the small intestines -- there usually isn't any specific treatment aside from strict avoidance of gluten, which usually works very well to reduce symptoms, and also ensure your gut and intestines are working as well as possible, and that no further damage occurs.<br><br>[Patient]<br><br>Oh ok. I've heard of this sort of thing. I'm still a little worried about my lack of energy. Will that get better with these changes? Also, do I have to be on any meds or supplements? Or will the new diet be enough to help?<br><br>[Doctor]<br><br>Good questions! I think your energy will definitely improve as the inflammation goes down over the coming weeks and months. It is something we should carefully watch though, in case it isn't improving. Based on your last blood tests, I don't think you'll need any specific supplements or medications at this time. Though, we should likely repeat these blood tests in a few months to ensure that your nutrition and absorption is adequate to meet your body's needs.<br><br>[Patient]

<table>
<tr>
<td>overall health, adjusting your plan as needed. We'll be checking for:

1. Symptom improvement (diarrhea, pain, fatigue)
2. Nutrient levels (iron, folate, B12)
3. Potential complications (anemia, osteoporosis)
4. Overall well-being

Appointment frequency will depend on your progress. We might start with more frequent visits and gradually space them out.

[Patient]

Alright, sounds like a plan!

[Doctor]

Great! I'm glad we have a plan. The dietitian will be a valuable resource. Please reach out if you have any questions or concerns before your next appointment. Take care!</td>
<td>Oh ok sounds good. Is there anyone I can see to get some help with the new diet? I'd like some ideas since I can't really eat how I usually used to.

[Doctor]

Yes, we can definitely connect you with a dietitian or nutritionist to help, to make sure you can get the right balance of macro/micronutrients/vitamins etc to support your body's needs. We might also work with them to recommend supplementation with calcium and/or vitamin D at that stage based on your diet and needs.

[Patient]

Oh ok sounds good. That's great.

[Doctor]

We should also plan a check-in in the next couple of months to make sure your symptoms (including fatigue) are continuing to improve, and we might recheck some blood tests then as well to make sure you are getting the right nutrition for your needs. Do you have any other questions for me?

[Patient]

No I think this covers everything. Thanks so much!

[Doctor]

Great -- I'm glad we could find an answer, and I think we have a good plan to make you feel even better soon. Take care for now, and we'll check in again shortly!</td>
</tr>
<tr>
<td colspan="2">Responses to Post-Questionnaire</td>
</tr>
</table>

Guidelines Selected as Relevant	
• [bmj122] Irritable bowel syndrome<br>• [bmj636] Coeliac disease<br>• [cg61] Irritable bowel syndrome in adults: diagnosis and management<br>• [ng20] Coeliac disease: recognition, assessment and management<br>• [qs134] Coeliac disease: quality standard	• [bmj636] Coeliac disease<br>• [ng20] Coeliac disease: recognition, assessment and management<br>• [qs134] Coeliac disease: quality standard
Differential Diagnosis	
1. Celiac disease	1. Celiac disease
In-visit Investigations	
None	None
Ordered Investigations	
1. Order a serum ferritin level to assess iron stores and guide iron supplementation adjustments.<br><br>2. Order a complete blood count (CBC) to monitor hemoglobin and hematocrit levels, assessing the effectiveness of iron supplementation and the resolution of anemia.<br><br>3. Order blood tests to check serum levels of folate and vitamin B12 to assess for deficiencies and guide potential supplementation.<br><br>4. Order a bone density scan (DEXA scan) to assess for osteoporosis, a potential long-term complication of celiac disease. This is particularly important given the patient's age and the presence of anemia.	1. Repeat bloodwork (CBC, iron studies / ferritin, vitamin D, B12, folate) in 2-3 months
Recommendations or Actions	
1. Refer the patient to a registered dietitian specializing in celiac disease for personalized dietary guidance, meal planning, and ongoing support. The dietitian should educate the patient on the importance of strict gluten-free	1. Referral to dietician<br><br>2. Discussion with patient and dietician regarding need for additional

diet adherence and strategies for avoiding cross-contamination.<br><br>2. Continue iron supplementation (ferrous sulfate 325mg daily) and advise the patient to take it with Vitamin C to enhance absorption. Monitor for and address any side effects (constipation, nausea).<br><br>3. Continue to strongly emphasize the importance of strict adherence to a gluten-free diet for life. Provide educational materials and resources, and answer any questions the patient has.<br><br>4. Schedule a follow-up appointment in 4-6 weeks to review blood test results (CBC, ferritin, folate, B12, Vitamin D, and DEXA scan), assess symptom progress, and discuss ongoing management. Address any concerns or questions the patient may have.<br><br>5. Educate John on the signs and symptoms of possible long-term celiac disease complications (osteoporosis, increased cancer risk). Provide resources for additional information and advice (e.g., contact information for local support groups or online resources).<br><br>6. Instruct the patient to monitor their symptoms (diarrhea, bloating, abdominal pain, fatigue) and contact the office immediately if symptoms worsen, new symptoms develop (especially bloody stools or fever), or if they experience any side effects from the iron supplementation or other problems with the gluten-free diet.<br><br>7. Referral to gastroenterologist for ongoing monitoring and management of celiac disease in 6-12 months.	supplementation (vitamin D, calcium) based on dietary plans<br><br>3. Check-in with the patient in 2-3 months to review symptoms, diet, and do bloodwork to assess vitamin/mineral levels
Did you intentionally deviate from one of the clinical guidelines you selected above?	

No	Yes
If yes, please explain why (e.g. guideline does not cover this clinical scenario, patient preference, your preference). If no, enter 'N/A':	
N/A	I usually think that I can provide more tailored plans regarding need for additional supplementation after a more in-depth assessment with the dietitian -- that is, whether their planned diet will have adequate levels of key minerals/vitamins that are of particular concern in celiac disease (calcium, vitamin D, b12, folate, iron). Once we've had that assessment, we can come up with a plan as to whether additional supplementation is necessary. Supplementation can be very important, but I'd like to be guided more by the patient's current/future diet, as well as their lab testing (which had showed normal levels of the key vitamins/minerals).
What are other acceptable management plans? Why did you choose yours?	
The management plan largely aligns with established guidelines for celiac disease. However, there's flexibility. For example, some clinicians might opt for a more conservative approach, delaying iron supplementation until after biopsy confirmation. My chosen plan prioritizes addressing the patient's anemia promptly due to its severity (Hb 11.5 g/dL, Hct 35%), even before definitive biopsy results. The potential benefits of early intervention outweigh the risks of delaying treatment, especially considering the patient's significant fatigue. Another approach might involve more frequent monitoring of blood glucose levels, especially given the patient's initial symptoms of diarrhea and bloating. However, given the confirmed diagnosis of celiac disease and the patient's reported symptom improvement on a gluten-free diet, this is less of a priority at this stage.	Referral to the dietitian and then close follow-up are likely the key steps of an acceptable management plan.
Please describe any competing priorities that affected your decisions:	
N/A	None
Describe the cost, effectiveness and side effects of your management plan:	

The cost is moderate, encompassing blood tests (CBC, ferritin, folate, B12, Vitamin D), a DEXA scan, and follow-up appointments. Effectiveness hinges on the patient's adherence to the gluten-free diet and iron supplementation. Side effects are minimal for the tests, but iron supplementation can cause constipation or nausea. The gluten-free diet may initially cause some inconvenience. Long-term effectiveness and cost-effectiveness depend on the patient's adherence and complication management.	This is a relatively low-cost and low-risk plan, and having the patient change their diet will likely have a significant shortterm and longer-term impact on their symptoms and their overall health.
Describe the prognosis with and without treatment of this patient:	
Without treatment, John's prognosis is poor. Untreated celiac disease can lead to severe nutritional deficiencies, anemia, osteoporosis, increased cancer risk, and a significantly decreased quality of life. Addressing the underlying cause is crucial for improving his prognosis.	Excellent prognosis with strict adherence to gluten-free diet. Without treatment, likelihood for worsening of symptoms and downstream and major health impacts on gut health, bone health, overall well-being and quality of life.
Does this case require escalation of this text-based consultation to a video-call or in-person consultation?	
No, does not require escalation	No, does not require escalation
Describe why it requires or does not require escalation:	
The current visit's management plan is appropriate for a text-based consultation. The investigations are standard and readily available. Escalation to a video call or in-person consultation would be considered if the initial test results were inconclusive or revealed alarming findings. However, given the confirmed diagnosis of celiac disease and the patient's reported symptom improvement on a gluten-free diet, a text-based follow-up is sufficient for now.	Diagnosis is established, and treatment plan is relatively straightforward.
Is a follow-up required?	
Yes	Yes
If yes, when would you like to follow-up the patient? Otherwise, enter 'N/A':	
4-6 weeks	2-3 months
Describe why it requires or does not require follow-up:	

To review blood test results (CBC, ferritin, folate, B12, Vitamin D, and DEXA scan), assess symptom progress, and discuss ongoing management, including potential adjustments to iron supplementation and addressing any concerns or questions the patient may have.	Ensure adherence to diet, ensure symptoms are improving, and check-in with follow-up lab tests.

Specialist Physician Ratings

		AMIE	PCP
**Diagnosis & Management**	**Confabulation Absent (Y/N)**	Yes	Yes
	**DDx Appropriateness**	Very favorable	Very favorable
	**DDx Comprehensiveness (4-point scale)**	Very favorable	Very favorable
	**Escalation Recommendation Appropriate (Y/N)**	Yes	Yes
	**Followup Recommendation Appropriate (Y/N)**	Yes	Yes
	**Guideline Entailment Overall**	Very favorable	Very favorable
	**Investigation Aligned With Guidelines**	Very favorable	Very favorable
	**Investigation Appropriate Recommended (Y/N)**	Yes	Yes
	**Investigation Cites Guidelines**	Very favorable	Very favorable
	**Investigation Inappropriate Avoided (Y/N)**	Yes	Yes
	**Investigation Sufficiently Precise (Y/N)**	Yes	Yes
	**Management Aligned With Guidelines (3-point scale)**	Favorable	Favorable
	**Management Free of Clinically Significant Error (Y/N)**	Yes	Yes

	**Management Plan Appropriateness**	Very favorable	Favorable
	**Remembering Important Information From Previous Visits (3-point scale)**	Favorable	Favorable
	**Treatment Aligned With Guidelines**	Very favorable	Very favorable
	**Treatment Appropriate Recommended (Y/N)**	Yes	Yes
	**Treatment Cites Guidelines**	Very favorable	Very favorable
	**Treatment Inappropriate Avoided (Y/N)**	Yes	Yes
	**Treatment Sufficiently Precise (Y/N)**	Yes	Yes
**MXEKF**	**Acting As Patient Teacher And Salesperson**	Very favorable	Very favorable
	**Communication And Shared Decision Making**	Very favorable	Very favorable
	**Contrasting\nand Selection**	Very favorable	Favorable
	**Illness Specific Knowledge**	Very favorable	Very favorable
	**Managing Interplay Of Competing Priorities**	Very favorable	Very favorable
	**Monitoring And Adjustment Of Management Plan**	Very favorable	Very favorable
	**Organization Of The Clinical Encounter**	Very favorable	Very favorable

	**Prioritization Of Preferences Constraints And Values**	Very favorable	Very favorable
	**Prognostication**	Very favorable	Very favorable
	**Relationship Fostering**	Very favorable	Very favorable
PACES	**Addressing Patient Concerns**	Very favorable	Very favorable
	**Clinical Judgement**	Very favorable	Very favorable
	**Differential Diagnosis**	Very favorable	Very favorable
	**Eliciting Family History**	Very favorable	Very favorable
	**Eliciting Medication History**	Very favorable	Very favorable
	**Eliciting Past Medical History**	Very favorable	Very favorable
	**Eliciting Presenting Complaint**	Very favorable	Very favorable
	**Eliciting Systems Review**	Favorable	Very favorable
	**Explaining Relevant Clinical Information Accurately**	Very favorable	Very favorable
	**Explaining Relevant Clinical Information Clearly**	Very favorable	Very favorable
	**Explaining Relevant Clinical Information Comprehensively**	Very favorable	Very favorable

	**Explaining Relevant Clinical Information Professionally**	Very favorable	Very favorable
	**Explaining Relevant Clinical Information With Structure**	Very favorable	Very favorable
	**Maintaining Patient Welfare**	Very favorable	Very favorable
	**Showing Empathy**	Very favorable	Very favorable
	**Understanding Patient Concerns**	Very favorable	Very favorable

Patient Actor Ratings

		AMIE	PCP
**GMCPQ**	**Appearing Honest And Trustworthy**	Cannot rate / Does not apply / Agent did not perform this	Cannot rate / Does not apply / Agent did not perform this
	**Assessing Medical Condition**	Very favorable	Very favorable
	**Being Polite**	Very favorable	Very favorable
	**Explaining Condition and Treatment**	Very favorable	Very favorable
	**Involving Patient in Treatment Decisions**	Very favorable	Very favorable
	**Listening to Patient**	Very favorable	Very favorable
	**Making Patient Feel At Ease**	Very favorable	Very favorable
	**Patient Confident About Care Provided (Y/N)**	Yes	Yes
	**Patient Happy to Return In Future (Y/N)**	Yes	Yes
	**Patient Trusts Information is Confidential**	Cannot rate / Does not apply / Agent did not perform this	Cannot rate / Does not apply / Agent did not perform this
	**Providing Appropriate Treatment Plan**	Very favorable	Very favorable

**MXEKF**	**Acting As Patient Teacher And Salesperson**	Very favorable	Very favorable
	**Communication And Shared Decision Making**	Very favorable	Very favorable
	**Managing Interplay Of Competing Priorities**	Cannot rate / Does not apply / Agent did not perform this	Very favorable
	**Monitoring And Adjustment Of Management Plan**	Very favorable	Very favorable
	**Organization Of The Clinical Encounter**	Very favorable	Very favorable
	**Prioritization Of Preferences Constraints And Values**	Cannot rate / Does not apply / Agent did not perform this	Very favorable
	**Relationship Fostering**	Cannot rate / Does not apply / Agent did not perform this	Very favorable
**PACES**	**Addressing Patient Concerns**	Very favorable	Favorable
	**Maintaining Patient Welfare**	Very favorable	Very favorable
	**Showing Empathy**	Very favorable	Very favorable
	**Understanding Patient Concerns**	Very favorable	Favorable
