# Supplementary material for: Towards conversational artificial intelligence for disease management
Source: Nature. 2026 Jun 17;655(8125):1292–9. doi: 10.1038/s41586-026-10764-5 (PMC13421318; doi:10.1038/s41586-026-10764-5)
Supplement: Supplementary file 6 — Peer Review File [file 41586_2026_10764_MOESM6_ESM.pdf]

nature portfolio

Peer Review File

Towards Conversational AI for Disease Management

Corresponding Author: Dr Mike Schaekermann

Any redactions in this file are there to maintain patient confidentiality, the confidentiality of unpublished data, or to remove third-party material.

This file contains all reviewer reports in order by version, followed by all author rebuttals in order by version.

Version 0:

Reviewer comments:

Referee #1

(Remarks to the Author)
This paper uses the articulate medical intelligence explorer (AMIE) in a randomized blinded virtual objective structured clinical examination study against 21 primary care physicians in 100 multi-visit case scenarios. The authors developed RxQA, a multiple choice question benchmark derived from national drug formularies. AMIE was non-inferior to PCPs on the case evaluation and performed better on RxQA. At this point, there have been many claims around the clinical abilities of models, but one of my biggest concerns is the lack of transparency around the datasets or the model and the complete inability to independently validate these claims.

Comments
Why was the methodological decision made to use Gemini 1.5 Flash instead of PaLM-2?
The scenario packs were prepared by physicians with 100 different scenarios across multiple clinical domains. However, the cases are not shared, so it's not clear how realistic the scenarios were, how "well packaged" the clinical information was.

Since there is no HIPAA here, this study should release both the physician-actor and AMIE-actor conversations so that reviewers can truly evaluate the model's performance. There are many ways to make the results look good without the model being actually clinically useful. The highest level of transparency is sharing the model, but the authors state that they will not do this. The second highest level of transparency is releasing the conversations and the "assessment" of the performance.

Table A.1, this case of pheochromocytoma seems really contrived (which makes me wonder what the other cases are like). This is a rare disease that doesn't usually present in such an obvious way.

Figure A.5: The Mx agent mentions talking about symptoms of heart attack and stroke in a patient with major depressive episode. This does not seem to make sense. Additionally, "asking about side effects" is mentioned, but the specific side effects are not listed.

Looking at BMJ's best practice guidelines in pheochromocytoma, Table A.1 does not appear to include important information like considering whether or not the patient is in hypertensive crisis, considering genetic testing after diagnosis is made. Figure A.3 likewise doesn't mention genetic testing.

There were time intervals between interactions with patient actors. While the models were allowed to "store" data, were the PCPs also allowed to take notes and review them? Physicians don't have to 'remember' every detail of the patient visit, and their personal documentation is an important piece to have.

Very little information on the raters' clinical experience and how they were trained to rate. Looking at figure A.25 – hard to say what the difference is between "slightly" and "somewhat" in rating responses.

Was there any information on inter-rater agreement? Or was only a single rater used?

The Management Reasoning Empirical Key Features (MXEKF) rubric is not externally validated, which is concerning and

difficult to assess.

Will RxQA be released?

There is no information on the experience of the PCPs used in this study.

Was there any overlap between the 20 validation scenarios described in 3.1.1 and the ultimate cases used to test PCPs and AMIE?

(Remarks on code availability)
cannot assess code as nothing is shared.

Referee #2

(Remarks to the Author)
A. Summary of the key results

This work extends the capabilities of the previously proposed Articulate Medical Intelligence Explorer (AMIE), a large language model (LLM) based AI system originally optimized for diagnostic conversations [1], to include disease management functionalities.

The authors evaluate and compare AMIE's disease management performance with primary care physicians (PCPs) in two aspects:

(1) Multi-visit OSCE study: It utilizes a text-chat interface to evaluate AMIE's ability to diagnose and manage patients over a longitudinal period, which helps assess its capability in adjusting management plans as patient information evolves. Results show that AMIE's disease management plans are non-inferior to those of PCPs, are better aligned with clinical guidelines, and are preferred by both patients and physicians.

(2) Medication knowledge and reasoning: Through a multiple-choice question benchmark called RxQA, the authors evaluate and compare AMIE's and PCPs' ability to prescribe medications. Results show that AMIE significantly outperforms PCPs on higher-difficulty questions, and achieves comparable performance on lower-difficulty questions.

Overall, the results indicate that AMIE's disease management abilities are comparable or superior to those of PCPs within simulated clinical scenarios.

B. Originality and Significance

Previous research on task-oriented conversational AI in healthcare has predominantly focused on advancing diagnostic abilities. In contrast, this study extends the application of LLM-based systems to disease management, which requires the ability to track disease progression, formulate treatment plans and prescriptions, and monitor therapeutic responses over multiple patient visits.

In my opinion, the significance of this work lies in the positive results on OSCE-based evaluations. Objective Structured Clinical Examinations (OSCEs) are widely recognized as a standard for evaluating clinical competencies among medical students and for medical licensing exams around the world. Therefore, the positive OSCE results in this study represent a significant step towards integrating AI systems into clinical practice for disease management. Although the current evaluations were limited to a text-based conversational interface—indicating the necessity for further real-world studies—these results provide preliminary evidence for subsequent clinical validation.

C. Data and Methodology

(1) Validity of approach: The design of using two agents—one "fast-thinking" dialogue agent to ensure quick conversational responses and one "slow-thinking" Mx agent for spending more inference-time compute on management reasoning—is well-motivated. **However, I have the following question (Q1) for the authors:** The conversation between AMIE and the patient actor is synchronous, while the agent state fields are updated asynchronously. Wouldn't this design introduce information mismatch between the agent state and the current dialogue turn? Ideally, to ensure optimal dialogue quality, the dialogue agent should have access to the most recent patient information up to the current dialogue turn.

(2) Validity of data:

a. Training data are mainly used to fine-tune the base model (i.e., Gemini 1.5 Flash) of the dialogue agent. The authors use simulated dialogues, real-world medical dialogues, medical question-answering (QA), and EHR summarization datasets for supervised fine-tuning (SFT). Following SFT, they use preference pairs of dialogue responses and case management recommendations to perform reinforcement learning with human / AI feedback (RLHF / RLAIF). Among the training data, only some datasets for medical QA and EHR summarization (MedQA, MultiMedQA, MIMIC-III) are open-source. Therefore, the quality of medical dialogues and preference pairs cannot be verified directly.

b. Testing data for multi-visit OSCE study consist of 100 scenarios, which were prepared by clinical providers in Canada and India. The scenarios encompass clinical cases from five different medical specialties to ensure diversity. Since the scenarios are not openly available, I cannot verify the validity of these data. As for testing data in RxQA medication benchmark, 600 questions are generated by Gemini 1.5 Flash based on medication labels from OpenFDA and British National Formulary (BNF), and the questions are further refined by board-certified pharmacists. **Since the base model of AMIE is also Gemini 1.5 Flash, this naturally raises the question (Q2) of whether Gemini 1.5 Flash has the bias to generate questions that can be answered by itself.** This bias would potentially make the findings in Figure 7 less convincing. Could the authors elaborate on the choice to use the same LLM for generating the questions? Did the authors experiment with other LLMs for question generation?

(3) Quality of presentation:

Overall, the manuscript is well-organized and clearly written. The detailed presentation of the Mx agent, including specific prompts and illustrative model outputs in Appendices A.1 through A.4, is particularly commendable. However, although the authors include prompts (Appendices A.7 and A.8) used for generating the dialogue agent's training data, actual examples of corresponding model outputs are absent. Similarly, examples of dialogues between AMIE and patient actors are also missing. Including representative model outputs corresponding to the prompts in Appendices A.7 and A.8, as well as sample dialogues between AMIE and patient actors, would facilitate a more comprehensive understanding of the work.

D. Appropriate Use of Statistics

The McNemar test is used for the evaluation axes in multi-visit OSCE and the accuracy on the RxQA benchmark, with p-values adjusted using false discovery rate (FDR) correction. As the data are paired (AMIE vs. PCPs for the same questions or evaluation items) and results are binarized, the statistical tests are appropriate. Error bars are defined in the corresponding figures.

E. Conclusions

Overall, the conclusions presented are valid and supported by the data. However, a previously noted concern remains regarding the RxQA medication benchmark, where the questions were generated by Gemini 1.5 Flash—the same base model underlying AMIE. This choice introduces a potential bias that may affect the robustness of findings in Figure 7. Clarification on the rationale behind using the same model and any experimentation with alternative models would be great.

F. Suggested Improvements

Following the previously raised concerns, I suggest the authors clarify the following aspects in the revised manuscript:

1. Agent state synchronization: The dialogue between AMIE and patient actors occurs synchronously, yet the agent's state fields are updated asynchronously. Could this design potentially lead to information mismatches between the agent state and the latest dialogue turn? If so, how would such mismatches influence conversational quality?

2. RxQA benchmark generation: The RxQA medication benchmark questions were generated using Gemini 1.5 Flash, which is also the base model of AMIE. Explain the motivation behind using the same model for question generation and whether alternative LLMs were considered or tested.

3. Additional illustrative examples: Include representative model outputs for the training data prompts (Appendices A.7 and A.8), as well as example dialogues between AMIE and patient actors.

G. References

The manuscript appropriately references relevant prior literature and situates its contributions within existing research on medical conversational AI.

H. Clarity and Context

The abstract, introduction, and conclusions are appropriate and contextualize the study's contributions and significance.

* References mentioned in this review:
[1] Tu, T., Palepu, A., Schaekermann, M., Saab, K., Freyberg, J., Tanno, R., ... & Natarajan, V. (2024). Towards conversational diagnostic AI. arXiv preprint arXiv:2401.05654.

(Remarks on code availability)
The authors do not provide the code in the submission.

Referee #3

(Remarks to the Author)
Review of Lievin et al. "Towards Conversational AI for Disease Management"

Lievin et al. build upon the same team's previous work (Tu et al. Nature 2025) that introduced the Articulate Medical Intelligence Explorer (AMIE), a conversational AI system designed to engage in dialogue with patients. While prior evaluations of AMIE have focused on diagnostic reasoning, the current analysis focuses on the disease management reasoning capabilities of AMIE. The topic of management reasoning of AMIE and of management reasoning by LLMs more broadly is timely and interesting but the present study, as reported, makes it hard to understand the critical question of where the reported management reasoning capabilities arise (from the new base LLM, post-training, or other modifications?) and thus reduces some of my enthusiasm. Major comments:

1. This version of AMIE is not the same as the version of AMIE introduced by Tu et al. in Nature earlier this year. For example, in Section 2.1.1 on post-training, the authors describe some of the changes to the Dialogue Agent, which is most comparable with the AMIE system in Tu et al. Nature 2025. First, the authors use Gemini 1.5 Flash instead of PaLM-2. Second, the authors "developed a new set of simulated dialogues...used to fine-tune the base model..." Third, the authors employ SFT/RLHF/RLAIF, however details are sparse. Most importantly, without rigorous ablation studies that compare AMIE to the new base LLM(s) and tease out the effects of individual components of the system (e.g. dual-agent Mx Agent and Dialogue Agent setup, new dialogues used in fine-tuning, SFT/RLHF design choices..etc.), it is very difficult to understand whether the management reasoning capabilities reported in the manuscript arise from the advancing capabilities of the base LLM(s) or the medical scaffolding in the AMIE system that is layered on top. This gets to the heart of a major debate (how much specialization is needed to use LLMs in medical contexts) that will be of substantial interest to readers of this paper and to the broader AI community, and is necessary to properly understand the major contributions in this manuscript.

2. Relatedly, the present manuscript would be strengthened by comparing AMIE to any or several of a readily available number of leading large language models (e.g. OpenAI o-series, Meta's Llama, newer versions of Gemini) or other medical-specialized LLMs, several of which have demonstrated strong performance on a broad array of other benchmarks including those related to management reasoning.

3. Strengths of the study include rich, multi-dimensional evaluations of management reasoning as well as a randomized study using Objective Structure Clinical Examination (OSCE) scenarios. At the same time, there are a very large number of effects and p-values reported across visits and evaluation axes from these experiments, and I am both concerned about multiplicity and unsure how some of the significance calculations were performed (e.g. for "providing appropriate follow-up recommendations (100% vs. 98%, $p<0.001$)"). Could the authors clarify the statistical reporting generally and how the FDR-adjustment was calculated specifically [e.g. across all tests performed in the OSCE study or some subset for each sub-analysis..etc.]?

4. The authors assess AMIE and PCPs based in part on clinical guidelines (e.g. items "Selected Applicable Guidelines" or "Aligned with Guidelines" in Figure 5) — the guidelines appears to be entirely from the UK but the 21 PCPs who participated in the study are based in India and Canada — is this a fair comparison for AMIE vs. PCPs in guideline use, or would PCPs do better if evaluated with respect to local guidelines? How might this affect generalizability?

5. Section 2.2.2 on long-context reasoning is interesting and could be expanded in the main manuscript.

6. The authors describe some of the changes to AMIE to create the management focused AMIE system in the present manuscript, but overall engineering details are sparse, model code and weights are not released, and the system is inaccessible. It is thus challenging to know how reproducible and sensitive the core findings of the manuscript are.

(Remarks on code availability)
Not made available by authors.

Version 1:

Reviewer comments:

Referee #1

(Remarks to the Author)
Thank you for the access to the model and the responses to the reviews.
Regarding reasoning traces of the model, you have responded, "Note that interrogation of reasoning traces allows inspection of latent reasoning errors that are not propagated into the final (appropriate) Mx Plan. In this case, a reasoning trace inspection reveals that AMIE's intermediate reasoning analysis suggests educating the patient about emergency symptoms of heart attack and stroke which are not clinically relevant in this context."
I think it's concerning that there are latent reasoning errors in the reasoning traces. How often does this happen? How often does this lead to a correct or incorrect answer?

Thank you for sharing the scenarios and the PCP conversations. As a physician, the first scenario's PCP seems highly questionable in how they are acting for this scenario – it does not seem like how an actual doctor would interact with a patient. In the rheumatoid arthritis case, the physician jumps to the conclusion immediately without asking further inquiries. I have shown this example to other physicians who agree that it's concerning what the "baseline" for physicians were. For the second scenario, the doctor seems much more in line with physician behavior. Since the baseline DOES matter, it would be

good to release not only all scenarios but how they are graded.

I did converse with the model myself – it was very slow and clunky and difficult to get through a scenario because of how long inference took. It asked a lot of questions, reminding me of a medical student without diagnostic abilities, rather than discerning questions that would help take it down a path of a differential diagnosis.

(Remarks on code availability)
The inference on the interface was very slow, which made it difficult to stick with a scenario. The answers seemed like a medical student more so than a physician.

Referee #2

(Remarks to the Author)
A. Summary of the key results

My original comments:““““

This work extends the capabilities of the previously proposed Articulate Medical Intelligence Explorer (AMIE), a large language model (LLM) based AI system originally optimized for diagnostic conversations [1], to include disease management functionalities.

The authors evaluate and compare AMIE’s disease management performance with primary care physicians (PCPs) in two aspects:

(1) Multi-visit OSCE study: It utilizes a text-chat interface to evaluate AMIE’s ability to diagnose and manage patients over a longitudinal period, which helps assess its capability in adjusting management plans as patient information evolves. Results show that AMIE’s disease management plans are non-inferior to those of PCPs, are better aligned with clinical guidelines, and are preferred by both patients and physicians.

(2) Medication knowledge and reasoning: Through a multiple-choice question benchmark called RxQA, the authors evaluate and compare AMIE’s and PCPs’ ability to prescribe medications. Results show that AMIE significantly outperforms PCPs on higher-difficulty questions, and achieves comparable performance on lower-difficulty questions.

Overall, the results indicate that AMIE’s disease management abilities are comparable or superior to those of PCPs within simulated clinical scenarios.

””””

Authors’ response:

““““

We thank R2 for their thoughtful review of our manuscript and address the reviewer’s comments inline below.

””””

My comments to authors’ response:

““““

Thanks for the careful, point-by-point response to my comments provided below.

””””

B. Originality and Significance

My original comments:

““““

Previous research on task-oriented conversational AI in healthcare has predominantly focused on advancing diagnostic abilities. In contrast, this study extends the application of LLM-based systems to disease management, which requires the ability to track disease progression, formulate treatment plans and prescriptions, and monitor therapeutic responses over multiple patient visits.

In my opinion, the significance of this work lies in the positive results on OSCE-based evaluations. Objective Structured Clinical Examinations (OSCEs) are widely recognized as a standard for evaluating clinical competencies among medical students and for medical licensing exams around the world. Therefore, the positive OSCE results in this study represent a

significant step towards integrating AI systems into clinical practice for disease management. Although the current evaluations were limited to a text-based conversational interface—indicating the necessity for further real-world studies—these results provide preliminary evidence for subsequent clinical validation.

”””

Authors’ response:

“““

We appreciate R2’s assessment that the positive results from our OSCE-based evaluations are of particular significance to this work. In particular, we expanded on OSCE study methods from Tu et al. 2025 by introducing a multi-visit longitudinal variant of this OSCE approach, incorporating the use of clinical guidelines, to evaluate the performance of our technical contribution. We thank the reviewer for recognizing the particular significance of the methodological contribution in this work.

”””

My comments to authors’ response:

“““

Thank you for the response. I have no further concerns about the originality and significance of this work.

”””

C. Data and Methodology

My original comments and authors’ responses:

“““

(1) Validity of approach: The design of using two agents—one “fast-thinking” dialogue agent to ensure quick conversational responses and one “slow-thinking” Mx agent for spending more inference-time compute on management reasoning—is well-motivated. However, **I have the following question (Q1) for the authors: The conversation between AMIE and the patient actor is synchronous, while the agent state fields are updated asynchronously. Wouldn’t this design introduce information mismatch between the agent state and the current dialogue turn?** Ideally, to ensure optimal dialogue quality, the dialogue agent should have access to the most recent patient information up to the current dialogue turn.

Authors’ response: We thank R2 for raising this important question. In the revised manuscript, we have further emphasized that the dialogue agent indeed does have the full conversation transcript including all patient information available at each turn of the conversation (added “full dialogue history up to that point” in Section 2.1.2 Chain-of-Reasoning). In addition, the dialogue agent updates its state object in a synchronous manner. The only part being updated in an asynchronously every few turns is the management (Mx) plan produced by the Mx Agent. This plan helps steer the dialogue agent’s line of inquiry throughout the conversation.

We acknowledge that due to the interleaved and asynchronous nature of tool calling in this context, the latest generated Mx plan may lag behind by a few conversation turns. These asynchronous updates are a necessary tradeoff to enable acceptable latency.

We observed that, in the majority of turns, the Mx plan remains stable. In addition, the dialogue agent is only conditioned on the output of the Mx Agent; therefore, it retains the flexibility to adjust its response based on the latest conversation state. Finally, note that at the end of the conversation, a final Mx plan is computed based on the full information shared throughout the conversation. This allows us to surface AMIE’s final clinical assessments such as differential diagnosis, investigation, and treatment recommendations, which are used to inform post-questionnaire responses.

(2) Validity of data:

a. Training data are mainly used to fine-tune the base model (i.e., Gemini 1.5 Flash) of the dialogue agent. The authors use simulated dialogues, real-world medical dialogues, medical question-answering (QA), and EHR summarization datasets for supervised fine-tuning (SFT). Following SFT, they use preference pairs of dialogue responses and case management recommendations to perform reinforcement learning with human / AI feedback (RLHF / RLAIF). Among the training data, only some datasets for medical QA and EHR summarization (MedQA, MultiMedQA, MIMIC-III) are open-source. Therefore, the quality of medical dialogues and preference pairs cannot be verified directly.

Authors’ response: This is an accurate summary of the datasets involved in model development. As R2 correctly points out, parts of the training data are open-source, as described in the Data and Code Availability sections of the manuscript. Unfortunately, we are unable to share the other parts of the training data or model weights due to the commercial nature of these artifacts. We thank R2 for requesting “representative model outputs corresponding to the prompts in Appendices A.7

and A.8" (in other comment below) which we have provided in the revision.

b. Testing data for multi-visit OSCE study consist of 100 scenarios, which were prepared by clinical providers in Canada and India. The scenarios encompass clinical cases from five different medical specialties to ensure diversity. Since the scenarios are not openly available, I cannot verify the validity of these data. As for testing data in RxQA medication benchmark, 600 questions are generated by Gemini 1.5 Flash based on medication labels from OpenFDA and British National Formulary (BNF), and the questions are further refined by board-certified pharmacists. **Since the base model of AMIE is also Gemini 1.5 Flash, this naturally raises the question (Q2) of whether Gemini 1.5 Flash has the bias to generate questions that can be answered by itself.** This bias would potentially make the findings in Figure 7 less convincing. Could the authors elaborate on the choice to use the same LLM for generating the questions? Did the authors experiment with other LLMs for question generation?

Authors' response: We address R2's two comments regarding OSCE scenario data and the RxQA benchmark separately below.

OSCE scenario data:

In the revised manuscript, we provide metadata (specialty category, location, condition, relevant clinical guidelines, difficulty type) for all 120 scenarios, including the 20 scenarios used for validation and the 100 scenarios used in the OSCE study (Appendix Section A.16 Scenarios Overview).

In addition, we have added a full representation of two sample scenarios, including all OSCE scenario details, patient-actor-AMIE and patient-actor-PCP conversations, as well as post-questionnaire responses from both AMIE and PCP for all three visits in the scenario. Due to the extensive content (over 70 pages), these materials are provided as a separate PDF file including its own table of contents, overview of the data and commentary from qualitative review. We hope that this added information may provide avenues to enable independent validation of our claims.

RxQA benchmark:

We have now released the RxQA benchmark dataset to the public at https://github.com/Google-Health/rxqa. This release includes the part of the benchmark derived from OpenFDA drug labels (300 questions). Due to licensing restrictions, the BNF portion cannot be shared directly by Google. However, we have shared this portion with BNF itself, and the interested reader is encouraged to contact BNF to request access to the data.

In the revised manuscript, we have also added Appendix Table A.13 with new comparisons of AMIE to currently available LLMs on RxQA, including DeepSeek-V3, o-3, GPT-5, Gemini (1.5 Flash, 2.5 Flash, 2.5 Pro), as well as a variant of AMIE based on the newer Gemini 2.5 Pro.

Comparisons with Gemini models are provided for the full RxQA benchmark, including both OpenFDA and BNF portions. Due to licensing restrictions on the BNF portion, the comparison to non-Google models (DeepSeek-V3, o-3, GPT-5) was possible only on the OpenFDA portion.

Results show that access to drug labels (in the open-book setting) consistently increases accuracy for all models compared to the closed-book setting (without access to drug labels). This finding validates the key purpose of this benchmark to test for the ability to retrieve and use information from authoritative drug formularies to inform medication reasoning. GPT-5 performed slightly worse than AMIE (2.5 Pro) on the closed-book setting (71.7% vs. 74.0%), but both are on par in the open-book setting (76.3% for both models).

We thank R2 for raising the valid question of whether Gemini 1.5 Flash has the bias to generate questions that can be answered by itself.

For question generation, we were unable to leverage competitor models due to legal considerations. We did explore using Gemini 1.5 Pro, but qualitatively found little difference in the quality and style of the questions. Thus, we selected Gemini 1.5 Flash, a smaller and less computationally expensive model. We agree with the reviewer that following a similar process with other families of models (GPT, Claude, etc.) could improve question diversity, and we encourage others to follow the question generation process in the appendix with other base model choices.

While the point about potential bias in RxQA is valid, a few technical details help mitigate this risk, and we hope that our benchmarking results with competitor models help address the concern. Firstly, in our filtering process, we exclude generations that Gemini 1.5 Flash was able to answer zero-shot without context. Secondly, the pharmacists not only made revisions to the questions and answer choices, but also selected the correct answer without knowledge of what the model thought the correct answer was. Thirdly, for the longer style of questions, which makes up two-thirds of the RxQA dataset, questions were generated through a complex multi-step process. Finally, our benchmarking results with competitor models — in particular, superior performance of DeepSeek-V3, o-3, GPT-5 compared to base Gemini 1.5 Flash, as well as parity between GPT-5 on and AMIE (2.5 Pro) in the open-book setting — demonstrate that the benchmark does not necessarily favor Gemini models.

(3) Quality of presentation:

Overall, the manuscript is well-organized and clearly written. The detailed presentation of the Mx agent, including specific prompts and illustrative model outputs in Appendices A.1 through A.4, is particularly commendable. However, although the authors include prompts (Appendices A.7 and A.8) used for generating the dialogue agent's training data, actual examples of corresponding model outputs are absent. Similarly, examples of dialogues between AMIE and patient actors are also missing. Including representative model outputs corresponding to the prompts in Appendices A.7 and A.8, as well as sample dialogues between AMIE and patient actors, would facilitate a more comprehensive understanding of the work.

Authors' response: We thank R2 for encouraging sharing of sample dialogues between patient actors and AMIE or PCPs, as well as representative model outputs for Appendices A.7 and A.8.

Along with this revision, we provide a detailed view of two sample scenarios, including all OSCE scenario details, patient-actor-AMIE and patient-actor-PCP conversations, as well as post-questionnaire responses from both AMIE and PCP for all three visits in the scenario. Due to the extensive content (over 70 pages), these materials are provided as a separate PDF file including its own table of contents, overview of the data and commentary from qualitative review.

To give a sense of the case distribution overall, we also provide metadata (specialty category, location, condition, relevant clinical guidelines, difficulty type) for all 120 scenarios, including the 20 scenarios used for validation and the 100 scenarios used in the OSCE study (Appendix Section A.16 Scenarios Overview).

Finally, we have added representative model outputs corresponding to the prompts in Appendices A.7 and A.8 to facilitate a more comprehensive understanding of the work, including outputs for synthetic rewrites of single-visit dialogues (Figure A.15), example outputs for multi-visit dialogue generation (Figure A.19), and example outputs from the auto-evaluation process used to filter dialogues during training (Figure A.22).

”””

My comments to authors' response:

“““

Thank you for the point-by-point responses. For (1) validity of approach, the authors have addressed my concerns with reasonable rationales and clarifications. For (2) validity of data, authors provide comprehensive and acceptable responses. For (3) quality of presentation, I really appreciate authors' efforts in making AMIE's outputs and sample OSCE scenarios available for inspection, and I encourage the authors to make all these information public as well.

”””

D. Appropriate Use of Statistics

My original comments:

“““

The McNemar test is used for the evaluation axes in multi-visit OSCE and the accuracy on the RxQA benchmark, with p-values adjusted using false discovery rate (FDR) correction. As the data are paired (AMIE vs. PCPs for the same questions or evaluation items) and results are binarized, the statistical tests are appropriate. Error bars are defined in the corresponding figures.

”””

Authors' response: “““We appreciate R2's careful review of our statistical approach.”””

My comments to authors' response: “““Thank you. I have no further comments on the use of statistics.”””

E. Conclusions

My original comments:

“““

Overall, the conclusions presented are valid and supported by the data. However, a previously noted concern remains regarding the RxQA medication benchmark, where the questions were generated by Gemini 1.5 Flash—the same base model underlying AMIE. This choice introduces a potential bias that may affect the robustness of findings in Figure 7. Clarification on the rationale behind using the same model and any experimentation with alternative models would be great.

”””

Authors' response:

""""

We agree with this notion and hope that our response above serves to address these considerations.

""""

My comments to authors' response:

""""

The authors have addressed my concern regarding RxQA with reasonable evidence.

""""

F. Suggested Improvements

My original comments:

""""

Following the previously raised concerns, I suggest the authors clarify the following aspects in the revised manuscript:

1. Agent state synchronization: The dialogue between AMIE and patient actors occurs synchronously, yet the agent's state fields are updated asynchronously. Could this design potentially lead to information mismatches between the agent state and the latest dialogue turn? If so, how would such mismatches influence conversational quality?

2. RxQA benchmark generation: The RxQA medication benchmark questions were generated using Gemini 1.5 Flash, which is also the base model of AMIE. Explain the motivation behind using the same model for question generation and whether alternative LLMs were considered or tested.

3. Additional illustrative examples: Include representative model outputs for the training data prompts (Appendices A.7 and A.8), as well as example dialogues between AMIE and patient actors.

""""

Authors' response: """"We refer the reviewer to our response above.""""

My comments to authors' response: """"The authors have made the suggested improvements. I have no further suggestions for now.""""

G. References

My original comments:

""""

The manuscript appropriately references relevant prior literature and situates its contributions within existing research on medical conversational AI.

""""

Authors' response: """"We thank R2 for reviewing related work and appreciate their assessment.""""

My comments to authors' response: """"I thank authors for their response.""""

H. Clarity and Context

My original comments:

""""

The abstract, introduction, and conclusions are appropriate and contextualize the study's contributions and significance.

""""

Authors' response:

""""

We are delighted about R2's assessment with respect to clarity and context of our abstract, introduction and conclusion sections.

"""

My comments to authors' response: """I have no further comments."""

Reviewer signature: Cheng-Kuang Wu

(Remarks on code availability)
The authors have made the repository of RxQA public. However, they were unable to provide training data or model weights due to commercial reasons. I have inspected the FDA subset of RxQA and checked its quality.

Referee #3

(Remarks to the Author)
1. The revised manuscript is substantially improved; in particular I appreciate that the authors conducted a new ablation analysis and made some of the resources available.

2. At the same time, the ablation analysis receives almost no attention in the main manuscript and does not inform the interpretation of the results, despite calling into question some of the central findings. As seen in the supplement, using Base 2.5 Flash shows substantial performance gains (Table A.9 in Section A.17) across the board, substantially outperforming AMIE 1.5 Flash. Moreover, in many evaluation subcategories (e.g. "Elicits Medication History"), the improvements with CoR and/or Mx Agent are minimal. Does the ablation analysis not call into question several central claims of the manuscript, e.g. that the post-training and complicated multi-agent setup are necessary to achieve the main results? Is AMIE already supplanted simply by newer versions of the base LLM? At the least, the authors should reference and discuss the ablation analysis. I think the manuscript will be improved by trying to wrestle with these questions in the Discussion and by tempering the claims.

3. Similarly, the authors do not wrestle or even discuss the striking results presented in Table A.13 in the main manuscript. Gemini 2.5 Pro roughly matches AMIE (2.5 Pro) on the OpenFDA portion and BNF portion of the RxQA benchmark – does this not imply minimal value in AMIE for contemporary versions of the base model? o3 and GPT-5 are also roughly comparable to AMIE (2.5 Pro), again a crucial finding that is not addressed in the manuscript.

4. While the authors have released RxQA and prompts in A.7 and A.8, it remains very hard to judge or to learn from the core technical contributions with what has been released. Overall, it remains unclear to me whether AMIE post-training and the multi-agent setup is necessary to achieve the core results presented here given newer base models.

(Remarks on code availability)
Detailed code is not shared.

Version 3:

Reviewer comments:

Referee #1

(Remarks to the Author)
Thank you for your response. I do not feel that my major concerns are fully addressed.
1) I think the reasoning trace issue is still a major one - errors in reasoning traces that still lead to the "right answer" means that there will definitely be cases where it leads to the wrong answer. If we are arguing that this will be used in disease management, this seems like an important thing to fully address.
2) I'm still really unable to assess this manuscript without actually being able to see the scenarios/responses from the models and physician baselines. The two cases shown make me wary of whether or not there could be issues in the scenarios and baselines. Since this paper is making huge claims, it feels like there should be more transparency here. I would not use or suggest implementation of such a tool without more transparency. Such openness would also move the field forward.

(Remarks on code availability)
Previously reviewed the shared model, had issues with the model acting slow.

Referee #2

(Remarks to the Author)
The manuscript is substantially improved after the second revision, and I'd like to comment on the following aspects:

1. Based on the empirical findings in Section A.17 and A.18, it appears that advancements in newer foundation LLMs (e.g., Gemini-2.5-Flash) are the primary driver of performance gains on clinical tasks. In particular, later base LLMs without AMIE often outperform earlier LLMs augmented with AMIE, and the additional benefits of AMIE on top of newer LLMs (e.g., Gemini 2.5 Pro) tend to show diminishing returns, as evidenced by Table A.13. Although this observation may somewhat diminish the perceived contribution of AMIE itself, I believe it is still an important and useful finding, as it helps clarify where the gains are actually coming from and provides a more accurate picture of the respective roles of base-model progress and post-training / scaffolding.

2. The OSCE scenarios added in A.16 span diverse and common diseases, and their full release would be a valuable contribution for developing and evaluating future medical AI systems. I encourage the authors to release the complete contents of all 120 OSCE scenarios (ideally presented in the same format as the two samples in the Supplementary Material) in addition to the metadata.

3. On the evaluation methodology, the benchmarks and rubrics of (1) multi-visit OSCE study and (2) medication knowledge (RxQA) would be useful for improving disease management abilities of future models. The RxQA benchmark has been released publicly, and I encourage the authors to release full details of 120 OSCE scenarios in addition to their metadata.

Overall, I believe this work represents a meaningful step towards evaluating disease management abilities of AI systems, especially given the fact that previous works have largely focused on evaluating and advancing diagnostic abilities. The multi-visit OSCE design also aligns more closely with real-world clinical practice than previous benchmarks.

Reviewer signature: Cheng-Kuang Wu

(Remarks on code availability)
Source code is not provided

Referee #3

(Remarks to the Author)
I commend the authors for engaging substantively with the feedback and think the manuscript has improved considerably -- I have no further comments.

(Remarks on code availability)

Version 4:

Reviewer comments:

Referee #1

(Remarks to the Author)
Thank you for agreeing to release the 120 scenarios. I believe this will be an excellent contribution.

(Remarks on code availability)

Referee #2

(Remarks to the Author)
I have read through the authors' response to my comments as well as the other reviews, and I believe the concerns raised in previous rounds of review have been sufficiently addressed. Specifically, the authors have agreed to release all OSCE prompts and AMIE responses, and I found their discussion of the latent reasoning errors reasonable. Completely eliminating reasoning errors in language models is currently infeasible. Therefore, as long as the research provides insightful findings or useful data toward alleviating this issue and clearly flags this limitation, I consider it a solid contribution to this line of research. Building on this point, I believe the release of all OSCE prompts and AMIE responses will be valuable to future researchers seeking to investigate latent reasoning errors further.

Overall, I consider the current manuscript ready for publication.

Reviewer signature: Cheng-Kuang Wu

(Remarks on code availability)
The authors are not able to release training data or model weights due to commercial reasons.

Referee #3

(Remarks to the Author)
I believe the authors have engaged thoughtfully with the reviewer feedback and I have no further comments.

(Remarks on code availability)

Open Access This Peer Review File is licensed under a Creative Commons Attribution 4.0 International License, which permits use, sharing, adaptation, distribution and reproduction in any medium or format, as long as you give appropriate credit to the original author(s) and the source, provide a link to the Creative Commons license, and indicate if changes were made.
In cases where reviewers are anonymous, credit should be given to 'Anonymous Referee' and the source.
The images or other third party material in this Peer Review File are included in the article's Creative Commons license, unless indicated otherwise in a credit line to the material. If material is not included in the article's Creative Commons license and your intended use is not permitted by statutory regulation or exceeds the permitted use, you will need to obtain permission directly from the copyright holder.
To view a copy of this license, visit https://creativecommons.org/licenses/by/4.0/

Response to the Referees

Response to the Editor
Response to R1
Response to R2
Response to R3

List of documents

This document provides inline responses to comments from the three external referees as well as the Editor. In addition, we also provide the following documents as part of the revision:

1. **Cover letter**
2. **Revised manuscript** with tracked changes (one color per referee)
3. **Revised appendix** with tracked changes (one color per referee)
4. **Detailed view of two sample scenarios** (70+ pages, with separate table of contents)
5. **Instructions for interactive access to the system**
6. **Updated checklists** (editorial policy, reporting summary, software policy)

Summary of changes

In addition to the inline responses below, we provide a summary of the key changes included in this revision. We believe that the feedback from the Editor and referees has helped us to strengthen the manuscript significantly and hope that the additional information provided may help to enable independent validation of our claims:

1. **Agentic ablation analysis:** We provide a detailed ablation analysis of the system based on an entirely AI-simulated end-to-end re-creation of our multi-visit OSCE study. Results are provided in Appendix Section A.17.

2. **Comparison to other LLMs:** We have added comparisons of AMIE to currently available LLMs on the RxQA medication reasoning benchmark, including DeepSeek-V3, o-3, GPT-5, Gemini (1.5 Flash, 2.5 Flash, 2.5 Pro), as well as a variant of AMIE based on the newer Gemini 2.5 Pro. Results are provided in Appendix Table A.13.

3. **Sharing of OSCE scenarios and dialogues:** We provide metadata (specialty category, location, condition, relevant clinical guidelines, difficulty type) for all 120 scenarios,

including the 20 scenarios used for validation and the 100 scenarios used in the OSCE study (Appendix Section A.16 Scenarios Overview). In addition, we provide a full representation of two sample scenarios, including all OSCE scenario details, patient-actor-AMIE and patient-actor-PCP conversations, as well as post-questionnaire responses from both AMIE and PCP for all three visits in each scenario. Due to the extensive content (over 70 pages), these materials are provided as a separate PDF file including its own table of contents, overview of the data and commentary from qualitative review.

4. **Interactive access to the system:** We have provisioned username/password combinations and a web link to provide access to an interactive version of AMIE as tested by patient actors in the OSCE study. The interactive access will allow reviewers to try out the system at different parts of the scenario sequence, including interactions starting with the 1st, 2nd and 3rd visit respectively.

5. **Release of data, pseudocode, prompts:**

    a. **Benchmark data:** We have released the RxQA benchmark dataset to the public at https://github.com/Google-Health/rxqa. This release includes the part of the benchmark derived from OpenFDA drug labels (300 questions total). Due to licensing restrictions, the BNF portion cannot be shared directly by Google. However, we have shared this portion with BNF itself, and the interested reader is encouraged to contact BNF to request access to the data.

    b. **Pseudocode and prompts:** Our original submission contains detailed prompts and data classes used to build the agentic architecture, as well as representative model outputs for some, but not all prompt sections. We thank R2 for requesting "representative model outputs corresponding to the prompts in Appendices A.7 and A.8" which we have provided in the revision.

6. **Improvements to methods and results:**

    a. We have expanded the results from single-specialist to **triplicate-specialist** ratings per case, and provide an inter-rater reliability analysis in Appendix A.14.

    b. In response to R3, we provide a detailed **elaboration on our statistical approach**, and have resolved one inconsistency identified in the process.

    c. We provide metadata (geographic location, specialty category and years of post-residency experience at the time of study) for each of the 30 specialist physician raters and 21 PCP participants involved in the OSCE study.

    d. Several smaller changes to the manuscript to address all reviewer comments in a comprehensive manner. These are mentioned in our inline responses.

Response to the Editor

Dear Dr Schaekermann

Your manuscript, "Towards Conversational AI for Disease Management", has now been seen by 3 referees, whose comments are attached below. While they find your work of potential interest, as do we, they have raised important concerns that in our view need to be addressed before we can consider publication in Nature.

We would ask that a revised study address the concerns of the reviewers, with specific attention given to the following points:

Response: We thank the Editor and reviewers for their consideration of our manuscript and their thoughtful feedback and comments, which have enabled us to make substantial improvements to the manuscript. We have addressed requests and comments inline below, as well as through corresponding revisions to the manuscript.

1) We would ask that the revised version of the study provide the agentic ablation analysis requested by reviewer #3, and that there be a clear demonstration of which changes between the original AMIE and the version of AMIE here account for the management reasoning performance.

Response: In the revision, we include an agentic ablation analysis based on an entirely AI-simulated re-creation of our OSCE study (Appendix Section A.17). In the ablation analysis, we compare 12 configurations of a doctor agent ranging from the base Gemini 1.5 Flash model without agentic architecture to the version of AMIE used in the study, including post-training, Chain-of-Reasoning (CoR) in the Dialogue Agent and access to response from the Mx Agent. In addition, we also tested the newer Gemini 2.5 Flash model which was released after the study was conducted. Overall, the ablation analysis demonstrates clear improvements to AMIE's management reasoning performance across several auto-evaluation criteria stemming from the two-agent architecture and CoR, as well as how our post-training techniques improved AMIE's conversational history taking and diagnostic reasoning (with regressions on some other criteria). Details are provided in our response to R3 below and in Appendix Section A.17.

We thank the Editor and R3 for encouraging us to perform this ablation analysis which helped to elucidate how improvements and trade-offs stemming from different components of the system architecture.

2) We also ask that the model be benchmarked, when possible, to currently available LLMs and medical LMMs/LLMs.

Response: We have added comparisons of AMIE to currently available LLMs on the RxQA medication reasoning benchmark, including DeepSeek-V3, o-3, GPT-5, Gemini (1.5 Flash, 2.5

Flash, 2.5 Pro), as well as a variant of AMIE based on the newer Gemini 2.5 Pro. Results are provided in Appendix Table A.13.

Comparisons with Gemini models are provided for the full RxQA benchmark, including both OpenFDA and BNF portions. Due to licensing restrictions on the BNF portion, the comparison to non-Google models (DeepSeek-V3, o-3, GPT-5) was possible only on the OpenFDA portion. Results show that access to drug labels (in the open-book setting) consistently increases accuracy for all models compared to the closed-book setting (without access to drug labels). This finding validates the key purpose of this benchmark to test for the ability to retrieve and use information from authoritative drug formularies to inform medication reasoning. GPT-5 performed slightly worse than AMIE (2.5 Pro) on the closed-book setting (71.7% vs. 74.0%), but both were on par in the open-book setting (76.3% for both models).

We thank R2 for raising the valid concern of Gemini's dual involvement in benchmark creation and test-taking, and hope that this benchmarking exercise with other available models — in particular, parity between GPT-5 on and AMIE (2.5 Pro) in the open-book setting — helps to address this concern.

3) With the revised study, we ask that the OSCE training questions and transcripts of the dialogues between AMIE and the patient actors and the physicians and the patient actors be provided.

Response: In the revised manuscript, we provide metadata (specialty category, location, condition, relevant clinical guidelines, difficulty type) for all 120 scenarios, including the 20 scenarios used for validation and the 100 scenarios used in the OSCE study (Appendix Section A.16 Scenarios Overview).

In addition, we provide a full representation of two sample scenarios, including all OSCE scenario details, patient-actor-AMIE and patient-actor-PCP conversations, as well as post-questionnaire responses from both AMIE and PCP for all three visits in the scenario. Due to the extensive content (over 70 pages), these materials are provided as a separate PDF file including its own table of contents, overview of the data and commentary from qualitative review.

4) With the TTA on file at Nature, we will allow the reviewers access to AMIE in the next round of review.

Response: We have provisioned username/password combinations and a web link to provide access to an interactive version of AMIE as tested by patient actors in the OSCE study. The interactive access will allow reviewers to try out the system at different parts of the scenario sequence, including cases starting with the first visit, as well as cases starting the second or third visit. Reviewers will also see the scenario information presented to patient actors for each of these visits while interacting with the system. We have shared the web link and username/password combinations with the Editor in the cover letter.

5) We ask that the team consider release of as much training data, model code, and weights as possible. At the very least, we ask that detailed pseudocode be provided for review by the referees.

Response: We appreciate the encouragement to release as much data, code and weights as possible to enable reproducibility within the research community. We have taken the following measures to that effect:

1. **Benchmark Data:** We have released the RxQA benchmark dataset to the public at https://github.com/Google-Health/rxqa. This release includes the part of the benchmark derived from OpenFDA drug labels (300 questions total). Due to licensing restrictions, the BNF portion cannot be shared directly by Google. However, we have shared this portion with BNF itself, and the interested reader is encouraged to contact BNF to request access to the data.
2. **Pseudocode and prompts:** Our original submission contains detailed prompts and data classes used to build the agentic architecture, as well as representative model outputs for some, but not all prompt sections. We thank R2 for requesting "representative model outputs corresponding to the prompts in Appendices A.7 and A.8" which we have provided in the revision.
3. **Training data and model weights:** Parts of the training data are open-source, as described in the Data and Code Availability sections of the manuscript. Unfortunately, we are unable to share the other parts of the training data or model weights due to the commercial nature of these artifacts.

I would be happy to discuss these points and the reviewer reports by email or video call.

Should further experiments allow you to address these criticisms, we would be happy to consider a revised manuscript (unless something similar has been accepted at Nature or appeared elsewhere in the meantime). We hope to receive your revised paper within four to six months. If you cannot complete the required revisions within this time frame, please let us know when you would anticipate being able to submit a revised manuscript.

Any revised manuscript should conform to our format instructions and publication policies (see here). We also strongly suggest that your revised manuscript has tracked changes, which is increasingly requested by referees to aid in their re-review.

Response: Our revised manuscript has tracked changes to aid the referees in their re-review.

We would appreciate your careful attention to the following:

STATISTICS: When revising your manuscript, you should ensure that any statistical analysis used is sound and that it conforms to Nature's guidelines). A collection of articles explaining the basics of statistical analysis and advice on how to best present it can be found here.

REPRODUCIBILITY: All of the checklists provided with the current submission (Reporting summary, and Code and software checklist (if applicable)) should be updated to reflect the revisions made and submitted with the revised manuscript.

DATA AND CODE AVAILABILITY STATEMENTS: All original research manuscripts published in Nature Portfolio journals must include a Data availability statement. This statement must make the conditions of access to the “minimum dataset” that is necessary to interpret, verify and extend the research in the article, transparent to readers. This minimum dataset may be provided through deposition in public community/discipline-specific repositories, custom proprietary repositories for certain types of datasets, or general repositories like Figshare, Zenodo and Dryad. Providing large datasets in Supplementary Information is strongly discouraged; the preferred approach is to make data available in repositories. More information on Nature Portfolio’s reporting standards and preparing your Data availability statement can be found here.

For all studies using custom code or mathematical algorithms that are deemed central to the conclusions, a Code availability statement must be included, indicating whether and how the code or algorithm can be accessed, including any restrictions to access. The Code availability statement should be provided as a separate section after the Data availability statement but before the references. Code should be deposited in a DOI-minting repository such as Zenodo, Gigantum or Code Ocean and cited in the reference list. We encourage you to manage subsequent code versions and to use a license approved by the open source initiative. Additional details can be found here.

METHODS and ETHICS STATEMENT: After the main text figure legends there should be a section entitled "Methods", which provides the full, step-by-step instructions that would allow other researchers to replicate the results. The Methods section will not appear in print but will appear online in the full-text HTML and PDF versions. The Methods section should be written as concisely as possible but should contain all elements necessary to allow interpretation and reproduction of the results. If there are additional references in the Methods section, their numbering should continue from the last reference in the main text, and they should be listed following the Methods section. Specialized methods that require chemical structures, figures or tables, or methods requiring equations, cannot be accommodated in the Methods section of the main text file. If such information is part of the Methods, the entire Methods section must instead be included within a Supplementary Information text file.

For research involving human research participants, the Methods section must include an ethics statement. This statement should provide the name of the committee that approved the study; confirm that the research was performed in accordance with all relevant guidelines and regulations; and confirm that informed consent was obtained from all participants. If the study was granted an exemption from requiring ethics approval, details of the committee granting the exemption must be included.
For research involving human embryos or gametes, or human stem cells in contexts requiring ethical oversight, the Methods section must include an ethics statement. This statement should provide the name of the committee that approved the study and confirm that the research was

performed in accordance with all relevant guidelines and regulations. We encourage authors to follow the principles laid out in the 2021 ISSCR Guidelines for Stem Cell Research and Clinical Translation: https://www.isscr.org/guidelines. Where necessary, the ethics statement should also describe the conditions of donation of materials, such as human embryos or gametes, and confirm that informed consent was obtained from all donors of cells or tissues.

EXTENDED DATA: Extended Data do not appear in the print version of the paper but are included online within the full-text HTML and at the end of the online PDF. Extended Data are an integral part of the paper, and only data that directly contribute to the main message should be included. All Extended Data must be referred to in the main text, and their legends should be listed sequentially at the end of the main text, not in the Extended Data files. Extended Data should be assembled into a maximum of 10 A4 size, multi-panelled display items, submitted as individual files in .jpg, .tif or .eps format only. They should be of the same quality as print figures, but there are important differences in their formatting. More specific instructions are provided here. If you need to describe complex processes, we encourage you to include a schematic of the main finding as part of the Extended Data to aid readers unfamiliar with the immediate discipline.

SUPPLEMENTARY INFORMATION: Supplementary Information (SI) is online-only, peer-reviewed material that is essential background to the study (e.g., large data sets, more complex methods, and calculations), but which is too large or impractical, or of interest only to a few specialists, to justify inclusion in the print version of the paper (see here for further details). While SI should not typically contain data figures (any figures additional to those appearing in the main text should be formatted as Extended Data), we require that the raw, uncropped data for gels be presented as an SI figure (see below). Tables may be included in SI, but only if they are unsuitable for formatting as Extended Data (e.g., tables containing large data sets or raw data tables that are best suited to Excel files). If a manuscript has SI, each discrete item of the SI (e.g., videos, tables) must be referred to at an appropriate point in the main manuscript.

SOURCE DATA (GRAPHS): To increase transparency, we strongly encourage you to provide, in spreadsheet form, the data underlying the graphical representations used in figures. In the case of all experiments presenting data from animal models, this is a requirement and is not optional. This is in addition to our well-established data-deposition policy for specific types of experiments and large datasets. Online readers of the manuscript will be able to access the graphical source data directly from the figure legend. Spreadsheets must be submitted in .xls, .xlsx or .csv formats. One file per figure is permitted. If there is a multi-panelled figure, the source data for each panel should be clearly labeled in the file; alternatively the source data for a figure can be included in multiple, clearly labeled sheets within an Excel file. File sizes of up to 30 MB are permitted, but it is expected that the vast majority of graphical source data files will be considerably smaller than this. When submitting these files with your manuscript, you should select the "Source Data" file type and use the title field in the file description tab to indicate the figure(s) to which the source data pertain.

RAW DATA (GELS): You must provide the original source images for all data obtained by electrophoretic separation (e.g., EMSA, northern/Southern/western blots, etc). The raw images

should be assembled into a single .pdf or .tif file (multiple gels on a single page is encouraged). The file should be uploaded as Supplementary Figure 1. The full scanned images must be in uncropped form and contain labeled size/molecular weight markers and loading controls. There should be an accurate indication of how the gels were cropped for the final figure. The figure legends and raw data files should indicate whether controls (such as beta-actin) were run on the same gel as loading controls or on separate gels as sample processing controls (see here). While the data can be displayed in a relatively informal style, there must be a correspondence between each source data image and a specific main text or Extended Data figure. The main text or Extended Data figure legends should refer to the uncropped scans explicitly (e.g., "For gel source data, see Supplementary Figure 1."). For examples, see here or here.

THIRD PARTY RIGHTS: Please identify any content used in your article, whether in the main text, Extended Data or Supplementary Information, that comes from a third party. This could include figures, tables, images, videos or text boxes that are reproductions or adaptations of items that have previously been published elsewhere and/or are owned by a third party. It also encompasses pictures taken by professional photographers, maps and images downloaded from the internet. You must obtain the right to use each of these items and provide evidence that you have these rights. You will also need to give proper attribution to the copyright holders in your paper. We ask that you fill out a Third party rights table and upload this with your revised manuscript. You can find more information about third-party rights here.

In the meantime, we hope that you will find our referees' comments helpful. Please do not hesitate to contact me if there is anything you would like to discuss.

Yours sincerely

George Caputa, PhD
Senior Editor
Nature

Response to R1

This paper uses the articulate medical intelligence explorer (AMIE) in a randomized blinded virtual objective structured clinical examination study against 21 primary care physicians in 100 multi-visit case scenarios. The authors developed RxQA, a multiple choice question benchmark derived from national drug formularies. AMIE was non-inferior to PCPs on the case evaluation and performed better on RxQA. At this point, there have been many claims around the clinical abilities of models, but one of my biggest concerns is the lack of transparency around the datasets or the model and the complete inability to independently validate these claims.

Response: We thank the reviewer for their thoughtful comments, including considerations around transparency of the underlying datasets. We have taken several measures to address these concerns and hope that these measures may provide avenues to enable independent validation of our claims:

(1) We have now released the RxQA benchmark dataset to the public at https://github.com/Google-Health/rxqa. This release includes the part of the benchmark derived from OpenFDA drug labels (300 questions total). Due to licensing restrictions, the BNF portion cannot be shared directly by Google. However, we have shared this portion with BNF itself, and the interested reader is encouraged to contact BNF to request access to the data.

(2) In the revised manuscript, we provide metadata (specialty category, location, condition, relevant clinical guidelines, difficulty type) for all 120 scenarios, including the 20 scenarios used for validation and the 100 scenarios used in the OSCE study (Appendix Section A.16 Scenarios Overview).

(3) In addition, we provide a full representation of two sample scenarios, including all OSCE scenario details, patient-actor-AMIE and patient-actor-PCP conversations, as well as post-questionnaire responses from both AMIE and PCP for all three visits in the scenario. Due to the extensive content (over 70 pages), these materials are provided as a separate PDF file including its own table of contents, overview of the data and commentary from qualitative review.

(4) We provide a mechanism through which reviewers can interact with the version of AMIE used in this study. Access to the model will be facilitated by the Nature editor to preserve reviewer anonymity.

Comments
Why was the methodological decision made to use Gemini 1.5 Flash instead of PaLM-2?

Response: We chose to use Gemini 1.5 Flash for this study (rather than PaLM 2) primarily due to its long-context capabilities (2M token context window) necessary for inference-time reasoning across a large corpus of medical knowledge (clinical guidelines, drug formularies).

This was a key capability studied in this work and would not have been possible with the version of AMIE based on PaLM 2, which supports 8k tokens in the context window (250 times less than Gemini). In addition, Gemini 1.5 Flash was the most advanced base model available to us at the time the study was conducted allowing us to study state-of-the-art reasoning capabilities. We have added clarification for this rationale in Methods section 2.1.1 Post-training.

The scenario packs were prepared by physicians with 100 different scenarios across multiple clinical domains. However, the cases are not shared, so it's not clear how realistic the scenarios were, how "well packaged" the clinical information was.

Since there is no HIPAA here, this study should release both the physician-actor and AMIE-actor conversations so that reviewers can truly evaluate the model's performance. There are many ways to make the results look good without the model being actually clinically useful. The highest level of transparency is sharing the model, but the authors state that they will not do this. The second highest level of transparency is releasing the conversations and the "assessment" of the performance.

Response: We thank R1 for raising this concern. In the revised manuscript, we provide metadata (specialty category, location, condition, relevant clinical guidelines, difficulty type) for all 120 scenarios, including the 20 scenarios used for validation and the 100 scenarios used in the OSCE study (Appendix Section A.16 Scenarios Overview). In addition, we provide a full representation of two sample scenarios, including all OSCE scenario details, patient-actor-AMIE and patient-actor-PCP conversations, as well as post-questionnaire responses from both AMIE and PCP for all three visits. Due to the extensive content (over 70 pages), these materials are provided as a separate PDF file including its own table of contents, overview of the data and commentary from qualitative review. We hope that this will allow for an assessment of scenario distributions and realism as well as an assessment of model behavior.

Table A.1, this case of pheochromocytoma seems really contrived (which makes me wonder what the other cases are like). This is a rare disease that doesn't usually present in such an obvious way.

Response: Thank you for this comment. We agree that a robust assessment requires a wide range of clinical challenges. Our full scenario set is built to reflect this, spanning from common presentations of common diseases to more complex cases which may involve diagnostic uncertainty, atypical presentations, and multi-morbidity. We have added a new section in the appendix (A.16 Scenarios Overview) with metadata for all 120 scenarios (100 evaluation scenarios + 20 validation scenarios) to provide an overview of the case distribution. This pheochromocytoma case in particular was designed to test recognition and investigation of a particular rare but "cant-miss" diagnosis where ample clues were provided early on (headaches, diaphoresis and high blood pressure). We also provide a detailed account for two sample scenarios in a separate PDF file (over 70 pages), including all OSCE scenario details, patient-actor-AMIE and patient-actor-PCP conversations, as well as post-questionnaire responses from both AMIE and PCP for all three visits.

Figure A.5: The Mx agent mentions talking about symptoms of heart attack and stroke in a patient with major depressive episode. This does not seem to make sense. Additionally, “asking about side effects” is mentioned, but the specific side effects are not listed.

Response: We appreciate the referee’s careful review of this reasoning trace. The underlying scenario was designed such that the doctor should consider potential adverse effects of the treatment (Escitalopram). As R1 correctly points out, AMIE’s intermediate reasoning mentions that it is “important to educate the patient about the emergency symptoms of heart attack, stroke and worsening symptoms of depression.” While this consideration is not reflected in the final Mx plan produced by AMIE, we agree that heart attack and stroke are not relevant in this context, as these are not expected side effects of Escitalopram. We thank the reviewer for raising this imperfection and have added the following note to the Figure caption:

“Note that interrogation of reasoning traces allows inspection of latent reasoning errors that are not propagated into the final (appropriate) Mx Plan. In this case, a reasoning trace inspection reveals that AMIE's intermediate reasoning analysis suggests educating the patient about emergency symptoms of heart attack and stroke which are not clinically relevant in this context.”

Looking at BMJ’s best practice guidelines in pheochromocytoma, Table A.1 does not appear to include important information like considering whether or not the patient is in hypertensive crisis, considering genetic testing after diagnosis is made. Figure A.3 likewise doesn’t mention genetic testing.

Response: We thank the reviewer for raising this concern. While the plan does not mention hypertensive crisis explicitly, it recommends certain investigations potentially consistent with that consideration (“Review Gurmeet’s medication history [...]”, “Measure Gurmeet’s blood pressure in both arms, heart rate, respiratory rate, and temperature”). We agree that genetic testing is missing from both Table A.1 and Figure A.3, and have added the following to both captions:

- Added to Table A.1 caption: *“Note the imperfection that recommendations in this sample plan do not include genetic testing which could be considered appropriate in this setting. In addition, AMIE could have explicitly considered whether the patient is in a hypertensive crisis.”*

- Added to Figure A.3 caption: *“Note the imperfection that recommendations in this sample plan do not include genetic testing which could be considered appropriate in this setting.”*

There were time intervals between interactions with patient actors. While the models were allowed to “store” data, were the PCPs also allowed to take notes and review them? Physicians don’t have to ‘remember’ every detail of the patient visit, and their personal documentation is an important piece to have.

Response: We thank the reviewer for raising this important question. Yes, PCPs were allowed to take notes and review them. In addition, the interface displayed full conversation transcripts from all previous visits during each session so as to allow PCPs to review details from prior conversations about the same case as needed. We have clarified this in Methods.

Very little information on the raters' clinical experience and how they were trained to rate.

Response: We thank the reviewer for requesting more information regarding the clinical experience and training for specialist physician raters. In addition to the aggregate statistics provided in Section 3.1.3 (rater location, median + IQR of years post-residency experience), we have added Appendix A.15 "Metadata for Clinicians Involved in the OSCE Study" in the revised manuscript. A.15 tabulates the geographic location, specialty category and years of post-residency experience (at the time of study) for each of the 30 specialist physician raters and 21 PCP participants involved in the OSCE study.

Prior to involvement in the final evaluation study, specialist physician raters completed a set of pilot rating tasks (based on the 20 scenarios in the validation set) to familiarize themselves with the task setup and instructions. Detailed rating instructions were embedded in the rating interface itself along with tooltips contextualizing specific rating prompts. We have added this clarification in Methods 3.1.4.

Looking at figure A.25 – hard to say what the difference is between "slightly" and "somewhat" in rating responses.

Response: We agree that ordinal ratings using Likert scales are inherently subjective and a source of inter-rater variability. Given that different raters may apply different thresholds for "slightly" versus "somewhat", we made the methodological decision to have individual raters assess both AMIE and PCP for any given scenario (in a blinded and randomized manner), rather than AMIE and PCP output to entirely different raters. Per our elaboration below, we were able to increase the rating step from single ratings to triplicate ratings per case, and followed the same procedure here: each of the 3 raters assigned to a given case assessed both AMIE and PCP for the given scenario (in a blinded and randomized manner). The specific choice of "somewhat" as a distinction from "slightly" is common for Likert scales. Another common alternative is "moderately" and both have been used to rate subjective constructs like confidence / familiarity / awareness (see examples here).

Was there any information on inter-rater agreement? Or was only a single rater used?

Response: We appreciate the reviewer's request for information on inter-rater agreement. The original submission included results from a single rater per rating task. Since then, we completed additional data collection and managed to collect triplicate ratings for all cases. The revised manuscript includes results based on these triplicate ratings (aggregated using the median) as well as inter-rater reliability statistics in Appendix A.14 "Inter-rater Reliability Analysis

for Specialist Physician Ratings”, suggesting ‘substantial’ or higher (κ > 0.6) agreement across criteria.

The Management Reasoning Empirical Key Features (MXEKF) rubric is not externally validated, which is concerning and difficult to assess.

Response: While inspired by prior work (Cook et al. “Management reasoning: empirical determination of key features and a conceptual model” Academic Medicine 2023; https://pubmed.ncbi.nlm.nih.gov/35830267/), we agree that MXEKF lacks external validation. We describe MXEKF as a “new pilot evaluation rubric” in the manuscript, and hope that the additional inter-rater reliability statistics provided in the revision can help to contextualize rubric’s value and limitations. We encourage further validation in future work.

Will RxQA be released?

Response: We have now released the RxQA benchmark dataset to the public at https://github.com/Google-Health/rxqa. This release includes the part of the benchmark derived from OpenFDA drug labels (300 questions total). Due to licensing restrictions, the BNF portion cannot be shared directly by Google. However, we have shared this portion with BNF itself, and the interested reader is encouraged to contact BNF to request access to the data.

There is no information on the experience of the PCPs used in this study.

Response: We describe PCP experience in Section 3.1.3 as “9 years of post-residency experience (interquartile range of 5 to 12 years)”. To shed more light on rater experience, in the revised manuscript, we have added Appendix A.15 “Metadata for Clinicians Involved in the OSCE Study” in the revised manuscript. A.15 tabulates the geographic location, specialty category and years of post-residency experience (at the time of study) for each of the 30 specialist physician raters and 21 PCP participants involved in the OSCE study.

Was there any overlap between the 20 validation scenarios described in 3.1.1 and the ultimate cases used to test PCPs and AMIE?

Response: There was no overlap between validation scenarios and the scenarios used for evaluation in the OSCE study. In the revised manuscript, we provide metadata (specialty category, location, condition, relevant clinical guidelines, difficulty type) for all 120 scenarios, including the 20 scenarios used for validation and the 100 scenarios used in the OSCE study (Appendix Section A.16 Scenarios Overview), and have clarified in the table that validation scenarios were used for used for auto-evaluation and piloting only (not for the OSCE study).

Referee #1 (Remarks on code availability):

cannot assess code as nothing is shared.

Response to R2

A. Summary of the key results

This work extends the capabilities of the previously proposed Articulate Medical Intelligence Explorer (AMIE), a large language model (LLM) based AI system originally optimized for diagnostic conversations [1], to include disease management functionalities.

The authors evaluate and compare AMIE's disease management performance with primary care physicians (PCPs) in two aspects:

(1) Multi-visit OSCE study: It utilizes a text-chat interface to evaluate AMIE's ability to diagnose and manage patients over a longitudinal period, which helps assess its capability in adjusting management plans as patient information evolves. Results show that AMIE's disease management plans are non-inferior to those of PCPs, are better aligned with clinical guidelines, and are preferred by both patients and physicians.

(2) Medication knowledge and reasoning: Through a multiple-choice question benchmark called RxQA, the authors evaluate and compare AMIE's and PCPs' ability to prescribe medications. Results show that AMIE significantly outperforms PCPs on higher-difficulty questions, and achieves comparable performance on lower-difficulty questions.

Overall, the results indicate that AMIE's disease management abilities are comparable or superior to those of PCPs within simulated clinical scenarios.

Response: We thank R2 for their thoughtful review of our manuscript and address the reviewer's comments inline below.

B. Originality and Significance

Previous research on task-oriented conversational AI in healthcare has predominantly focused on advancing diagnostic abilities. In contrast, this study extends the application of LLM-based systems to disease management, which requires the ability to track disease progression, formulate treatment plans and prescriptions, and monitor therapeutic responses over multiple patient visits.

In my opinion, the significance of this work lies in the positive results on OSCE-based evaluations. Objective Structured Clinical Examinations (OSCEs) are widely recognized as a standard for evaluating clinical competencies among medical students and for medical licensing exams around the world. Therefore, the positive OSCE results in this study represent a significant step towards integrating AI systems into clinical practice for disease management. Although the current evaluations were limited to a text-based conversational

interface—indicating the necessity for further real-world studies—these results provide preliminary evidence for subsequent clinical validation.

Response: We appreciate R2's assessment that the positive results from our OSCE-based evaluations are of particular significance to this work. In particular, we expanded on OSCE study methods from Tu et al. 2025 by introducing a *multi-visit longitudinal* variant of this OSCE approach, incorporating the use of clinical guidelines, to evaluate the performance of our technical contribution. We thank the reviewer for recognizing the particular significance of the methodological contribution in this work.

C. Data and Methodology

(1) Validity of approach: The design of using two agents—one "fast-thinking" dialogue agent to ensure quick conversational responses and one "slow-thinking" Mx agent for spending more inference-time compute on management reasoning—is well-motivated. **However, I have the following question (Q1) for the authors:** The conversation between AMIE and the patient actor is synchronous, while the agent state fields are updated asynchronously. Wouldn't this design introduce information mismatch between the agent state and the current dialogue turn? Ideally, to ensure optimal dialogue quality, the dialogue agent should have access to the most recent patient information up to the current dialogue turn.

Response: We thank R2 for raising this important question. In the revised manuscript, we have further emphasized that the dialogue agent indeed does have the full conversation transcript including all patient information available at *each* turn of the conversation (added "*full* dialogue history up to that point" in Section 2.1.2 Chain-of-Reasoning). In addition, the dialogue agent updates its state object in a synchronous manner. The only part being updated in an asynchronously every few turns is the management (Mx) plan produced by the Mx Agent. This plan helps steer the dialogue agent's line of inquiry throughout the conversation.

We acknowledge that due to the interleaved and asynchronous nature of tool calling in this context, the latest generated Mx plan may lag behind by a few conversation turns. These asynchronous updates are a necessary tradeoff to enable acceptable latency.

We observed that, in the majority of turns, the Mx plan remains stable. In addition, the dialogue agent is only conditioned on the output of the Mx Agent; therefore, it retains the flexibility to adjust its response based on the latest conversation state. Finally, note that at the end of the conversation, a final Mx plan is computed based on the full information shared throughout the conversation. This allows us to surface AMIE's final clinical assessments such as differential diagnosis, investigation, and treatment recommendations, which are used to inform post-questionnaire responses.

(2) Validity of data:

a. Training data are mainly used to fine-tune the base model (i.e., Gemini 1.5 Flash) of the dialogue agent. The authors use simulated dialogues, real-world medical dialogues, medical question-answering (QA), and EHR summarization datasets for supervised fine-tuning (SFT). Following SFT, they use preference pairs of dialogue responses and case management recommendations to perform reinforcement learning with human / AI feedback (RLHF / RLAIF). Among the training data, only some datasets for medical QA and EHR summarization (MedQA, MultiMedQA, MIMIC-III) are open-source. Therefore, the quality of medical dialogues and preference pairs cannot be verified directly.

Response: This is an accurate summary of the datasets involved in model development. As R2 correctly points out, parts of the training data are open-source, as described in the Data and Code Availability sections of the manuscript. Unfortunately, we are unable to share the other parts of the training data or model weights due to the commercial nature of these artifacts. We thank R2 for requesting "representative model outputs corresponding to the prompts in Appendices A.7 and A.8" (in other comment below) which we have provided in the revision.

b. Testing data for multi-visit OSCE study consist of 100 scenarios, which were prepared by clinical providers in Canada and India. The scenarios encompass clinical cases from five different medical specialties to ensure diversity. Since the scenarios are not openly available, I cannot verify the validity of these data. As for testing data in RxQA medication benchmark, 600 questions are generated by Gemini 1.5 Flash based on medication labels from OpenFDA and British National Formulary (BNF), and the questions are further refined by board-certified pharmacists. **Since the base model of AMIE is also Gemini 1.5 Flash, this naturally raises the question (Q2) of whether Gemini 1.5 Flash has the bias to generate questions that can be answered by itself.** This bias would potentially make the findings in Figure 7 less convincing. Could the authors elaborate on the choice to use the same LLM for generating the questions? Did the authors experiment with other LLMs for question generation?

Response: We address R2's two comments regarding OSCE scenario data and the RxQA benchmark separately below.

OSCE scenario data:

In the revised manuscript, we provide metadata (specialty category, location, condition, relevant clinical guidelines, difficulty type) for all 120 scenarios, including the 20 scenarios used for validation and the 100 scenarios used in the OSCE study (Appendix Section A.16 Scenarios Overview).

In addition, we have added a full representation of two sample scenarios, including all OSCE scenario details, patient-actor-AMIE and patient-actor-PCP conversations, as well as post-questionnaire responses from both AMIE and PCP for all three visits in the scenario. Due to the extensive content (over 70 pages), these materials are provided as a separate PDF file

including its own table of contents, overview of the data and commentary from qualitative review. We hope that this added information may provide avenues to enable independent validation of our claims.

RxQA benchmark:

We have now released the RxQA benchmark dataset to the public at https://github.com/Google-Health/rxqa. This release includes the part of the benchmark derived from OpenFDA drug labels (300 questions). Due to licensing restrictions, the BNF portion cannot be shared directly by Google. However, we have shared this portion with BNF itself, and the interested reader is encouraged to contact BNF to request access to the data.

In the revised manuscript, we have also added Appendix Table A.13 with new comparisons of AMIE to currently available LLMs on RxQA, including DeepSeek-V3, o-3, GPT-5, Gemini (1.5 Flash, 2.5 Flash, 2.5 Pro), as well as a variant of AMIE based on the newer Gemini 2.5 Pro.

Comparisons with Gemini models are provided for the full RxQA benchmark, including both OpenFDA and BNF portions. Due to licensing restrictions on the BNF portion, the comparison to non-Google models (DeepSeek-V3, o-3, GPT-5) was possible only on the OpenFDA portion.

Results show that access to drug labels (in the open-book setting) consistently increases accuracy for all models compared to the closed-book setting (without access to drug labels). This finding validates the key purpose of this benchmark to test for the ability to retrieve and use information from authoritative drug formularies to inform medication reasoning. GPT-5 performed slightly worse than AMIE (2.5 Pro) on the closed-book setting (71.7% vs. 74.0%), but both are on par in the open-book setting (76.3% for both models).

We thank R2 for raising the valid question of whether Gemini 1.5 Flash has the bias to generate questions that can be answered by itself.

For question generation, we were unable to leverage competitor models due to legal considerations. We did explore using Gemini 1.5 Pro, but qualitatively found little difference in the quality and style of the questions. Thus, we selected Gemini 1.5 Flash, a smaller and less computationally expensive model. We agree with the reviewer that following a similar process with other families of models (GPT, Claude, etc.) could improve question diversity, and we encourage others to follow the question generation process in the appendix with other base model choices.

While the point about potential bias in RxQA is valid, a few technical details help mitigate this risk, and we hope that our benchmarking results with competitor models help address the concern. Firstly, in our filtering process, we exclude generations that Gemini 1.5 Flash was able to answer zero-shot without context. Secondly, the pharmacists not only made revisions to the questions and answer choices, but also selected the correct answer without knowledge of what the model thought the correct answer was. Thirdly, for the longer style of questions, which

makes up two-thirds of the RxQA dataset, questions were generated through a complex multi-step process. Finally, our benchmarking results with competitor models — in particular, superior performance of DeepSeek-V3, o-3, GPT-5 compared to base Gemini 1.5 Flash, as well as parity between GPT-5 on and AMIE (2.5 Pro) in the open-book setting — demonstrate that the benchmark does not necessarily favor Gemini models.

(3) Quality of presentation:

Overall, the manuscript is well-organized and clearly written. The detailed presentation of the Mx agent, including specific prompts and illustrative model outputs in Appendices A.1 through A.4, is particularly commendable. However, although the authors include prompts (Appendices A.7 and A.8) used for generating the dialogue agent's training data, actual examples of corresponding model outputs are absent. Similarly, examples of dialogues between AMIE and patient actors are also missing. Including representative model outputs corresponding to the prompts in Appendices A.7 and A.8, as well as sample dialogues between AMIE and patient actors, would facilitate a more comprehensive understanding of the work.

Response: We thank R2 for encouraging sharing of sample dialogues between patient actors and AMIE or PCPs, as well as representative model outputs for Appendices A.7 and A.8.

Along with this revision, we provide a detailed view of two sample scenarios, including all OSCE scenario details, patient-actor-AMIE and patient-actor-PCP conversations, as well as post-questionnaire responses from both AMIE and PCP for all three visits in the scenario. Due to the extensive content (over 70 pages), these materials are provided as a separate PDF file including its own table of contents, overview of the data and commentary from qualitative review.

To give a sense of the case distribution overall, we also provide metadata (specialty category, location, condition, relevant clinical guidelines, difficulty type) for all 120 scenarios, including the 20 scenarios used for validation and the 100 scenarios used in the OSCE study (Appendix Section A.16 Scenarios Overview).

Finally, we have added representative model outputs corresponding to the prompts in Appendices A.7 and A.8 to facilitate a more comprehensive understanding of the work, including outputs for synthetic rewrites of single-visit dialogues (Figure A.15), example outputs for multi-visit dialogue generation (Figure A.19), and example outputs from the auto-evaluation process used to filter dialogues during training (Figure A.22).

D. Appropriate Use of Statistics

The McNemar test is used for the evaluation axes in multi-visit OSCE and the accuracy on the RxQA benchmark, with p-values adjusted using false discovery rate (FDR) correction. As the data are paired (AMIE vs. PCPs for the same questions or evaluation items) and results are

binarized, the statistical tests are appropriate. Error bars are defined in the corresponding figures.

Response: We appreciate R2's careful review of our statistical approach.

E. Conclusions

Overall, the conclusions presented are valid and supported by the data. However, a previously noted concern remains regarding the RxQA medication benchmark, where the questions were generated by Gemini 1.5 Flash—the same base model underlying AMIE. This choice introduces a potential bias that may affect the robustness of findings in Figure 7. Clarification on the rationale behind using the same model and any experimentation with alternative models would be great.

Response: We agree with this notion and hope that our response above serves to address these considerations.

F. Suggested Improvements

Following the previously raised concerns, I suggest the authors clarify the following aspects in the revised manuscript:

1. Agent state synchronization: The dialogue between AMIE and patient actors occurs synchronously, yet the agent's state fields are updated asynchronously. Could this design potentially lead to information mismatches between the agent state and the latest dialogue turn? If so, how would such mismatches influence conversational quality?

Response: We refer the reviewer to our response above.

2. RxQA benchmark generation: The RxQA medication benchmark questions were generated using Gemini 1.5 Flash, which is also the base model of AMIE. Explain the motivation behind using the same model for question generation and whether alternative LLMs were considered or tested.

Response: Please refer to our response above addressing this valid concern.

3. Additional illustrative examples: Include representative model outputs for the training data prompts (Appendices A.7 and A.8), as well as example dialogues between AMIE and patient actors.

Response: We have added representative model outputs corresponding to the prompts in Appendices A.7 and A.8 per our response to the corresponding comment above.

G. References

The manuscript appropriately references relevant prior literature and situates its contributions within existing research on medical conversational AI.

Response: We thank R2 for reviewing related work and appreciate their assessment.

H. Clarity and Context

The abstract, introduction, and conclusions are appropriate and contextualize the study's contributions and significance.

Response: We are delighted about R2's assessment with respect to clarity and context of our abstract, introduction and conclusion sections.

* References mentioned in this review:
[1] Tu, T., Palepu, A., Schaekermann, M., Saab, K., Freyberg, J., Tanno, R., ... & Natarajan, V. (2024). Towards conversational diagnostic AI. arXiv preprint arXiv:2401.05654.

Referee #2 (Remarks on code availability):

The authors do not provide the code in the submission.

Response to R3

Review of Lievin et al. "Towards Conversational AI for Disease Management"

Lievin et al. build upon the same team's previous work (Tu et al. Nature 2025) that introduced the Articulate Medical Intelligence Explorer (AMIE), a conversational AI system designed to engage in dialogue with patients. While prior evaluations of AMIE have focused on diagnostic reasoning, the current analysis focuses on the disease management reasoning capabilities of AMIE. The topic of management reasoning of AMIE and of management reasoning by LLMs more broadly is timely and interesting but the present study, as reported, makes it hard to understand the critical question of where the reported management reasoning capabilities arise (from the new base LLM, post-training, or other modifications?) and thus reduces some of my enthusiasm. Major comments:

1. This version of AMIE is not the same as the version of AMIE introduced by Tu et al. in Nature earlier this year. For example, in Section 2.1.1 on post-training, the authors describe some of the changes to the Dialogue Agent, which is most comparable with the AMIE system in Tu et al. Nature 2025. First, the authors use Gemini 1.5 Flash instead of PaLM-2. Second, the authors "developed a new set of simulated dialogues...used to fine-tune the base model..." Third, the authors employ SFT/RLHF/RLAIF, however details are sparse. Most importantly, without rigorous ablation studies that compare AMIE to the new base LLM(s) and tease out the effects of individual components of the system (e.g. dual-agent Mx Agent and Dialogue Agent setup, new dialogues used in fine-tuning, SFT/RLHF design choices..etc.), it is very difficult to understand whether the management reasoning capabilities reported in the manuscript arise from the advancing capabilities of the base LLM(s) or the medical scaffolding in the AMIE system that is layered on top. This gets to the heart of a major debate (how much specialization is needed to use LLMs in medical contexts) that will be of substantial interest to readers of this paper and to the broader AI community, and is necessary to properly understand the major contributions in this manuscript.

Response: We thank R3 for your comprehensive feedback. We agree that further ablation results are necessary to enhance the scientific rigor of our paper and have designed additional experiments to help address these concerns. In the revision, we include results from an entirely AI-simulated re-creation of our OSCE study, conducted to ablate the effects of different backend models and different agent configurations (Appendix Section A.17).

In particular, compared the 12 configurations of a doctor agent resulting from different combinations of

Backend models:

1. **Base 1.5 Flash:** Gemini 1.5 Flash without modifications
2. **AMIE 1.5 Flash:** Gemini 1.5 Flash post-trained using the procedure described in Section 2.1.1
3. **Base 2.5 Flash:** Gemini 2.5 Flash without modifications

Agent configurations:

1. **Dialogue Agent (Prompt):** A basic variant of the Dialogue Agent, calling the backend model directly using a basic prompt, *without* Chain-of-Reasoning (CoR) and *without* Mx Agent.
2. **Dialogue Agent (CoR):** Same as (1), but using the Chain-of-Reasoning (CoR) as described in Section 2.1.2.
3. **Dialogue Agent (Prompt) + Mx Agent:** Same as (1), but with the Mx Agent as described in Section 2.2.
4. **Dialogue Agent (CoR) + Mx Agent:** Same as (2), but with the Mx Agent as described in Section 2.2.

The configuration used in our OSCE study corresponds to backend model 2 (AMIE 1.5 Flash) paired with agent configuration 4 (Dialogue Agent (CoR) + Mx Agent). We provide auto-evaluation scores for various criteria across PACES (Table A.9), Diagnosis & Management (Table A.10) and MXEKF (Table A.11) rubrics. In summary, the results from our ablation analysis support that combining Dialogue Agent with the Mx Agent into a two-agent architecture and enhancing the Dialogue Agent with a Chain-of-Reasoning (CoR) improves scores across most criteria and backend models.

For the backend model, the ablation analysis suggests a trade-off. On the one hand, the post-training for AMIE 1.5 Flash helped to improve the system's ability to elicit and explain clinical information in a conversational manner (reflected in more favorable PACES ratings in auto-evaluation) and allowed it to perform more accurate diagnostic reasoning based on that information (reflected in higher DDx accuracy in auto-evaluation). On the other hand, we observed regressions in other areas for the post-trained model. The backend model tested in the OSCE study is supported by these results, as high-quality conversational interaction with patient actors, especially across multiple sequential visits, was crucial for the study. A comparison to PaLM-2 base models was not possible; PaLM-2 only supported up to 8K token context, insufficient for some longer multi-visit interactions, and the technical infrastructure had evolved such that older models were no longer supported.

We would like to thank R3 for the excellent suggestion to shed light on various ablations of backend models and agent configurations which we believe has significantly strengthened this manuscript.

2. Relatedly, the present manuscript would be strengthened by comparing AMIE to any or several of a readily available number of leading large language models (e.g. OpenAI o-series, Meta's Llama, newer versions of Gemini) or other medical-specialized LLMs, several of which have demonstrated strong performance on a broad array of other benchmarks including those related to management reasoning.

Response: We thank R3 for suggesting comparisons to other leading LLMs. In the revised manuscript, we have added comparisons of AMIE to currently available LLMs on the RxQA

medication reasoning benchmark, including DeepSeek-V3, o-3, GPT-5, Gemini (1.5 Flash, 2.5 Flash, 2.5 Pro), as well as a variant of AMIE based on the newer Gemini 2.5 Pro. Results are provided in Appendix Table A.13.

Comparisons with Gemini models are provided for the full RxQA benchmark, including both OpenFDA and BNF portions. Due to licensing restrictions on the BNF portion, the comparison to non-Google models (DeepSeek-V3, o-3, GPT-5) was possible only on the OpenFDA portion.

These new benchmarking results demonstrate that access to drug labels (in the open-book setting) consistently increases accuracy across all models compared to the closed-book setting (without access to drug labels). While GPT-5 performed slightly worse than AMIE (2.5 Pro) on the closed-book setting (71.7% vs. 74.0%), both were on par in the open-book setting (76.3% for both models). A similar trend was observed for o-3 with a closed-book accuracy of 70.1% and open-book accuracy of 76.5%. Note that, in the open-book setting, our testing infrastructure put competitor models at a slight advantage by providing the relevant drug labels as static context to them, whereas AMIE models were tasked with the more difficult task of first retrieving the relevant drug labels from the corpus before answering the question.

Separately, we provide a detailed ablation analysis on a simulated multi-visit OSCE evaluation with various combinations of backend model and agent architectures in Appendix Section A.17, including a newer version of Gemini (2.5 Flash). A comparison of AMIE with competitor models in this end-to-end OSCE simulation was not possible due to licensing restrictions on the scenario materials. We hope that the comparison on RxQA serves to address the reviewer's comment.

3. Strengths of the study include rich, multi-dimensional evaluations of management reasoning as well as a randomized study using Objective Structure Clinical Examination (OSCE) scenarios. At the same time, there are a very large number of effects and p-values reported across visits and evaluation axes from these experiments, and I am both concerned about multiplicity and unsure how some of the significance calculations were performed (e.g. for "providing appropriate follow-up recommendations (100% vs. 98%, p<0.001)"). Could the authors clarify the statistical reporting generally and how the FDR-adjustment was calculated specifically [e.g. across all tests performed in the OSCE study or some subset for each sub-analysis..etc.]?

Response: We appreciate R3's question about statistical reporting and provide elaboration below.

Note that results in the revised manuscript are based on **triplicate** ratings stemming from 3 independent specialist physician ratings for each case. The original submission was based on single-specialist ratings per case. Expanding from single-specialist to triplicate-specialist ratings was done to address comments regarding inter-rater reliability and to improve overall robustness of results. Triplicate ratings aggregated using the median; beyond that, all other statistical reporting is identical to the original submission.

Importantly, given R3's feedback, we were able to identify and fix one inconsistency present in our original submission that affected a small number of tests (5 of 45 McNemar tests), specifically those where contingency tables for McNemar tests had one row with all-zero counts.

Details for statistical testing on management plan quality (Section 4.1 and Figure 5):

Measures:

- 15 evaluation axes (9 Yes/No scales + 6 Likert scales) were mapped to favorable vs. unfavorable as follows:
  - 5x Overall:
    - Overall Appropriate (5-point, top-2 map to 'favorable')
    - Free of Significant Error (Yes/No)
    - Follow-up Appropriate (Yes/No)
    - Selected Applicable Guidelines (5-point, top-2 map to 'favorable')
    - No Guideline Conflict or Conflict Justified (Yes/No)
  - 5x Investigations:
    - Appropriate Recommended (Yes/No)
    - Inappropriate Avoided (Yes/No)
    - Sufficiently Precise (Yes/No)
    - Aligned with Guidelines (5-point, top-2 map to 'favorable')
    - References Guidelines (4-point, top-2 map to 'favorable')
  - 5x Treatments:
    - Appropriate Recommended (Yes/No)
    - Inappropriate Avoided (Yes/No)
    - Sufficiently Precise (Yes/No)
    - Aligned with Guidelines (5-point, top-2 map to 'favorable')
    - References Guidelines (4-point, top-2 map to 'favorable')
- For each of 15 evaluation axes and each of 3 visits, this resulted in 100 paired binary ratings (favorable vs. unfavorable), corresponding to one rating for each study arm (AMIE vs. PCP) across 100 OSCE scenarios.

Exclusions:

- Cases where *either* AMIE or PCP had an N/A rating for a given evaluation axis and visit number were excluded, resulting in slightly fewer than 100 paired binary ratings for some axes / visits, see table below.

Test:

- The McNemar test was performed 45 times (15 evaluation axes x 3 visits), each with N=100 (or slightly fewer) corresponding to paired binary ratings for 100 scenarios.

- Contingency tables were generated using the following case counts:

# Both favorable	# AMIE favorable, PCP not
# PCP favorable, AMIE not	# Both NOT favorable

  - We used the following method from the Python statsmodels package:
    - statsmodels.stats.contingency_tables.mcnemar(contingency_table, exact=True) # using default value of correction=True
    - https://www.statsmodels.org/dev/generated/statsmodels.stats.contingency_tables.mcnemar.html

FDR correction:

- The 45 McNemar test resulted in 45 test statistics and 45 p-values.
- The 45 p-values were consequently adjusted using FDR correction using the following method from the Python statsmodels package:
  - statsmodels.stats.multitest.fdrcorrection(p_values) # using default values of alpha=0.05, method='indep', is_sorted=False
  - https://www.statsmodels.org/dev/generated/statsmodels.stats.multitest.fdrcorrection.html

Reporting:

- P-values reported in the paper correspond to those after FDR correction, see column "P-value (final after FDR)" in the table below.
- Percentage values were used to summarize the portion of favorable cases per study arm and visit.

Inconsistency identified and resolved:

- We thank R3 for inquiring about the details of statistical reporting. Providing the details allowed us to identify one inconsistency present in our original submission that affected 5 of 45 tests where the bottom row of the contingency table for the McNemar test was all zero counts.
- The underlying reason for this inconsistency was the behavior of pandas.crosstab (https://pandas.pydata.org/docs/reference/api/pandas.crosstab.html) which behaved in ways previously unaccounted for in those cases where one row in the contingency table was all-zero counts.
- Of 45 tests (15 evaluation axes x 3 visits), the following 5 tests were affected (the relevant rows are highlighted with yellow background in the table below):
  - **Overall: Follow-up Appropriate [Visit 2]** → p-value prior to bug fix was corrected p=1.26E-29 and after the bug fix was p=0.25; the FDR-corrected p-value is NOT significant (p = 0.3041)
  - **Investigations: References Guidelines [Visit 1]** → p-value prior to bug fix was corrected p=1.01E-28 and after the bug fix was p=0.03125; the FDR-corrected p-value is NOT significant (p = 0.0521)
  - **Investigations: References Guidelines [Visit 1, 2, 3]** → for these axes, FDR-corrected p-values after the bug fix were still significant ($p < 0.05$ for visit 1; $p < 0.01$ for visit 2; $p < 0.001$ for visit 3)

- The specific example R3 inquired about – “providing appropriate follow-up recommendations (100% vs. 98%, p<0.001)” – was affected by this inconsistency present in the original submission and has been resolved in the revised manuscript.

Detailed counts and test results, including rows affected by inconsistency:

Visit	N	# Both favorable	# Both NOT favorable	# AMIE favorable, PCP not	# PCP favorable, AMIE not	% Favorable AMIE	% Favorable PCP	P-value before FDR correction	Incorrect P-value (prior to bug fix) before FDR correction	P-value (final after FDR)	Signifi-cance
**Overall: Overall Appropriate**											
1	100	70	3	25	2	95%	72%	5.65E-06	Not affected	4.24E-05	***
2	100	77	1	19	3	96%	80%	0.0008554458618	Not affected	0.002405941486	**
3	100	80	1	18	1	98%	81%	7.63E-05	Not affected	0.0003121115945	***
**Overall: Free of Significant Error**											
1	100	87	1	9	3	96%	90%	0.1459960938	Not affected	0.1990855824	n.s.
2	100	89	2	7	2	96%	91%	0.1796875	Not affected	0.2310267857	n.s.
3	100	88	1	10	1	98%	89%	0.01171875	Not affected	0.02109375	*
**Overall: Follow-up Appropriate**											
1	100	100	0	0	0	100%	100%	1	Not affected	1	n.s.
2	100	97	0	3	0	100%	97%	0.25	1.26E-29	0.3040540541	n.s.
3	100	94	0	5	1	99%	95%	0.21875	Not affected	0.2734375	n.s.
**Overall: Selected Applicable Guidelines**											
1	100	80	2	11	7	91%	87%	0.480682373	Not affected	0.5407676697	n.s.
2	100	87	1	5	7	92%	94%	0.7744140625	Not affected	0.8409970306	n.s.
3	100	85	2	6	7	91%	92%	1	Not affected	1	n.s.
**Overall: No Guideline Conflict or Conflict Justified**											
1	100	75	6	13	6	88%	81%	0.1670684814	Not affected	0.221120049	n.s.
2	100	75	7	11	7	86%	82%	0.480682373	Not affected	0.5407676697	n.s.
3	100	76	8	9	7	85%	83%	0.8036193848	Not affected	0.8409970306	n.s.
**Investigations: Appropriate Recommended**											
1	100	65	10	18	7	83%	72%	0.04328525066	Not affected	0.06716676827	n.s.
2	100	73	4	21	2	94%	75%	6.60E-05	Not affected	0.0002971887589	***
3	100	76	5	16	3	92%	79%	0.004425048828	Not affected	0.009482247489	**
**Investigations: Inappropriate Avoided**											
1	100	76	3	15	6	91%	82%	0.07835388184	Not affected	0.1175308228	n.s.
2	100	80	3	11	6	91%	86%	0.3323059082	Not affected	0.3935201545	n.s.
3	100	81	3	7	9	88%	90%	0.8036193848	Not affected	0.8409970306	n.s.
**Investigations: Sufficiently Precise**											
1	100	86	1	12	1	98%	87%	0.00341796875	Not affected	0.008095189145	**
2	98	80	1	17	0	99%	82%	1.53E-05	Not affected	8.58E-05	***

3	99	81	2	16	0	98%	82%	3.05E-05	Not affected	0.0001525878906	***
**Investigations: Aligned with Guidelines**											
1	100	74	6	17	3	91%	77%	0.002576828003	Not affected	0.006821015302	**
2	100	74	4	20	2	94%	76%	0.0001211166382	Not affected	0.0004225510817	***
3	100	80	5	13	2	93%	82%	0.007385253906	Not affected	0.01444940982	*
**Investigations: References Guidelines**											
1	100	94	0	6	0	100%	94%	0.03125	1.01E-28	0.05208333333	n.s.
2	100	87	0	12	1	99%	88%	0.00341796875	Not affected	0.008095189145	**
3	100	88	0	10	2	98%	90%	0.03857421875	Not affected	0.06199428013	n.s.
**Treatments: Appropriate Recommended**											
1	100	60	7	27	6	87%	66%	0.0003240634687	Not affected	0.0009721904062	***
2	100	60	8	30	2	90%	62%	2.46E-07	Not affected	3.70E-06	***
3	100	69	4	25	2	94%	71%	5.65E-06	Not affected	4.24E-05	***
**Treatments: Inappropriate Avoided**											
1	100	87	0	10	3	97%	90%	0.09228515625	Not affected	0.1339623236	n.s.
2	100	90	0	9	1	99%	91%	0.021484375	Not affected	0.03718449519	*
3	100	90	0	8	2	98%	92%	0.109375	Not affected	0.1538085938	n.s.
**Treatments: Sufficiently Precise**											
1	98	59	2	35	2	96%	62%	1.02E-08	Not affected	4.61E-07	***
2	99	62	3	32	2	95%	65%	6.94E-08	Not affected	1.56E-06	***
3	100	63	1	32	4	95%	67%	1.94E-06	Not affected	2.18E-05	***
**Treatments: Aligned with Guidelines**											
1	100	74	5	17	4	91%	78%	0.007197380066	Not affected	0.01444940982	*
2	100	72	4	21	3	93%	75%	0.0002771615982	Not affected	0.0008908765657	***
3	100	79	3	18	0	97%	79%	7.63E-06	Not affected	4.90E-05	***
**Treatments: References Guidelines**											
1	100	92	0	8	0	100%	92%	0.0078125	4.04E-28	0.0146484375	*
2	100	91	0	9	0	100%	91%	0.00390625	8.08E-28	0.0087890625	**
3	100	86	0	14	0	100%	86%	0.0001220703125	2.58E-26	0.0004225510817	***

4. The authors assess AMIE and PCPs based in part on clinical guidelines (e.g. items "Selected Applicable Guidelines" or "Aligned with Guidelines" in Figure 5) — the guidelines appears to be entirely from the UK but the 21 PCPs who participated in the study are based in India and Canada — is this a fair comparison for AMIE vs. PCPs in guideline use, or would PCPs do better if evaluated with respect to local guidelines? How might this affect generalizability?

Response: R3 raises an excellent point that warrants further elaboration. We have expanded the Discussion as follows to acknowledge this limitation:

"While NICE Guidance and BMJ Best Practice guidelines are primarily authored by UK-based institutions, they find application or adaptation also in other parts of the world. We acknowledge that PCPs participating in this study were based in Canada and India; thus, their familiarity with these guidelines based on everyday practice may have been limited. However, all participating PCPs were given access to the corpus of guidelines throughout the entire study period and were free to peruse the guidelines without time limitation after the virtual consultations. We encourage future work to explore the use of localized guidelines in specific geographies and contexts of care."

5. Section 2.2.2 on long-context reasoning is interesting and could be expanded in the main manuscript.

Response: We appreciate the reviewer's suggestion to expand Section 2.2.2 on long-context reason and have added the following paragraph:

"Long-context capabilities allow reasoning models to interact with large amounts of data via repeated in-context interactions. This reasoning process can be seen as emulating multi-step agentic workflows. In a single call, the model can look up key information in source documents, summarize and reference multiple passages, and search the documents again following preliminary conclusions. Long-context models are competitive with specialist systems on *Corpus-in-Context* tasks like open-domain question answering [24]. To make our system more robust, we utilized decoding constraints to enforce target behaviours citing sources and summarizing high-level management strategy based on the in-context documents (read more in section 2.2.4: "Structured Generation'')."

6. The authors describe some of the changes to AMIE to create the management focused AMIE system in the present manuscript, but overall engineering details are sparse, model code and weights are not released, and the system is inaccessible. It is thus challenging to know how reproducible and sensitive the core findings of the manuscript are.

Response: We thank the R3 for their considerations around transparency of the underlying datasets and access to the system. We have taken several measures to address these concerns and hope that these measures may provide avenues to enable independent validation of our claims:

(1) We have now released the RxQA benchmark dataset to the public at https://github.com/Google-Health/rxqa. This release includes the part of the benchmark derived from OpenFDA drug labels (300 questions total). Due to licensing restrictions, the BNF portion cannot be shared directly by Google. However, we have shared this portion with BNF itself, and the interested reader is encouraged to contact BNF to request access to the data.

(2) In the revised manuscript, we provide metadata (specialty category, location, condition, relevant clinical guidelines, difficulty type) for all 120 scenarios, including the 20 scenarios used

for validation and the 100 scenarios used in the OSCE study (Appendix Section A.16 Scenarios Overview).

(3) In addition, we provide a full representation of two sample scenarios, including all OSCE scenario details, patient-actor-AMIE and patient-actor-PCP conversations, as well as post-questionnaire responses from both AMIE and PCP for all three visits in the scenario. Due to the extensive content (over 70 pages), these materials are provided as a separate PDF file including its own table of contents, overview of the data and commentary from qualitative review.

(4) We provide a mechanism through which reviewers can interact with the version of AMIE used in this study. Access to the model will be facilitated by the Nature editor to preserve reviewer anonymity.

Referee #3 (Remarks on code availability):

Not made available by authors.

Response to the Referees

Response to R1
Response to R2
Response to R3

List of documents

This document provides inline responses to comments from the three external referees. In addition, we also provide the following documents as part of the revision:

1. **Cover letter**
2. **Revised manuscript** with tracked changes (one color per referee) including edits from the first round of revisions as well as additional edits in response to the latest round of referees' comments
3. **Revised appendix** with tracked changes (one color per referee) corresponding to the edits from the first round of revisions
4. **Detailed view of two sample scenarios** (107 pages, with separate table of contents; this document now also includes specialist physician gradings and patient actor gradings highlighted in blue font)

Response to R1

Thank you for the access to the model and the responses to the reviews.
Regarding reasoning traces of the model, you have responded, “Note that interrogation of reasoning traces allows inspection of latent reasoning errors that are not propagated into the final (appropriate) Mx Plan. In this case, a reasoning trace inspection reveals that AMIE's intermediate reasoning analysis suggests educating the patient about emergency symptoms of heart attack and stroke which are not clinically relevant in this context.”
I think it’s concerning that there are latent reasoning errors in the reasoning traces. How often does this happen? How often does this lead to a correct or incorrect answer?

Response: We highlight the possibility of latent errors in reasoning traces through qualitative examples like the one cited above to complement the detailed quantitative results on final management plan quality (Figure 5) and management reasoning per AI/PCP post-questionnaire responses (Figure 6, Table A.4, Section 3.1.4, see below) which are key contributions in the paper. A *quantitative* analysis of the system’s reasoning traces would require detailed expert clinician review of ~1200+ lengthy reasoning traces (~4 or more reasoning traces per conversation x 3 visits x 100 scenarios), which renders a comprehensive analysis prohibitive and therefore beyond the scope of this work.

We concur with R1 that this is indeed an interesting question and have expanded the Discussion as follows:

“This work assessed management reasoning on the basis of conversation content and post-questionnaire responses from both AMIE and PCPs. In addition, the AMIE system produced structured reasoning traces at multiple points throughout each conversation. While we provide qualitative examples of these reasoning traces and highlight the possibility of latent errors within such traces, a comprehensive quantitative analysis of such traces is beyond the scope of this work. We encourage future work to study reasoning traces of medical AI systems in a comprehensive and quantitative manner.”

Except from Section 3.1.4 Evaluation Measures: *“[...] To enable an assessment of MXEKF rubrics from a specialist physician perspective, the post-questionnaire also probed for aspects of management reasoning including whether and how AMIE/PCPs intentionally deviated from one of the selected clinical guidelines as well as comments about other acceptable management plans, competing priorities, the cost, effectiveness and side effects of the proposed plan, the prognosis with and without treatment of this patient, and recommendations for escalation and follow-up.”*

The specific questions from the AI/PCP post-questionnaire related to management reasoning (in addition to separate questions about clinical guidelines, differential diagnosis, investigations and specific treatments):

- Did you intentionally deviate from one of the clinical guidelines you selected above? Yes/No
- If yes please explain why (e.g. guideline does not cover this clinical scenario, patient preference, your preference).
- What are other acceptable management plans? Why did you choose yours?
- Please describe any competing priorities that affected your decisions.
- Describe the cost, effectiveness and side effects of your management plan.
- Describe the prognosis with and without treatment of this patient.
- Does this case require escalation of this text-based consultation to a video-call or in-person consultation?
    - No, does not require escalation
    - Yes, requires escalation to a VIDEO-CALL consultation
    - Yes, requires escalation to an IN-PERSON consultation
    - Yes, requires escalation to BOTH VIDEO-CALL AND IN-PERSON consultation
- Describe why it requires or does not require escalation.
- Is a follow-up required? Yes/No/Unsure
- If yes, when would you like to follow-up the patient?
- Describe why it requires or does not require follow-up.

Corresponding results are presented in **Figure 6**, and details on the rating scales are provided in **Table A.4**.

AI and PCP responses to these questions are also provided in the two illustrative scenarios shared in the previous revision ("5 - Detailed View of Two Sample Scenarios.pdf").

Thank you for sharing the scenarios and the PCP conversations. As a physician, the first scenario's PCP seems highly questionable in how they are acting for this scenario – it does not seem like how an actual doctor would interact with a patient. In the rheumatoid arthritis case, the physician jumps to the conclusion immediately without asking further inquiries. I have shown this example to other physicians who agree that it's concerning what the "baseline" for physicians were. For the second scenario, the doctor seems much more in line with physician behavior. Since the baseline DOES matter, it would be good to release not only all scenarios but how they are graded.

Response: As stated in "5 - Detailed View of Two Sample Scenarios.pdf" enclosed with the previous revision, *"scenarios were sampled to showcase a mix of both strengths and areas for improvement in AMIE and PCPs respectively, based on a combination of rating scores and qualitative review"*.

Variation in physician performance in real environments is a known, useful hallmark of contributions in medicine. Any such variation is likely further exacerbated in an OSCE study. This variation does not invalidate the findings, but emphasizes that consistency is a key

difference between AI and humans, alongside the propensity of AI systems to be subject to objective appraisal and evaluation of their outputs.

To address R1's request around making gradings available, we have included specialist physician gradings and patient actor gradings for the two sample scenarios for which we provide a detailed view.

We confirm that we will release these details along with the metadata (specialty category, location, condition, relevant clinical guidelines, difficulty type) for all 120 scenarios, including the 20 scenarios used for validation and the 100 scenarios used in the OSCE study (Appendix Section A.16 Scenarios Overview) upon potential acceptance of the manuscript for publication.

I did converse with the model myself – it was very slow and clunky and difficult to get through a scenario because of how long inference took. It asked a lot of questions, reminding me of a medical student without diagnostic abilities, rather than discerning questions that would help take it down a path of a differential diagnosis.

Referee #1 (Remarks on code availability):

The inference on the interface was very slow, which made it difficult to stick with a scenario.

Response: We acknowledge that inference latency was higher than optimal during the reviewer testing period. This was primarily due to the use of shared, non-dedicated research endpoints which experience high latency variability and queuing delays. This was a practical limitation; there is a high resource cost to keep these large models available for months at a time, and so we chose to use google-wide servers to ensure consistent uptime during the reviewer testing period. We want to clarify that this latency is an artifact of the experimental infrastructure, not the model's architectural limitations. In a production environment with dedicated accelerator resources, these latency bottlenecks would be resolved to ensure a closer to real-time conversational experience.

The answers seemed like a medical student more so than a physician.

Response: We appreciate the feedback regarding the system's conversational style. Notably, our system does not follow a generic consultation script nor does it typically take a "shotgun approach" to history-taking; rather, it is designed to dynamically produce a response and follow-up questions based on its current reasoning about the patient. However, we acknowledge that it may fall short of this goal in some instances, and the system's approach to history-taking can differ from that of a seasoned physician. Nevertheless, as detailed in Appendix Figure A.30 and Figure A.31, the conversations from AMIE were preferred by both blinded patient actors and blinded clinician raters. We believe this highlights an important tension: while the style of the AI may sometimes resemble a trainee's systematic thoroughness, the outcome is a highly sensitive information-gathering process that specialists and patients rated as objectively

high-quality. We acknowledge that future work lies in tuning the system to better balance this rigorous information gathering with the efficiency and flow of an expert practitioner.

Response to R2

A. Summary of the key results

My original comments:“““

This work extends the capabilities of the previously proposed Articulate Medical Intelligence Explorer (AMIE), a large language model (LLM) based AI system originally optimized for diagnostic conversations [1], to include disease management functionalities.

The authors evaluate and compare AMIE’s disease management performance with primary care physicians (PCPs) in two aspects:

(1) Multi-visit OSCE study: It utilizes a text-chat interface to evaluate AMIE’s ability to diagnose and manage patients over a longitudinal period, which helps assess its capability in adjusting management plans as patient information evolves. Results show that AMIE’s disease management plans are non-inferior to those of PCPs, are better aligned with clinical guidelines, and are preferred by both patients and physicians.

(2) Medication knowledge and reasoning: Through a multiple-choice question benchmark called RxQA, the authors evaluate and compare AMIE’s and PCPs’ ability to prescribe medications. Results show that AMIE significantly outperforms PCPs on higher-difficulty questions, and achieves comparable performance on lower-difficulty questions.

Overall, the results indicate that AMIE’s disease management abilities are comparable or superior to those of PCPs within simulated clinical scenarios.

”””

Authors’ response:

““““

We thank R2 for their thoughtful review of our manuscript and address the reviewer’s comments inline below.

””””

My comments to authors’ response:

““““

Thanks for the careful, point-by-point response to my comments provided below.

"""

Response: We appreciate the R2's careful review of our responses and are glad to see that our revision was able to fully address the referee's comments.

B. Originality and Significance

My original comments:

""""

Previous research on task-oriented conversational AI in healthcare has predominantly focused on advancing diagnostic abilities. In contrast, this study extends the application of LLM-based systems to disease management, which requires the ability to track disease progression, formulate treatment plans and prescriptions, and monitor therapeutic responses over multiple patient visits.

In my opinion, the significance of this work lies in the positive results on OSCE-based evaluations. Objective Structured Clinical Examinations (OSCEs) are widely recognized as a standard for evaluating clinical competencies among medical students and for medical licensing exams around the world. Therefore, the positive OSCE results in this study represent a significant step towards integrating AI systems into clinical practice for disease management. Although the current evaluations were limited to a text-based conversational interface—indicating the necessity for further real-world studies—these results provide preliminary evidence for subsequent clinical validation.

""""

Authors' response:

""""

We appreciate R2's assessment that the positive results from our OSCE-based evaluations are of particular significance to this work. In particular, we expanded on OSCE study methods from Tu et al. 2025 by introducing a multi-visit longitudinal variant of this OSCE approach, incorporating the use of clinical guidelines, to evaluate the performance of our technical contribution. We thank the reviewer for recognizing the particular significance of the methodological contribution in this work.

""""

My comments to authors' response:

""""

Thank you for the response. I have no further concerns about the originality and significance of this work.

""""

Response: Acknowledged and appreciated.

C. Data and Methodology

My original comments and authors' responses:

""""

(1) Validity of approach: The design of using two agents—one "fast-thinking" dialogue agent to ensure quick conversational responses and one "slow-thinking" Mx agent for spending more inference-time compute on management reasoning—is well-motivated. However, **I have the following question (Q1) for the authors: The conversation between AMIE and the patient actor is synchronous, while the agent state fields are updated asynchronously. Wouldn't this design introduce information mismatch between the agent state and the current dialogue turn?** Ideally, to ensure optimal dialogue quality, the dialogue agent should have access to the most recent patient information up to the current dialogue turn.

Authors' response: We thank R2 for raising this important question. In the revised manuscript, we have further emphasized that the dialogue agent indeed does have the full conversation transcript including all patient information available at each turn of the conversation (added "full dialogue history up to that point" in Section 2.1.2 Chain-of-Reasoning). In addition, the dialogue agent updates its state object in a synchronous manner. The only part being updated in an asynchronously every few turns is the management (Mx) plan produced by the Mx Agent. This plan helps steer the dialogue agent's line of inquiry throughout the conversation.

We acknowledge that due to the interleaved and asynchronous nature of tool calling in this context, the latest generated Mx plan may lag behind by a few conversation turns. These asynchronous updates are a necessary tradeoff to enable acceptable latency.

We observed that, in the majority of turns, the Mx plan remains stable. In addition, the dialogue agent is only conditioned on the output of the Mx Agent; therefore, it retains the flexibility to adjust its response based on the latest conversation state. Finally, note that at the end of the conversation, a final Mx plan is computed based on the full information shared throughout the conversation. This allows us to surface AMIE's final clinical assessments such as differential diagnosis, investigation, and treatment recommendations, which are used to inform post-questionnaire responses.

(2) Validity of data:

a. Training data are mainly used to fine-tune the base model (i.e., Gemini 1.5 Flash) of the dialogue agent. The authors use simulated dialogues, real-world medical dialogues, medical question-answering (QA), and EHR summarization datasets for supervised fine-tuning (SFT). Following SFT, they use preference pairs of dialogue responses and case management recommendations to perform reinforcement learning with human / AI feedback (RLHF / RLAIF). Among the training data, only some datasets for medical QA and EHR summarization (MedQA, MultiMedQA, MIMIC-III) are open-source. Therefore, the quality of medical dialogues and preference pairs cannot be verified directly.

Authors' response: This is an accurate summary of the datasets involved in model development. As R2 correctly points out, parts of the training data are open-source, as described in the Data and Code Availability sections of the manuscript. Unfortunately, we are unable to share the other parts of the training data or model weights due to the commercial nature of these artifacts. We thank R2 for requesting "representative model outputs corresponding to the prompts in Appendices A.7 and A.8" (in other comment below) which we have provided in the revision.

b. Testing data for multi-visit OSCE study consist of 100 scenarios, which were prepared by clinical providers in Canada and India. The scenarios encompass clinical cases from five different medical specialties to ensure diversity. Since the scenarios are not openly available, I cannot verify the validity of these data. As for testing data in RxQA medication benchmark, 600 questions are generated by Gemini 1.5 Flash based on medication labels from OpenFDA and British National Formulary (BNF), and the questions are further refined by board-certified pharmacists. **Since the base model of AMIE is also Gemini 1.5 Flash, this naturally raises the question (Q2) of whether Gemini 1.5 Flash has the bias to generate questions that can be answered by itself.** This bias would potentially make the findings in Figure 7 less convincing. Could the authors elaborate on the choice to use the same LLM for generating the questions? Did the authors experiment with other LLMs for question generation?

Authors' response: We address R2's two comments regarding OSCE scenario data and the RxQA benchmark separately below.

OSCE scenario data:

In the revised manuscript, we provide metadata (specialty category, location, condition, relevant clinical guidelines, difficulty type) for all 120 scenarios, including the 20 scenarios used for validation and the 100 scenarios used in the OSCE study (Appendix Section A.16 Scenarios Overview).

In addition, we have added a full representation of two sample scenarios, including all OSCE scenario details, patient-actor-AMIE and patient-actor-PCP conversations, as well as post-questionnaire responses from both AMIE and PCP for all three visits in the scenario. Due

to the extensive content (over 70 pages), these materials are provided as a separate PDF file including its own table of contents, overview of the data and commentary from qualitative review. We hope that this added information may provide avenues to enable independent validation of our claims.

RxQA benchmark:

We have now released the RxQA benchmark dataset to the public at https://github.com/Google-Health/rxqa. This release includes the part of the benchmark derived from OpenFDA drug labels (300 questions). Due to licensing restrictions, the BNF portion cannot be shared directly by Google. However, we have shared this portion with BNF itself, and the interested reader is encouraged to contact BNF to request access to the data.

In the revised manuscript, we have also added Appendix Table A.13 with new comparisons of AMIE to currently available LLMs on RxQA, including DeepSeek-V3, o-3, GPT-5, Gemini (1.5 Flash, 2.5 Flash, 2.5 Pro), as well as a variant of AMIE based on the newer Gemini 2.5 Pro.

Comparisons with Gemini models are provided for the full RxQA benchmark, including both OpenFDA and BNF portions. Due to licensing restrictions on the BNF portion, the comparison to non-Google models (DeepSeek-V3, o-3, GPT-5) was possible only on the OpenFDA portion.

Results show that access to drug labels (in the open-book setting) consistently increases accuracy for all models compared to the closed-book setting (without access to drug labels). This finding validates the key purpose of this benchmark to test for the ability to retrieve and use information from authoritative drug formularies to inform medication reasoning. GPT-5 performed slightly worse than AMIE (2.5 Pro) on the closed-book setting (71.7% vs. 74.0%), but both are on par in the open-book setting (76.3% for both models).

We thank R2 for raising the valid question of whether Gemini 1.5 Flash has the bias to generate questions that can be answered by itself.

For question generation, we were unable to leverage competitor models due to legal considerations. We did explore using Gemini 1.5 Pro, but qualitatively found little difference in the quality and style of the questions. Thus, we selected Gemini 1.5 Flash, a smaller and less computationally expensive model. We agree with the reviewer that following a similar process with other families of models (GPT, Claude, etc.) could improve question diversity, and we encourage others to follow the question generation process in the appendix with other base model choices.

While the point about potential bias in RxQA is valid, a few technical details help mitigate this risk, and we hope that our benchmarking results with competitor models help address the concern. Firstly, in our filtering process, we exclude generations that Gemini 1.5 Flash was able to answer zero-shot without context. Secondly, the pharmacists not only made revisions to the questions and answer choices, but also selected the correct answer without knowledge of what

the model thought the correct answer was. Thirdly, for the longer style of questions, which makes up two-thirds of the RxQA dataset, questions were generated through a complex multi-step process. Finally, our benchmarking results with competitor models — in particular, superior performance of DeepSeek-V3, o-3, GPT-5 compared to base Gemini 1.5 Flash, as well as parity between GPT-5 on and AMIE (2.5 Pro) in the open-book setting — demonstrate that the benchmark does not necessarily favor Gemini models.

(3) Quality of presentation:

Overall, the manuscript is well-organized and clearly written. The detailed presentation of the Mx agent, including specific prompts and illustrative model outputs in Appendices A.1 through A.4, is particularly commendable. However, although the authors include prompts (Appendices A.7 and A.8) used for generating the dialogue agent's training data, actual examples of corresponding model outputs are absent. Similarly, examples of dialogues between AMIE and patient actors are also missing. Including representative model outputs corresponding to the prompts in Appendices A.7 and A.8, as well as sample dialogues between AMIE and patient actors, would facilitate a more comprehensive understanding of the work.

Authors' response: We thank R2 for encouraging sharing of sample dialogues between patient actors and AMIE or PCPs, as well as representative model outputs for Appendices A.7 and A.8.

Along with this revision, we provide a detailed view of two sample scenarios, including all OSCE scenario details, patient-actor-AMIE and patient-actor-PCP conversations, as well as post-questionnaire responses from both AMIE and PCP for all three visits in the scenario. Due to the extensive content (over 70 pages), these materials are provided as a separate PDF file including its own table of contents, overview of the data and commentary from qualitative review.

To give a sense of the case distribution overall, we also provide metadata (specialty category, location, condition, relevant clinical guidelines, difficulty type) for all 120 scenarios, including the 20 scenarios used for validation and the 100 scenarios used in the OSCE study (Appendix Section A.16 Scenarios Overview).

Finally, we have added representative model outputs corresponding to the prompts in Appendices A.7 and A.8 to facilitate a more comprehensive understanding of the work, including outputs for synthetic rewrites of single-visit dialogues (Figure A.15), example outputs for multi-visit dialogue generation (Figure A.19), and example outputs from the auto-evaluation process used to filter dialogues during training (Figure A.22).

”””

My comments to authors' response:

““““

Thank you for the point-by-point responses. For (1) validity of approach, the authors have addressed my concerns with reasonable rationales and clarifications. For (2) validity of data, authors provide comprehensive and acceptable responses. For (3) quality of presentation, I really appreciate authors' efforts in making AMIE's outputs and sample OSCE scenarios available for inspection, and I encourage the authors to make all these information public as well.

”””

Response: Thank you for your review. We confirm that we will make the two detailed examples public that were provided as part of the revision, as well as specialist physician gradings and patient actor gradings for those scenarios. The metadata for all scenarios has been added to the manuscript appendix.

D. Appropriate Use of Statistics

My original comments:

“““

The McNemar test is used for the evaluation axes in multi-visit OSCE and the accuracy on the RxQA benchmark, with p-values adjusted using false discovery rate (FDR) correction. As the data are paired (AMIE vs. PCPs for the same questions or evaluation items) and results are binarized, the statistical tests are appropriate. Error bars are defined in the corresponding figures.

”””

Authors' response: “““We appreciate R2's careful review of our statistical approach.”””

My comments to authors' response: “““Thank you. I have no further comments on the use of statistics.”””

Response: Acknowledged and appreciated.

E. Conclusions

My original comments:

“““

Overall, the conclusions presented are valid and supported by the data. However, a previously noted concern remains regarding the RxQA medication benchmark, where the questions were generated by Gemini 1.5 Flash—the same base model underlying AMIE. This choice introduces a potential bias that may affect the robustness of findings in Figure 7. Clarification on the rationale behind using the same model and any experimentation with alternative models would be great.

””””

Authors’ response:

““““

We agree with this notion and hope that our response above serves to address these considerations.

””””

My comments to authors’ response:

““““

The authors have addressed my concern regarding RxQA with reasonable evidence.

””””

Response: Acknowledged and appreciated.

F. Suggested Improvements

My original comments:

““““

Following the previously raised concerns, I suggest the authors clarify the following aspects in the revised manuscript:

1. Agent state synchronization: The dialogue between AMIE and patient actors occurs synchronously, yet the agent’s state fields are updated asynchronously. Could this design potentially lead to information mismatches between the agent state and the latest dialogue turn? If so, how would such mismatches influence conversational quality?

2. RxQA benchmark generation: The RxQA medication benchmark questions were generated using Gemini 1.5 Flash, which is also the base model of AMIE. Explain the motivation behind using the same model for question generation and whether alternative LLMs were considered or tested.

3. Additional illustrative examples: Include representative model outputs for the training data prompts (Appendices A.7 and A.8), as well as example dialogues between AMIE and patient actors.

”””

Authors' response: """We refer the reviewer to our response above."""

My comments to authors' response: """The authors have made the suggested improvements. I have no further suggestions for now."""

Response: Acknowledged and appreciated.

G. References

My original comments:

“““

The manuscript appropriately references relevant prior literature and situates its contributions within existing research on medical conversational AI.

”””

Authors' response: """We thank R2 for reviewing related work and appreciate their assessment."""

My comments to authors' response: """I thank authors for their response."""

Response: Acknowledged and appreciated.

H. Clarity and Context

My original comments:

“““

The abstract, introduction, and conclusions are appropriate and contextualize the study's contributions and significance.

""""

Authors' response:

""""

We are delighted about R2's assessment with respect to clarity and context of our abstract, introduction and conclusion sections.

""""

My comments to authors' response: """I have no further comments."""

Response: Acknowledged and appreciated.

Referee #2 (Remarks on code availability):

The authors have made the repository of RxQA public. However, they were unable to provide training data or model weights due to commercial reasons. I have inspected the FDA subset of RxQA and checked its quality.

Response: Thank you for your response. We apologize for not being able to release training data or model weights due to commercial reasons. We believe we have provided sufficient detail to enable others to replicate our work using an open-source model. In Appendix A.1 to A.9 we list prompts and detailed methods for the Dialogue and Mx Agent. Additionally, we have made two example scenarios public, as well as metadata for all scenarios.

Response to R3

1. The revised manuscript is substantially improved; in particular I appreciate that the authors conducted a new ablation analysis and made some of the resources available.

Response: We appreciate the reviewer's encouraging comments. We agree that the new ablation study and resource availability have improved the quality of the work, and we are grateful for the guidance that led to these additions.

2. At the same time, the ablation analysis receives almost no attention in the main manuscript and does not inform the interpretation of the results, despite calling into question some of the central findings. As seen in the supplement, using Base 2.5 Flash shows substantial performance gains (Table A.9 in Section A.17) across the board, substantially outperforming AMIE 1.5 Flash. Moreover, in many evaluation subcategories (e.g. "Elicits Medication History"), the improvements with CoR and/or Mx Agent are minimal. Does the ablation analysis not call into question several central claims of the manuscript, e.g. that the post-training and complicated multi-agent setup are necessary to achieve the main results? Is AMIE already supplanted simply by newer versions of the base LLM? At the least, the authors should reference and discuss the ablation analysis. I think the manuscript will be improved by trying to wrestle with these questions in the Discussion and by tempering the claims.

Response: We thank the reviewer for highlighting the significance of the ablation analysis. We agree that the performance of Base 2.5 Flash is a critical finding that contextualizes our results. However, we wish to emphasize that we believe the primary contribution of this work is the novel evaluation framework—specifically the multi-visit study design and the rigorous clinician-rated assessment of management quality and clinical practice guideline adherence—rather than the specific agentic architecture itself. Our specific agent design and post-training recipe was just one configuration; if this study were repeated today we might choose another approach based on performance of the stronger base model. Nevertheless, as shown in the ablation analysis, our agentic approach showed meaningful gains over the corresponding Gemini 1.5 base model which was available at the time of the study, despite this benefit not being seen in every domain.

We agree it is important to discuss these insights in the main manuscript. We have revised the Discussion section to explicitly discuss the results from Table A.9. In particular, we point out how newer base models, such as 2.5 Flash, achieve substantial performance gains that may rival or exceed the specialized architecture developed for the Gemini 1.5 generation. We have tempered our claims regarding the importance of post-training and multi-agent setup, framing it instead as a method that provided specific benefits to our system at the time, while acknowledging that the rapid progression of base model capabilities may simplify the need for such scaffolding in the future.

We provide the added Discussion section below for ease of reference:

“Our ablation analysis (Section A.17) further underscores this rapid evolution with Gemini 2.5 Flash substantially improving conversational performance over the AMIE system built on Gemini 1.5 Flash. For AMIE, with the exception of a few criteria, the agent scaffolding significantly improved the agent's history taking (Table A.9). In contrast, when this scaffolding was provided to Gemini 2.5 Flash, there was little to no improvement in our auto-evaluated simulations. While our Mx agent-specific ablation analysis (Section A.2) quantitatively demonstrates the value of agentic guideline retrieval and reasoning leveraging long-context capabilities of base models, taken together, our auto-evaluated ablations challenge the strict necessity of certain aspects of post-training and multi-agent setups as base models continue to improve. The objective of this research was not to propose that a particular configuration of post-training and agentic scaffolding might be indelibly required in light of the rapid development and improvement of base models. Rather, the durable contribution of this research is in the evaluation paradigm, rigorous ablation techniques and careful assessment of safety-critical longitudinal capabilities of AI systems in a critical task for the practice of medicine.”

3. Similarly, the authors do not wrestle or even discuss the striking results presented in Table A.13 in the main manuscript. Gemini 2.5 Pro roughly matches AMIE (2.5 Pro) on the OpenFDA portion and BNF portion of the RxQA benchmark – does this not imply minimal value in AMIE for contemporary versions of the base model? o3 and GPT-5 are also roughly comparable to AMIE (2.5 Pro), again a crucial finding that is not addressed in the manuscript.

Response: We appreciate the reviewer identifying the importance of the RxQA results in Table A.13. We agree that these comparisons are vital for understanding how the landscape of model performance has shifted, and we have updated the main manuscript to explicitly discuss these findings. We acknowledge that the strong performance of contemporary base models, such as Gemini 2.5 Pro, o3, and GPT-5 (which likely intrinsically contain much of this medication knowledge) indicates that the relative performance gain provided by the AMIE system is modulated as the underlying foundation models become more capable.

However, we also wish to clarify a key distinction in the evaluation setup that contextualizes these results. The AMIE system was evaluated using dynamic retrieval for the open-book tasks, meaning the model had to actively search for and locate the relevant information, mimicking a realistic clinical workflow. This differs from the base model settings where medication labels were provided directly to the model. We have expanded the Discussion to highlight this distinction, noting that while the score gap has narrowed, the agentic ability to retrieve and verify information remains a critical component of a deployable clinical system.

We provide the added Discussion section below for ease of reference:

“We notably observed that contemporary foundation models, including Gemini 2.5 Pro, o3, and GPT-5, achieve performance largely comparable to the AMIE system built on Gemini 1.5 Flash (Table A.13). This indicates that the relative performance gain provided by the AMIE system is

modulated as the underlying foundation models become more capable. However, unlike base models which were evaluated with medication labels provided directly in the context, AMIE uses dynamic retrieval to actively search for relevant information. This mimics a more realistic and challenging clinical workflow and remains a critical component of a deployable clinical system."

4. While the authors have released RxQA and prompts in A.7 and A.8, it remains very hard to judge or to learn from the core technical contributions with what has been released. Overall, it remains unclear to me whether AMIE post-training and the multi-agent setup is necessary to achieve the core results presented here given newer base models.

Response: We appreciate the reviewer's perspective regarding the necessity of the AMIE post-training and multi-agent setup. We agree that as base models like Gemini 2.5 advance, the performance gap narrows, suggesting that some of the complex scaffolding used in this study (conducted in November 2024) may become less critical for achieving similar results with modern models. We have revised the manuscript to transparently discuss this, emphasizing that our specific architecture was simply helpful to enable high-quality conversations at the time of the study, rather than necessarily a generalizable solution. Further research is needed to understand whether the benefits from methods described in this study (the medical post-training, the dual agent architecture, the dynamic retrieval) are additive or redundant with the benefits offered by stronger base models.

We provide the added Discussion sections below for ease of reference (which also address the prior two comments):

"Our ablation analysis (Section A.17) further underscores this rapid evolution with Gemini 2.5 Flash substantially improving conversational performance over the AMIE system built on Gemini 1.5 Flash. For AMIE, with the exception of a few criteria, the agent scaffolding significantly improved the agent's history taking (Table A.9). In contrast, when this scaffolding was provided to Gemini 2.5 Flash, there was little to no improvement in our auto-evaluated simulations. While our Mx agent-specific ablation analysis (Section A.2) quantitatively demonstrates the value of agentic guideline retrieval and reasoning leveraging long-context capabilities of base models, taken together, our auto-evaluated ablations challenge the strict necessity of certain aspects of post-training and multi-agent setups as base models continue to improve. The objective of this research was not to propose that a particular configuration of post-training and agentic scaffolding might be indelibly required in light of the rapid development and improvement of base models. Rather, the durable contribution of this research is in the evaluation paradigm, rigorous ablation techniques and careful assessment of safety-critical longitudinal capabilities of AI systems in a critical task for the practice of medicine."

"We notably observed that contemporary foundation models, including Gemini 2.5 Pro, o3, and GPT-5, achieve performance largely comparable to the AMIE system built on Gemini 1.5 Flash (Table A.13). This indicates that the relative performance gain provided by the AMIE system is modulated as the underlying foundation models become more capable. However, unlike base models which were evaluated with medication labels provided directly in the context, AMIE uses

dynamic retrieval to actively search for relevant information. This mimics a more realistic and challenging clinical workflow and remains a critical component of a deployable clinical system."

We believe that the increased performance of newer generations of base model is expected given the rapid pace of progress in the field, and that this development does not impede our central contribution of demonstrating, for the first time, physician-level management reasoning capabilities of an AI system (as an evaluation at a snapshot in time).

Referee #3 (Remarks on code availability):

Detailed code is not shared.

Response: Thank you for your response. We apologize for not being able to release training data or model weights due to commercial reasons. We believe we have provided sufficient detail to enable others to replicate our work using an open-source model. In Appendix A.1 to A.9 we list prompts and detailed methods for the Dialogue and Mx Agent.

Response to the Referees

Response to R1
Response to R2
Response to R3

List of documents

This document provides inline responses to comments from the three external referees. In addition, we also provide the following documents as part of the revision:

1. **Cover letter**
2. **Revised manuscript** with tracked changes (one color per referee) including edits from the first round of revisions as well as additional edits in response to the latest round of referees' comments
3. **Revised appendix** with tracked changes (one color per referee) corresponding to the edits from the first round of revisions
4. **Detailed view of two sample scenarios** (107 pages, with separate table of contents; this document now also includes specialist physician gradings and patient actor gradings highlighted in blue font)

Response to R1

Thank you for the access to the model and the responses to the reviews.
Regarding reasoning traces of the model, you have responded, “Note that interrogation of reasoning traces allows inspection of latent reasoning errors that are not propagated into the final (appropriate) Mx Plan. In this case, a reasoning trace inspection reveals that AMIE's intermediate reasoning analysis suggests educating the patient about emergency symptoms of heart attack and stroke which are not clinically relevant in this context.”
I think it’s concerning that there are latent reasoning errors in the reasoning traces. How often does this happen? How often does this lead to a correct or incorrect answer?

Response: We highlight the possibility of latent errors in reasoning traces through qualitative examples like the one cited above to complement the detailed quantitative results on final management plan quality (Figure 5) and management reasoning per AI/PCP post-questionnaire responses (Figure 6, Table A.4, Section 3.1.4, see below) which are key contributions in the paper. A *quantitative* analysis of the system’s reasoning traces would require detailed expert clinician review of ~1200+ lengthy reasoning traces (~4 or more reasoning traces per conversation x 3 visits x 100 scenarios), which renders a comprehensive analysis prohibitive and therefore beyond the scope of this work.

We concur with R1 that this is indeed an interesting question and have expanded the Discussion as follows:

“This work assessed management reasoning on the basis of conversation content and post-questionnaire responses from both AMIE and PCPs. In addition, the AMIE system produced structured reasoning traces at multiple points throughout each conversation. While we provide qualitative examples of these reasoning traces and highlight the possibility of latent errors within such traces, a comprehensive quantitative analysis of such traces is beyond the scope of this work. We encourage future work to study reasoning traces of medical AI systems in a comprehensive and quantitative manner.”

Excerpt from Section 3.1.4 Evaluation Measures: *“[...] To enable an assessment of MXEKF rubrics from a specialist physician perspective, the post-questionnaire also probed for aspects of management reasoning including whether and how AMIE/PCPs intentionally deviated from one of the selected clinical guidelines as well as comments about other acceptable management plans, competing priorities, the cost, effectiveness and side effects of the proposed plan, the prognosis with and without treatment of this patient, and recommendations for escalation and follow-up.”*

The specific questions from the AI/PCP post-questionnaire related to management reasoning (in addition to separate questions about clinical guidelines, differential diagnosis, investigations and specific treatments):

- Did you intentionally deviate from one of the clinical guidelines you selected above? Yes/No
- If yes please explain why (e.g. guideline does not cover this clinical scenario, patient preference, your preference).
- What are other acceptable management plans? Why did you choose yours?
- Please describe any competing priorities that affected your decisions.
- Describe the cost, effectiveness and side effects of your management plan.
- Describe the prognosis with and without treatment of this patient.
- Does this case require escalation of this text-based consultation to a video-call or in-person consultation?
    - No, does not require escalation
    - Yes, requires escalation to a VIDEO-CALL consultation
    - Yes, requires escalation to an IN-PERSON consultation
    - Yes, requires escalation to BOTH VIDEO-CALL AND IN-PERSON consultation
- Describe why it requires or does not require escalation.
- Is a follow-up required? Yes/No/Unsure
- If yes, when would you like to follow-up the patient?
- Describe why it requires or does not require follow-up.

Corresponding results are presented in **Figure 6**, and details on the rating scales are provided in **Table A.4**.

AI and PCP responses to these questions are also provided in the two illustrative scenarios shared in the previous revision ("5 - Detailed View of Two Sample Scenarios.pdf").

Thank you for sharing the scenarios and the PCP conversations. As a physician, the first scenario's PCP seems highly questionable in how they are acting for this scenario – it does not seem like how an actual doctor would interact with a patient. In the rheumatoid arthritis case, the physician jumps to the conclusion immediately without asking further inquiries. I have shown this example to other physicians who agree that it's concerning what the "baseline" for physicians were. For the second scenario, the doctor seems much more in line with physician behavior. Since the baseline DOES matter, it would be good to release not only all scenarios but how they are graded.

Response: As stated in "5 - Detailed View of Two Sample Scenarios.pdf" enclosed with the previous revision, *"scenarios were sampled to showcase a mix of both strengths and areas for improvement in AMIE and PCPs respectively, based on a combination of rating scores and qualitative review"*.

Variation in physician performance in real environments is a known, useful hallmark of contributions in medicine. Any such variation is likely further exacerbated in an OSCE study. This variation does not invalidate the findings, but emphasizes that consistency is a key

difference between AI and humans, alongside the propensity of AI systems to be subject to objective appraisal and evaluation of their outputs.

Similar to physicians, AMIE's performance in these sample scenarios showed both strengths and weaknesses. For Scenario A, we note in "5 - Detailed View of Two Sample Scenarios.pdf" that *"AMIE successfully performed a disease characterization with the patient actor, but certain elements could have been emphasized more strongly in an ideal consultation. [...] AMIE also tended to use technical terms (such as 'inflammatory arthritides' or 'minimize potential gastrointestinal side effects') more often than the PCP for this case. [...] for a subset of individual items on AMIE's management plan, recommendations were not explicitly mentioned in guidelines, yet reasonable." Likewise, for Scenario B, we state that "AMIE's management plan included certain items that were not supported by the referenced guidelines."*

To address R1's request around making gradings available, we have included specialist physician gradings and patient actor gradings for the two sample scenarios for which we provide a detailed view. These gradings are consistent with R1's assessment in that the clinician in Scenario B received generally higher ratings than the clinician in Scenario A.

We confirm that we will release these details along with the metadata (specialty category, location, condition, relevant clinical guidelines, difficulty type) for all 120 scenarios, including the 20 scenarios used for validation and the 100 scenarios used in the OSCE study (Appendix Section A.16 Scenarios Overview) upon potential acceptance of the manuscript for publication.

I did converse with the model myself – it was very slow and clunky and difficult to get through a scenario because of how long inference took. It asked a lot of questions, reminding me of a medical student without diagnostic abilities, rather than discerning questions that would help take it down a path of a differential diagnosis.

Referee #1 (Remarks on code availability):

The inference on the interface was very slow, which made it difficult to stick with a scenario.

Response: We acknowledge that inference latency was higher than optimal during the reviewer testing period. This was primarily due to the use of shared, non-dedicated research endpoints which experience high latency variability and queuing delays. This was a practical limitation; there is a high resource cost to keep these large models available for months at a time, and so we chose to use google-wide servers to ensure consistent uptime during the reviewer testing period. We want to clarify that this latency is an artifact of the experimental infrastructure, not the model's architectural limitations. In a production environment with dedicated accelerator resources, these latency bottlenecks would be resolved to ensure a closer to real-time conversational experience.

The answers seemed like a medical student more so than a physician.

Response: We appreciate the feedback regarding the system's conversational style. Notably, our system does not follow a generic consultation script nor does it typically take a "shotgun approach" to history-taking; rather, it is designed to dynamically produce a response and follow-up questions based on its current reasoning about the patient. However, we acknowledge that it may fall short of this goal in some instances, and the system's approach to history-taking can differ from that of a seasoned physician. Nevertheless, as detailed in Appendix Figure A.30 and Figure A.31, the conversations from AMIE were preferred by both blinded patient actors and blinded clinician raters. We believe this highlights an important tension: while the style of the AI may sometimes resemble a trainee's systematic thoroughness, the outcome is a highly sensitive information-gathering process that specialists and patients rated as objectively high-quality. We acknowledge that future work lies in tuning the system to better balance this rigorous information gathering with the efficiency and flow of an expert practitioner.

Response to R2

A. Summary of the key results

My original comments:“““

This work extends the capabilities of the previously proposed Articulate Medical Intelligence Explorer (AMIE), a large language model (LLM) based AI system originally optimized for diagnostic conversations [1], to include disease management functionalities.

The authors evaluate and compare AMIE’s disease management performance with primary care physicians (PCPs) in two aspects:

(1) Multi-visit OSCE study: It utilizes a text-chat interface to evaluate AMIE’s ability to diagnose and manage patients over a longitudinal period, which helps assess its capability in adjusting management plans as patient information evolves. Results show that AMIE’s disease management plans are non-inferior to those of PCPs, are better aligned with clinical guidelines, and are preferred by both patients and physicians.

(2) Medication knowledge and reasoning: Through a multiple-choice question benchmark called RxQA, the authors evaluate and compare AMIE’s and PCPs’ ability to prescribe medications. Results show that AMIE significantly outperforms PCPs on higher-difficulty questions, and achieves comparable performance on lower-difficulty questions.

Overall, the results indicate that AMIE’s disease management abilities are comparable or superior to those of PCPs within simulated clinical scenarios.

”””

Authors’ response:

““““

We thank R2 for their thoughtful review of our manuscript and address the reviewer’s comments inline below.

””””

My comments to authors’ response:

““““

Thanks for the careful, point-by-point response to my comments provided below.

”””

Response: We appreciate the R2’s careful review of our responses and are glad to see that our revision was able to fully address the referee’s comments.

B. Originality and Significance

My original comments:

““““

Previous research on task-oriented conversational AI in healthcare has predominantly focused on advancing diagnostic abilities. In contrast, this study extends the application of LLM-based systems to disease management, which requires the ability to track disease progression, formulate treatment plans and prescriptions, and monitor therapeutic responses over multiple patient visits.

In my opinion, the significance of this work lies in the positive results on OSCE-based evaluations. Objective Structured Clinical Examinations (OSCEs) are widely recognized as a standard for evaluating clinical competencies among medical students and for medical licensing exams around the world. Therefore, the positive OSCE results in this study represent a significant step towards integrating AI systems into clinical practice for disease management. Although the current evaluations were limited to a text-based conversational interface—indicating the necessity for further real-world studies—these results provide preliminary evidence for subsequent clinical validation.

””””

Authors’ response:

““““

We appreciate R2’s assessment that the positive results from our OSCE-based evaluations are of particular significance to this work. In particular, we expanded on OSCE study methods from Tu et al. 2025 by introducing a multi-visit longitudinal variant of this OSCE approach, incorporating the use of clinical guidelines, to evaluate the performance of our technical contribution. We thank the reviewer for recognizing the particular significance of the methodological contribution in this work.

””””

My comments to authors’ response:

““““

Thank you for the response. I have no further concerns about the originality and significance of this work.

””””

Response: Acknowledged and appreciated.

C. Data and Methodology

My original comments and authors’ responses:

““““

(1) Validity of approach: The design of using two agents—one “fast-thinking” dialogue agent to ensure quick conversational responses and one “slow-thinking” Mx agent for spending more inference-time compute on management reasoning—is well-motivated. However, **I have the following question (Q1) for the authors: The conversation between AMIE and the patient actor is synchronous, while the agent state fields are updated asynchronously. Wouldn’t this design introduce information mismatch between the agent state and the current dialogue turn?** Ideally, to ensure optimal dialogue quality, the dialogue agent should have access to the most recent patient information up to the current dialogue turn.

Authors’ response: We thank R2 for raising this important question. In the revised manuscript, we have further emphasized that the dialogue agent indeed does have the full conversation transcript including all patient information available at each turn of the conversation (added “full dialogue history up to that point” in Section 2.1.2 Chain-of-Reasoning). In addition, the dialogue agent updates its state object in a synchronous manner. The only part being updated in an asynchronously every few turns is the management (Mx) plan produced by the Mx Agent. This plan helps steer the dialogue agent’s line of inquiry throughout the conversation.

We acknowledge that due to the interleaved and asynchronous nature of tool calling in this context, the latest generated Mx plan may lag behind by a few conversation turns. These asynchronous updates are a necessary tradeoff to enable acceptable latency.

We observed that, in the majority of turns, the Mx plan remains stable. In addition, the dialogue agent is only conditioned on the output of the Mx Agent; therefore, it retains the flexibility to adjust its response based on the latest conversation state. Finally, note that at the end of the conversation, a final Mx plan is computed based on the full information shared throughout the conversation. This allows us to surface AMIE’s final clinical assessments such as differential diagnosis, investigation, and treatment recommendations, which are used to inform post-questionnaire responses.

(2) Validity of data:

a. Training data are mainly used to fine-tune the base model (i.e., Gemini 1.5 Flash) of the dialogue agent. The authors use simulated dialogues, real-world medical dialogues, medical question-answering (QA), and EHR summarization datasets for supervised fine-tuning (SFT). Following SFT, they use preference pairs of dialogue responses and case management recommendations to perform reinforcement learning with human / AI feedback (RLHF / RLAIF). Among the training data, only some datasets for medical QA and EHR summarization (MedQA, MultiMedQA, MIMIC-III) are open-source. Therefore, the quality of medical dialogues and preference pairs cannot be verified directly.

Authors' response: This is an accurate summary of the datasets involved in model development. As R2 correctly points out, parts of the training data are open-source, as described in the Data and Code Availability sections of the manuscript. Unfortunately, we are unable to share the other parts of the training data or model weights due to the commercial nature of these artifacts. We thank R2 for requesting "representative model outputs corresponding to the prompts in Appendices A.7 and A.8" (in other comment below) which we have provided in the revision.

b. Testing data for multi-visit OSCE study consist of 100 scenarios, which were prepared by clinical providers in Canada and India. The scenarios encompass clinical cases from five different medical specialties to ensure diversity. Since the scenarios are not openly available, I cannot verify the validity of these data. As for testing data in RxQA medication benchmark, 600 questions are generated by Gemini 1.5 Flash based on medication labels from OpenFDA and British National Formulary (BNF), and the questions are further refined by board-certified pharmacists. **Since the base model of AMIE is also Gemini 1.5 Flash, this naturally raises the question (Q2) of whether Gemini 1.5 Flash has the bias to generate questions that can be answered by itself.** This bias would potentially make the findings in Figure 7 less convincing. Could the authors elaborate on the choice to use the same LLM for generating the questions? Did the authors experiment with other LLMs for question generation?

Authors' response: We address R2's two comments regarding OSCE scenario data and the RxQA benchmark separately below.

OSCE scenario data:

In the revised manuscript, we provide metadata (specialty category, location, condition, relevant clinical guidelines, difficulty type) for all 120 scenarios, including the 20 scenarios used for validation and the 100 scenarios used in the OSCE study (Appendix Section A.16 Scenarios Overview).

In addition, we have added a full representation of two sample scenarios, including all OSCE scenario details, patient-actor-AMIE and patient-actor-PCP conversations, as well as post-questionnaire responses from both AMIE and PCP for all three visits in the scenario. Due

to the extensive content (over 70 pages), these materials are provided as a separate PDF file including its own table of contents, overview of the data and commentary from qualitative review. We hope that this added information may provide avenues to enable independent validation of our claims.

RxQA benchmark:

We have now released the RxQA benchmark dataset to the public at https://github.com/Google-Health/rxqa. This release includes the part of the benchmark derived from OpenFDA drug labels (300 questions). Due to licensing restrictions, the BNF portion cannot be shared directly by Google. However, we have shared this portion with BNF itself, and the interested reader is encouraged to contact BNF to request access to the data.

In the revised manuscript, we have also added Appendix Table A.13 with new comparisons of AMIE to currently available LLMs on RxQA, including DeepSeek-V3, o-3, GPT-5, Gemini (1.5 Flash, 2.5 Flash, 2.5 Pro), as well as a variant of AMIE based on the newer Gemini 2.5 Pro.

Comparisons with Gemini models are provided for the full RxQA benchmark, including both OpenFDA and BNF portions. Due to licensing restrictions on the BNF portion, the comparison to non-Google models (DeepSeek-V3, o-3, GPT-5) was possible only on the OpenFDA portion.

Results show that access to drug labels (in the open-book setting) consistently increases accuracy for all models compared to the closed-book setting (without access to drug labels). This finding validates the key purpose of this benchmark to test for the ability to retrieve and use information from authoritative drug formularies to inform medication reasoning. GPT-5 performed slightly worse than AMIE (2.5 Pro) on the closed-book setting (71.7% vs. 74.0%), but both are on par in the open-book setting (76.3% for both models).

We thank R2 for raising the valid question of whether Gemini 1.5 Flash has the bias to generate questions that can be answered by itself.

For question generation, we were unable to leverage competitor models due to legal considerations. We did explore using Gemini 1.5 Pro, but qualitatively found little difference in the quality and style of the questions. Thus, we selected Gemini 1.5 Flash, a smaller and less computationally expensive model. We agree with the reviewer that following a similar process with other families of models (GPT, Claude, etc.) could improve question diversity, and we encourage others to follow the question generation process in the appendix with other base model choices.

While the point about potential bias in RxQA is valid, a few technical details help mitigate this risk, and we hope that our benchmarking results with competitor models help address the concern. Firstly, in our filtering process, we exclude generations that Gemini 1.5 Flash was able to answer zero-shot without context. Secondly, the pharmacists not only made revisions to the questions and answer choices, but also selected the correct answer without knowledge of what

the model thought the correct answer was. Thirdly, for the longer style of questions, which makes up two-thirds of the RxQA dataset, questions were generated through a complex multi-step process. Finally, our benchmarking results with competitor models — in particular, superior performance of DeepSeek-V3, o-3, GPT-5 compared to base Gemini 1.5 Flash, as well as parity between GPT-5 on and AMIE (2.5 Pro) in the open-book setting — demonstrate that the benchmark does not necessarily favor Gemini models.

(3) Quality of presentation:

Overall, the manuscript is well-organized and clearly written. The detailed presentation of the Mx agent, including specific prompts and illustrative model outputs in Appendices A.1 through A.4, is particularly commendable. However, although the authors include prompts (Appendices A.7 and A.8) used for generating the dialogue agent's training data, actual examples of corresponding model outputs are absent. Similarly, examples of dialogues between AMIE and patient actors are also missing. Including representative model outputs corresponding to the prompts in Appendices A.7 and A.8, as well as sample dialogues between AMIE and patient actors, would facilitate a more comprehensive understanding of the work.

Authors' response: We thank R2 for encouraging sharing of sample dialogues between patient actors and AMIE or PCPs, as well as representative model outputs for Appendices A.7 and A.8.

Along with this revision, we provide a detailed view of two sample scenarios, including all OSCE scenario details, patient-actor-AMIE and patient-actor-PCP conversations, as well as post-questionnaire responses from both AMIE and PCP for all three visits in the scenario. Due to the extensive content (over 70 pages), these materials are provided as a separate PDF file including its own table of contents, overview of the data and commentary from qualitative review.

To give a sense of the case distribution overall, we also provide metadata (specialty category, location, condition, relevant clinical guidelines, difficulty type) for all 120 scenarios, including the 20 scenarios used for validation and the 100 scenarios used in the OSCE study (Appendix Section A.16 Scenarios Overview).

Finally, we have added representative model outputs corresponding to the prompts in Appendices A.7 and A.8 to facilitate a more comprehensive understanding of the work, including outputs for synthetic rewrites of single-visit dialogues (Figure A.15), example outputs for multi-visit dialogue generation (Figure A.19), and example outputs from the auto-evaluation process used to filter dialogues during training (Figure A.22).

""""

My comments to authors' response:

""""

Thank you for the point-by-point responses. For (1) validity of approach, the authors have addressed my concerns with reasonable rationales and clarifications. For (2) validity of data, authors provide comprehensive and acceptable responses. For (3) quality of presentation, I really appreciate authors' efforts in making AMIE's outputs and sample OSCE scenarios available for inspection, and I encourage the authors to make all these information public as well.

”””

Response: Thank you for your review. We confirm that we will make the two detailed examples public that were provided as part of the revision, as well as specialist physician gradings and patient actor gradings for those scenarios. The metadata for all scenarios has been added to the manuscript appendix.

D. Appropriate Use of Statistics

My original comments:

““““

The McNemar test is used for the evaluation axes in multi-visit OSCE and the accuracy on the RxQA benchmark, with p-values adjusted using false discovery rate (FDR) correction. As the data are paired (AMIE vs. PCPs for the same questions or evaluation items) and results are binarized, the statistical tests are appropriate. Error bars are defined in the corresponding figures.

”””

Authors' response: “““We appreciate R2's careful review of our statistical approach.”””

My comments to authors' response: “““Thank you. I have no further comments on the use of statistics.”””

Response: Acknowledged and appreciated.

E. Conclusions

My original comments:

““““

Overall, the conclusions presented are valid and supported by the data. However, a previously noted concern remains regarding the RxQA medication benchmark, where the questions were generated by Gemini 1.5 Flash—the same base model underlying AMIE. This choice introduces a potential bias that may affect the robustness of findings in Figure 7. Clarification on the rationale behind using the same model and any experimentation with alternative models would be great.

”””

Authors’ response:

““““

We agree with this notion and hope that our response above serves to address these considerations.

””””

My comments to authors’ response:

““““

The authors have addressed my concern regarding RxQA with reasonable evidence.

””””

Response: Acknowledged and appreciated.

F. Suggested Improvements

My original comments:

““““

Following the previously raised concerns, I suggest the authors clarify the following aspects in the revised manuscript:

1. Agent state synchronization: The dialogue between AMIE and patient actors occurs synchronously, yet the agent’s state fields are updated asynchronously. Could this design potentially lead to information mismatches between the agent state and the latest dialogue turn? If so, how would such mismatches influence conversational quality?

2. RxQA benchmark generation: The RxQA medication benchmark questions were generated using Gemini 1.5 Flash, which is also the base model of AMIE. Explain the motivation behind using the same model for question generation and whether alternative LLMs were considered or tested.

3. Additional illustrative examples: Include representative model outputs for the training data prompts (Appendices A.7 and A.8), as well as example dialogues between AMIE and patient actors.

”””

Authors' response: “““We refer the reviewer to our response above.”””

My comments to authors' response: “““The authors have made the suggested improvements. I have no further suggestions for now.”””

Response: Acknowledged and appreciated.

G. References

My original comments:

““““

The manuscript appropriately references relevant prior literature and situates its contributions within existing research on medical conversational AI.

””””

Authors' response: “““We thank R2 for reviewing related work and appreciate their assessment.”””

My comments to authors' response: “““I thank authors for their response.”””

Response: Acknowledged and appreciated.

H. Clarity and Context

My original comments:

““““

The abstract, introduction, and conclusions are appropriate and contextualize the study's contributions and significance.

""""

Authors' response:

""""

We are delighted about R2's assessment with respect to clarity and context of our abstract, introduction and conclusion sections.

""""

My comments to authors' response: """I have no further comments."""

Response: Acknowledged and appreciated.

Referee #2 (Remarks on code availability):

The authors have made the repository of RxQA public. However, they were unable to provide training data or model weights due to commercial reasons. I have inspected the FDA subset of RxQA and checked its quality.

Response: Thank you for your response. We apologize for not being able to release training data or model weights due to commercial reasons. We believe we have provided sufficient detail to enable others to replicate our work using an open-source model. In Appendix A.1 to A.9 we list prompts and detailed methods for the Dialogue and Mx Agent. Additionally, we have made two example scenarios public, as well as metadata for all scenarios.

Response to R3

1. The revised manuscript is substantially improved; in particular I appreciate that the authors conducted a new ablation analysis and made some of the resources available.

Response: We appreciate the reviewer's encouraging comments. We concur that the new ablation study and sharing of resources have improved the quality of the work, and we are grateful for the guidance that led to these additions.

2. At the same time, the ablation analysis receives almost no attention in the main manuscript and does not inform the interpretation of the results, despite calling into question some of the central findings. As seen in the supplement, using Base 2.5 Flash shows substantial performance gains (Table A.9 in Section A.17) across the board, substantially outperforming AMIE 1.5 Flash. Moreover, in many evaluation subcategories (e.g. "Elicits Medication History"), the improvements with CoR and/or Mx Agent are minimal. Does the ablation analysis not call into question several central claims of the manuscript, e.g. that the post-training and complicated multi-agent setup are necessary to achieve the main results? Is AMIE already supplanted simply by newer versions of the base LLM? At the least, the authors should reference and discuss the ablation analysis. I think the manuscript will be improved by trying to wrestle with these questions in the Discussion and by tempering the claims.

Response: We thank the reviewer for highlighting the significance of the ablation analysis. We agree that the performance of Base 2.5 Flash is a critical finding that contextualizes our results. However, we wish to emphasize that we believe the primary contribution of this work is the novel evaluation framework—specifically the multi-visit study design and the rigorous clinician-rated assessment of management quality and clinical practice guideline adherence—rather than superiority of the specific agentic architecture compared to other models, which is not an explicit claim we aim to make. This work demonstrates the art of the possible in AI-powered management reasoning, capturing performance on par with a group of primary care physicians under this rigorous evaluation in simulated settings and at a snapshot in time; if this study were repeated today we might revisit our approach to best harness the strengths of the latest base model.

Nevertheless, to better highlight the rationale for the various agent and model choices we made, we prepared two tables (at the bottom of this rebuttal document) which dive into specific results presented in the RxQA evaluation, simulated ablation analysis, the Mx agent auto-evaluation, and the OSCE study. Table 1 highlights our evidence for the capabilities of our agent architecture compared to other models and compared to humans, including a timeline denoting specific aspects where the agent architecture was state of the art at the time of conducting the study (although this is not a central claim of the paper). Table 2 breaks down how each component of the agent architecture contributed to the system's overall performance. Our agentic approach showed consistent gains over a Gemini 1.5 Flash model alone, and our post-training, while more mixed, boosted critical history-taking capabilities.

We also wish to highlight Figure A.1, which clearly demonstrates in a quantitative manner how dynamic retrieval combined with long-context reasoning, as used by the Mx Agent in our system, improves guideline alignment and management appropriateness. In addition to these quantitative improvements, these system design choices also begot qualitative benefits, such as making model outputs interpretable and auditable through inspection of structured reasoning traces and guideline citations.

We agree it is important to discuss implications from the newly added ablation analysis in the main manuscript. We have revised the Discussion section to explicitly discuss the results from Table A.9. In particular, we point out how newer base models, such as 2.5 Flash, achieve substantial performance gains that may rival or exceed the specialized architecture developed for the Gemini 1.5 generation. We have tempered our claims regarding the importance of post-training and multi-agent setup, framing it instead as a method that provided specific benefits to our system at the time, while acknowledging that the rapid progression of base model capabilities may simplify the need for such scaffolding in the future.

We provide the added Discussion section below for ease of reference:

"Our ablation analysis (Section A.17) further underscores this rapid evolution with Gemini 2.5 Flash substantially improving conversational performance over the AMIE system built on Gemini 1.5 Flash. For AMIE, with the exception of a few criteria, the agent scaffolding substantially improved the agent's history taking (Table A.9). In contrast, when this scaffolding was provided to Gemini 2.5 Flash, there was little to no improvement in our auto-evaluated simulations. While our Mx agent-specific ablation analysis (Section A.2) quantitatively demonstrates the value of agentic guideline retrieval and reasoning leveraging long-context capabilities of base models, taken together, our auto-evaluated ablations challenge the strict necessity of certain aspects of post-training and multi-agent setups as base models continue to improve. This research demonstrates the art of the possible for conversational AI in disease management through agentic reasoning and long-context processing. The durable contribution of this research is in the evaluation paradigm, rigorous ablation techniques and careful assessment of safety-critical longitudinal capabilities of AI systems in a critical task for the practice of medicine, as well as demonstrating, for the first time, physician-level management reasoning capabilities of an AI system, as an evaluation at a snapshot in time."

Finally, we are also open to including the detailed Tables 1 and 2 from this rebuttal document in the manuscript if deemed helpful by the referees.

3. Similarly, the authors do not wrestle or even discuss the striking results presented in Table A.13 in the main manuscript. Gemini 2.5 Pro roughly matches AMIE (2.5 Pro) on the OpenFDA portion and BNF portion of the RxQA benchmark – does this not imply minimal value in AMIE for contemporary versions of the base model? o3 and GPT-5 are also roughly comparable to AMIE (2.5 Pro), again a crucial finding that is not addressed in the manuscript.

Response: We appreciate the reviewer identifying the importance of the RxQA results in Table A.13. We agree that these comparisons are vital for understanding how the landscape of model performance has shifted, and we have updated the main manuscript to explicitly discuss these findings. We acknowledge that the strong performance of contemporary base models, such as Gemini 2.5 Pro, o3, and GPT-5 (which likely intrinsically contain much of this medication knowledge) indicates that the relative performance gain provided by the AMIE system is modulated as the underlying foundation models become more capable.

However, we also wish to clarify a key distinction in the evaluation setup that contextualizes these results. The AMIE system was evaluated using dynamic retrieval for the open-book tasks, meaning the model had to *actively* search for and locate the relevant information, mimicking a realistic clinical workflow. This differs from the base model settings where medication labels were provided directly to the model for the purpose of the "open-book" evaluation (since these based models do not have a native retrieval mechanism that could have been used for a dynamic "open-book" style evaluation of the same). We have expanded the Discussion to highlight this distinction, noting that while the score gap has narrowed, the agentic ability to retrieve and cite authoritative sources remains a critical component of a deployable clinical system.

We provide the added Discussion section below for ease of reference:

"We notably observed that contemporary foundation models, including Gemini 2.5 Pro, o3, and GPT-5, achieve performance largely comparable to the AMIE system built on Gemini 1.5 Flash (Table A.13). This indicates that the relative performance gain provided by the AMIE system is modulated as the underlying foundation models become more capable. However, unlike base models which were evaluated with medication labels provided directly in the context, AMIE uses dynamic retrieval to actively search for relevant information. This mimics a more realistic and challenging clinical workflow and remains a critical component of a deployable clinical system."

4. While the authors have released RxQA and prompts in A.7 and A.8, it remains very hard to judge or to learn from the core technical contributions with what has been released. Overall, it remains unclear to me whether AMIE post-training and the multi-agent setup is necessary to achieve the core results presented here given newer base models.

Response: We appreciate the reviewer's perspective regarding the necessity of the AMIE post-training and multi-agent setup. We agree that as base models like Gemini 2.5 advance, the performance gap narrows, suggesting that some of the complex scaffolding used in this study (conducted in November 2024) may become less critical for achieving similar results with modern models. We have revised the manuscript to transparently discuss this, emphasizing that our specific architecture was simply helpful to enable high-quality conversations at the time of the study, rather than necessarily a generalizable solution. Further research is needed to understand whether the benefits from methods described in this study (the medical

post-training, the dual agent architecture, the dynamic retrieval) are additive or redundant with the benefits offered by stronger and more capable base models.

We provide the added Discussion sections below for ease of reference (which also address the prior two comments):

"Our ablation analysis (Section A.17) further underscores this rapid evolution with Gemini 2.5 Flash substantially improving conversational performance over the AMIE system built on Gemini 1.5 Flash. For AMIE, with the exception of a few criteria, the agent scaffolding significantly improved the agent's history taking (Table A.9). In contrast, when this scaffolding was provided to Gemini 2.5 Flash, there was little to no improvement in our auto-evaluated simulations. While our Mx agent-specific ablation analysis (Section A.2) quantitatively demonstrates the value of agentic guideline retrieval and reasoning leveraging long-context capabilities of base models, taken together, our auto-evaluated ablations challenge the strict necessity of certain aspects of post-training and multi-agent setups as base models continue to improve. The objective of this research was not to propose that a particular configuration of post-training and agentic scaffolding might be indelibly required in light of the rapid development and improvement of base models. Rather, the durable contribution of this research is in the evaluation paradigm, rigorous ablation techniques and careful assessment of safety-critical longitudinal capabilities of AI systems in a critical task for the practice of medicine."

"We notably observed that contemporary foundation models, including Gemini 2.5 Pro, o3, and GPT-5, achieve performance largely comparable to the AMIE system built on Gemini 1.5 Flash (Table A.13). This indicates that the relative performance gain provided by the AMIE system is modulated as the underlying foundation models become more capable. However, unlike base models which were evaluated with medication labels provided directly in the context, AMIE uses dynamic retrieval to actively search for relevant information. This mimics a more realistic and challenging clinical workflow and remains a critical component of a deployable clinical system."

We believe that the increased performance of newer generations of base model is expected given the rapid pace of progress in the field, and that this development does not impede our central contribution of demonstrating, for the first time, physician-level management reasoning capabilities of an AI system (as an evaluation at a snapshot in time).

Referee #3 (Remarks on code availability):

Detailed code is not shared.

Response: Thank you for your response. We apologize for not being able to release training data or model weights due to commercial reasons. We believe we have provided sufficient detail to enable others to replicate our work using an open-source model. In Appendix A.1 to A.9 we list prompts and detailed methods for the Dialogue and Mx Agent.

Table 1. Evidence for the capabilities of our agent architecture compared to other models and humans.

<table>
<tr><th>Capability</th><th>Evidence + Location in paper</th><th>Compared to?</th><th>Detailed Explanation</th></tr>
<tr><td>Medication reasoning</td><td>RxQA benchmark

(Table A.13)</td><td>Gemini 1.5 Flash baseline (which AMIE was built on in this paper)</td><td>AMIE outperforms the relevant baseline significantly in the closed book setting.

OpenFDA, closed book (+10.9):
AMIE: 59.0 (53.4, 64.6)
Gemini 1.5 Flash: 48.1 (42.3, 53.7)

BNF, closed book (+8.3):
AMIE: 44.3 (38.4, 49.6)
Gemini 1.5 Flash: 36.0 (30.6, 41.4)

AMIE shows some performance gain in the open-book setting, though the gap is more narrow and not statistically significant. Importantly, in the open-book setting, AMIE uses dynamic retrieval of medication labels rather than manually putting the correct label(s) in context, which reflect the real world setting where correct labels are unknown.

OpenFDA, open book (+3.0):
AMIE: 72.0 (66.9, 77.1)
Gemini 1.5 Flash: 69.0 (63.8, 74.2)

BNF, open book (+0.8):
AMIE: 58.7 (53.1, 64.2)
Gemini 1.5 Flash: 57.9 (52.1, 63.3)

Table A.13 – AMIE with dynamic retrieval performs on par with other public models (DeepSeek, o-3, GPT-5) evaluated with ground truth drug labels provided in context (= no dynamic retrieval). Note that two generations of models were evaluated in this paper: Gemini 1.5 (used for the OSCE study) and Gemini 2.0/2.5 (evaluated at rebuttal time). All non-Google models were released after Gemini 2.0. Below is a table with release dates for these models:

<table>
<tr><th>Model</th><th>Release date</th></tr>
<tr><td>Gemini (1.5 Flash)</td><td>5/14/2024</td></tr>
<tr><td>AMIE Mx – OSCE study launch</td><td>11/1/2024 (internal)</td></tr>
<tr><td>Gemini (2.0 Flash)</td><td>12/11/2024</td></tr>
<tr><td>DeepSeek-V3</td><td>12/26/2024</td></tr>
<tr><td>o3-mini</td><td>1/31/2025</td></tr>
<tr><td>AMIE (1.5 Flash)</td><td>3/6/2025 (public)</td></tr>
</table>
</td></tr>
</table>

<table>
<tr>
<td></td>
<td></td>
<td></td>
<td>
<table>
<tr><td>o3</td><td>4/16/2025</td></tr>
<tr><td>Gemini (2.5 Pro)</td><td>05/06/2025</td></tr>
<tr><td>Gemini (2.5 Flash)</td><td>6/17/2025</td></tr>
<tr><td>GPT-5</td><td>8/7/2025</td></tr>
</table>
</td>
</tr>
<tr>
<td>Management Reasoning</td>
<td>OSCE Study

(Fig 5, Fig 6, Fig A.30, Fig A.31)</td>
<td>PCPs, no other models</td>
<td>Fig 5: Management plan quality
Across the 3 visits, specialist clinician evaluators rated AMIE’s management as more appropriate, precise, and aligned with guidelines than PCPs.

Fig 6: Management reasoning features
Across the 3 visits, patient actors and specialist evaluators drastically preferred the management from AMIE for all criteria. This included things like how it enabled shared decision making, how it managed competing priorities, how it acted as a teacher and fostered the relationship, its prognostication, and more.

Fig A.30: Patient actor detailed results
Patient actors preferred the communication from AMIE over PCPs. It was superior for most criteria, and at least on par for all. Significant benefits of AMIE were seen in many criteria, such as listening to the patient, explaining the condition and treatment, and showing empathy.

Fig A.31: Specialist physician detailed ratings
Specialist physicians preferred the history taking of AMIE over PCPs for all criteria, including things like eliciting presenting complaints or past medical history, explaining clinical information clearly, and addressing patient concerns. DDx appropriateness was equivalent for both arms, while the specialists preferred AMIE’s management plans.</td>
</tr>
</table>

Table 2. Evidence for how components of the agent architecture contribute to overall performance.

**Component of agent architecture**	**Evidence + Location in paper**	**Detailed Explanation**
Overall	Ablation analysis<br><br>(Tables A.9, A.10)	**Summary:**<br><br>The multi-agent framework showed clear and consistent benefits. Both the Dialogue Agent and Mx Agent provided performance gains across nearly all domains when applied to Gemini 1.5 Flash and our post-trained version of it.<br><br>The post-trained model, while not superior in every domain over the relevant baseline, showed meaningful improvements across many of the key history-taking metrics and was a strong choice for the study.
Base model choice / post-training	Ablation analysis<br><br>(Tables A.9, A.10)	**AMIE vs Gemini 1.5 Flash**<br>Several key history-taking criteria are improved when comparing AMIE to the relevant base model at the time, regardless of the choice of agent harness. Some examples:<br><br>**Confirming patient understanding:**<br>No harness: (0.627 -> 0.697)<br>With harness: (0.773 -> 0.807)<br><br>**Family history:**<br>No harness: (0.643 -> 0.820)<br>With harness: (0.847 -> 0.900)<br><br>**Medication history:**<br>No harness: (0.907 -> 0.987)<br>With harness: (0.967 -> 0.990)<br><br>**Past medical history:**<br>No harness: (0.940 -> 0.990)<br>With harness: (0.990 -> 1.000)<br><br>While the ablation analysis suggests potential tradeoffs (with regressions on several criteria), it was reasonable to proceed with this checkpoint for the study. The general sentiment from internal clinicians performing manual testing of the agent prior to launching it in the study was that the history taking was significantly improved, which is corroborated by some of the specific performance gains we observe in the ablation analysis.
Dialogue agent	Ablation analysis<br><br>(Tables A.9, A.10, A.11)	**End-to-end effects of Dialogue & Mx Agent**<br><br>For all but one of the 25 criteria presented in the ablation analysis, AMIE with the scaffolding performs equally or better than the model alone, with large performance gaps for many criteria. Some examples:<br><br>**Confirms patient understanding:**<br>(0.697 -> 0.807)<br><br>**Elicits family history:**<br>(0.820 -> 0.900)<br><br>**DDx appropriateness:**<br>(0.830 -> 0.867)<br><br>**Follow-up appropriateness:**<br>(0.927 -> 0.960)

		**Escalation appropriateness:**<br>(0.850 -> 0.897)<br><br>**Mx appropriateness:**<br>(0.697 -> 0.723)<br><br>**Shared decision making:**<br>(0.873 -> 0.940)<br><br>**Monitoring & adjustment:**<br>(0.590 -> 0.730)<br><br>**Appropriate prognostication:**<br>(0.787 -> 0.860)<br><br>Similarly, the addition of just the Dialogue Agent is equal or better in 23 out of 25 criteria, and the addition of just the Mx agent is equal or better in 24 out of 25 criteria. While the combination of both agents performs best in many cases, there are some instances where just one of the agents alone outperforms the combination.<br><br>The benefit of agent scaffolding was not specific to the AMIE post-trained base model; similar benefits can be seen by applying the same scaffolding to Gemini 1.5 Flash, the corresponding base model at the time. Interestingly, this effect is not seen with a more recent base model, Gemini 2.5 Flash, likely in part because the new model is already nearly saturated in performance on many criteria in this evaluation.
Mx agent	Mx agent auto eval<br><br>(Figure A.1)	**Improved guideline alignment, intervention, investigation, and follow-up appropriateness.**<br><br>The addition of the Mx Agent is a key novelty of this paper compared to prior AMIE publications. For the first time, we demonstrate how long-context reasoning models can be applied to ground a live clinical conversation in an extensive corpus of medical guidelines.<br><br>To demonstrate that each component of the Mx Agent's architecture contributes positively to overall performance, we ran an extensive ablation ablation specifically for the Mx agent in isolation, reported in Figure A.1.<br><br>Our auto-evaluation procedure measured how often the generated management plans were preferred over clinician-authored reference plans (win-rate, N=4 samples). The data clearly supports our claim that each architectural addition—moving from basic unstructured reasoning, to structured reasoning, to adding ensemble refinement (drafting), and finally adding long-context retrieval—cumulatively contributes to performance.<br><br>Win-rates for each of 5 eval axes across 3 Mx agent configurations are provided below, demonstrating increased performance with each improvement to the Mx agent:<br><br>***1st metric:*** *Unstructured Chain-of-Thought, No Retrieval ->*<br>***2nd metric:*** *Structured Reasoning, No Retrieval ->*<br>***3rd metric:*** *Final Mx Agent: Structured Reasoning + 4 Drafts + 256k Tokens Retrieval)*<br><br>• **Best overall management plan win rate:** 0.82 -> 0.84 -> 0.94<br>• **Guideline alignment win rate:** 0.82 -> 0.83 -> 0.94<br>• **Investigation appropriateness win rate:** 0.82 -> 0.83 -> 0.93<br>• **Intervention appropriateness win rate:** 0.80 -> 0.83 -> 0.94<br>• **Follow-up appropriateness win rate:** 0.87 -> 0.89 -> 0.95<br><br>Because these results effectively isolate the value of the Mx agent design, these ablations were not repeated in the newly added ablation analysis covering the entire multi-agent system (Tables A.9, A.10, A.11)

Response to the Referees

[Response to R1]
[Response to R2]
[Response to R3]

List of documents

This document provides inline responses to comments from the three external referees. In addition, we also provide the following documents as part of the revision:

1. **Cover letter**
2. **Revised manuscript** with tracked changes (one color per referee) including edits from the first two rounds of revisions as well as additional elaboration on limitations in response to R1 in the latest set of comments
3. **Revised appendix** with tracked changes (one color per referee) corresponding to the edits from the first two rounds of revisions
4. **Detailed view of two sample scenarios** (107 pages, with separate table of contents; this document now also includes specialist physician gradings and patient actor gradings highlighted in blue font)
5. **Scenario details and AMIE output for all 120 scenarios** provided as a PDF (2483 pages total, including 29 pages for dedicated table of contents) and as a structured comma-separated values (CSV) file

Response to R1

Thank you for your response. I do not feel that my major concerns are fully addressed.

1) I think the reasoning trace issue is still a major one - errors in reasoning traces that still lead to the "right answer" means that there will definitely be cases where it leads to the wrong answer. If we are arguing that this will be used in disease management, this seems like an important thing to fully address.

Response: Thank you for sharing this concern. We acknowledge that situations where the system arrives at the proper conclusion, although the underlying reasoning chain can exhibit latent errors, are a reality of the AMIE system selected for evaluation in our OSCE study.

We wish to emphasize that our system was designed to mitigate such latent reasoning errors, as well as errors in the final management plan output. The primary mitigation approach consisted in explicit grounding of the model's reasoning in relevant clinical guidelines and structured generation with guideline citations to increase alignment with clinical authoritative knowledge. Our Mx agent auto-evaluation (Figure A.1) showed quantitatively that this approach helps to increase both the ability to retrieve and cite relevant guidelines (first row of plots) and the quality and guideline alignment of the final management plan output (second and third row of plots). This auto-evaluation was one factor informing the selection of the specific system configuration we chose to test in our OSCE study.

However, as correctly pointed out by the reviewer, the current system does not guarantee complete absence of latent reasoning errors.

We encourage future work to aim for further mitigation of such errors in latent reasoning traces. In early experiments for this work, we explored alternative architectures involving separate agent components to critique reasoning traces and verify individual guideline citations during inference time. Our early explorations revealed that such architectures incurred high computational and latency cost, while only resulting in marginal improvements in the final management plan output. These kinds of approaches, if successfully implemented in a low-latency manner, could be potential candidates for exploration in future work.

Lastly, we thank R1 for the important note that *"[if] we are arguing that this will be used in disease management, this seems like an important thing to fully address."* This is correct. As we explicitly note in prominent sections of the manuscript (e.g., abstract and conclusion), the system investigated in this research is *not* ready for real-world translation and requires further research to mitigate these and other issues, as well as prospective clinical studies to provide the real-world evidence needed before any real-world translation. This research is an art-of-the-possible demonstration of AMIE's capabilities and limitations in longitudinal

management reasoning via simulated settings with patient actors, and should be interpreted through that lens.

We have expanded our limitations as follows, to further emphasize this important aspect:

“We encourage future work to study reasoning traces of medical AI systems in a comprehensive and quantitative manner, and to test system architectures that further reduce errors in the latent reasoning traces. Such approaches could involve agentic components that proactively critique the system's reasoning traces and verify the correctness of guideline citations during inference time. While incurring additional computational or latency cost, such approaches, if implemented in an efficient manner, have the potential to further mitigate latent reasoning errors and are therefore suitable candidates for future work.

We emphasize that this research is an art-of-the-possible demonstration of AMIE’s capabilities and limitations in longitudinal management reasoning via simulated settings with patient actors. The system investigated in this research, while exhibiting promising capabilities, is not ready for real-world translation and requires further research to mitigate issues like latent reasoning errors, as well as prospective clinical studies to provide the real-world evidence needed before real-world translation.”

2) I'm still really unable to assess this manuscript without actually being able to see the scenarios/responses from the models and physician baselines. The two cases shown make me wary of whether or not there could be issues in the scenarios and baselines. Since this paper is making huge claims, it feels like there should be more transparency here. I would not use or suggest implementation of such a tool without more transparency. Such openness would also move the field forward.

Response: We thank the reviewer for this comment. To improve transparency, we have enclosed the following materials:

- **Full scenario details for all 120 scenarios**: In addition to the metadata enclosed in prior revisions, this includes patient background (name, sex, age, ethnic background, location, lifestyle, social and personal circumstances, occupational history, travel history, family history), as well as visit-specific scenario information for each of 3 visits per scenario, including the presenting complaint, initial presentation, context for the visit, information available at the beginning of the visit (including new lab or imaging results, etc), information to be shared to the doctor if the patient is explicitly asked, past medical history and surgical history, current medications, relevant previous medications, allergies and adverse reactions, patient concerns, expectations and wishes, and questions the patient wants to ask the doctor.
- **All AMIE outputs for all 120 scenarios:** For all 3 visits per scenario, we additionally include the AMIE-patient-actor conversation, as well as AMIE’s post-questionnaire responses.

These materials are provided as a separate PDF (2483 pages total, including 29 pages for dedicated table of contents) and as a structured comma-separated values (CSV) file. We confirm that we will make these materials available to the public in case of acceptance of our manuscript for publication.

A release of PCP output for all 120 scenarios, unfortunately, is infeasible in our particular setting as it would require additional contracting with each of the 20+ clinicians involved, to clear permissions for data release.

We believe that providing the full breadth of scenarios and AI responses helps to improve transparency and will be a valuable resource to move the field forward.

Finally, we would like to thank the reviewer for encouraging us to take this step which we believe will strengthen the contribution of this work.

Referee #1 (Remarks on code availability):

Previously reviewed the shared model, had issues with the model acting slow.

Response: As discussed in the previous revision, we acknowledge that inference latency was higher than optimal during the reviewer testing period. This was a practical limitation and primarily due to the use of shared, non-dedicated research endpoints which experience high latency variability and queuing delays. It was an artifact of the experimental infrastructure, not the model's architectural limitations. In a production environment with dedicated accelerator resources, these latency bottlenecks would be resolved to ensure a closer to real-time conversational experience.

Response to R2

The manuscript is substantially improved after the second revision, and I'd like to comment on the following aspects:

1. Based on the empirical findings in Section A.17 and A.18, it appears that advancements in newer foundation LLMs (e.g., Gemini-2.5-Flash) are the primary driver of performance gains on clinical tasks. In particular, later base LLMs without AMIE often outperform earlier LLMs augmented with AMIE, and the additional benefits of AMIE on top of newer LLMs (e.g., Gemini 2.5 Pro) tend to show diminishing returns, as evidenced by Table A.13. Although this observation may somewhat diminish the perceived contribution of AMIE itself, I believe it is still an important and useful finding, as it helps clarify where the gains are actually coming from and provides a more accurate picture of the respective roles of base-model progress and post-training / scaffolding.

Response: We thank the reviewer for this insightful comment. We agree that many of the benefits of the agentic harness (AMIE) show diminishing returns when applied to increasingly capable base models and we have elaborated on this in the discussion. Several factors likely contribute to this:

- **Native "thinking" capabilities:** The clinical reasoning elicited in our original agentic harness (optimized for the Gemini 1.5 family of models) via structured decoding has become an inherent feature of newer "thinking" models. Consequently, the additive benefit of explicitly guiding the model to reason before responding is reduced when the base model performs such thinking natively, unlike older models which proceed directly to outputting a response when prompted.
- **Optimization for older baselines:** The specific scaffolding used in this study was built and tuned on Gemini 1.5 Flash. It is possible that better tailoring the harness towards the remaining losses observed in the Gemini 2.5 models would show more benefit than this harness which was designed around a weaker model.

2. The OSCE scenarios added in A.16 span diverse and common diseases, and their full release would be a valuable contribution for developing and evaluating future medical AI systems. I encourage the authors to release the complete contents of all 120 OSCE scenarios (ideally presented in the same format as the two samples in the Supplementary Material) in addition to the metadata.

Response: We thank you for this comment. We will be releasing the full details of the 120 multi-visit OSCE scenarios along with this study, as well as AMIE outputs for all 120 scenarios.

These materials are provided as a separate PDF (2483 pages total, including 29 pages for dedicated table of contents) and as a structured comma-separated values (CSV) file. We

confirm that we will make these materials available to the public in case of acceptance of our manuscript for publication.

This newly enclosed PDF follows the same format as the previous PDF with detailed views of two sample scenarios.

3. On the evaluation methodology, the benchmarks and rubrics of (1) multi-visit OSCE study and (2) medication knowledge (RxQA) would be useful for improving disease management abilities of future models. The RxQA benchmark has been released publicly, and I encourage the authors to release full details of 120 OSCE scenarios in addition to their metadata.

Response: We thank you for this comment. We will be releasing the full details of the 120 multi-visit OSCE scenarios along with this study (see our more detailed response above).

Overall, I believe this work represents a meaningful step towards evaluating disease management abilities of AI systems, especially given the fact that previous works have largely focused on evaluating and advancing diagnostic abilities. The multi-visit OSCE design also aligns more closely with real-world clinical practice than previous benchmarks.

Response: Thank you for your response.

Referee #2 (Remarks on code availability):

Source code is not provided

Response: Thank you for your response. We apologize for not being able to release training data or model weights due to commercial reasons. We believe we have provided sufficient detail to enable others to replicate our work using an open-source model. In Appendix A.1 to A.9 we list prompts and detailed methods for the Dialogue and Mx Agent.

Response to R3

I commend the authors for engaging substantively with the feedback and think the manuscript has improved considerably -- I have no further comments.

Response: We appreciate R3's careful review of our responses and are glad to see that our revision was able to fully address the referee's comments.

Response to the Referees

Response to R1

Thank you for agreeing to release the 120 scenarios. I believe this will be an excellent contribution.

Response: Thank you for making this suggestion. We agree that this will be a useful contribution for the research community.

Response to R2

I have read through the authors' response to my comments as well as the other reviews, and I believe the concerns raised in previous rounds of review have been sufficiently addressed. Specifically, the authors have agreed to release all OSCE prompts and AMIE responses, and I found their discussion of the latent reasoning errors reasonable. Completely eliminating reasoning errors in language models is currently infeasible. Therefore, as long as the research provides insightful findings or useful data toward alleviating this issue and clearly flags this limitation, I consider it a solid contribution to this line of research. Building on this point, I believe the release of all OSCE prompts and AMIE responses will be valuable to future researchers seeking to investigate latent reasoning errors further.

Overall, I consider the current manuscript ready for publication.

Response: Thank you for your comment. We appreciate the careful review of our response in the last revision, and are delighted to hear that the OSCE scenario details and AMIE output are received as a welcome contribution for the research community.

Response to R3

The authors are not able to release training data or model weights due to commercial reasons.

Referee #3 (Remarks to the Author):

I believe the authors have engaged thoughtfully with the reviewer feedback and I have no further comments.

Response: We appreciate R3’s careful review and are glad that our revisions addressed the referee’s comments.
